# Alcohol/Water-Relay Sulfurative Oxygenation of Vinyl Bromides Enabled by Photoinduced EDA Complexes

**DOI:** 10.1021/acs.orglett.6c03155

**Published:** 2026-08-14

**Authors:** Indrajit Karmakar, Ling-You Chang, Ci-Yang Sun, Chien-Wei Chiang, Chin-Fa Lee

**Affiliations:** † Department of Chemistry, 34916 National Chung Hsing University, Taichung 40227, Taiwan (R.O.C.); ‡ *i*-Center for Advanced Science and Technology (iCAST), 34916 National Chung Hsing University, Taichung 40227, Taiwan (R.O.C.); § Innovation and Development Center of Sustainable Agriculture (IDCSA), 34916 National Chung Hsing University, Taichung 40227, Taiwan (R.O.C.); ∥ Department of Chemistry, Soochow University, No. 70 Linhsi Road, Shihlin District, Taipei 111002, Taiwan (R.O.C.)

## Abstract

The direct conversion of vinyl halides into oxygenated sulfur-containing carbonyl compounds using water as the oxygen source remains challenging because it requires coordinated C-halogen activation, radical capture, oxygen incorporation, and C–S bond formation under mild conditions. Here we report a photoinduced electron donor–acceptor complex strategy that enables the sulfurative oxygenation of vinyl bromides with thiols in an EtOH/H_2_O medium under metal- and oxidant-free conditions. In this process, thiolate formation triggers EDA complex assembly with vinyl bromides, and light excitation initiates C–Br fragmentation to generate a styrenyl radical that is intercepted through an alcohol/water relay. The reaction provides α-thioaryl ketones in up to 81% yield across 32 examples and can be performed on a gram scale. Mechanistic experiments, including radical trapping, EPR, H_2_^18^O labeling, UV–vis spectroscopy, NMR titration, cyclic voltammetry, and DFT calculations, support a radical-polar pathway involving ethanol trapping, water addition, sulfur transfer, and tautomerization.

## Graphical abstract

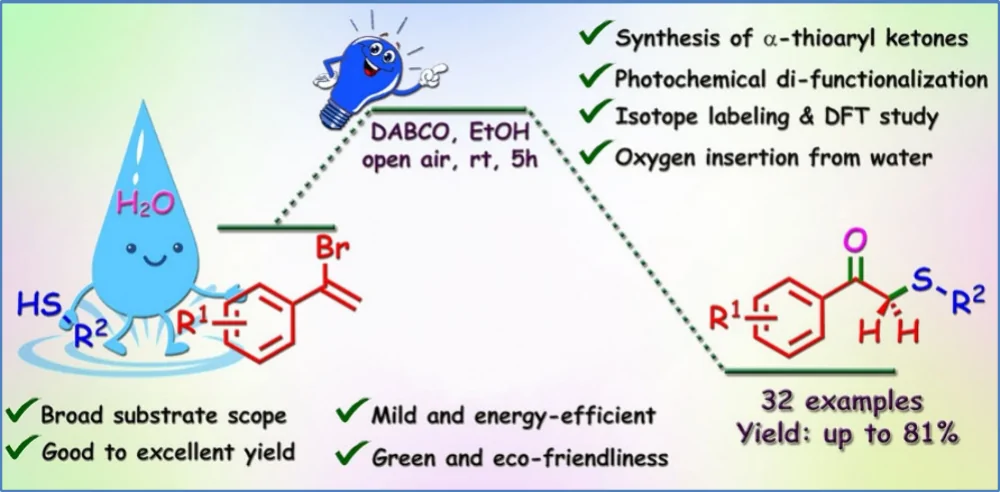

Molecules bearing sulfur atoms are widely recognized for their distinctive chemical behavior and their essential roles in numerous biological and chemical processes.^1^ α-Thioaryl ketones represent a valuable class of sulfur-containing compounds widely recognized for their roles in organic and medicinal chemistry. Their presence in biologically active molecules, along with their versatility as key intermediates, makes them important building blocks for the synthesis of structurally diverse and functionally relevant compounds.^2^ Among sulfur-containing compounds, α-thioaryl ketones constitute a prominent class of bioactive molecules exhibiting diverse pharmacological activities. These compounds have demonstrated significant anticancer and antitumor potential, primarily by suppressing tumor cell proliferation through selective interactions with key biological macromolecules, including proteins and enzymes, as well as by disrupting cell-cycle progression and inducing apoptosis.^3^ In addition to their anticancer properties, α-thioaryl ketones have also shown antiviral activity by inhibiting viral replication through modulation of essential enzymatic processes, such as serine protease activity, thereby limiting viral propagation.^4^ Several representative drugs and bioactive molecules featuring the α-thioaryl ketone framework are depicted in Figure 1.

**Figure 1:**
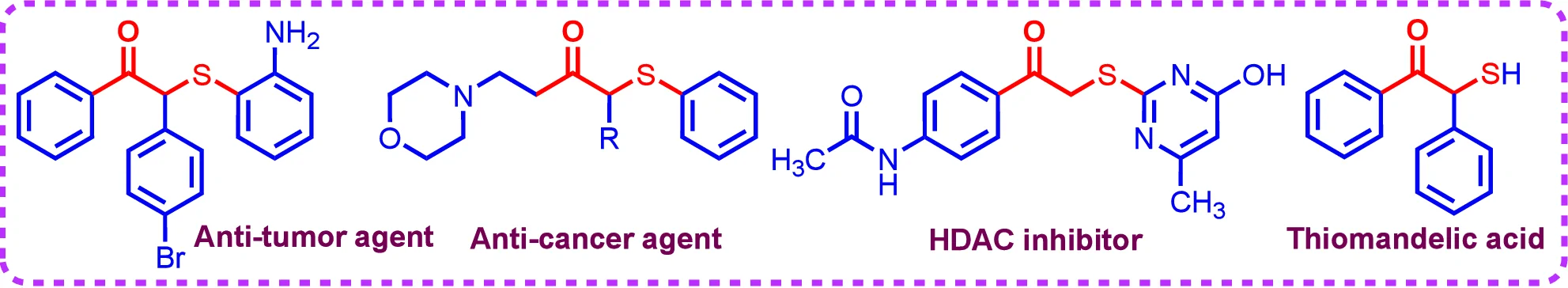
Representative examples of drugs and bioactive α-thioaryl ketones.

Vinyl bromides are readily available and bench-stable precursors, yet their direct use in radical-polar oxygenative difunctionalization remains underdeveloped. Conventional approaches to α-thioaryl ketones commonly rely on prefunctionalized carbonyl surrogates, transition-metal catalysis, strong oxidants, or thermally demanding conditions (Scheme 1).^5–7^ Visible-light photochemistry has emerged as a sustainable approach in organic synthesis.^8^ Among its applications, difunctionalization reactions efficiently install two functional groups in a single step, enabling rapid access to value-added molecules.^9^ Developing a mild process that simultaneously activates the C–Br bond, incorporates oxygen from water, and forges a C–S bond would provide a distinct entry to α-sulfurated carbonyl compounds while addressing a broader challenge in the difunctionalization of vinyl halides.

**Scheme 1:**
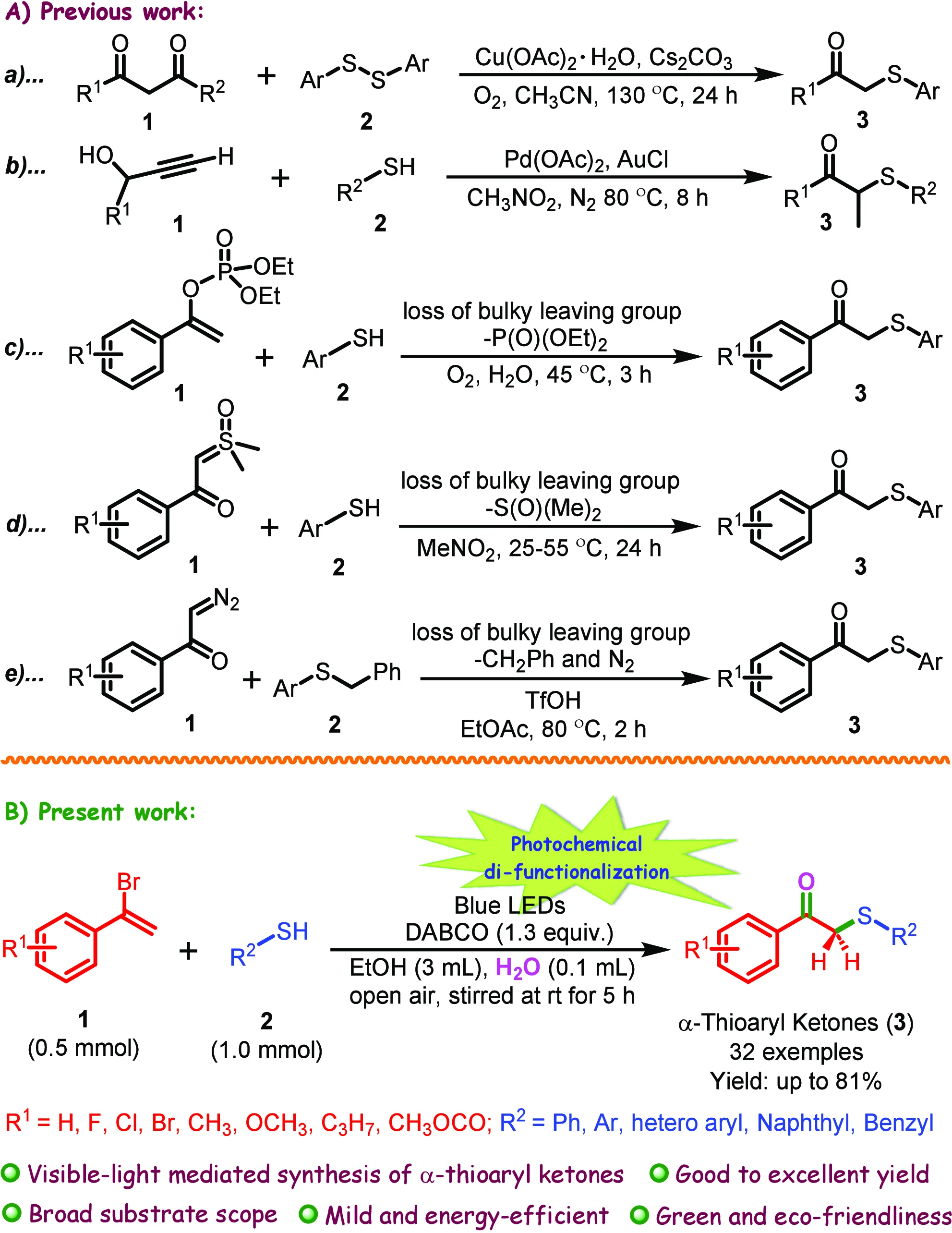
Previous Approaches and the Present Photoinduced EDA-Complex-Enabled Sulfurative Oxygenation of Vinyl Bromides with Water as the Oxygen source

We envisioned that thiolate species, generated in situ from thiols and a mild base, could engage vinyl bromides through donor–acceptor association. Photoexcitation of this EDA complex would bypass direct oxidation of the vinyl bromide and trigger C–Br fragmentation, generating a styrenyl radical that could be directed into an alcohol/water-assisted oxygenation sequence. To our delight,^10^ here we demonstrate this concept in the metal- and oxidant-free sulfurative oxygenation of vinyl bromides, furnishing α-thioaryl ketones under ambient conditions. Mechanistic studies and DFT calculations^11^ reveal an ethanol/water relay that connects EDA-mediated radical generation with downstream sulfur transfer and tautomerization.

We selected (1-bromovinyl)­benzene **1a** and benzenethiol **2a** as the model substrates and identified DABCO in EtOH/H_2_O under blue LED irradiation as the optimal system, affording **3aa** in 81% isolated yield within 5 h (Table 1, entry 1). Control reactions established the essential roles of light, base, and water: no product was detected in the absence of light, DABCO, or water, whereas reactions under O_2_ and N_2_ gave comparable outcomes, indicating that molecular oxygen is not the oxygen source (Table 1, entries 2–5). Solvent and base/additive screening further highlighted the unique efficiency of the aqueous ethanol-DABCO combination, with other alcohols, organic solvents, water alone, and inorganic bases giving lower yields (Table 1, entries 6–8). These results define a mild and operationally simple protocol using visible light, aqueous ethanol, and a stoichiometric organic base under ambient conditions.

**Table 1:**
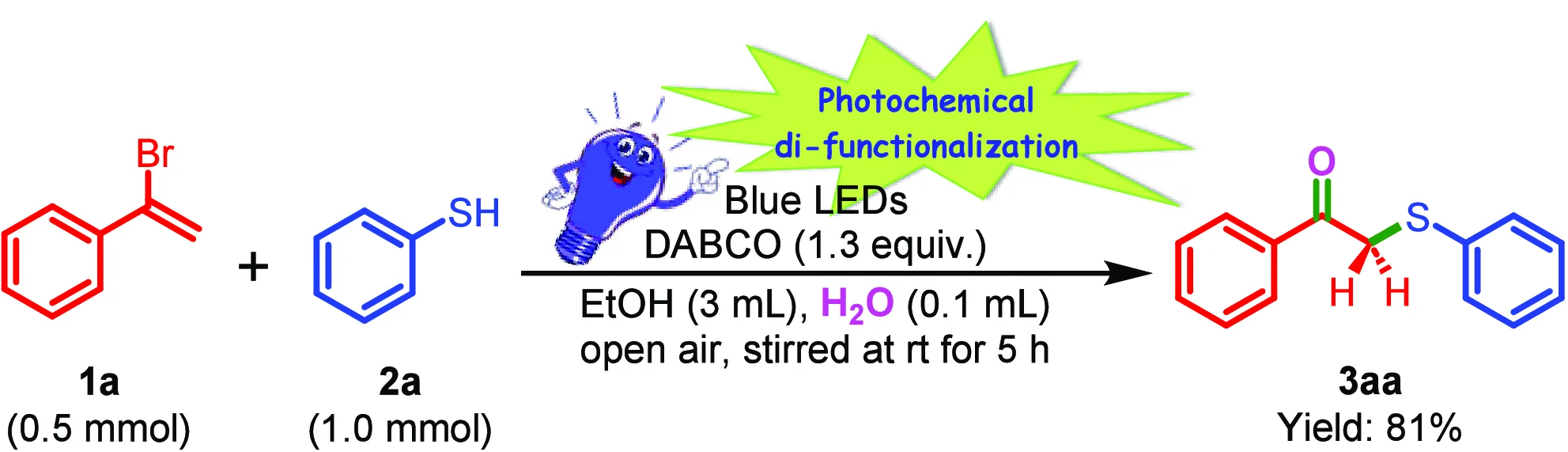
Optimization of Reaction Conditions^a^ ^,^ ^b^

Entry	Conditions	Yield (%)^a^ ^,^ ^b^
1	None	81
2	No catalyst, light; no catalyst	
3	Using anhydrous EtOH	
4	EtOH:H_2_O (3.0:0.5); in air; O_2_; N_2_	80; 80; 67
5	White LEDs; Sunlight; No light	76; 42; –
6	Using MeOH; DMSO; Acetone; Toluene; EtOAc; Et_2_O; Hexane; THF; H_2_O, and each solvent contained 0.1 mL of H_2_O	45; 18; 46; 56; 59; 54; 61; 11; 36
7	DABCO (0.25 mmol); DABCO (1.0 mmol)	54; 61
8	DBU; Et_3_N; ^*i*^Pr_2_NEt; K_2_CO_3_; NaHCO_3_	54; 50; 45; 65; 69

a Reaction conditions: (1-bromovinyl)­benzene (**1a**, 0.5 mmol) was reacted with benzenethiol (**2a**, 1.0 mmol) at room temperature (25–28 °C) in the presence of various bases/additives and solvent systems under different visible-light sources and atmospheric conditions.

b Isolated yields.

With the optimized conditions established, we evaluated the generality of this photochemical difunctionalization using a range of thiol and vinyl bromide coupling partners (Table 2). The reaction first showed good compatibility with halogenated aromatic thiols. Chloro- and bromo-substituted benzenethiols at different positions, including **2b**–**2e**, reacted smoothly with **1a** to afford the corresponding α-thioaryl ketones **3ab**–**3ae** in 66–71% yields. Electron-rich aryl thiols were also suitable substrates. Mono- and dialkyl-substituted thiols **2f**–**2m**, bearing methyl, isopropyl, *tert*-butyl, and dimethyl groups, delivered products **3af**-**3am** in 59–73% yields, indicating that moderate steric variation around the thiol component is tolerated. Strongly electron-donating hydroxy- and methoxy-substituted thiols **2n**–**2r** furnished **3an**–**3ar** in 61–73% yields, suggesting that competitive oxidation or side reactions of these electron-rich substrates are not dominant under the optimized conditions. An electron-deficient thiol bearing a trifluoromethyl group, **2s**, also participated efficiently to give **3as** in 67% yield. The protocol could further be extended to naphthyl and benzyl thiols, with naphthalene-2-thiol **2t**, phenylmethanethiol **2u**, and (2-chlorophenyl)­methanethiol **2v** affording products **3at**–**3av** in 56–74% yields.

**Table 2:**
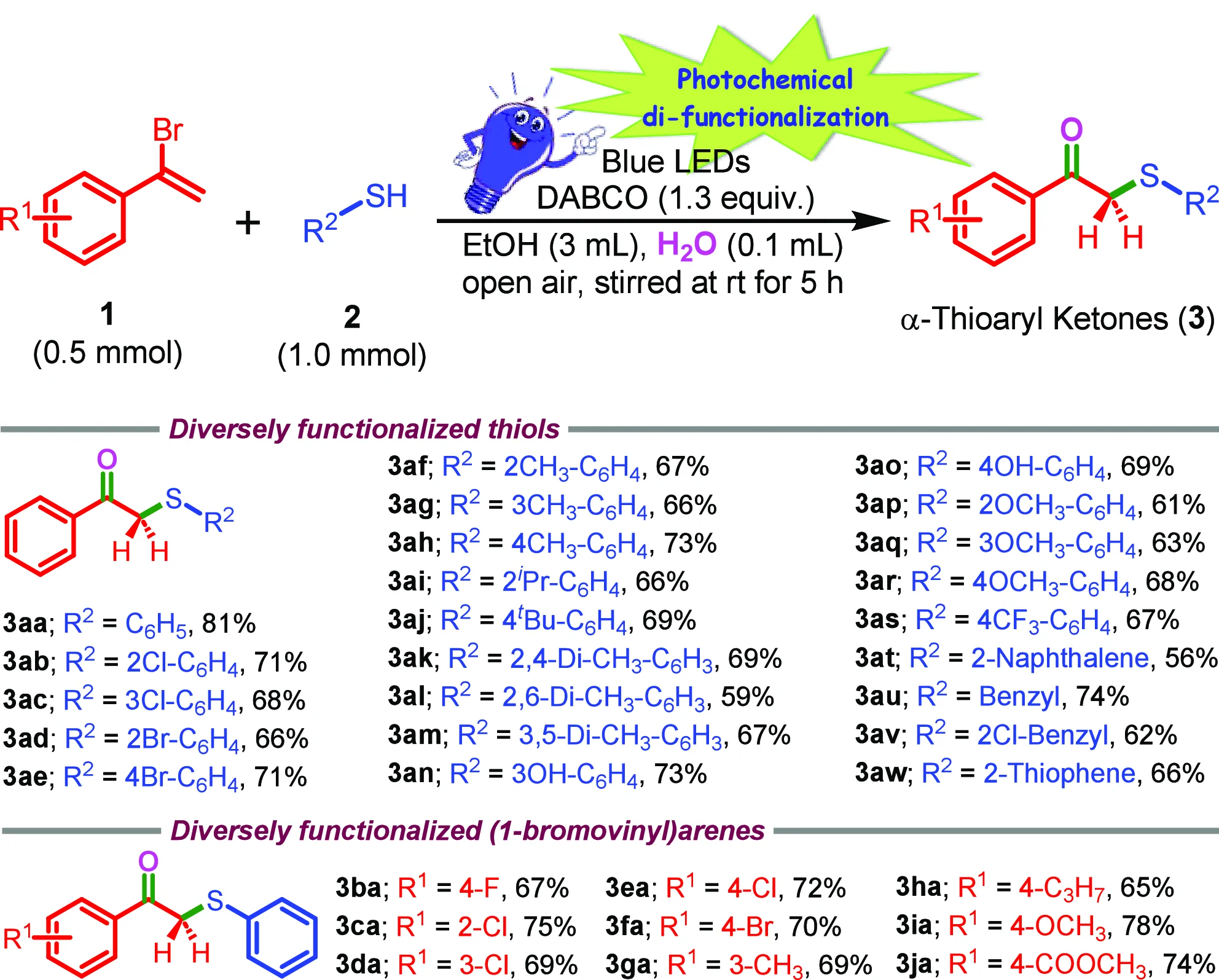
Substrate Scope of α-Thioaryl Ketones^a^ ^,^ ^b^

a Reaction conditions: A mixture of (1-bromovinyl)­arenes (**1**, 0.5 mmol), thiols (**2**, 1.0 mmol), and DABCO (1.3 equiv) in EtOH/H_2_O (3.0:0.1 mL) was stirred under blue LED irradiation at room temperature (25–28 °C) in open air for 5 h.

b Isolated yields.

We next examined the scope of the vinyl bromide component. Halogen-substituted (1-bromovinyl)­arenes **1b**–**1f**, including fluoro-, chloro-, and bromo-substituted derivatives, reacted efficiently with benzenethiol **2a** to furnish **3ba**–**3fa** in 67–75% yields. These halogenated products also retain useful synthetic handles for further derivatization. Electron-rich and electron-poor vinyl bromides were likewise compatible: methyl-, propyl-, methoxy-, and ester-substituted substrates **1g**–**1j** provided the corresponding α-thioaryl ketones **3ga**–**3ja** in 65–78% yields. Overall, these results demonstrate that the reaction accommodates electronically and sterically diverse thiols and vinyl bromides, while maintaining useful functional-group tolerance under mild, metal- and oxidant-free photochemical conditions. All α-thioaryl ketones were synthesized and fully characterized (*see* Supporting Information), further underscoring the broad substrate scope, functional group tolerance, and robustness of the method.

To demonstrate the practicality of the method, a gram-scale experiment was conducted using the model substrate (5.0 mmol, 10-fold increase). The reaction proceeded smoothly under the standard conditions, affording **3aa** in 76% isolated yield (Scheme 2). Despite a slight decrease in yield compared with the 0.5 mmol scale, the reaction maintained good efficiency with only minor adjustments in reaction time, demonstrating its scalability.

**Scheme 2:**
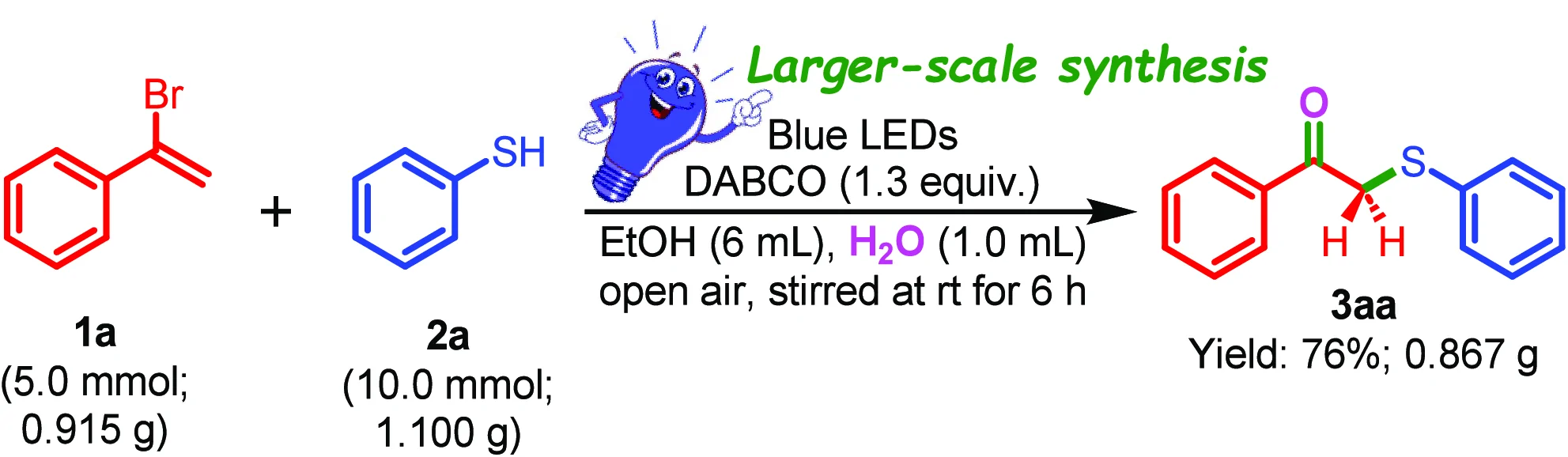
Larger-Scale Synthetic Applications

To interrogate the origin of oxygen incorporation and the nature of the photoinduced activation event, we performed a series of mechanistic experiments (Scheme 3). Radical scavengers including TEMPO, *p*-benzoquinone, and DMPO substantially suppressed product formation, and HRMS detection of a TEMPO-trapped sulfur radical adduct supports the generation of thiol-derived radical species. EPR analysis further confirmed radical formation under the photochemical conditions. Reactions under nitrogen remained productive, whereas rigorously anhydrous ethanol completely shut down product formation. The H_2_^18^O-labeling experiment led to ^18^O incorporation into the product, establishing water as the oxygen donor. These observations indicate that product formation involves radical intermediates and water-dependent oxygen incorporation rather than oxidation by molecular oxygen. Control experiments with **1a** and diphenyl disulfide, diphenyl diselenide, or aniline showed no reaction.

**Scheme 3:**
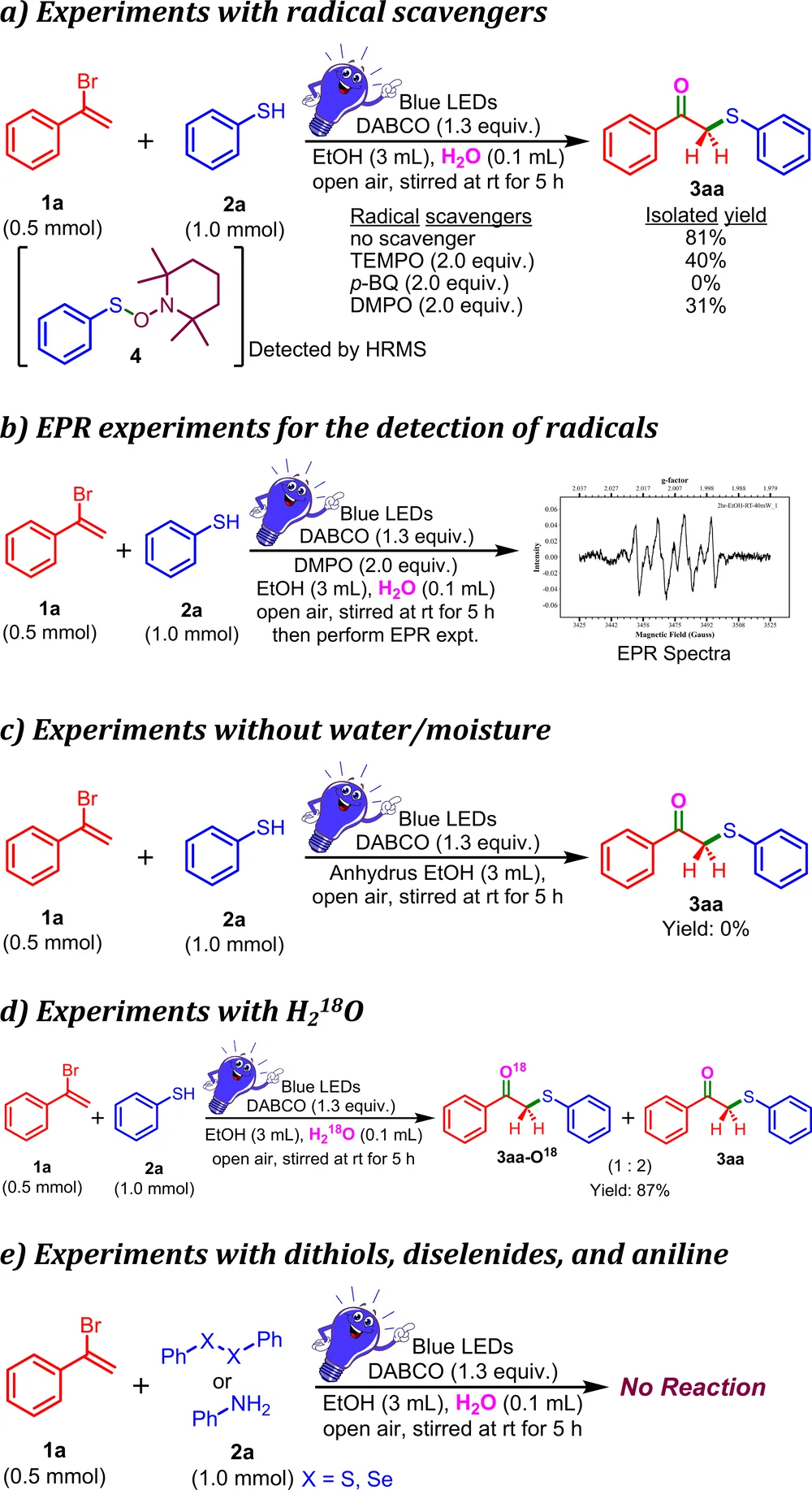
Control Experiments

Based on the control experiments (Scheme 3), cyclic voltammetry, spectroscopic studies, and DFT calculations (Figure 2), a photoinduced EDA-complex-mediated pathway is proposed (Scheme 4).^12^ Cyclic voltammetry (Figure S1) showed no obvious oxidation peak for (1-bromovinyl)­benzene **1a** or significant change upon addition of DABCO, indicating that direct oxidation of **1a** and appreciable interaction between these components are unlikely. Benzenethiol **2a** exhibited an oxidation peak at +1.4 V vs Ag/Ag^+^, which shifted to +0.4 V upon addition of DABCO; DABCO alone was oxidized at approximately +1.1 V. These observations suggest that DABCO promotes formation of thiolate **2a** ^–^, a more readily oxidizable electron donor.

**Scheme 4:**
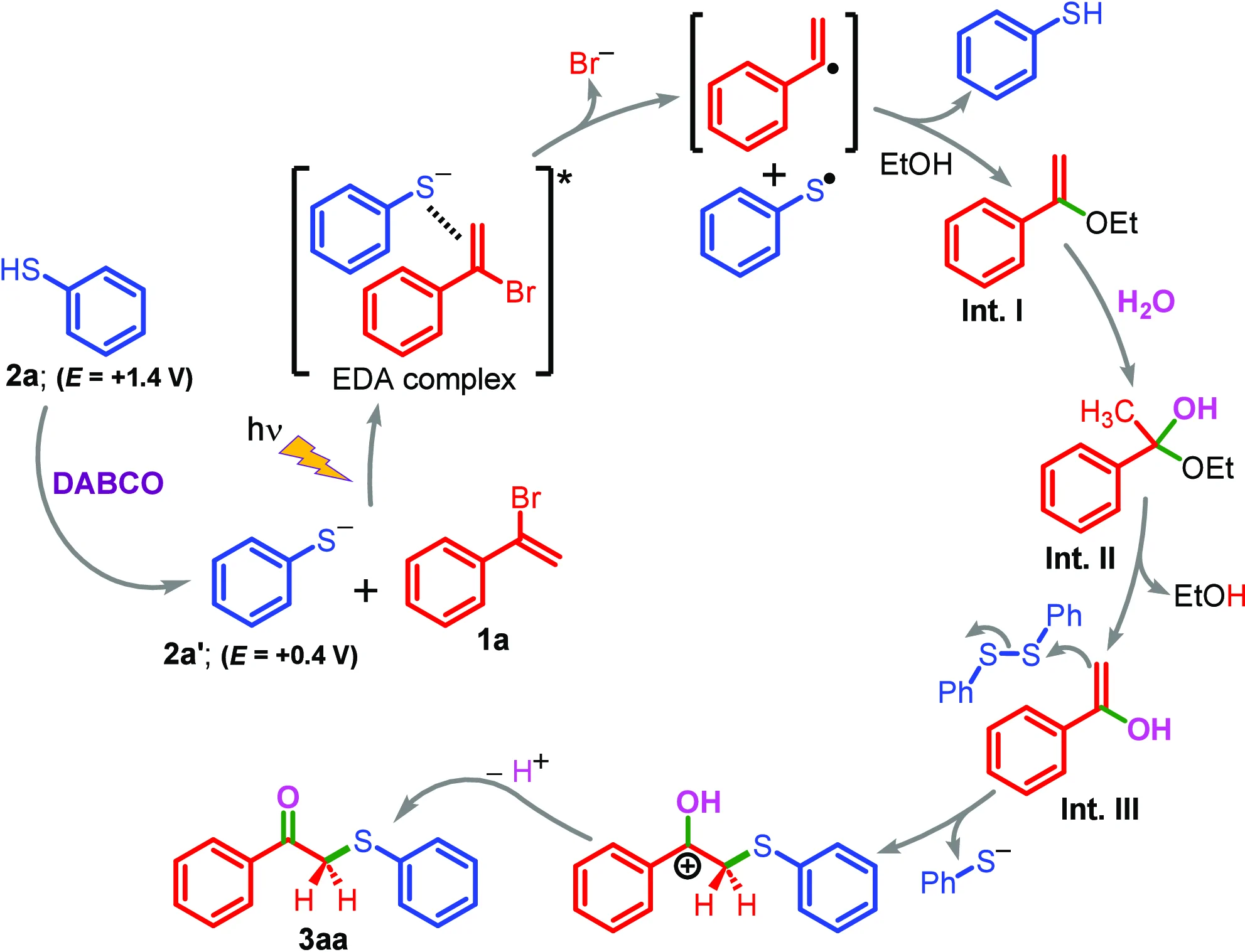
Proposed Photoinduced EDA-Complex Pathway Involving C–Br Fragmentation and Alcohol/Water-Relay Sulfurative Oxygenation

**Figure 2:**
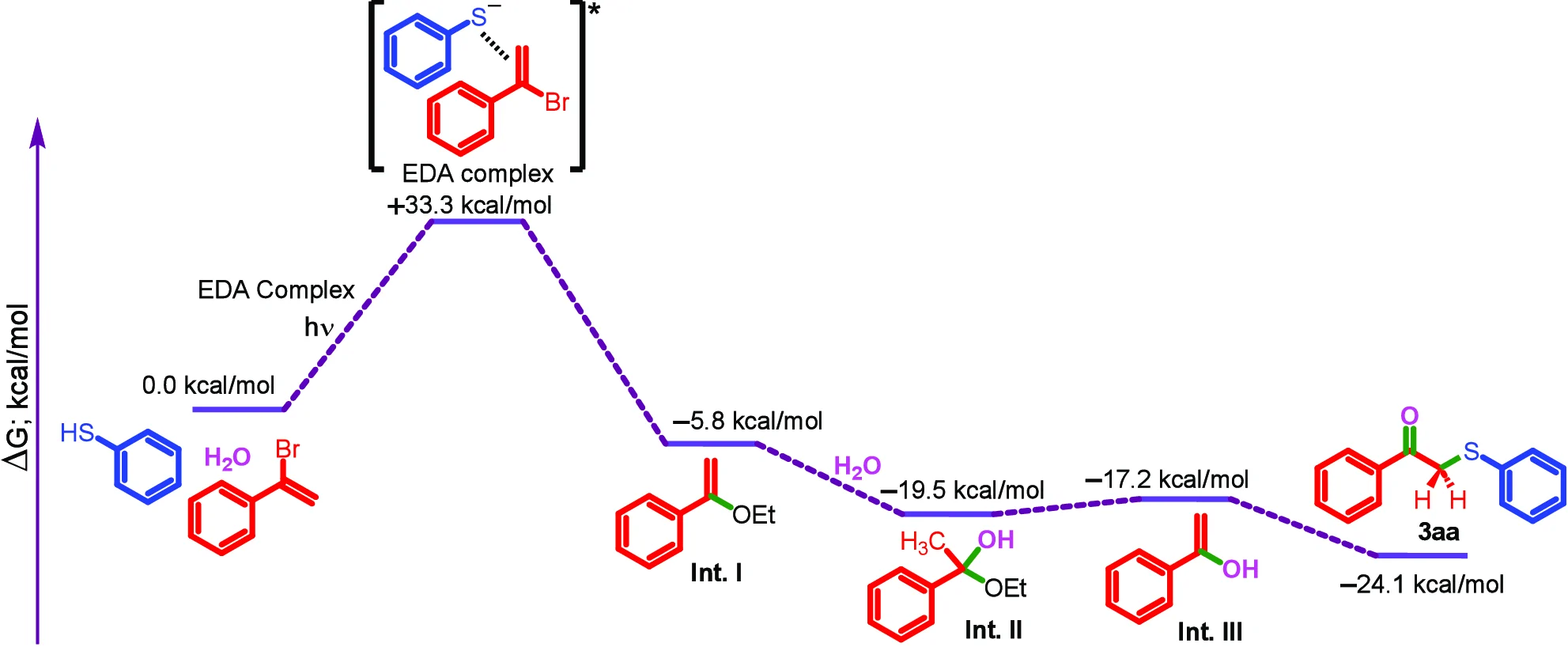
Computed free-energy profile for the alcohol/water-assisted conversion of vinyl bromide **1a** to α-thioaryl ketone **3aa**.

Interaction of **2a** ^–^ with vinyl bromide **1a** generates an electron donor–acceptor complex, as supported by the chemical-shift changes observed during ^1^H NMR titration (Figures S4 and S5) and the appearance of a broad absorption band in the UV–vis spectrum (Figure S3). Visible-light excitation raises this complex to a state calculated to lie 33.3 kcal mol^–1^ above the ground-state complex. Subsequent intracomplex electron transfer and C–Br fragmentation release bromide and generate a styrenyl radical^13^ together with a thiyl radical.

DFT calculations support the subsequent solvent-assisted conversion of the styrenyl radical. Trapping by ethanol affords ethoxy-substituted alkene **Int I** (−5.8 kcal mol^–1^), followed by favorable hydration to **Int II** (−19.5 kcal mol^–1^). Subsequent elimination of ethanol produces enol **Int III** (−17.2 kcal mol^–1^). Sulfur transfer from diphenyl disulfide and/or phenylthiolate then gives a sulfur-substituted enolic intermediate, which undergoes deprotonation and tautomerization to furnish α-thioaryl ketone **3aa**, the most stable species along the proposed pathway (−24.1 kcal mol^–1^). Collectively, the electrochemical, spectroscopic, labeling, radical-trapping, EPR, and computational results support DABCO-enabled thiolate formation and EDA-complex assembly. Photoexcitation induces C–Br cleavage, after which the resulting styrenyl radical undergoes ethanol/water-assisted oxygenation, sulfur transfer, and tautomerization to afford **3aa**.

In conclusion, we have developed a photoinduced EDA-complex-enabled sulfurative oxygenation of vinyl bromides with thiols using water as the oxygen source. The reaction proceeds under metal- and oxidant-free conditions in aqueous ethanol at ambient temperature and provides α-thioaryl ketones in synthetically useful yields across a range of thiol and vinyl bromide partners. Mechanistic experiments and DFT calculations support a radical-polar pathway in which thiolate-enabled EDA complexation activates the vinyl bromide C–Br bond under light irradiation, while an alcohol/water relay channels the resulting styrenyl radical into oxygenated sulfur-containing carbonyl products. This strategy establishes a mechanistically distinct platform for sulfurative oxygenation using water as the terminal oxygen source and may guide the design of related radical-polar difunctionalization reactions of vinyl halides.

## Supplementary Material



## Data Availability

The data underlying this study are available in the published Article and its Supporting Information.

## Notes

General information, experimental procedures (including gram-scale synthesis), characterization data for all compounds (**3aa**–**3aw** and **3ba**–**3ja**; 32 derivatives), ^1^H, ^13^C, and ^19^F NMR spectra (**3as** and **3ba**), HRMS of adduct **4**, and **3aa-O** ^**18**^, cyclic voltammetry, UV–vis, ^1^H NMR titration, and DFT computational data (PDF)
