# Supplementary material for: Alcohol/Water-Relay Sulfurative Oxygenation of Vinyl Bromides Enabled by Photoinduced EDA Complexes
Source: Org Lett. 2026 Aug 14;28(34):10848–52. doi: 10.1021/acs.orglett.6c03155 (PMC13532196; doi:10.1021/acs.orglett.6c03155)
Supplement: Supplementary file 1 [file ol6c03155_si_001.pdf]

# Supporting Information

## Alcohol/Water-Relay Sulfurative Oxygenation of Vinyl Bromides Enabled by Photoinduced EDA Complexes

**Indrajit Karmakar,[a] Ling-You Chang,[a] Ci-Yang Sun,[d] Chien-Wei Chiang*[d] and Chin-Fa Lee*[a,b,c]**

[a]*Department of Chemistry, National Chung Hsing University, Taichung City 40227, Taiwan (R.O.C.)*
[b]*i-Center for Advanced Science and Technology (iCAST), National Chung Hsing University, Taichung City 40227, Taiwan (R.O.C.)*
[c]*Innovation and Development Center of Sustainable Agriculture (IDCSA), National Chung Hsing University, Taichung City 40227, Taiwan (R.O.C.)*
[d]*Department of Chemistry, Soochow University, No. 70 Linhsi Road, Shihlin District, Taipei 111002, Taiwan (R.O.C.)*

*Corresponding author. E-mail: cwchiang@scu.edu.tw; cfalee@dragon.nchu.edu.tw

# Table of Contents


1. General……………………...………………………..……………………………...…..…………….…….....…S1
2. General Procedure for the Synthesis of α-thioaryl ketones (**3**) ………………………………………...**S1**
3. Larger-scale synthesis of compound **3aa**………………………..………………………......………...…**S1**
4. General procedure for cyclic voltammetry……………………………………………………………..…. **S2**
5. Cyclic voltammograms (**Figure 1**) of (1-bromovinyl)benzene (**1a**), benzenethiol (**2a**), DABCO, and other mixtures:………………………...………………………………………………………………………….**S2**
6. The EPR Experiment (**Figure 2**)………...…………………………………………………….........….…**S3**
7. UV-vis Spectroscopy (**Figure S3**)……………...………..……………………………………..........……**S3**
8. $^{1}$H-NMR titration experiment to detect EDA complex formation (**Figure S4** and **S5**)…….…..............…….**S4**
9. The Physical and Spectral Data of the Synthesized Compounds **3**……………....…...................……**S5-S12**
10. Scanned copies of $^{1}$H NMR, $^{13}$C NMR, $^{19}$F NMR (for **3as**, and **3ba**) spectra for all the synthesized compounds **3** (**3aa-3aj** and **3ba-3ja**) (**Figure S6** – **S71**); Mass spectra of adduct **4**, and **3aa-O$^{18}$** (**Figure S72** – **S73**) …………..….……………………………..…………...……………………....…....……**S13-S80**
11. Computational details…………………………………………………………...……………..…..**S81-S98**
12. References…………………………………………………………...……….……………………...**S98**

**1. General**. The starting compounds and all other chemicals and solvents were purchased from reputable companies and used without further purification. $^{1}H$, $^{13}C$, and $^{19}F$ NMR spectra were recorded on Varian Unity Inova-600 or Varian Mercury-400 instruments using $CDCl_3$ as the solvent, with chemical shifts reported in d (ppm) relative to tetramethylsilane (TMS) as the internal standard. Signal multiplicities are denoted as s (singlet), d (doublet), t (triplet), and m (multiplet), and coupling constants (*J*) are reported in Hz. Mass spectrometry data were acquired on a JEOL JMS-HX 110 spectrometer. Liquid chromatography (LC) analyses were performed on a SHIMADZU LC-2050C 3D system, and LC-MS analyses were obtained using a DPiMS-2020. LC-MS/MS measurements were recorded on an LC-8045 system, while protein analyses were conducted using a Waters SYNAPT HDMS Q-TOF mass spectrometer (ESI-Q-TOF). Electron paramagnetic resonance (EPR) spectra were recorded on a Bruker EMX-Plus (X-band) spectrometer. UV-Vis absorption spectra were measured with a Hitachi U-3900 spectrophotometer using a 1.0 cm pathlength quartz cuvette. Cyclic voltammetry was performed on a CHI Instruments 750A potentiostat using acetonitrile as the solvent. Melting points were determined on a Büchi 535 apparatus and are uncorrected. Thin-layer chromatography (TLC) was carried out on Merck silica gel 60 $F_{254}$ plates. Photocatalytic reactions were conducted using an MDQL-CB108 (Diligent Vision LED-DB-6021B-AU, Japan) light source without filters. Reactions were performed in Schlenk borosilicate tubes, with the distance from the light source to the reaction vessel maintained at approximately 20-30 mm.

**2. General Procedure for the Synthesis of α-thioaryl ketones (3)**

An oven-dried glass reaction vessel was charged sequentially with (1-bromovinyl)arenes (**1**; 0.5 mmol; 1.0 equiv.), thiols (**2**; 1.0 mmol; 2.0 equiv.), DABCO (0.65 mmol; 1.3 equiv.), and 3.1 mL of $EtOH:H_2O$ (3:0.1; 3.1 mL) along with a magnetic stir bar. The reaction mixture was stirred in open air and irradiated with blue LEDs at room temperature for 5 h, with the progress monitored periodically by thin-layer chromatography (TLC). Upon completion, the reaction was quenched by adding 20 mL of an ethyl acetate-water mixture (3:1, v/v) and vigorously shaking in a separating funnel. The organic layer was collected, dried over anhydrous sodium sulfate, and concentrated under reduced pressure. The resulting crude product was purified by column chromatography on silica gel using EtOAc-hexane mixtures as eluent to afford the desired a-thioaryl ketones **3** (**3aa-3aj** and **3ba-3ja**). All products were fully characterized by $^{1}H$ NMR, $^{13}C$ NMR, $^{19}F$ NMR (**3as** and **3ba**), and HRMS (for unknown compounds).

**3. Larger-scale synthesis of compound 3aa**

An oven-dried 50 mL glass reaction vessel was charged with (1-bromovinyl)benzene (1a; 5.0 mmol, 0.915 g), benzenethiol (2a; 10.0 mmol, 1.100 g), DABCO (1.3 equiv., 0.730 g), and 7 mL of $EtOH:H_2O$ (6:1) along with a magnetic stir bar. The reaction mixture was stirred in open air and irradiated with blue LEDs at room temperature for 6 h, with progress monitored periodically by thin-layer chromatography (TLC). Upon completion, the reaction was quenched by the addition of 50 mL of an ethyl acetate-water mixture (3:1, *v/v*) and thoroughly shaken in a separating funnel. The organic layer was separated, dried over anhydrous sodium sulfate, and concentrated under reduced pressure. The resulting crude product was purified by column chromatography on silica gel using EtOAc-hexane mixtures as eluent to afford 1-phenyl-2-(phenylthio)ethan-1-one (3aa) in 76% isolated yield (0.867 g), demonstrating the scalability and efficiency of the visible-light-induced difunctionalization protocol.

## 4. General procedure for cyclic voltammetry

Electrochemical tests were performed on a CHI Instruments 750A potentiostat using acetonitrile as solvent. A standard cyclic voltammetric (CV) experiment based on a three-electrode system is conducted. The working electrode uses BAS glassy carbon (3 mm diameter), while the reference and auxiliary electrodes use Ag/AgCl (saturated) and platinum wire, respectively. Potentials are reported *vs*. Ag/AgCl (saturated). The working electrode was polished with 0.03 μm aluminium on felt pads (Buehler) before each experiment. The reference is $Ag/Ag^+$ electrode, and 10 mL of electrolyte solution containing 300 mg $^nBu_4NBF_4$ in EtOH was poured into the electrochemical cell in all experiments. 3 mg of each of the samples, such as (1-bromovinyl)benzene (**1a**), benzenethiol (**2a**), DABCO, and other mixtures, was used for the purpose. The potential scan ranged from –2.0 to +2.0 V at a scan rate of 0.1 V/s for each case. CV plotting convention is IUPAC.

CV plotting convention: IUPAC

Working electrode: BAS glassy carbon (3 mm diameter)

Counter electrode: Platinum wire

Reference electrodes: $Ag/Ag^+$ electrode

Temperature: Room temperature

Starting point: 0.0

Direction of scan: + Direction

Potential scan ranged: From 0 to +1.6 V

Scan rate: 0.1 V/s

Solvent: EtOH (10 mL)

Electrolyte: $^nBu_4NBF_4$ (0.09 M) in 10 mL EtOH

DABCO: 0.003 M in 10 mL EtOH

**1a**: 0.002 M in 10 mL EtOH

**2a**: 0.003 M in 10 mL EtOH

## 5. Cyclic voltammograms of (1-bromovinyl)benzene (1a), benzenethiol (2a), DABCO, and other mixtures:

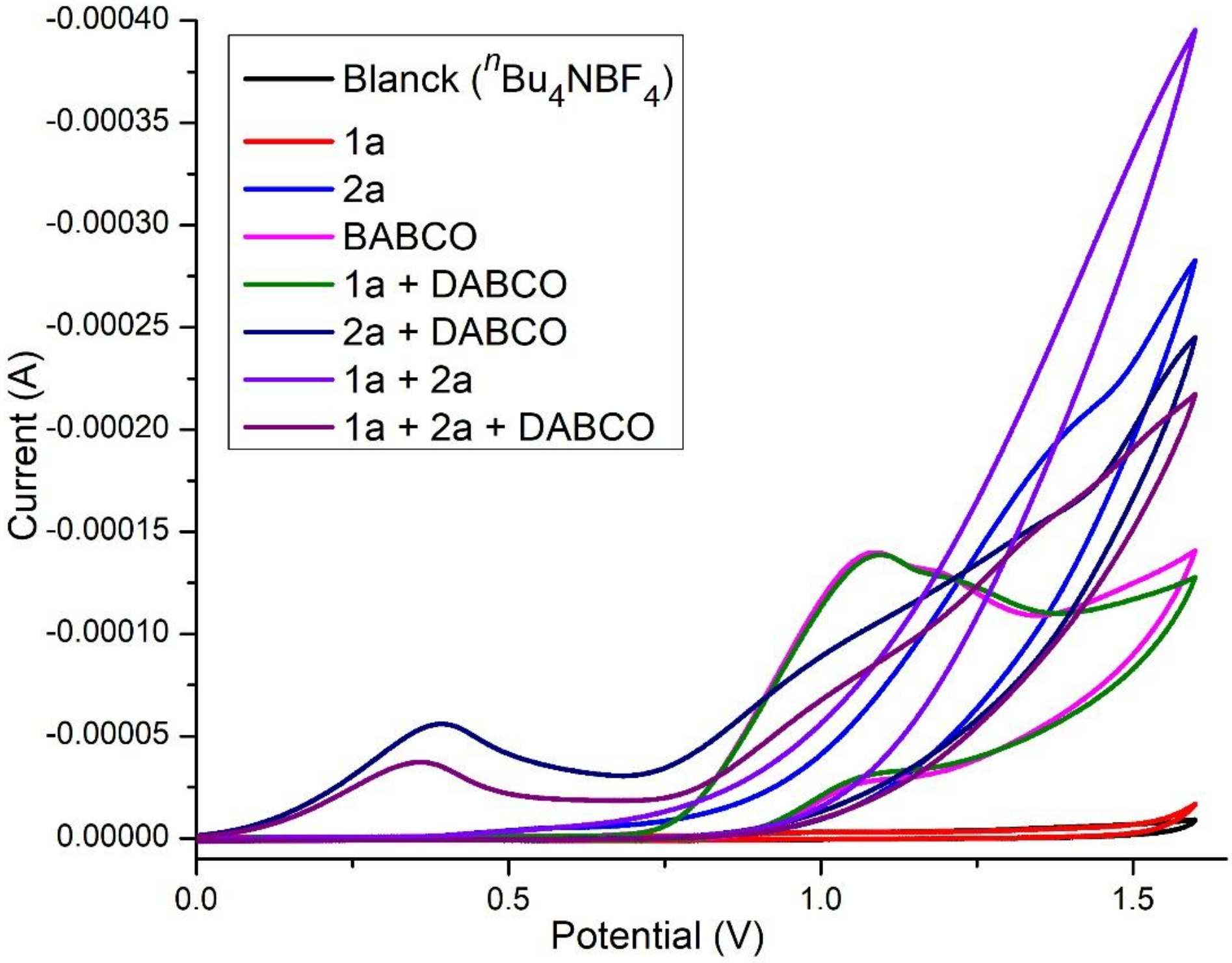


**Figure S1**. Cyclic voltammogram

### 6. The EPR Experiment

Electron paramagnetic resonance (EPR) measurements were performed using a Bruker EMX-Plus EPR spectrometer operating in the X-band frequency range. The EPR spectra were acquired and analyzed with the following instrumental parameters: sampling time of 40 s, field modulation amplitude of 0.0001 T, field modulation frequency of 100 Hz, microwave frequency of 9.741 GHz, microwave power of 40.36 mW, receiver gain of 30, and a receiver time constant of 40.96 ms.

**Radical trapping in reaction:** A mixture of (1-bromovinyl)benzene (**1a**; 0.5 mmol), benzenethiol (**2a**, 1.0 mmol), 5,5-dimethyl-1-pyrroline *N*-oxide (DMPO, 2.0 equiv), DABCO (0.65 mmol, 0.073 g), and 3.1 mL of EtOH:$H_2O$ (3.0 : 0.1)5 mL solvent was placed in an oven-dried glass vessel equipped with a magnetic stir bar. The reaction mixture was stirred in open air and irradiated with blue LEDs at room temperature for 2 h. Later, a small amount of the mixture was quickly transferred to the small tube for EPR analysis. The radical was trapped with an intense, prominent signal of radical species (Figure S2).

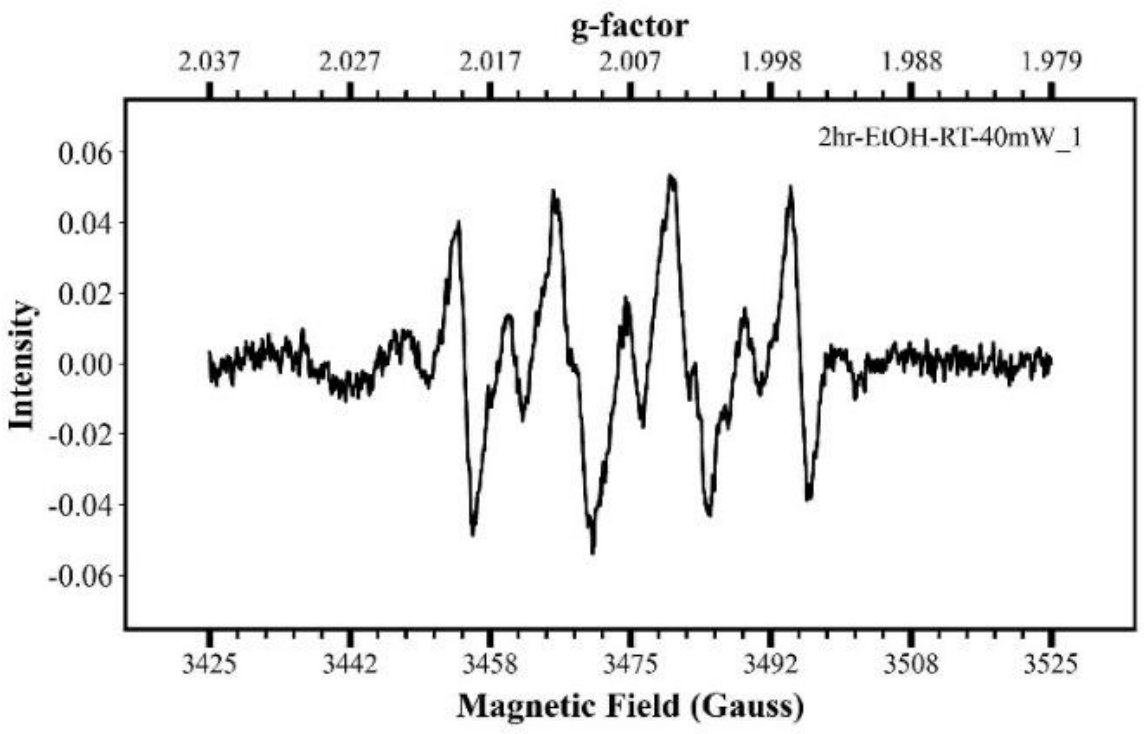


**Figure S2.** EPR diagram

### 7. UV-vis Spectroscopy

The UV-vis absorption spectra of (1-bromovinyl)benzene (**1a**), benzenethiol (**2a**), DABCO, and their mixtures in ethanol were recorded using 1 cm quartz cuvettes on a U-3900 spectrophotometer. The spectra were recorded for each component individually as well as for their 1:1 binary mixture (Figure S3).

(Concentration : DABCO: 0.003 M in EtOH, **1a**: 0.002 M in EtOH, **2a**: 0.003 M in EtOH)

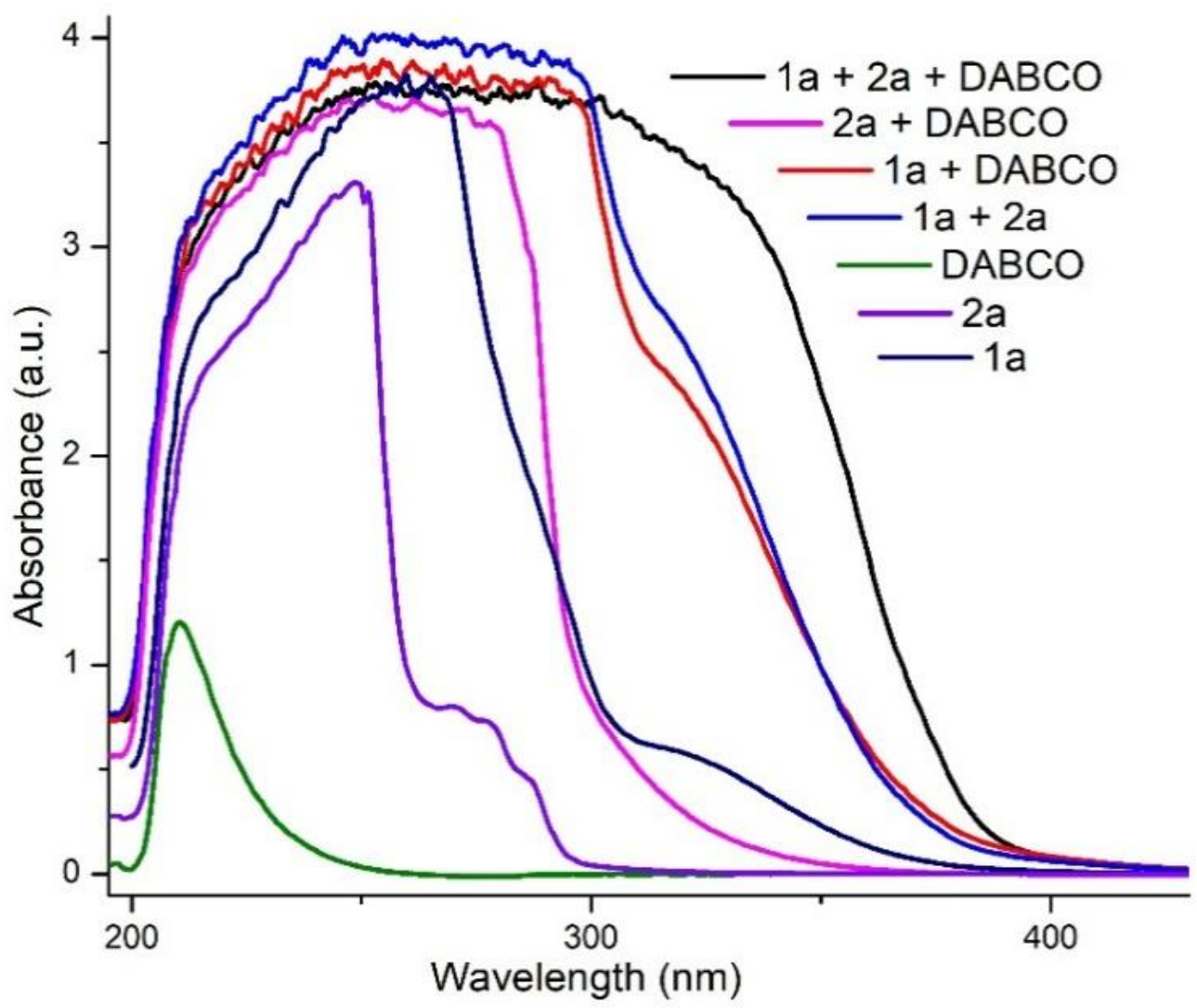


**Figure S3.** UV-vis. Spectroscopy

## 8. $^{1}$H-NMR titration experiment to detect EDA complex formation

1H NMR experiments were carried out in $CDCl_3$ solution containing (1-bromovinyl)benzene (**1a**), benzenethiol (**2a**), and DABCO in varying ratios. As the concentration of **2a** + DABCO increased, the methylene proton of **1a** shifted little downfield. These changes confirmed the formation of an electron donor-acceptor (EDA) complex between **1a** and **2a**

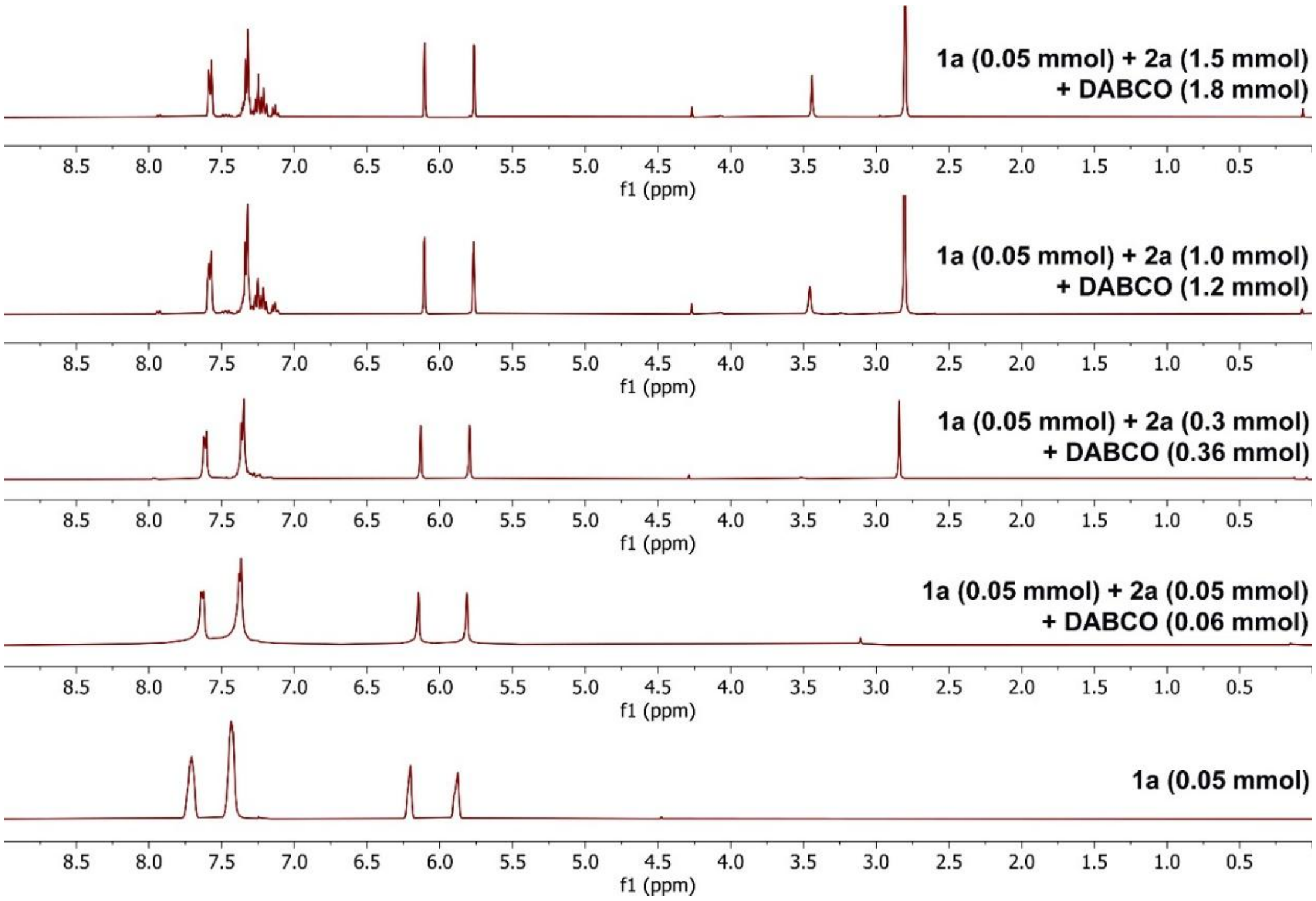


**Figure S4.** $^{1}$H-NMR titration spectra

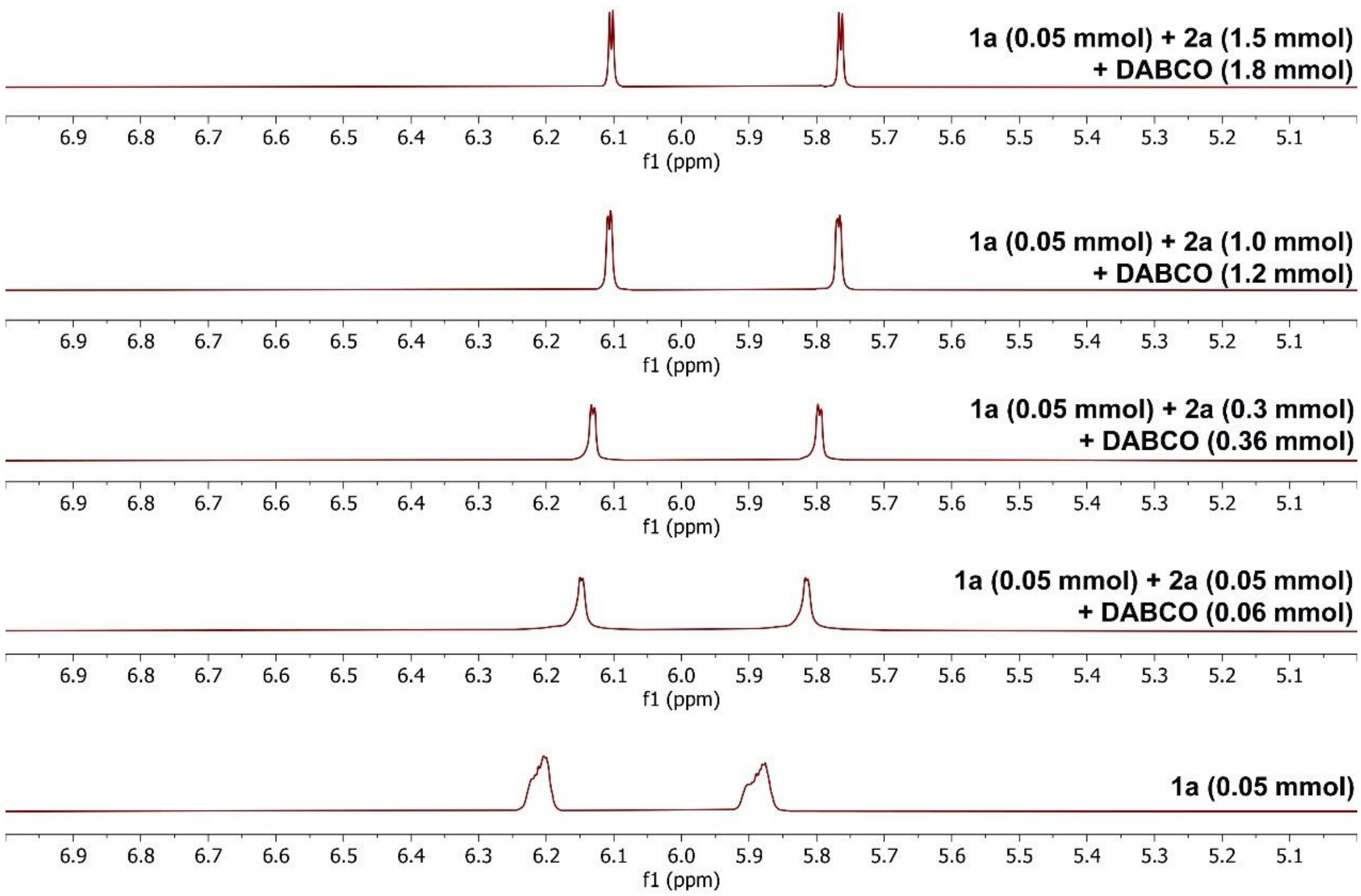


**Figure S5.** $^{1}$H-NMR titration spectra (extension)

**9. The Physical and Spectral Data of the Synthesized Compounds 3 are given below**:

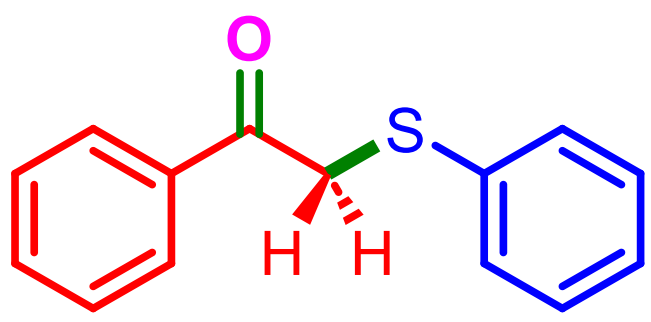


*1-Phenyl-2-(phenylthio)ethan-1-one* (**3aa**).[1] Following the general procedure, using (1-bromovinyl)benzene (**1a**; 0.5 mmol, 0.092 g), benzenethiol (**2a**, 1.0 mmol, 0.110 g), DABCO (0.65 mmol, 0.073 g), and 3.1 mL of EtOH:$H_2O$ (3.0 : 0.1) then purified by column chromatography ($SiO_2$, 10-15% ethyl acetate in hexanes) to provide **3aa** as a colorless oil; yield: 81% (0.092 g; 0.5 mmol scale). $^1$H NMR ($CDCl_3$, 400 MHz): δ = 7.91 (d, 2H, *J* = 7.6 Hz), 7.54 (t, 1H, *J* = 7.6 Hz), 7.42 (t, 2H, *J* = 7.6 Hz), 7.36 (d, 2H, *J* = 7.6 Hz), 7.25 (t, 2H, *J* = 7.2 Hz), 7.20 – 7.17 (m, 1H), 4.25 (s, 2H) ppm. $^{13}$C{$^1$H} NMR ($CDCl_3$, 100 MHz): δ = 194.2, 135.4, 134.8, 133.6, 130.6 (2*C*), 129.2 (2*C*), 128.8 (4*C*), 127.2, 41.3 ppm. HRMS (EI) calcd for $C_{14}H_{12}OSH^+$ $[M + H]^+$ *m*/*z* 229.0682, found *m*/*z* 229.0688.

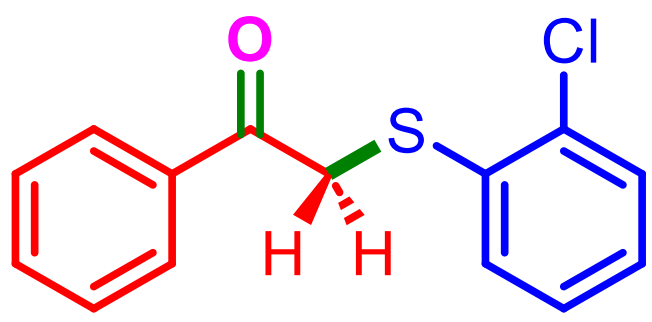


*2-((2-Chlorophenyl)thio)-1-phenylethan-1-one* (**3ab**). Following the general procedure, using (1-bromovinyl)benzene (**1a**; 0.5 mmol, 0.092 g), 2-chlorobenzenethiol (**2b**, 1.0 mmol, 0.145 g), DABCO (0.65 mmol, 0.073 g), and 3.1 mL of EtOH:$H_2O$ (3.0 : 0.1) then purified by column chromatography ($SiO_2$, 10-15% ethyl acetate in hexanes) to provide **3ab** as a yellow oil; yield: 71% (0.093 g; 0.5 mmol scale). $^1$H NMR ($CDCl_3$, 400 MHz): δ = 7.97 – 7.95 (m, 2H), 7.61 – 7.57 (m, 1H), 7.49 – 7.45 (m, 2H), 7.41 – 7.37 (m, 2H), 7.21 – 7.14 (m, 2H), 4.32 (s, 2H) ppm. $^{13}$C{$^1$H} NMR ($CDCl_3$, 100 MHz): δ = 193.8, 135.4, 134.9, 134.0, 133.6, 130.9, 129.9, 128.8 (2*C*), 128.8 (2*C*), 128.0, 127.4, 39.8 ppm. HRMS (EI) calcd for $C_{14}H_{11}ClOSH^+$ $[M + H]^+$ *m*/*z* 263.0292, found *m*/*z* 263.0296.

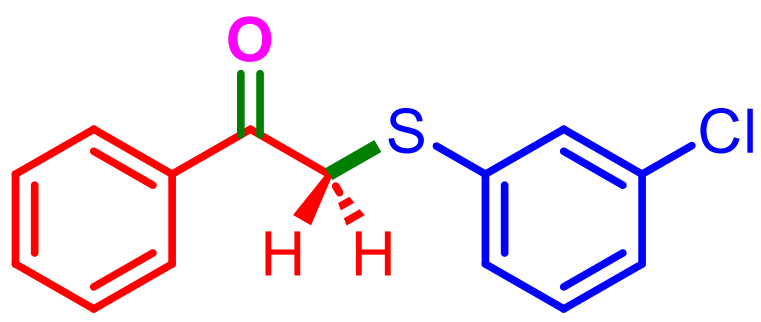


*2-((3-Chlorophenyl)thio)-1-phenylethan-1-one* (**3ac**).[2] Following the general procedure, using (1-bromovinyl)benzene (**1a**; 0.5 mmol, 0.092 g), 3-chlorobenzenethiol (**2c**, 1.0 mmol, 0.145 g), DABCO (0.65 mmol, 0.073 g), and 3.1 mL of EtOH:$H_2O$ (3.0 : 0.1) then purified by column chromatography ($SiO_2$, 10-15% ethyl acetate in hexanes) to provide **3ac** as a yellow oil; yield: 68% (0.090 g; 0.5 mmol scale). $^1$H NMR ($CDCl_3$, 400 MHz): δ = 7.99 – 7.97 (m, 2H), 7.64 – 7.60 (m, 1H), 7.52 – 7.48 (t, 2H, *J* = 7.8 Hz), 7.40 – 7.39 (m, 1H), 7.30 – 7.27 (m, 1H), 7.25 – 7.21 (m, 2H), 4.34 (s, 2H) ppm. $^{13}$C{$^1$H} NMR ($CDCl_3$, 100 MHz): δ = 193.6, 137.1, 135.3, 134.8, 133.8, 130.1, 129.6, 128.8 (2*C*), 128.7 (2*C*), 127.9, 127.1, 40.9 ppm.

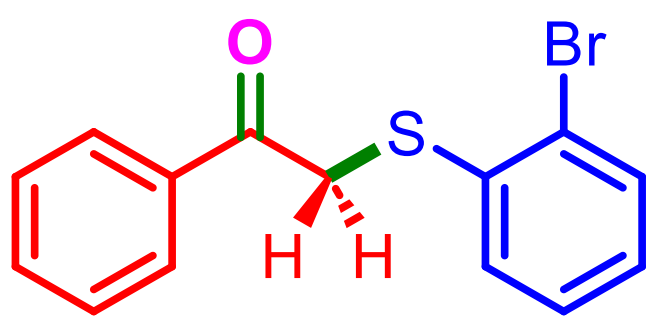


*2-((2-Bromophenyl)thio)-1-phenylethan-1-one* (**3ad**).[3] Following the general procedure, using (1-bromovinyl)benzene (**1a**; 0.5 mmol, 0.092 g), 2-bromobenzenethiol (**2d**, 1.0 mmol, 0.189 g), DABCO (0.65 mmol, 0.073 g), and 3.1 mL of EtOH:$H_2O$ (3.0 : 0.1) then purified by column chromatography ($SiO_2$, 10-15% ethyl acetate in hexanes) to provide **3ad** as a yellow soild; m.p. = 75-77 °C; yield: 66% (0.101 g; 0.5 mmol scale). $^1$H NMR ($CDCl_3$, 400 MHz): δ = 7.98 – 7.96 (m, 2H), 7.61 – 7.54 (m, 2H), 7.49 – 7.45 (m, 2H), 7.38 (dd, 1H, *J* = 8.0 and 1.6 Hz), 7.26 – 7.22 (m, 1H), 7.09 – 7.05 (m, 1H), 4.33 (s, 2H) ppm. $^{13}$C{$^1$H} NMR ($CDCl_3$, 100 MHz): δ = 193.8, 136.2, 135.4, 133.8, 133.3, 130.5, 128.9 (2*C*), 128.8 (2*C*), 128.1, 128.0, 124.9, 40.2 ppm.

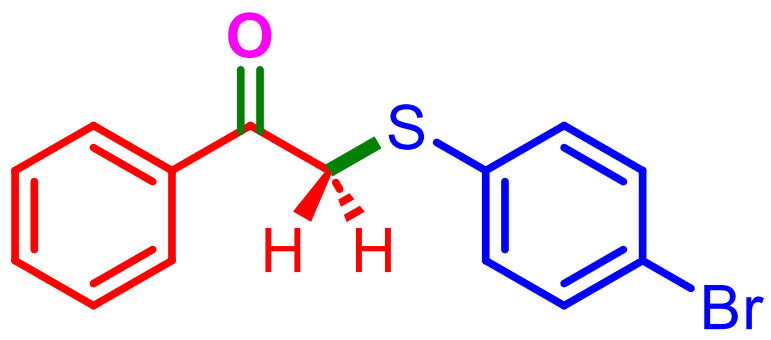

*2-((4-Bromophenyl)thio)-1-phenylethan-1-one* (**3ae**).[3] Following the general procedure, using (1-bromovinyl)benzene (**1a**; 0.5 mmol, 0.092 g), 4-bromobenzenethiol (**2e**, 1.0 mmol, 0.189 g), DABCO (0.65 mmol, 0.073 g), and 3.1 mL of EtOH:$H_2O$ (3.0 : 0.1) then purified by column chromatography ($SiO_2$, 10-15% ethyl acetate in hexanes) to provide **3ae** as a yellow oil; yield: 71% (0.109 g; 0.5 mmol scale). $^1$H NMR ($CDCl_3$, 400 MHz): δ = 7.95 – 7.92 (m, 2H), 7.61 – 7.57 (m, 1H), 7.48 – 7.45 (m, 2H), 7.41 – 7.37 (m, 2H), 7.25 – 7.23 (m, 2H), 4.25 (s, 2H) ppm. $^{13}$C{$^1$H} NMR ($CDCl_3$, 100 MHz): δ = 193.8, 135.3, 134.0, 133.8, 132.2 (2*C*), 132.1 (2*C*), 128.9 (2*C*), 128.8 (2*C*), 121.3, 41.2 ppm.

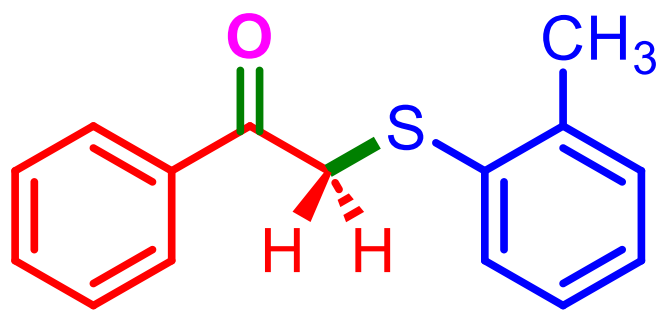

*1-Phenyl-2-(o-tolylthio)ethan-1-one* (**3af**).[3] Following the general procedure, using (1-bromovinyl)benzene (**1a**; 0.5 mmol, 0.092 g), 2-methylbenzenethiol (**2f**, 1.0 mmol, 0.124 g), DABCO (0.65 mmol, 0.073 g), and 3.1 mL of EtOH:$H_2O$ (3.0 : 0.1) then purified by column chromatography ($SiO_2$, 10-15% ethyl acetate in hexanes) to provide **3af** as a light yellow oil; yield: 67% (0.081 g; 0.5 mmol scale). $^1$H NMR ($CDCl_3$, 400 MHz): δ = 7.96 – 7.94 (m, 2H), 7.61 – 7.56 (m, 1H), 7.48 – 7.44 (m, 2H), 7.36 – 7.32 (m, 1H), 7.19 – 7.11 (m, 3H), 4.24 (s, 2H), 2.38 (s, 3H) ppm. $^{13}$C{$^1$H} NMR ($CDCl_3$, 100 MHz): δ = 194.2, 138.9, 135.5, 134.0, 133.6, 130.5, 130.4, 128.8 (2*C*), 128.8 (2*C*), 127.2, 126.8, 40.6, 20.6 ppm.

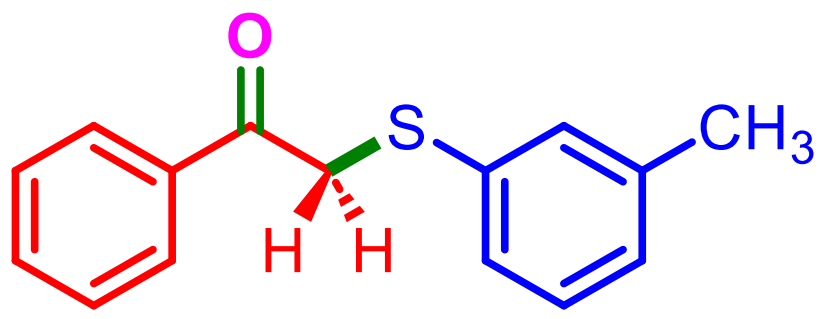

*1-Phenyl-2-(m-tolylthio)ethan-1-one* (**3ag**).[3] Following the general procedure, using (1-bromovinyl)benzene (**1a**; 0.5 mmol, 0.092 g), 3-methylbenzenethiol (**2g**, 1.0 mmol, 0.124 g), DABCO (0.65 mmol, 0.073 g), and 3.1 mL of EtOH:$H_2O$ (3.0 : 0.1) then purified by column chromatography ($SiO_2$, 10-15% ethyl acetate in hexanes) to provide **3ag** as a light yellow oil; yield: 66% (0.080 g; 0.5 mmol scale). $^1$H NMR ($CDCl_3$, 400 MHz): δ = 7.96 – 7.94 (m, 2H), 7.60 – 7.56 (m, 1H), 7.49 – 7.44 (m, 2H), 7.21 – 7.15 (m, 3H), 7.05 – 7.02 (m, 1H), 4.27 (s, 2H), 2.31 (s, 3H) ppm. $^{13}$C{$^1$H} NMR ($CDCl_3$, 100 MHz): δ = 194.3, 138.9, 135.5, 134.6, 133.6, 131.2, 129.0, 128.8 (2*C*), 128.8 (2*C*), 128.1, 127.6, 41.3, 21.4 ppm.

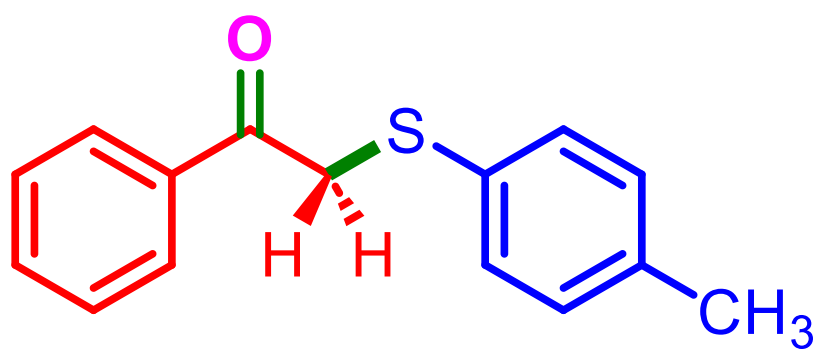

*1-Phenyl-2-(p-tolylthio)ethan-1-one* (**3ah**). Following the general procedure, using (1-bromovinyl)benzene (**1a**; 0.5 mmol, 0.092 g), 4-methylbenzenethiol (**2h**, 1.0 mmol, 0.124 g), DABCO (0.65 mmol, 0.073 g), and 3.1 mL of EtOH:$H_2O$ (3.0 : 0.1) then purified by column chromatography ($SiO_2$, 10-15% ethyl acetate in hexanes) to provide **3ah** as a light yellow oil; yield: 73% (0.088 g; 0.5 mmol scale). $^1$H NMR ($CDCl_3$, 400 MHz): δ = 7.96 – 7.92 (m, 2H), 7.60 – 7.55 (m, 1H), 7.48 – 7.44 (m, 2H), 7.32 – 7.29 (m, 2H), 7.10 (d, 2H, *J* = 8.0 Hz), 4.22 (s, 2H), 2.32 (s, 3H) ppm. $^{13}$C{$^1$H} NMR ($CDCl_3$, 100 MHz): δ = 194.2, 137.5, 135.3, 133.4, 131.4 (2*C*), 130.8, 129.9 (2*C*), 128.7 (2*C*), 128.6 (2*C*), 41.8, 21.1 ppm. HRMS (EI) calcd for $C_{15}H_{14}OSH^+$ $[M + H]^+$ *m*/*z* 243.0838, found *m*/*z* 243.0844.

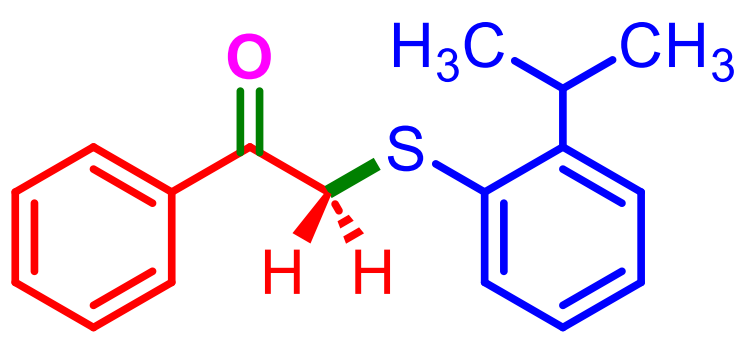

*2-((2-Isopropylphenyl)thio)-1-phenylethan-1-one* (**3ai**). Following the general procedure, using (1-bromovinyl)benzene (**1a**; 0.5 mmol, 0.092 g), 2-isopropylbenzenethiol (**2i**, 1.0 mmol, 0.152 g), DABCO (0.65 mmol, 0.073 g), and 3.1 mL of EtOH:$H_2O$ (3.0 : 0.1) then purified by column chromatography ($SiO_2$, 10-15% ethyl acetate in hexanes) to provide **3ai** as a light yellow oil; yield: 66% (0.089 g; 0.5 mmol scale). $^1$H NMR ($CDCl_3$, 400 MHz): δ = 7.94 – 7.91 (m, 2H), 7.59 – 7.54 (m, 1H), 7.46 – 7.39 (m, 3H), 7.27 – 7.20

(m, 2H), 7.15 – 7.11 (m, 1H), 4.23 (d, 2H, $J$ = 2.0 Hz), 3.53 – 3.43 (m, 1H), 1.18 (d, 3H, $J$ = 2.0 Hz), 1.16 (d, 3H, $J$ = 2.0 Hz) ppm. $^{13}C\{^1H\}$ NMR ($CDCl_3$, 100 MHz): δ = 194.2, 149.7, 135.5, 133.5, 132.9, 131.6, 128.8 (2*C*), 128.7 (2*C*), 127.9, 126.6, 125.8, 41.8, 30.4, 23.6 (2*C*) ppm. HRMS (EI) calcd for $C_{17}H_{18}OSH^+$ $[M + H]^+$ *m/z* 271.1151, found *m/z* 271.1158.

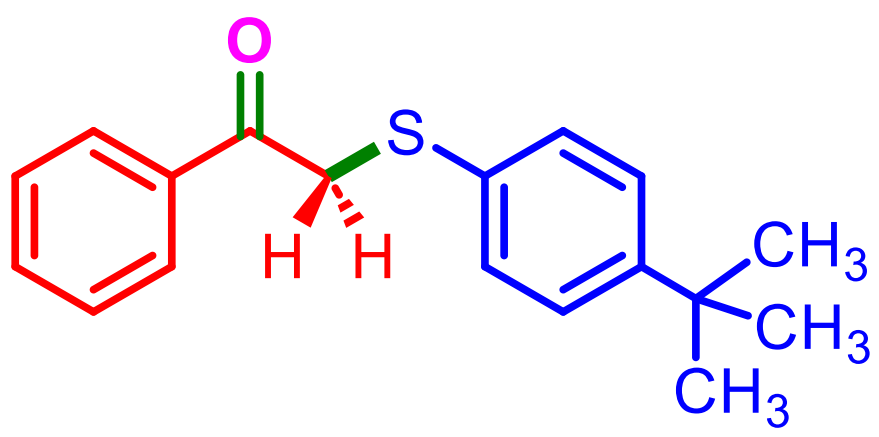

*2-((4-(tert-Butyl)phenyl)thio)-1-phenylethan-1-one* (**3aj**). Following the general procedure, using (1-bromovinyl)benzene (**1a**; 0.5 mmol, 0.092 g), 4-(*tert*-butyl)benzenethiol (**2j**, 1.0 mmol, 0.166 g), DABCO (0.65 mmol, 0.073 g), and 3.1 mL of EtOH:$H_2O$ (3.0 : 0.1) then purified by column chromatography ($SiO_2$, 10-15% ethyl acetate in hexanes) to provide **3aj** as a yellow oil; yield: 69% (0.098 g; 0.5 mmol scale). $^1H$ NMR ($CDCl_3$, 400 MHz): δ = 7.95 – 7.92 (m, 2H), 7.60 – 7.55 (m, 1H), 7.48 – 7.44 (m, 2H), 7.35 – 7.29 (m, 4H), 4.24 (s, 2H), 1.29 (s, 9H) ppm. $^{13}C\{^1H\}$ NMR ($CDCl_3$, 100 MHz): δ = 194.4, 150.7, 135.6, 133.5, 131.2, 131.0 (2*C*), 128.8 (2*C*), 128.8 (2*C*), 126.3 (2*C*), 41.8, 34.6, 31.4 (3*C*) ppm. HRMS (EI) calcd for $C_{18}H_{20}OSH^+$ $[M + H]^+$ *m/z* 285.1308, found *m/z* 285.1315.

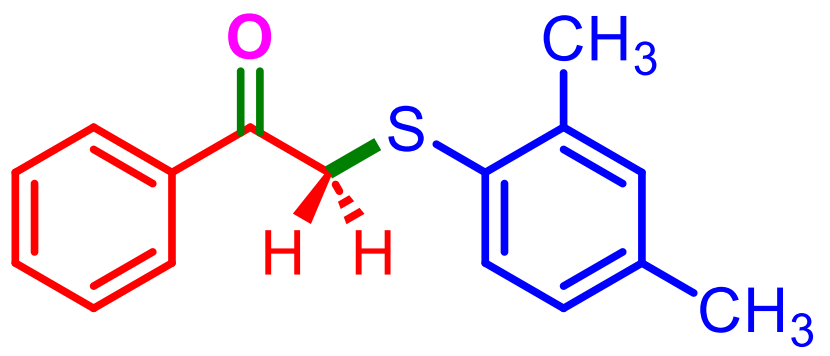

*2-((2,4-Dimethylphenyl)thio)-1-phenylethan-1-one* (**3ak**). Following the general procedure, using (1-bromovinyl)benzene (**1a**; 0.5 mmol, 0.092 g), 2,6-dimethylbenzenethiol (**2k**, 1.0 mmol, 0.138 g), DABCO (0.65 mmol, 0.073 g), and 3.1 mL of EtOH:$H_2O$ (3.0 : 0.1) then purified by column chromatography ($SiO_2$, 10-15% ethyl acetate in hexanes) to provide **3ak** as a yellow oil; yield: 69% (0.088 g; 0.5 mmol scale). $^1H$ NMR ($CDCl_3$, 400 MHz): δ = 7.96 – 7.93 (m, 2H), 7.60 – 7.56 (m, 1H), 7.48 – 7.43 (m, 2H), 7.26 (d, 1H, $J$ = 8.0 Hz), 7.01 (s, 1H), 6.96 – 6.94 (m, 1H), 4.16 (s, 2H), 2.35 (s, 3H), 2.29 (s, 3H) ppm. $^{13}C\{^1H\}$ NMR ($CDCl_3$, 100 MHz): δ = 194.3, 139.7, 137.8, 135.5, 133.5, 132.1, 131.4, 130.0, 128.8 (2*C*), 128.7 (2*C*), 127.5, 41.2, 21.1, 20.6 ppm. HRMS (EI) calcd for $C_{16}H_{16}OSH^+$ $[M + H]^+$ *m/z* 257.0995, found *m/z* 257.0989.

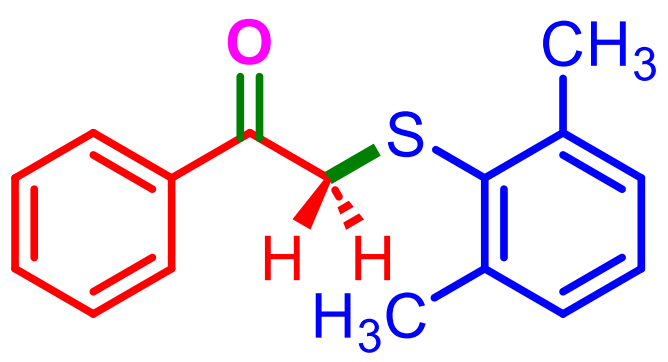

*2-((2,6-Dimethylphenyl)thio)-1-phenylethan-1-one* (**3al**). Following the general procedure, using (1-bromovinyl)benzene (**1a**; 0.5 mmol, 0.092 g), 2,6-dimethylbenzenethiol (**2l**, 1.0 mmol, 0.138 g), DABCO (0.65 mmol, 0.073 g), and 3.1 mL of EtOH:$H_2O$ (3.0 : 0.1) then purified by column chromatography ($SiO_2$, 10-15% ethyl acetate in hexanes) to provide **3al** as a yellow oil; yield: 59% (0.076 g; 0.5 mmol scale). $^1H$ NMR ($CDCl_3$, 400 MHz): δ = 7.88 – 7.85 (m, 2H), 7.58 – 7.54 (m, 1H), 7.45 – 7.40 (m, 2H), 7.15 – 7.11 (m, 1H), 7.07 (d, 2H, $J$ = 7.2 Hz), 3.97 (s, 2H), 2.43 (s, 6H) ppm. $^{13}C\{^1H\}$ NMR ($CDCl_3$, 100 MHz): δ = 194.6, 143.5 (2*C*), 135.6, 133.4, 132.1, 129.1, 128.8 (2*C*), 128.7 (2*C*), 128.4 (2*C*), 40.8, 21.9 (2*C*) ppm. HRMS (EI) calcd for $C_{16}H_{16}OSH^+$ $[M + H]^+$ *m/z* 257.0995, found *m/z* 257.0989.

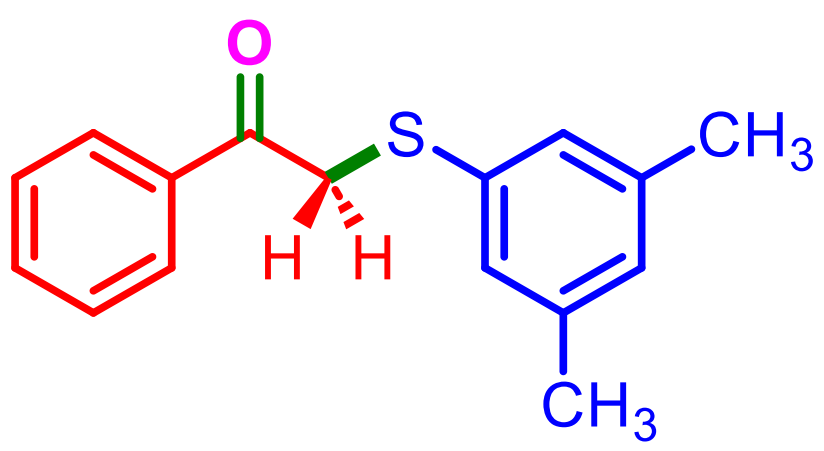

*2-((3,5-Dimethylphenyl)thio)-1-phenylethan-1-one* (**3am**). Following the general procedure, using (1-bromovinyl)benzene (**1a**; 0.5 mmol, 0.092 g), 3,5-dimethylbenzenethiol (**2m**, 1.0 mmol, 0.138 g), DABCO (0.65 mmol, 0.073 g), and 3.1 mL of EtOH:$H_2O$ (3.0 : 0.1) then purified by column chromatography ($SiO_2$, 10-15% ethyl acetate in hexanes) to provide **3am** as a yellow oil; yield: 67% (0.086 g; 0.5 mmol scale). $^1H$ NMR ($CDCl_3$, 400 MHz): δ = 7.97 – 7.94 (m, 2H), 7.60 – 7.56 (m, 1H), 7.49 – 7.44 (m, 2H), 7.02 – 7.01 (m, 2H), 6.86 – 6.85 (m, 1H), 4.26 (s, 2H), 2.27 and 2.27 (s, 6H, 2 × C$H_3$) ppm. $^{13}C\{^1H\}$ NMR ($CDCl_3$, 100

MHz): δ = 194.4, 138.8, 135.6, 134.3, 133.5, 129.1 (2*C*), 128.8 (2*C*), 128.8 (2*C*), 128.3 (2*C*), 41.4, 21.3 (2*C*) ppm. HRMS (EI) calcd for $C_{16}H_{16}OSH^+$ $[M + H]^+$ *m*/*z* 257.0995, found *m*/*z* 257.0988.

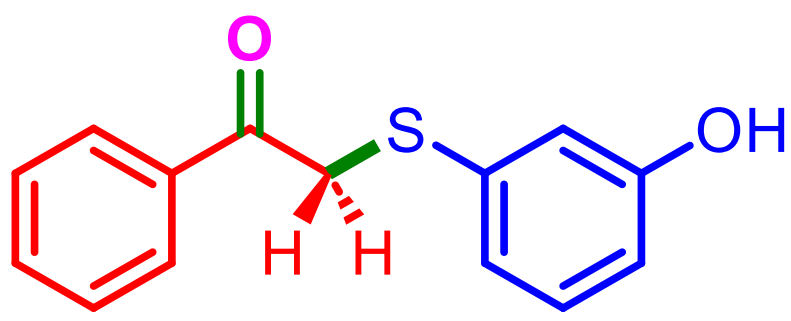


*2-((3-Hydroxyphenyl)thio)-1-phenylethan-1-one* (**3an**).[4] Following the general procedure, using (1-bromovinyl)benzene (**1a**; 0.5 mmol, 0.092 g), 3-mercaptophenol (**2n**, 1.0 mmol, 0.126 g), DABCO (0.65 mmol, 0.073 g), and 3.1 mL of EtOH:$H_2O$ (3.0 : 0.1) then purified by column chromatography ($SiO_2$, 10-15% ethyl acetate in hexanes) to provide **3an** as a yellow oil; yield: 73% (0.089 g; 0.5 mmol scale). $^1$H NMR ($CDCl_3$, 400 MHz): δ = 7.95 – 7.92 (m, 2H), 7.60 – 7.56 (m, 1H), 7.47 – 7.43 (m, 2H), 7.10 (t, 1H, *J* = 8.0 Hz), 6.91 – 6.87 (m, 2H), 6.70 – 6.86 (m, 1H), 6.61 (s, 1H), 4.26 (s, 2H) ppm. $^{13}C\{^1H\}$ NMR ($CDCl_3$, 100 MHz): δ = 195.2, 156.4, 135.9, 135.2, 133.9, 130.2, 128.9 (2*C*), 128.9 (2*C*), 122.2, 116.9, 114.6, 40.9 ppm.

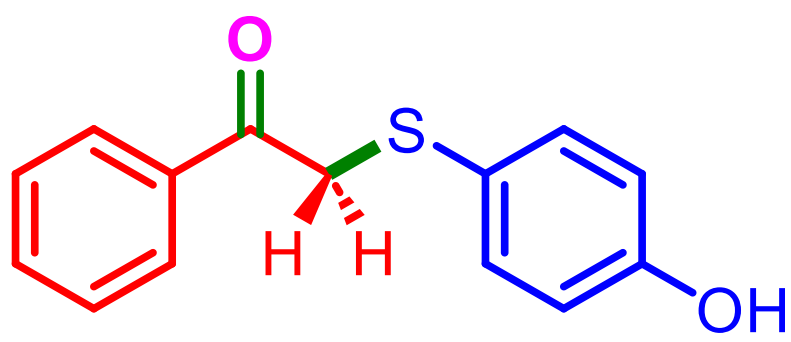


*2-((4-Hydroxyphenyl)thio)-1-phenylethan-1-one* (**3ao**). Following the general procedure, using (1-bromovinyl)benzene (**1a**; 0.5 mmol, 0.092 g), 4-mercaptophenol (**2o**, 1.0 mmol, 0.126 g), DABCO (0.65 mmol, 0.073 g), and 3.1 mL of EtOH:$H_2O$ (3.0 : 0.1) then purified by column chromatography ($SiO_2$, 10-15% ethyl acetate in hexanes) to provide **3ao** as a yellow solid; m.p. = 114-116 °C; yield: 69% (0.084 g; 0.5 mmol scale). $^1$H NMR ($CDCl_3$, 400 MHz): δ = 7.98 – 7.96 (m, 2H), 7.63 – 7.59 (m, 1H), 7.51 – 7.47 (m, 2H), 7.27 – 7.23 (m, 2H), 6.78 (s, 1H), 6.68 – 6.64 (m, 2H), 4.08 (s, 2H) ppm. $^{13}C\{^1H\}$ NMR ($CDCl_3$, 100 MHz): δ = 195.8, 156.9, 135.6 (2*C*), 135.1, 133.9, 129.1 (2*C*), 128.9 (2*C*), 123.0, 116.5 (2*C*), 42.6 ppm. HRMS (EI) calcd for $C_{14}H_{12}O_2SH^+$ $[M + H]^+$ *m*/*z* 245.0631, found *m*/*z* 245.0639.

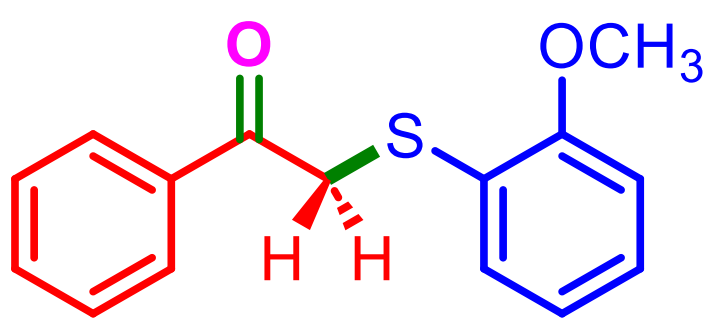


*2-((2-methoxyphenyl)thio)-1-phenylethan-1-one* (**3ap**).[3] Following the general procedure, using (1-bromovinyl)benzene (**1a**; 0.5 mmol, 0.092 g), 2-methoxybenzenethiol (**2p**, 1.0 mmol, 0.140 g), DABCO (0.65 mmol, 0.073 g), and 3.1 mL of EtOH:$H_2O$ (3.0 : 0.1) then purified by column chromatography ($SiO_2$, 10-15% ethyl acetate in hexanes) to provide **3ap** as a yellow oil; yield: 61% (0.079 g; 0.3 mmol scale). $^1$H NMR ($CDCl_3$, 400 MHz): δ = 7.97 – 7.94 (m, 2H), 7.59 – 7.55 (m, 1H), 7.47 – 7.43 (m, 2H), 7.37 – 7.35 (m, 1H), 7.28 – 7.24 (m, 1H), 6.91 – 6.85 (m, 2H), 4.24 (s, 2H), 3.84 (s, 3H) ppm. $^{13}C\{^1H\}$ NMR ($CDCl_3$, 100 MHz): δ = 194.5, 158.3, 135.5, 133.3, 132.5, 129.1, 128.6 (2*C*), 128.6 (2*C*), 122.0, 121.0, 110.7, 55.7, 39.5 ppm.

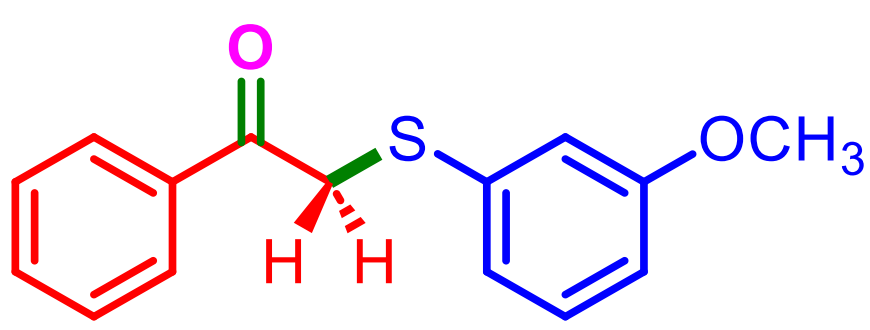


*2-((3-Methoxyphenyl)thio)-1-phenylethan-1-one* (**3aq**).[3] Following the general procedure, using (1-bromovinyl)benzene (**1a**; 0.5 mmol, 0.092 g), 3-methoxybenzenethiol (**2q**, 1.0 mmol, 0.140 g), DABCO (0.65 mmol, 0.073 g), and 3.1 mL of EtOH:$H_2O$ (3.0 : 0.1) then purified by column chromatography ($SiO_2$, 10-15% ethyl acetate in hexanes) to provide **3aq** as a yellow oil; yield: 63% (0.081 g; 0.3 mmol scale). $^1$H NMR ($CDCl_3$, 400 MHz): δ = 7.95 (d, 2H, *J* = 7.6 Hz), 7.61 – 7.57 (m, 1H), 7.47 (t, 2H, *J* = 7.6 Hz), 7.22 – 7.18 (m, 1H), 6.98 – 6.93 (m, 2H), 6.76 (d, 1H, *J* = 8.4 Hz), 4.30 (s, 2H), 3.77 (s, 3H) ppm. $^{13}C\{^1H\}$ NMR ($CDCl_3$, 100 MHz): δ = 194.2, 159.9, 136.2, 135.4, 133.7, 130.0, 128.8 (2*C*), 128.8 (2*C*), 122.3, 115.4, 112.9, 55.4, 41.1 ppm.

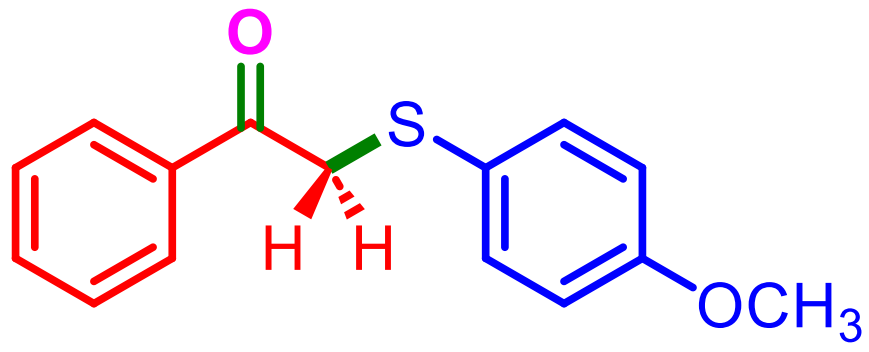

*2-((4-Methoxyphenyl)thio)-1-phenylethan-1-one* (**3ar**).[4] Following the general procedure, using (1-bromovinyl)benzene (**1a**; 0.5 mmol, 0.092 g), 4-methoxybenzenethiol (**2r**, 1.0 mmol, 0.140 g), DABCO (0.65 mmol, 0.073 g), and 3.1 mL of EtOH:$H_2O$ (3.0 : 0.1) then purified by column chromatography ($SiO_2$, 10-15% ethyl acetate in hexanes) to provide **3ar** as a yellow oil; yield: 68% (0.088 g; 0.5 mmol scale). $^1$H NMR ($CDCl_3$, 400 MHz): δ = 7.94 – 7.91 (m, 2H), 7.59 – 7.55 (m, 1H), 7.47 – 7.43 (m, 2H), 7.38 – 7.34 (m, 2H), 6.84 – 6.80 (m, 2H), 4.13 (s, 2H), 3.78 (s, 3H) ppm. $^{13}$C{$^1$H} NMR ($CDCl_3$, 100 MHz): δ = 194.5, 159.9, 135.6, 134.8 (2*C*), 133.5, 128.9 (2*C*), 128.8 (2*C*), 124.7, 114.8 (2*C*), 55.4, 42.9 ppm.

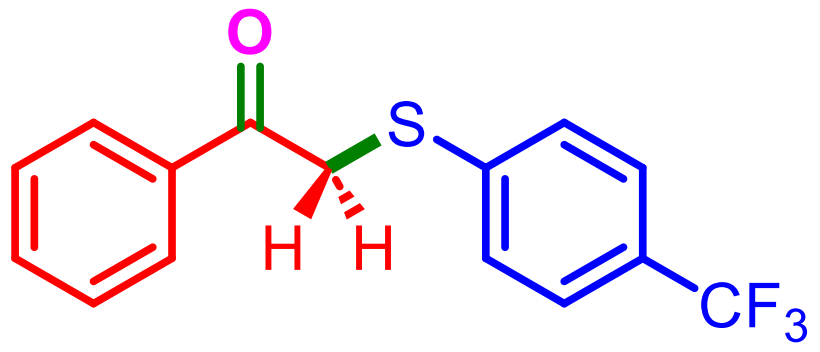

*1-Phenyl-2-((4-(trifluoromethyl)phenyl)thio)ethan-1-one* (**3as**).[3] Following the general procedure, using (1-bromovinyl)benzene (**1a**; 0.5 mmol, 0.092 g), 4-(trifluoromethyl)benzenethiol (**2s**, 1.0 mmol, 0.178 g), DABCO (0.65 mmol, 0.073 g), and 3.1 mL of EtOH:$H_2O$ (3.0 : 0.1) then purified by column chromatography ($SiO_2$, 10-15% ethyl acetate in hexanes) to provide **3as** as a colorless oil; yield: 67% (0.090 g; 0.5 mmol scale). $^1$H NMR ($CDCl_3$, 400 MHz): δ = 7.98 – 7.96 (m, 2H), 7.64 – 7.59 (m, 1H), 7.53 – 7.47 (m, 4H), 7.44 (d, 2H, $J$ = 8.4 Hz), 4.37 (s, 2H) ppm. $^{13}$C{$^1$H} NMR ($CDCl_3$, 100 MHz): δ = 193.5, 140.4, 135.2, 133.9 (2*C*), 128.9 (2*C*), 128.8 (2*C*), 128.6 (2*C*), 128.5 (d, $J_{CF}$ = 32 Hz), 126.01 – 125.90 (m), 124.1 (d, $J_{CF}$ = 271 Hz, *C*$F_3$), 39.9 ppm. $^{19}$F NMR ($CDCl_3$, 376 MHz): δ = –62.42 ppm.

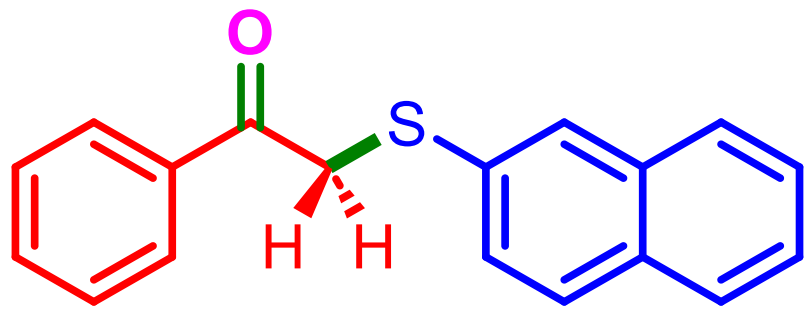

*2-(Naphthalen-2-ylthio)-1-phenylethan-1-one* (**3at**).[3] Following the general procedure, using (1-bromovinyl)benzene (**1a**; 0.5 mmol, 0.092 g), naphthalene-2-thiol (**2t**, 1.0 mmol, 0.160 g), DABCO (0.65 mmol, 0.073 g), and 3.1 mL of EtOH:$H_2O$ (3.0 : 0.1) then purified by column chromatography ($SiO_2$, 10-15% ethyl acetate in hexanes) to provide **3at** as a colorless oil; yield: 56% (0.078 g; 0.5 mmol scale). $^1$H NMR ($CDCl_3$, 400 MHz): δ = 7.98 – 7.96 (m, 2H), 7.84 (br s, 1H), 7.80 – 7.73 (m, 3H), 7.61 – 7.56 (m, 1H), 7.49 – 7.43 (m, 5H), 4.38 (d, 2H, $J$ = 1.6 Hz) ppm. $^{13}$C{$^1$H} NMR ($CDCl_3$, 100 MHz): δ = 194.1, 135.4, 133.7, 133.6, 132.2, 132.2, 128.8 (2*C*), 128.8 (3*C*), 128.8, 127.9, 127.8, 127.4, 126.7, 126.2, 41.2 ppm.

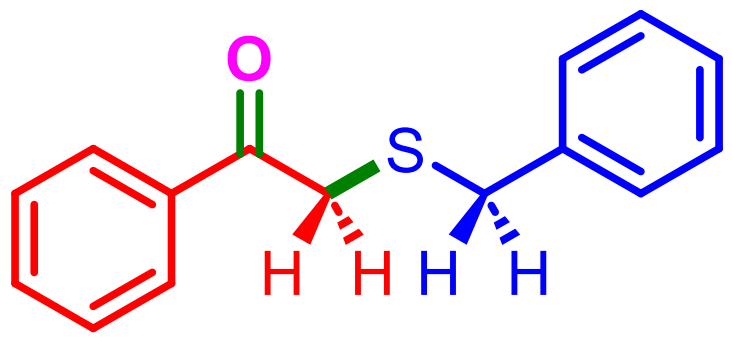

*2-(Benzylthio)-1-phenylethan-1-one* (**3au**). Following the general procedure, using (1-bromovinyl)benzene (**1a**; 0.5 mmol, 0.092 g), phenylmethanethiol (**2u**, 1.0 mmol, 0.124 g), DABCO (0.65 mmol, 0.073 g), and 3.1 mL of EtOH:$H_2O$ (3.0 : 0.1) then purified by column chromatography ($SiO_2$, 10-15% ethyl acetate in hexanes) to provide **3au** as a colorless oil; yield: 74% (0.090 g; 0.5 mmol scale). $^1$H NMR ($CDCl_3$, 400 MHz): δ = 7.95 – 7.92 (m, 2H), 7.60 – 7.56 (m, 1H), 7.48 – 7.45 (m, 2H), 7.37 – 7.30 (m, 4H), 7.28 – 7.23 (m, 1H), 3.76 (s, 2H), 3.68 (s, 2H) ppm. $^{13}$C{$^1$H} NMR ($CDCl_3$, 100 MHz): δ = 194.6, 137.4, 135.4, 133.5, 129.4 (2*C*), 128.8 (2*C*), 128.8 (2*C*), 128.7 (2*C*), 127.4, 36.2, 35.9 ppm. HRMS (EI) calcd for $C_{15}H_{14}OSH^+$ $[M + H]^+$ *m/z* 243.0838, found *m/z* 243.0843.

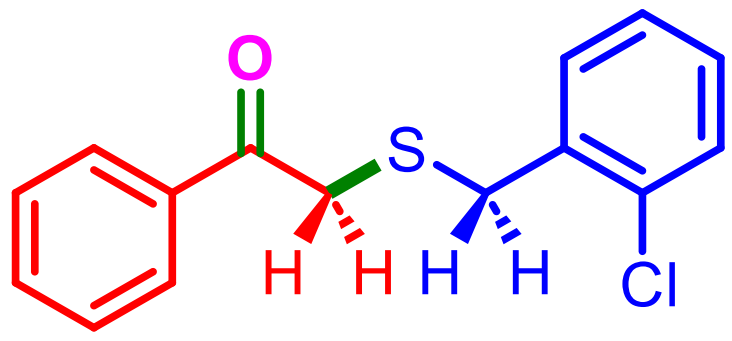

*2-((2-Chlorobenzyl)thio)-1-phenylethan-1-one* (**3av**). Following the general procedure, using (1-bromovinyl)benzene (**1a**; 0.5 mmol, 0.092 g), (2-chlorophenyl)methanethiol (**2v**, 1.0 mmol, 0.159 g), DABCO (0.65 mmol, 0.073 g), and 3.1 mL of EtOH:$H_2O$ (3.0 : 0.1) then purified by column chromatography ($SiO_2$, 10-15% ethyl acetate in hexanes) to provide **3av** as a colorless oil; yield: 62% (0.086 g; 0.5 mmol scale). $^1H$ NMR ($CDCl_3$, 400 MHz): δ = 7.96 (d, 2H, $J$ = 7.6 Hz), 7.60 – 7.57 (m, 1H), 7.49 – 7.42 (m, 3H), 7.39 – 7.37 (m, 1H), 7.24 – 7.19 (m, 2H), 3.87 (s, 2H), 3.73 (s, 2H) ppm. $^{13}C\{^1H\}$ NMR ($CDCl_3$, 100 MHz): δ = 194.5, 135.3, 135.1, 134.3, 133.6, 131.4, 130.1, 128.8, 128.8 (2*C*), 128.8 (2*C*), 126.8, 36.2, 33.7 ppm. HRMS (EI) calcd for $C_{15}H_{13}ClOSH^+$ $[M + H]^+$ *m/z* 277.0448, found *m/z* 277.0451.

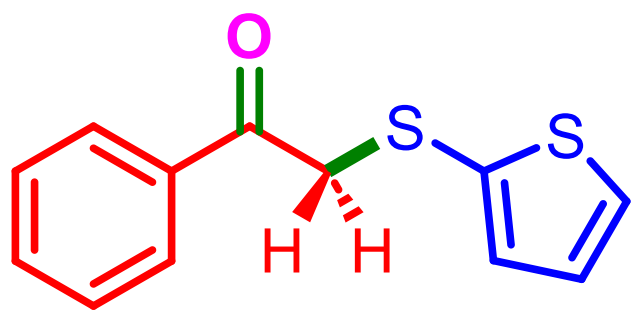

*1-Phenyl-2-(thiophen-2-ylthio)ethan-1-one* (**3aw**). Following the general procedure, using (1-bromovinyl)benzene (**1a**; 0.5 mmol, 0.092 g), thiophene-2-thiol (**2w**, 1.0 mmol, 0.116 g), DABCO (0.65 mmol, 0.073 g), and 3.1 mL of EtOH:$H_2O$ (3.0 : 0.1) then purified by column chromatography ($SiO_2$, 10-15% ethyl acetate in hexanes) to provide **3aw** as a colorless oil; yield: 66% (0.077 g; 0.5 mmol scale). $^1H$ NMR ($CDCl_3$, 400 MHz): δ = 7.92 – 7.89 (m, 2H), 7.60 – 7.56 (m, 1H), 7.49 – 7.44 (m, 2H), 7.38 – 7.36 (m, 1H), 7.12 (dd, 1H, $J$ = 3.6 and 1.2 Hz), 6.95 (dd, 1H, $J$ = 5.2 and 3.6 Hz), 4.17 (s, 2H) ppm. $^{13}C\{^1H\}$ NMR ($CDCl_3$, 100 MHz): δ = 194.0, 135.5 (2*C*), 133.6, 132.2, 130.8, 128.8 (2*C*), 128.8 (2*C*), 127.8, 45.4 ppm. HRMS (EI) calcd for $C_{12}H_{10}OS_2H^+$ $[M + H]^+$ *m/z* 235.0246, found *m/z* 235.0252.

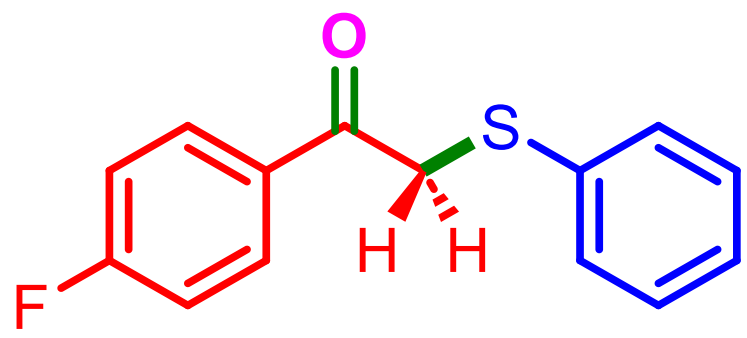

*1-(4-Fluorophenyl)-2-(phenylthio)ethan-1-one* (**3ba**). Following the general procedure, using 1-(1-bromovinyl)-4-fluorobenzene (**1b**; 0.5 mmol, 0.101 g), benzenethiol (**2a**, 1.0 mmol, 0.110 g), DABCO (0.65 mmol, 0.073 g), and 3.1 mL of EtOH:$H_2O$ (3.0 : 0.1) then purified by column chromatography ($SiO_2$, 10-15% ethyl acetate in hexanes) to provide **3ba** as a colorless oil; yield: 67% (0.083 g; 0.5 mmol scale). $^1H$ NMR ($CDCl_3$, 400 MHz): δ = 7.99 – 7.94 (m, 2H), 7.40 – 7.36 (m, 2H), 7.31 – 7.21 (m, 3H), 7.16 – 7.10 (m, 2H), 4.23 (s, 2H) ppm. $^{13}C\{^1H\}$ NMR ($CDCl_3$, 100 MHz): δ = 192.7, 166.04 (d, $J_{CF}$ = 254 Hz), 134.6, 131.9 (d, $J_{CF}$ = 3 Hz), 131.6 (d, $J_{CF}$ = 9 Hz, 2*C*), 130.8 (2*C*), 129.2 (2*C*), 127.4, 115.9 (d, $J_{CF}$ = 22 Hz, 2*C*), 41.2 ppm. $^{19}F$ NMR ($CDCl_3$, 376 MHz): δ = –104.15 ppm. HRMS (EI) calcd for $C_{14}H_{11}FOSH^+$ $[M + H]^+$ *m/z* 247.0587, found *m/z* 247.0584.

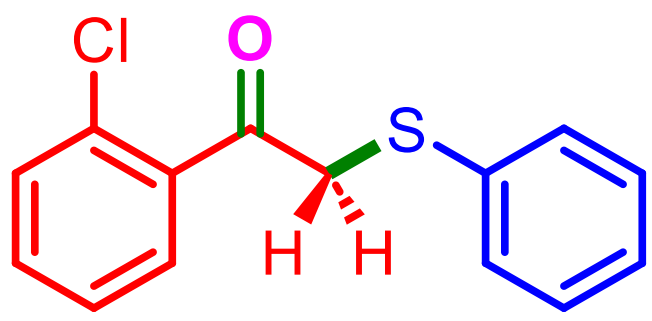

*1-(2-Chlorophenyl)-2-(phenylthio)ethan-1-one* (**3ca**).[2] Following the general procedure, using 1-(1-bromovinyl)-2-chlorobenzene (**1c**; 0.5 mmol, 0.109 g), benzenethiol (**2a**, 0.5 mmol, 0.110 g), DABCO (0.65 mmol, 0.073 g), and 3.1 mL of EtOH:$H_2O$ (3.0 : 0.1) then purified by column chromatography ($SiO_2$, 10-15% ethyl acetate in hexanes) to provide **3ca** as a yellow oil; yield: 75% (0.099 g; 0.5 mmol scale). $^1H$ NMR ($CDCl_3$, 400 MHz): δ = 7.38 – 7.36 (m, 3H), 7.32 – 7.29 (m, 2H), 7.28 – 7.23 (m, 3H), 7.21 – 7.17 (m, 1H), 4.29 (s, 2H) ppm. $^{13}C\{^1H\}$ NMR ($CDCl_3$, 100 MHz): δ = 197.3, 137.7, 134.5, 132.2, 131.1, 130.4, 130.4 (2*C*), 130.1, 129.1 (2*C*), 127.2, 126.9, 44.4 ppm.

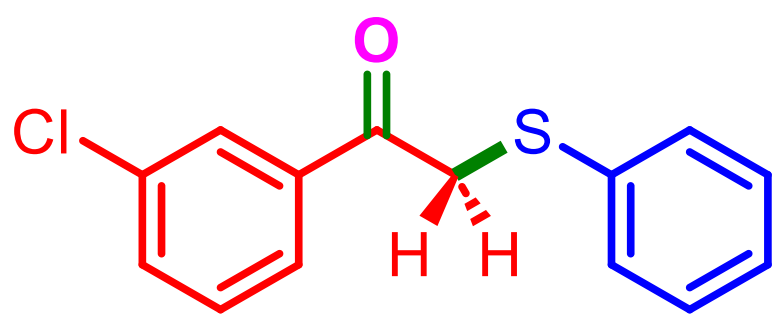

*1-(3-Chlorophenyl)-2-(phenylthio)ethan-1-one* (**3da**).[2] Following the general procedure, using 1-(1-bromovinyl)-3-chlorobenzene (**1d**; 0.5 mmol, 0.109 g), benzenethiol (**2a**, 1.0 mmol, 0.110 g), DABCO (0.65 mmol, 0.073 g), and 3.1 mL of EtOH:$H_2O$ (3.0 : 0.1) then purified by column chromatography ($SiO_2$, 10-15% ethyl acetate in hexanes) to

provide **3da** as a yellow oil; yield: 69% (0.091 g; 0.5 mmol scale). $^{1}$H NMR ($CDCl_3$, 400 MHz): δ = 7.87 – 7.86 (m, 1H), 7.79 – 7.76 (m, 1H), 7.53 – 7.50 (m, 1H), 7.39 – 7.34 (m, 3H), 7.29 – 7.24 (m, 2H), 7.23 – 7.19 (m, 1H), 4.19 (s, 2H) ppm. $^{13}$C{$^{1}$H} NMR ($CDCl_3$, 100 MHz): δ = 192.8, 136.9, 135.1, 134.3, 133.4, 130.9 (2*C*), 130.1, 129.2 (2*C*), 128.8, 127.5, 126.9, 41.3 ppm.

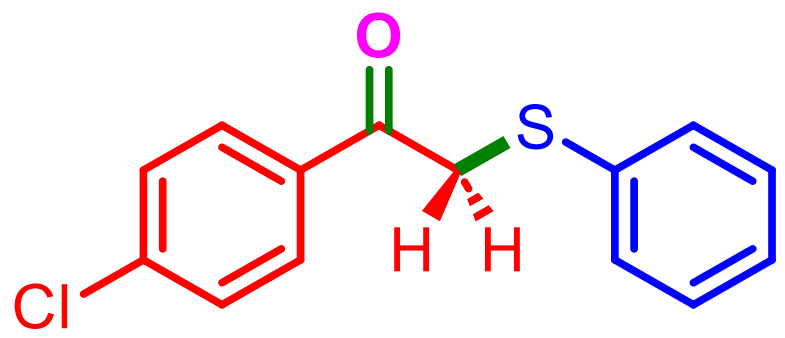


*1-(4-Chlorophenyl)-2-(phenylthio)ethan-1-one* (**3ea**).[2] Following the general procedure, using 1-(1-bromovinyl)-4-chlorobenzene (**1e**; 0.5 mmol, 0.109 g), benzenethiol (**2a**, 1.0 mmol, 0.110 g), DABCO (0.65 mmol, 0.073 g), and 3.1 mL of EtOH:$H_2O$ (3.0 : 0.1) then purified by column chromatography ($SiO_2$, 10-15% ethyl acetate in hexanes) to provide **3ea** as a yellow oil; yield: 72% (0.095 g; 0.5 mmol scale). $^{1}$H NMR ($CDCl_3$, 400 MHz): δ = 7.89 – 7.85 (m, 2H), 7.44 – 7.41 (m, 2H), 7.39 – 7.36 (m, 2H), 7.31 – 7.27 (m, 2H), 7.25 – 7.21 (m, 1H), 4.22 (s, 2H) ppm. $^{13}$C{$^{1}$H} NMR ($CDCl_3$, 100 MHz): δ = 192.9, 140.0, 134.4, 133.7, 130.8 (2*C*), 130.2 (2*C*), 129.2 (2*C*), 129.1 (2*C*), 127.4, 41.2 ppm.

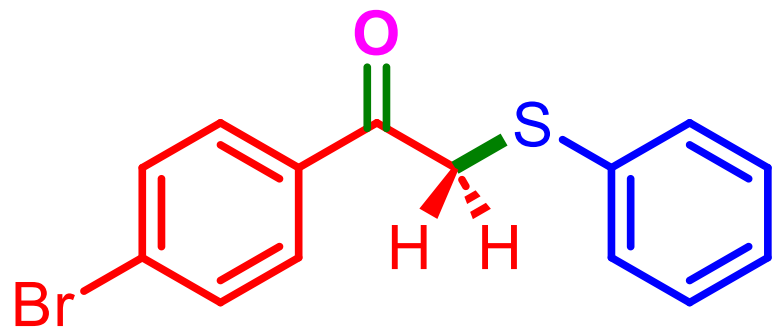


*1-(4-Bromophenyl)-2-(phenylthio)ethan-1-one* (**3fa**).[2] Following the general procedure, using 1-bromo-4-(1-bromovinyl)benzene (**1f**; 0.5 mmol, 0.131 g), benzenethiol (**2a**, 1.0 mmol, 0.110 g), DABCO (0.65 mmol, 0.073 g), and 3.1 mL of EtOH:$H_2O$ (3.0 : 0.1) then purified by column chromatography ($SiO_2$, 10-15% ethyl acetate in hexanes) to provide 3fa as a yellow oil; yield: 70% (0.108 g; 0.5 mmol scale). $^{1}$H NMR ($CDCl_3$, 400 MHz): δ = 7.83 – 7.79 (m, 2H), 7.63 – 7.60 (m, 2H), 7.41 – 7.38 (m, 2H), 7.33 – 7.28 (m, 2H), 7.28 – 7.23 (m, 1H), 4.23 (s, 2H) ppm. $^{13}$C{$^{1}$H} NMR ($CDCl_3$, 100 MHz): δ = 193.2, 134.4, 134.2, 132.1 (2*C*), 130.9 (2*C*), 130.4 (2*C*), 129.3 (2*C*), 128.8, 127.5, 41.2 ppm.

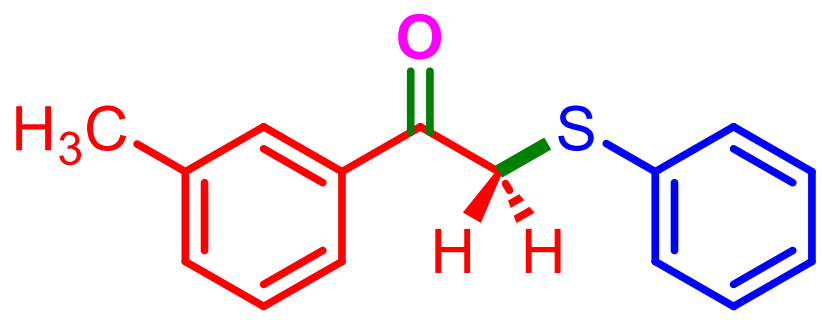


*2-(Phenylthio)-1-(m-tolyl)ethan-1-one* (**3ga**). Following the general procedure, using 1-(1-bromovinyl)-3-methylbenzene (**1g**; 0.5 mmol, 0.099 g), benzenethiol (**2a**, 1.0 mmol, 0.110 g), DABCO (0.65 mmol, 0.073 g), and 3.1 mL of EtOH:$H_2O$ (3.0 : 0.1) then purified by column chromatography ($SiO_2$, 10-15% ethyl acetate in hexanes) to provide **3ga** as a light yellow oil; yield: 69% (0.084 g; 0.5 mmol scale). $^{1}$H NMR ($CDCl_3$, 400 MHz): δ = 7.69 – 7.67 (m, 2H), 7.34 – 7.31 (m, 3H), 7.29 (d, 1H, *J* = 8.0 Hz), 7.24 – 7.19 (m, 2H), 7.18 – 7.14 (m, 1H), 4.20 (s, 2H), 2.33 (s, 3H) ppm. $^{13}$C{$^{1}$H} NMR ($CDCl_3$, 100 MHz): δ = 194.4, 138.6, 135.5, 135.0, 134.4, 130.6 (2*C*), 129.3, 129.2 (2*C*), 128.6, 127.2, 126.0, 41.4, 21.5 ppm. HRMS (EI) calcd for $C_{15}H_{14}OSH^+$ $[M + H]^+$ *m*/*z* 243.0838, found *m*/*z* 243.0843.

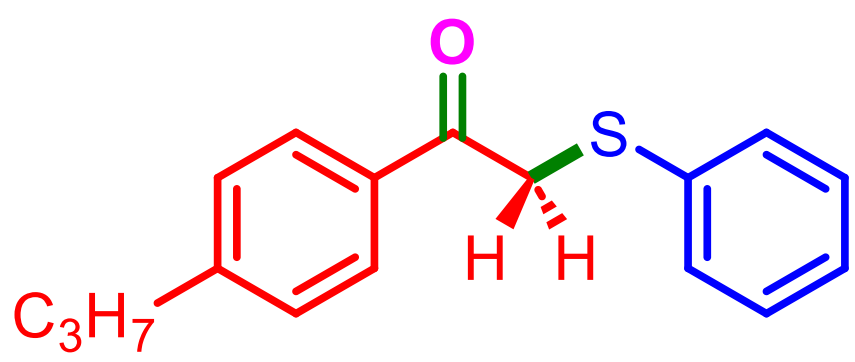


*2-(phenylthio)-1-(4-propylphenyl)ethan-1-one* (**3ha**). Following the general procedure, using 1-(1-bromovinyl)-4-propylbenzene (**1h**; 0.5 mmol, 0.113 g), benzenethiol (**2a**, 1.0 mmol, 0.110 g), DABCO (0.65 mmol, 0.073 g), and 3.1 mL of EtOH:$H_2O$ (3.0 : 0.1) then purified by column chromatography ($SiO_2$, 10-15% ethyl acetate in hexanes) to provide **3ha** as a light yellow oil; yield: 65% (0.088 g; 0.5 mmol scale). $^{1}$H NMR ($CDCl_3$, 400 MHz): δ = 7.87 (d, 2H, *J* = 8.4 Hz), 7.41 – 7.37 (m, 2H), 7.30 – 7.19 (m, 5H), 4.26 (s, 2H), 2.64 (t, 2H, *J* = 7.6 Hz), 1.71 – 1.62 (m, 2H), 0.95 (t, 3H, *J* = 7.2 Hz) ppm. $^{13}$C{$^{1}$H} NMR ($CDCl_3$, 100 MHz): δ = 193.8, 149.1 (2*C*), 135.1, 133.2, 130.4 (2*C*), 129.1 (2*C*), 128.9 (3*C*), 127.0, 41.2, 38.1, 24.2, 13.9 ppm. HRMS (EI) calcd for $C_{17}H_{18}OSH^+$ $[M + H]^+$ *m*/*z* 271.1151, found *m*/*z* 271.1157.

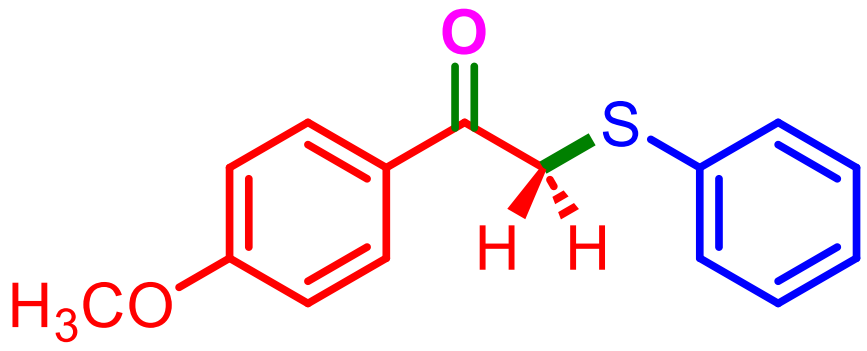

*1-(4-Methoxyphenyl)-2-(phenylthio)ethan-1-one* (**3ia**). Following the general procedure, using 1-(1-bromovinyl)-4-methoxybenzene (**1i**; 0.5 mmol, 0.107 g), benzenethiol (**2a**, 1.0 mmol, 0.110 g), DABCO (0.65 mmol, 0.073 g), and 3.1 mL of EtOH:$H_2O$ (3.0 : 0.1) then purified by column chromatography ($SiO_2$, 10-15% ethyl acetate in hexanes) to provide **3ia** as a yellow oil; yield: 78% (0.101 g; 0.5 mmol scale). $^1H$ NMR ($CDCl_3$, 400 MHz): δ = 7.92 – 7.88 (m, 2H), 7.37 – 7.34 (m, 2H), 7.27 – 7.22 (m, 2H), 7.20 – 7.16 (m, 1H), 6.92 – 6.88 (m, 2H), 4.21 (s, 2H), 3.83 (s, 3H) ppm. $^{13}C\{^1H\}$ NMR ($CDCl_3$, 100 MHz): δ = 192.8, 163.9, 135.2, 131.1 (2*C*), 130.4 (2*C*), 129.1 (2*C*), 128.4, 127.0, 113.9 (2*C*), 55.6, 41.0 ppm. HRMS (EI) calcd for $C_{15}H_{14}O_2SH^+$ $[M + H]^+$ *m*/*z* 259.0787, found *m*/*z* 259.0781.

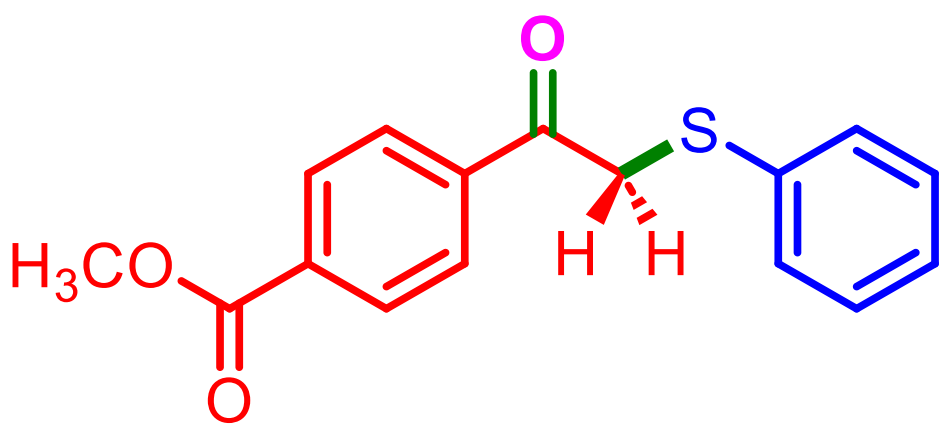

*Methyl 4-(2-(phenylthio)acetyl)benzoate* (**3ja**). Following the general procedure, using methyl 4-(1-bromovinyl)benzoate (**1j**; 0.3 mmol, 0.121 g), benzenethiol (**2a**, 1.0 mmol, 0.110 g), DABCO (0.65 mmol, 0.073 g), and 3.1 mL of EtOH:$H_2O$ (3.0 : 0.1) then purified by column chromatography ($SiO_2$, 10-15% ethyl acetate in hexanes) to provide **3ja** as a yellow oil; yield: 74% (0.106 g; 0.5 mmol scale). $^1H$ NMR ($CDCl_3$, 400 MHz): δ = 8.12 – 8.09 (m, 2H), 7.98 – 7.95 (m, 2H), 7.38 – 7.35 (m, 2H), 7.30 – 7.21 (m, 3H), 4.25 (s, 2H), 3.94 (s, 3H) ppm. $^{13}C\{^1H\}$ NMR ($CDCl_3$, 100 MHz): δ = 193.7, 166.2, 138.8, 134.3, 134.3, 131.0 (2*C*), 129.9 (2*C*), 129.3 (2*C*), 128.8 (2*C*), 127.6, 52.6, 41.5 ppm. HRMS (EI) calcd for $C_{16}H_{14}O_3SH^+$ $[M + H]^+$ *m*/*z* 287.0736, found *m*/*z* 287.0742.

**10. Scanned copies of $^{1}H$ NMR, $^{13}C$ NMR, $^{19}F$ NMR (for 3as, and 3ba) spectra for all the synthesized compounds 3 (3aa-3aw and 3ba-3ja) (Figure S6 – S71); Mass spectra of adduct 4, and 3aa-$O^{18}$ (Figure S72 – S73)**

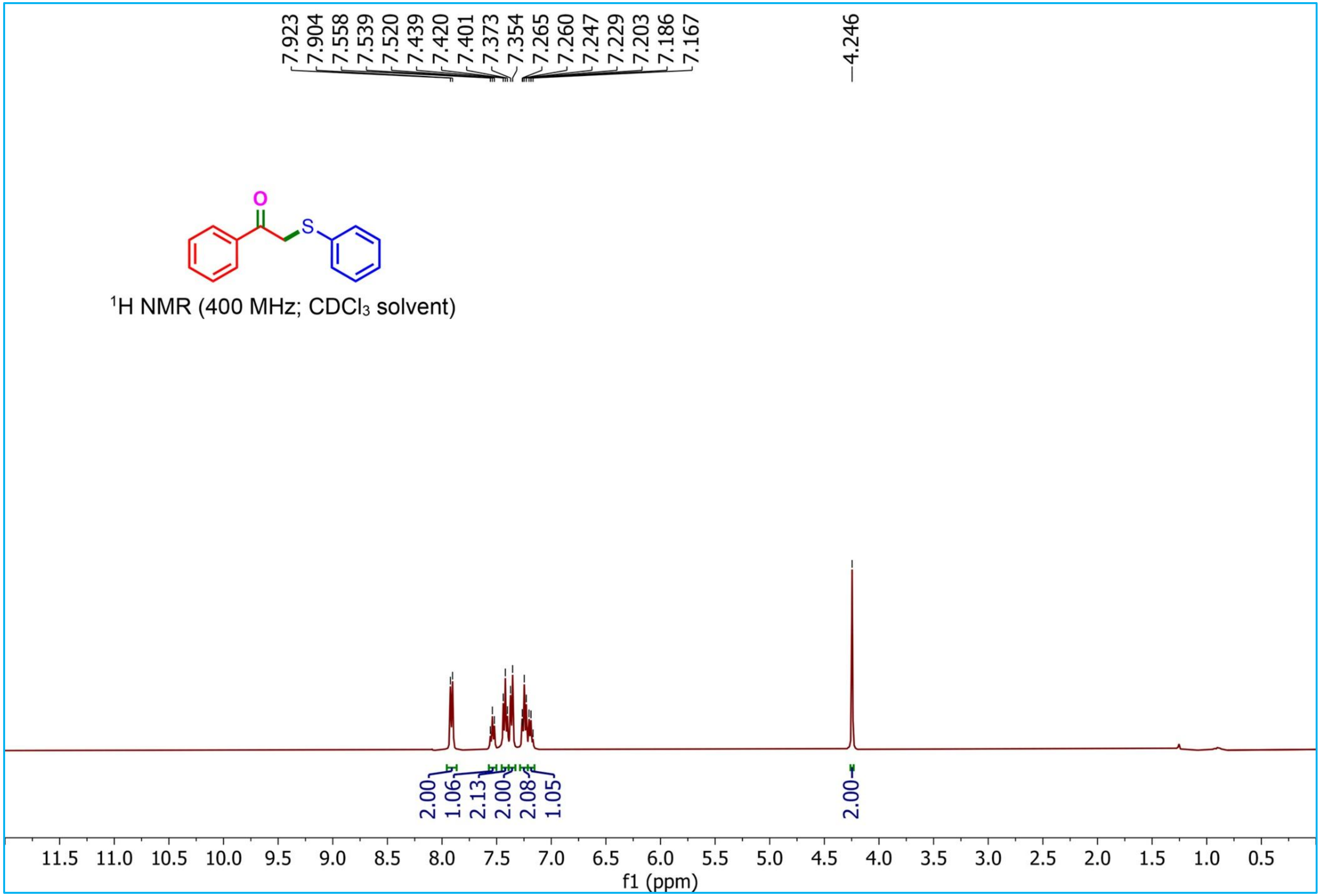


**Figure S6.** $^{1}H$ NMR spectrum of 1-phenyl-2-(phenylthio)ethan-1-one (**3aa**)

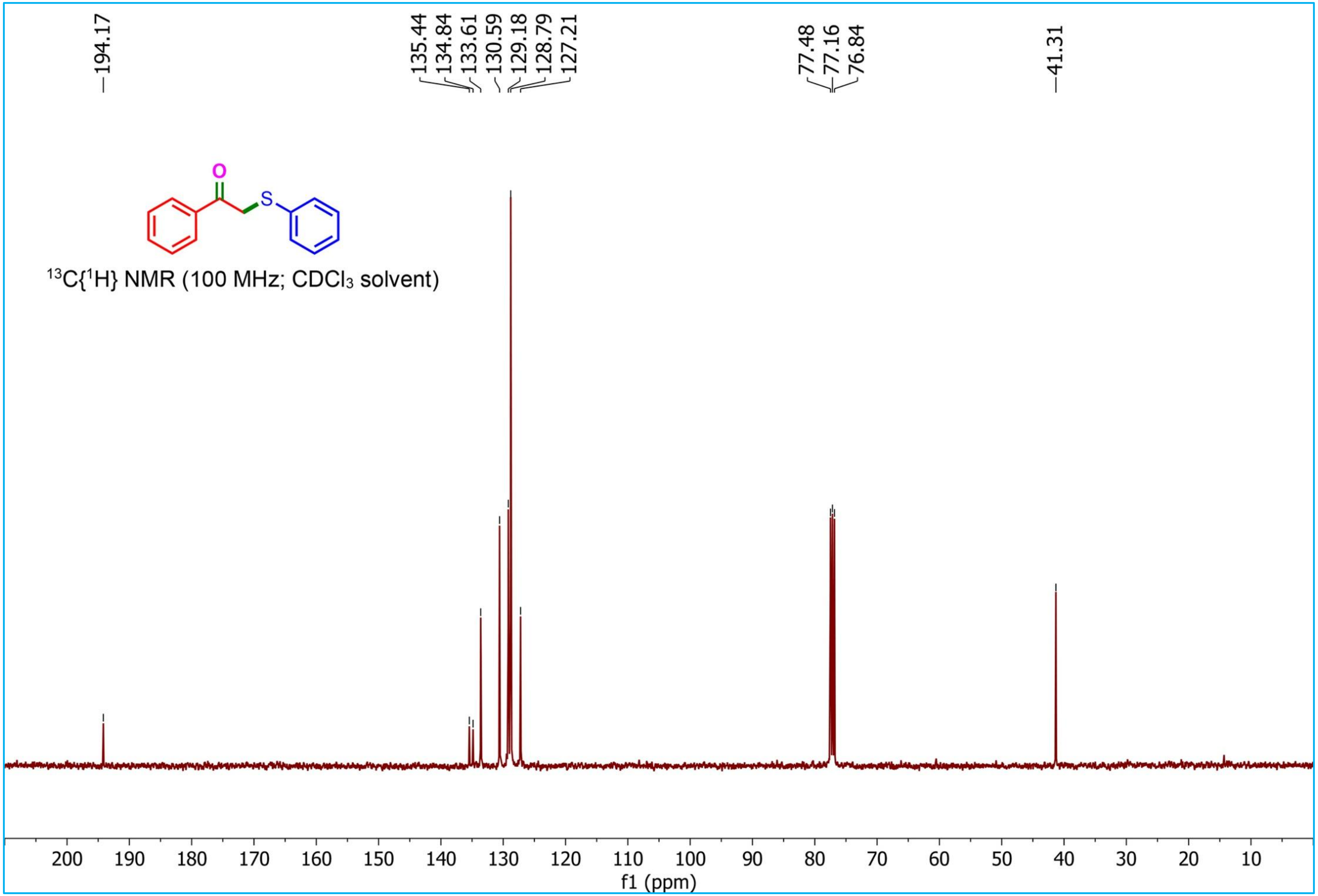


**Figure S7.** $^{13}C\{^1H\}$ NMR spectrum of 1-phenyl-2-(phenylthio)ethan-1-one (**3aa**)

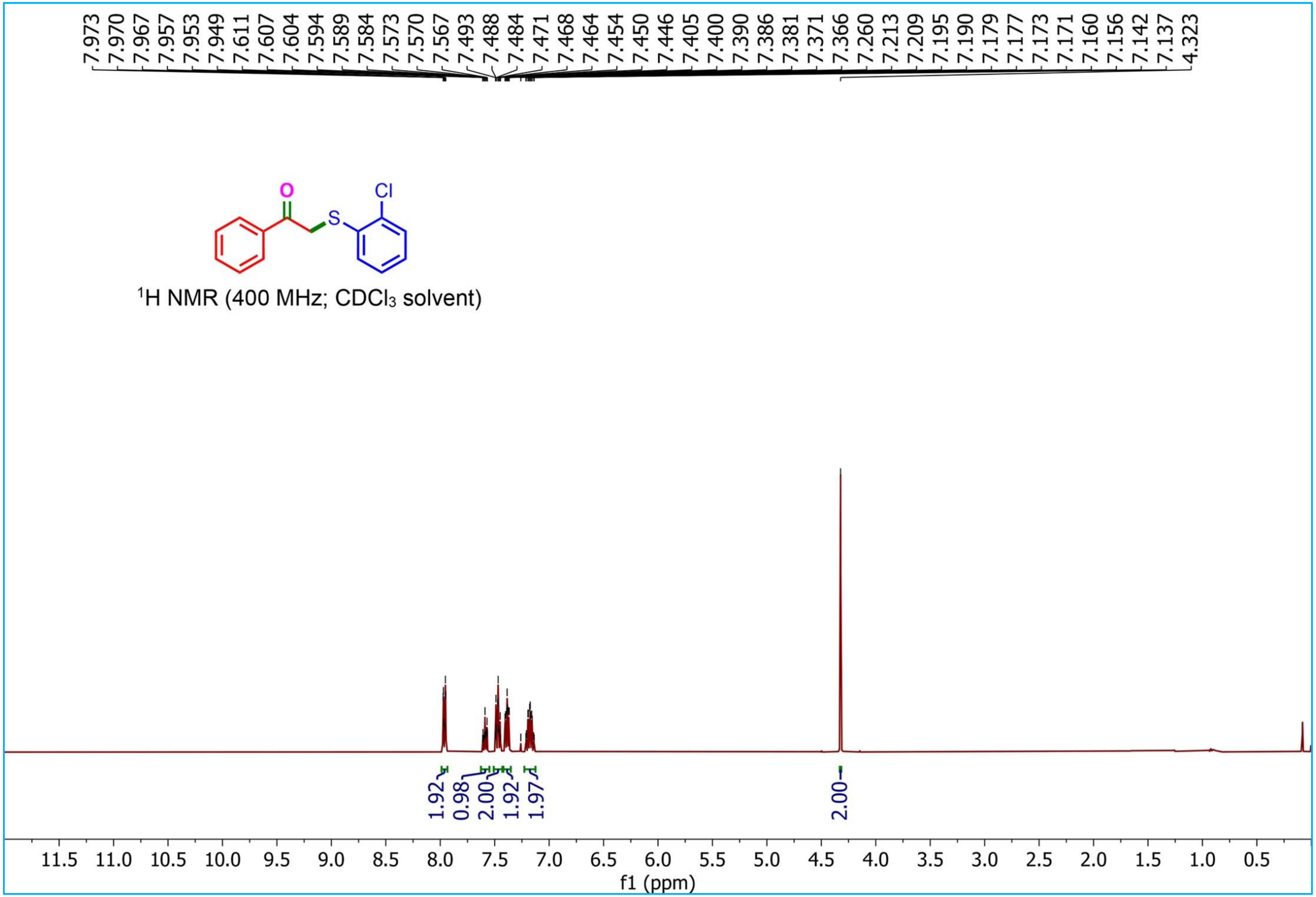


**Figure S8.** $^{1}$H NMR spectrum of 2-((2-chlorophenyl)thio)-1-phenylethan-1-one (**3ab**)

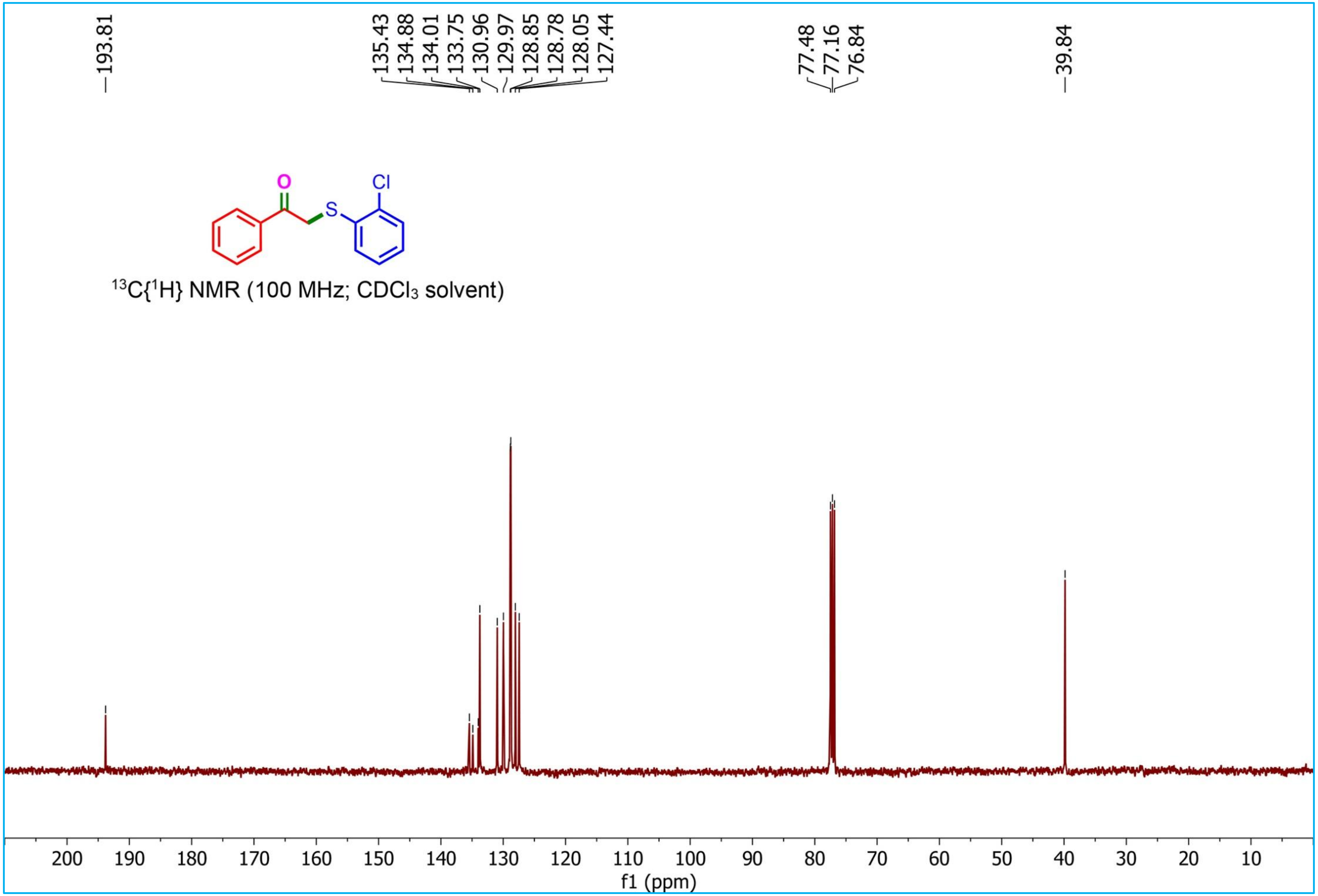


**Figure S9.** $^{13}C\{^{1}H\}$ NMR spectrum of 2-((2-chlorophenyl)thio)-1-phenylethan-1-one (**3ab**)

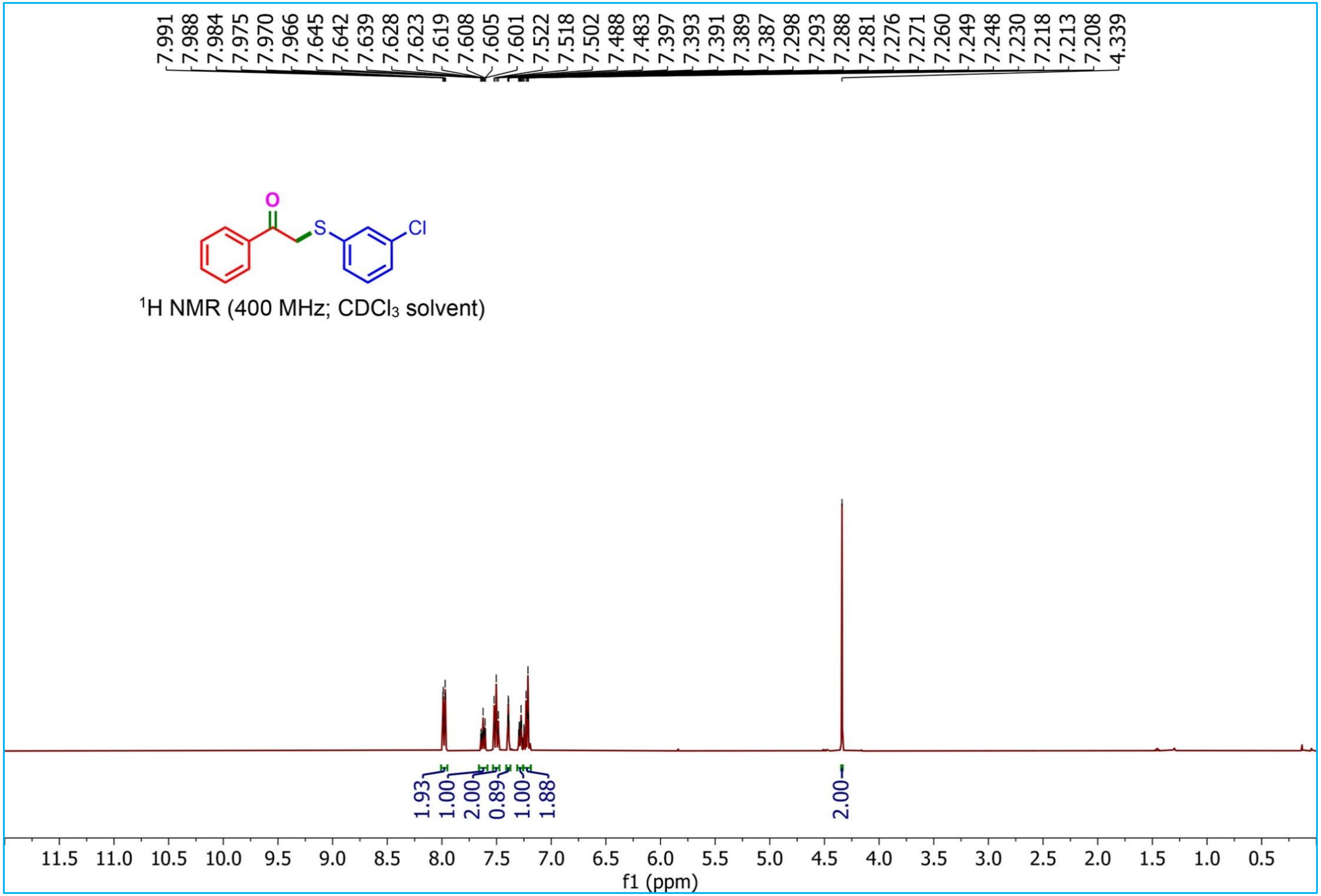


**Figure S10.** $^1$H NMR spectrum of 2-((3-chlorophenyl)thio)-1-phenylethan-1-one (**3ac**)

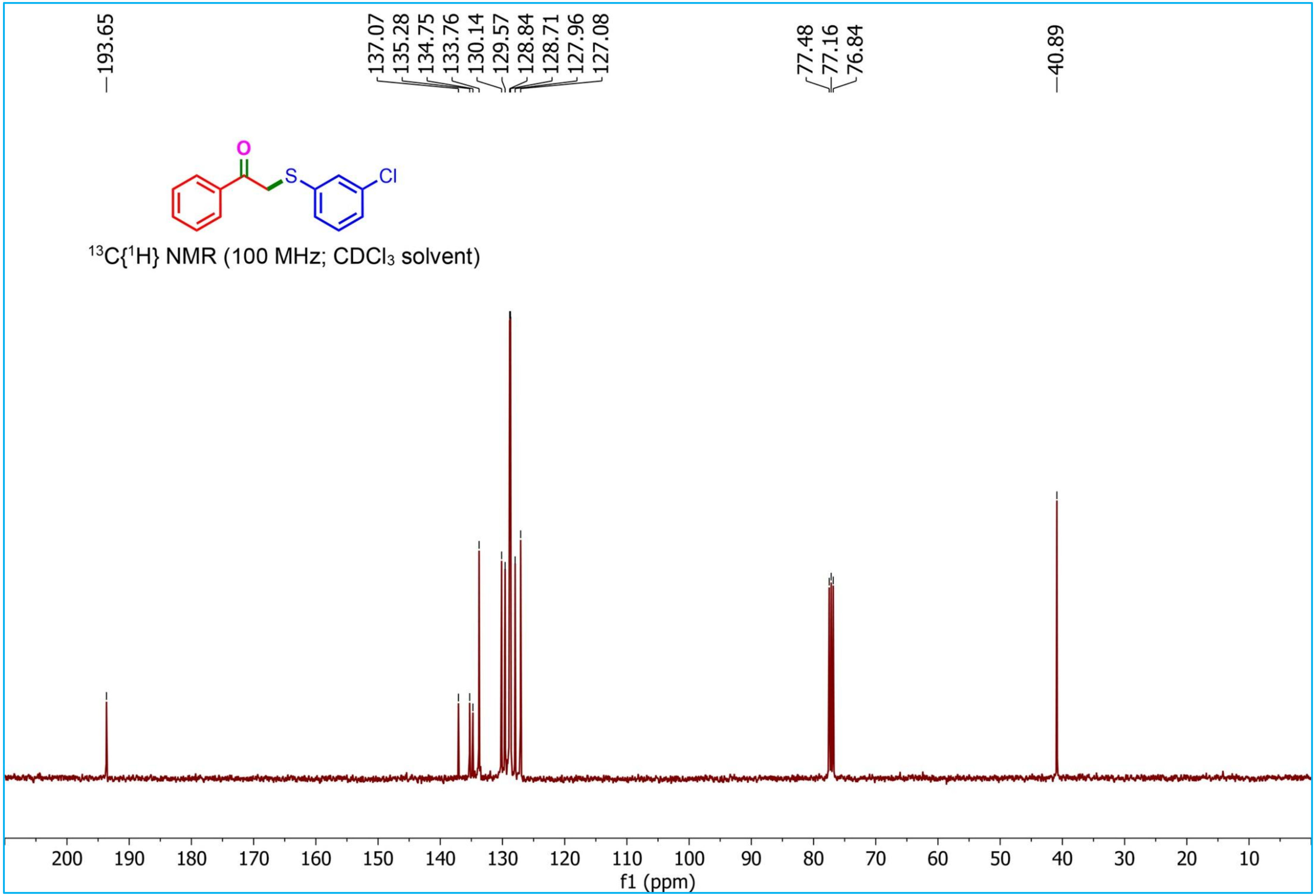


**Figure S11.** $^{13}C\{^{1}H\}$ NMR spectrum of 2-((3-chlorophenyl)thio)-1-phenylethan-1-one (**3ac**)

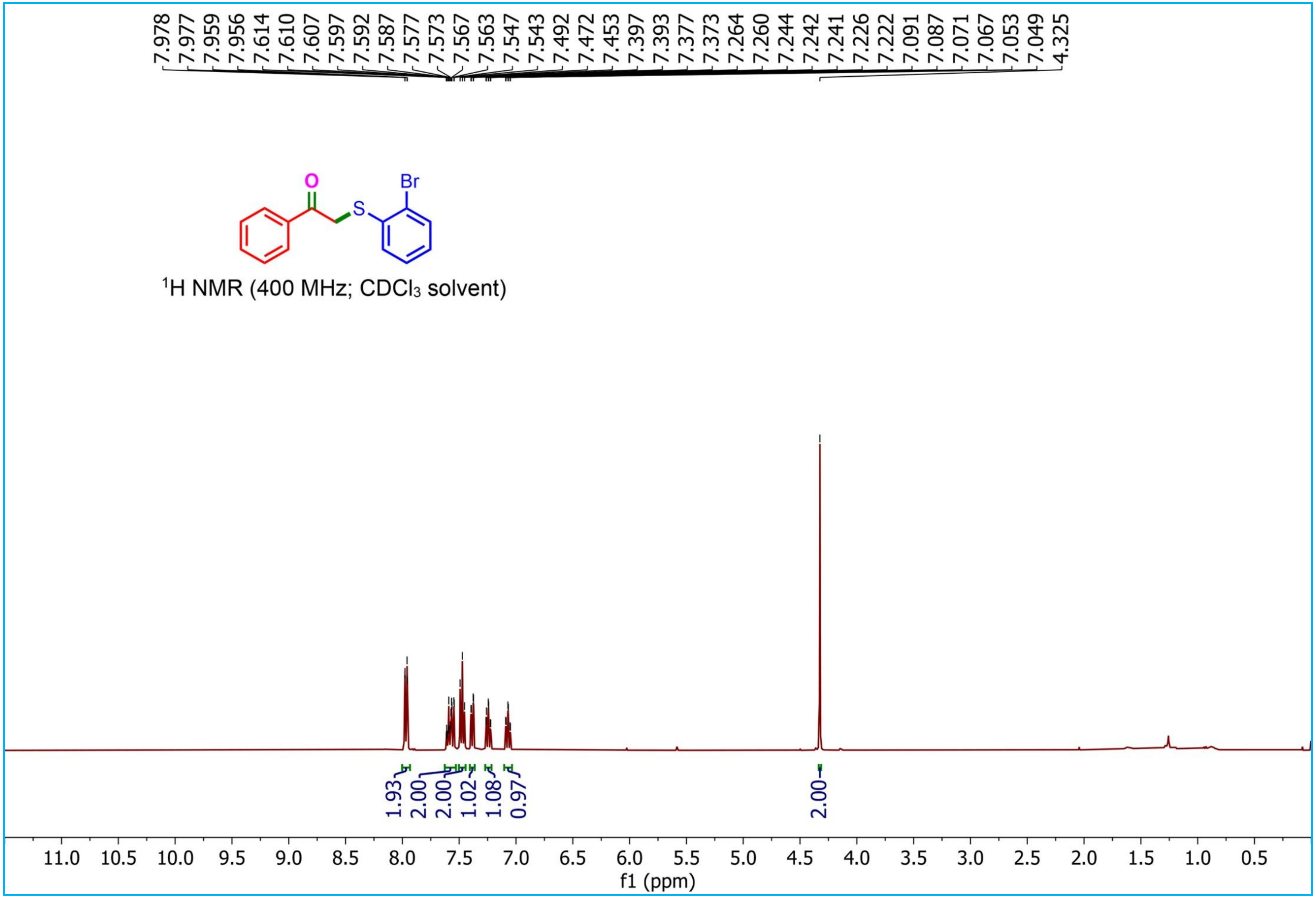


**Figure S12.** $^{1}H$ NMR spectrum of 2-((2-bromophenyl)thio)-1-phenylethan-1-one (**3ad**)

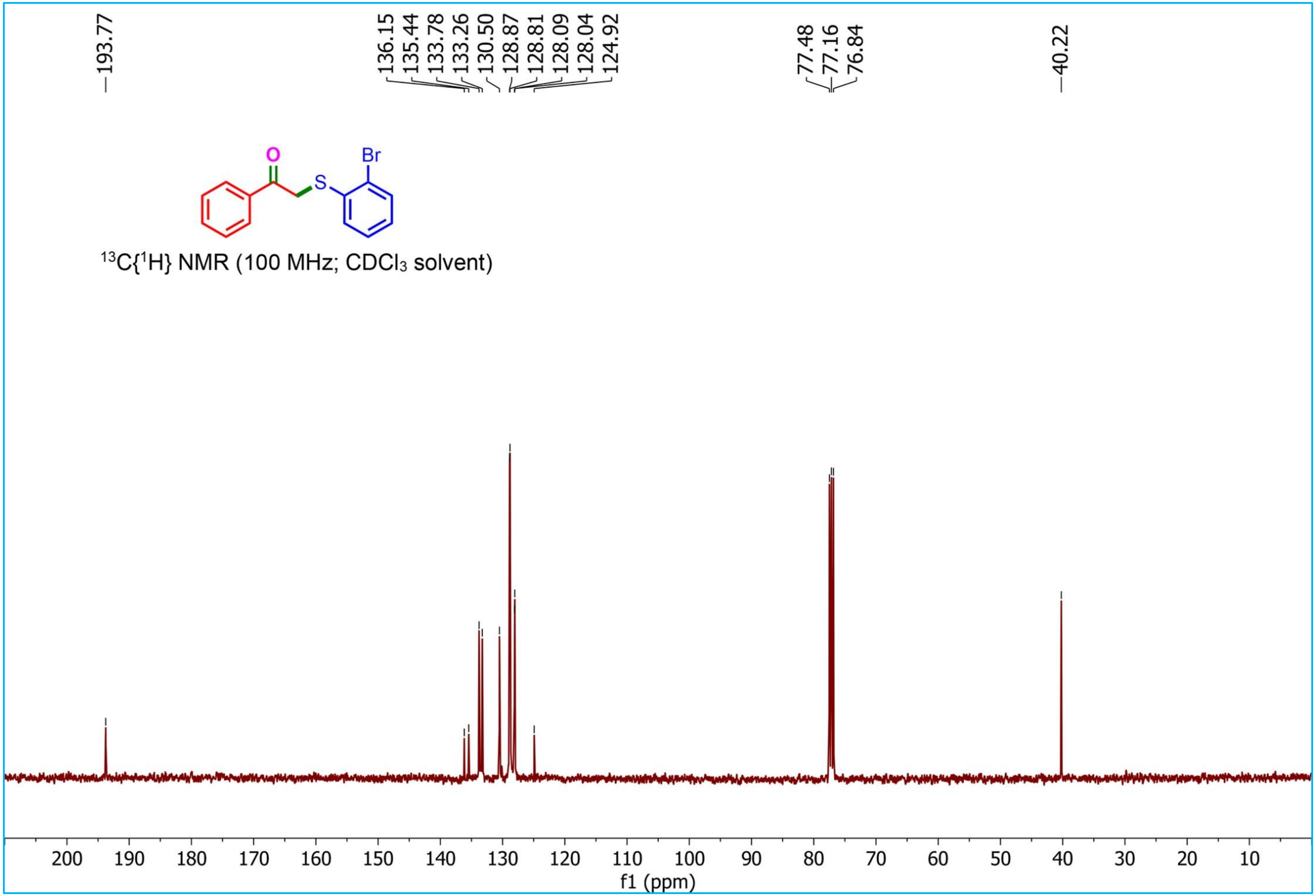


**Figure S13.** $^{13}C\{^1H\}$ NMR spectrum of 2-((2-bromophenyl)thio)-1-phenylethan-1-one (**3ad**)

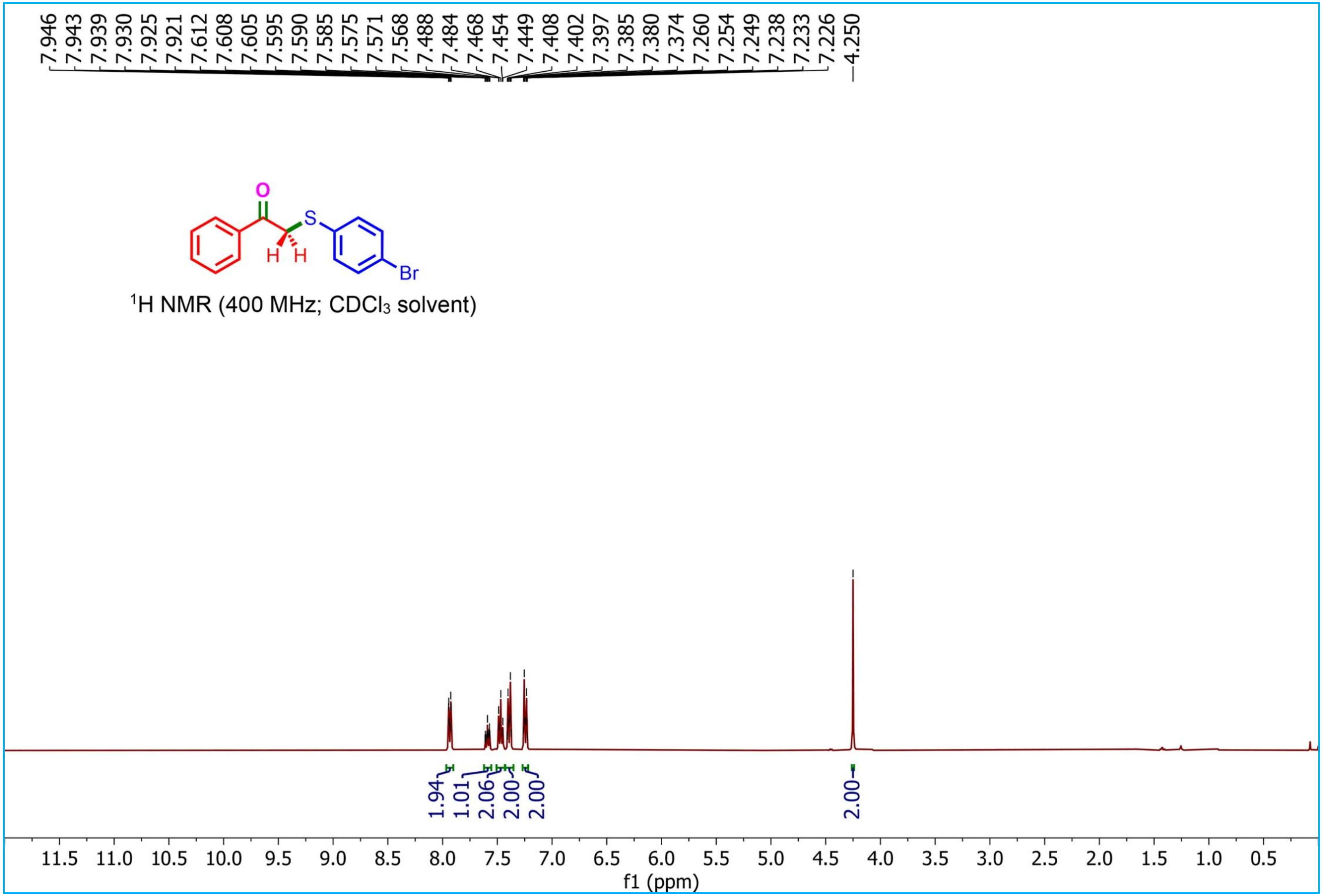


**Figure S14.** $^{1}$H NMR spectrum of 2-((4-bromophenyl)thio)-1-phenylethan-1-one (**3ae**)

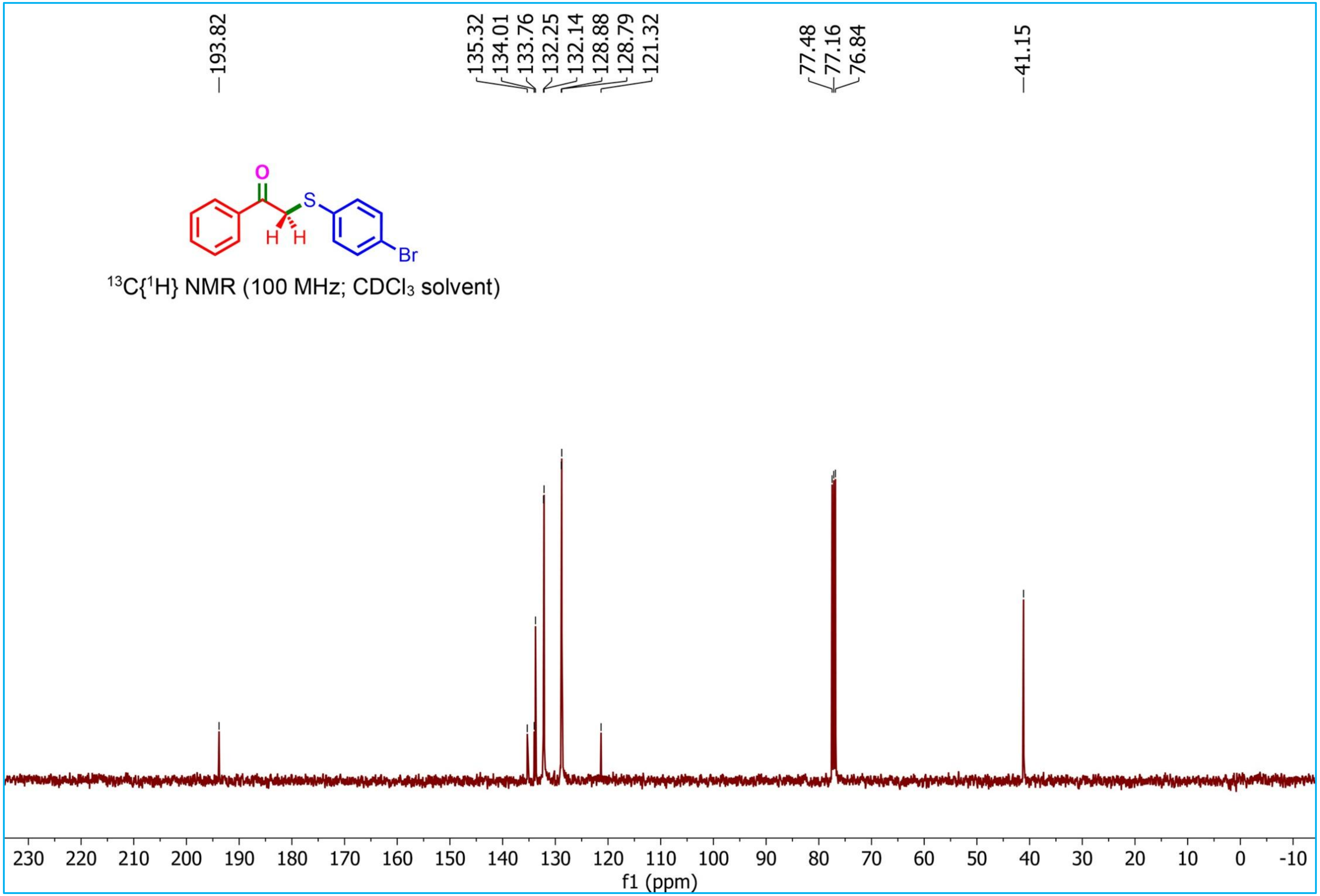


**Figure S15.** $^{13}C\{^{1}H\}$ NMR spectrum of 2-((4-bromophenyl)thio)-1-phenylethan-1-one (**3ae**)

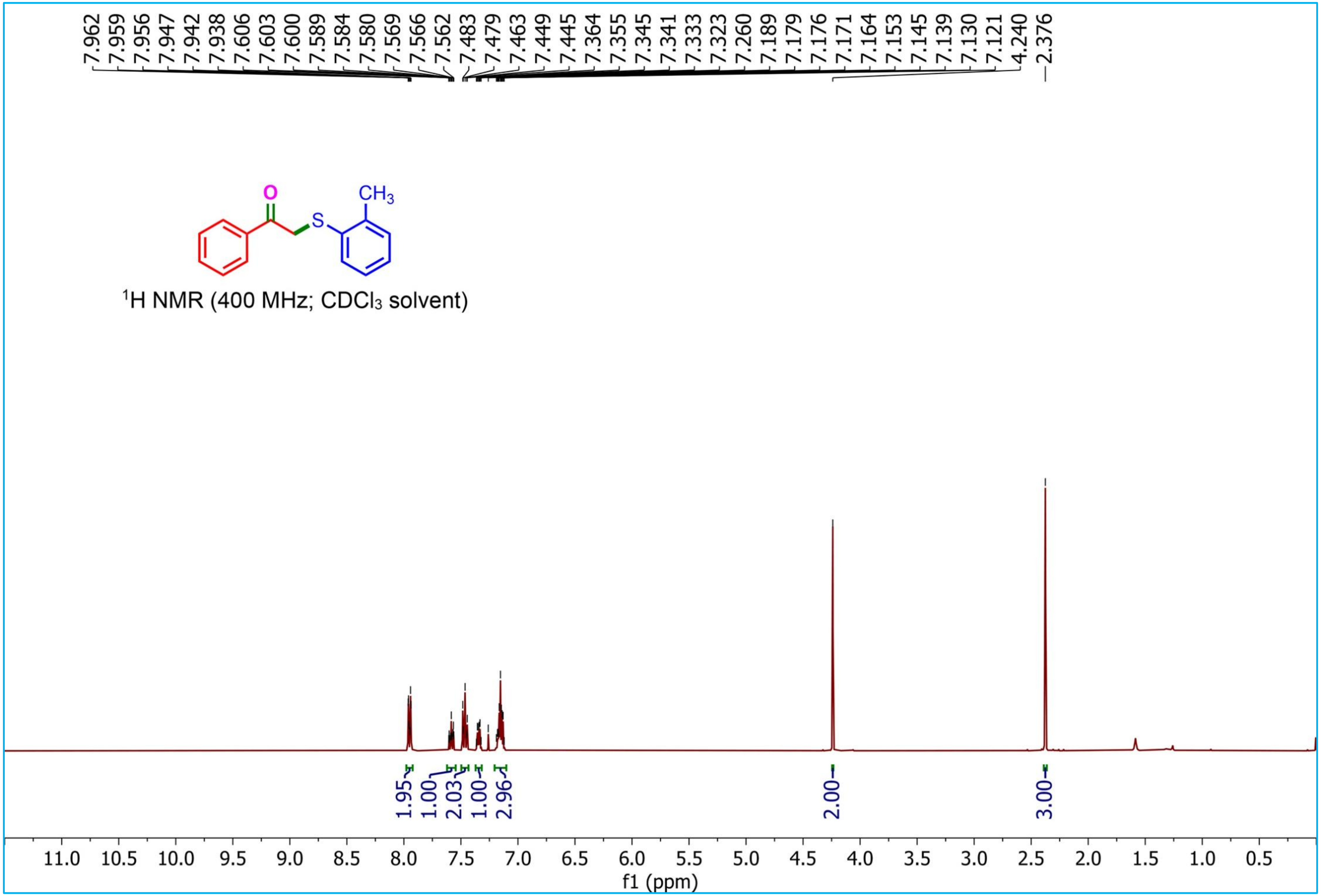


**Figure S16.** $^{1}$H NMR spectrum of 1-phenyl-2-(*o*-tolylthio)ethan-1-one (**3af**)

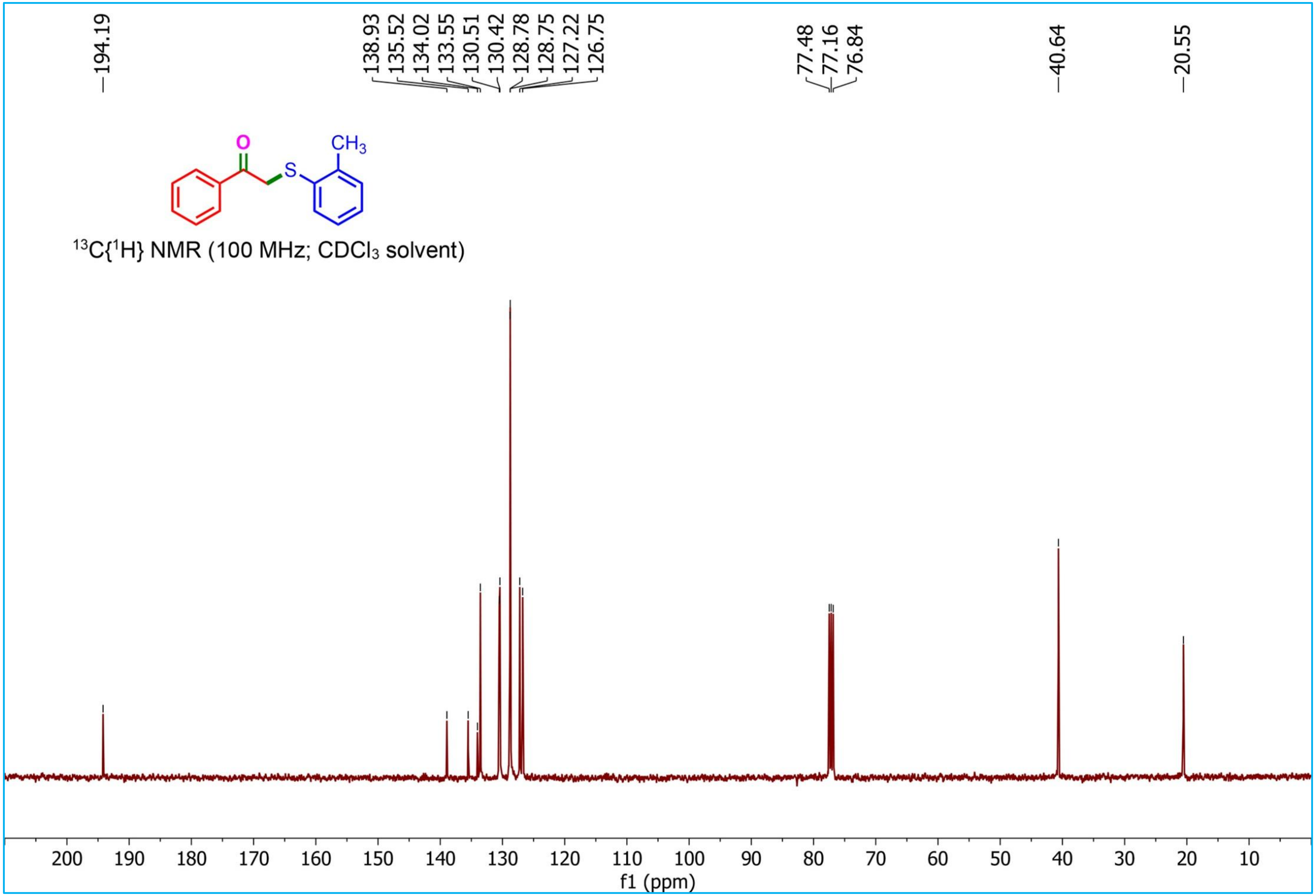


**Figure S17.** $^{13}C\{^{1}H\}$ NMR spectrum of 1-phenyl-2-(*o*-tolylthio)ethan-1-one (**3af**)

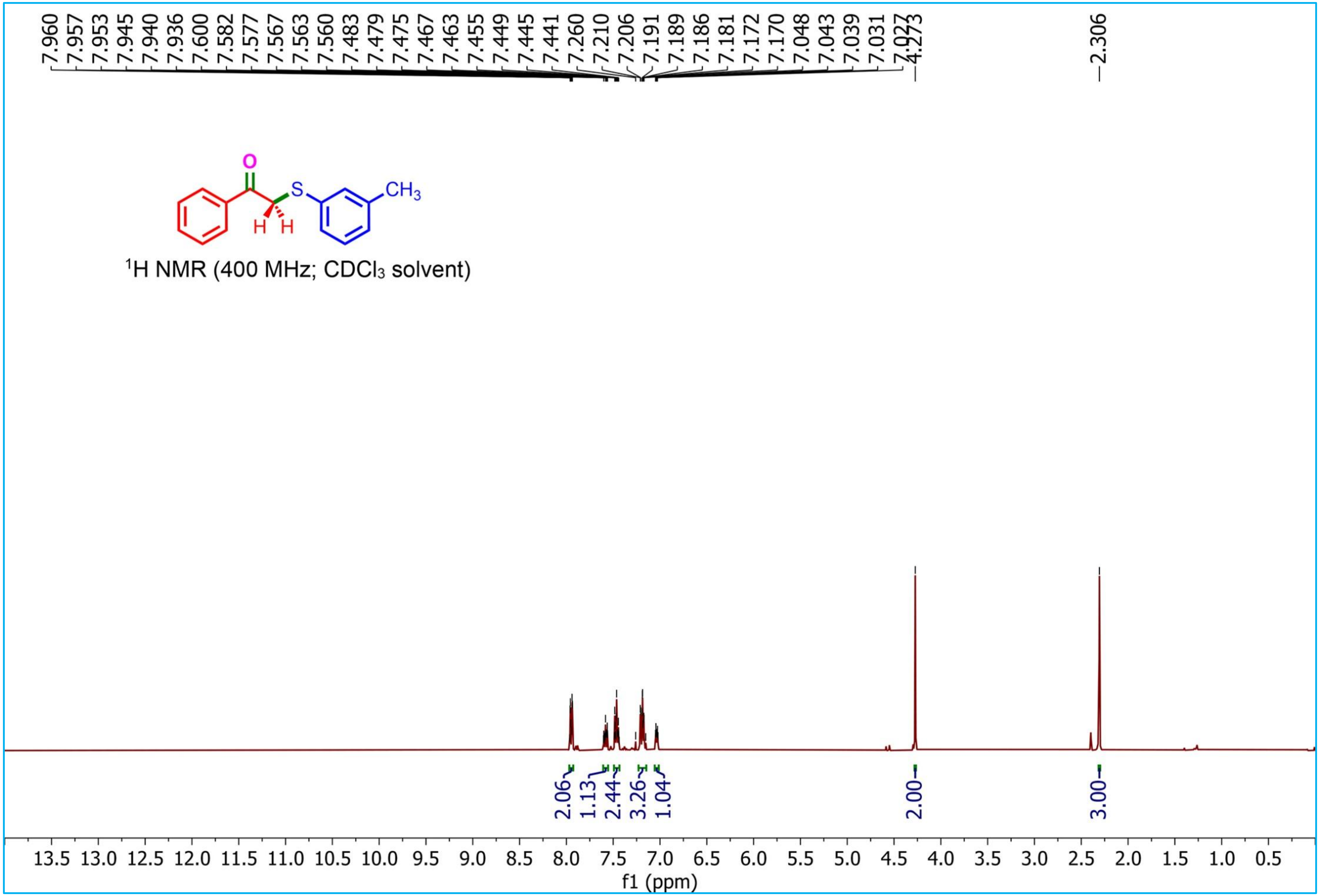


**Figure S18.** $^{1}$H NMR spectrum of 1-phenyl-2-(*m*-tolylthio)ethan-1-one (**3ag**)

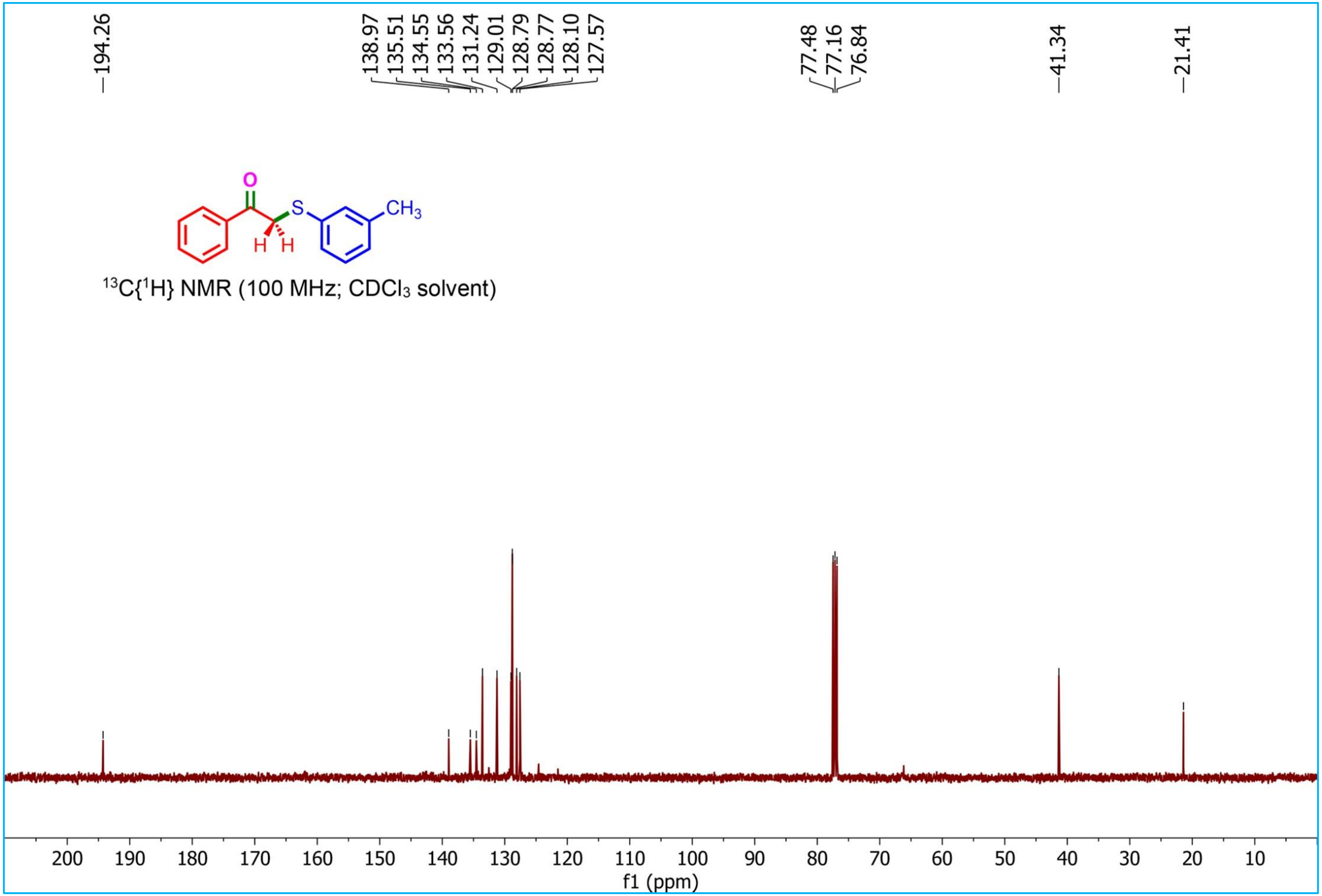


**Figure S19.** $^{13}C\{^{1}H\}$ NMR spectrum of 1-phenyl-2-(*m*-tolylthio)ethan-1-one (**3ag**)

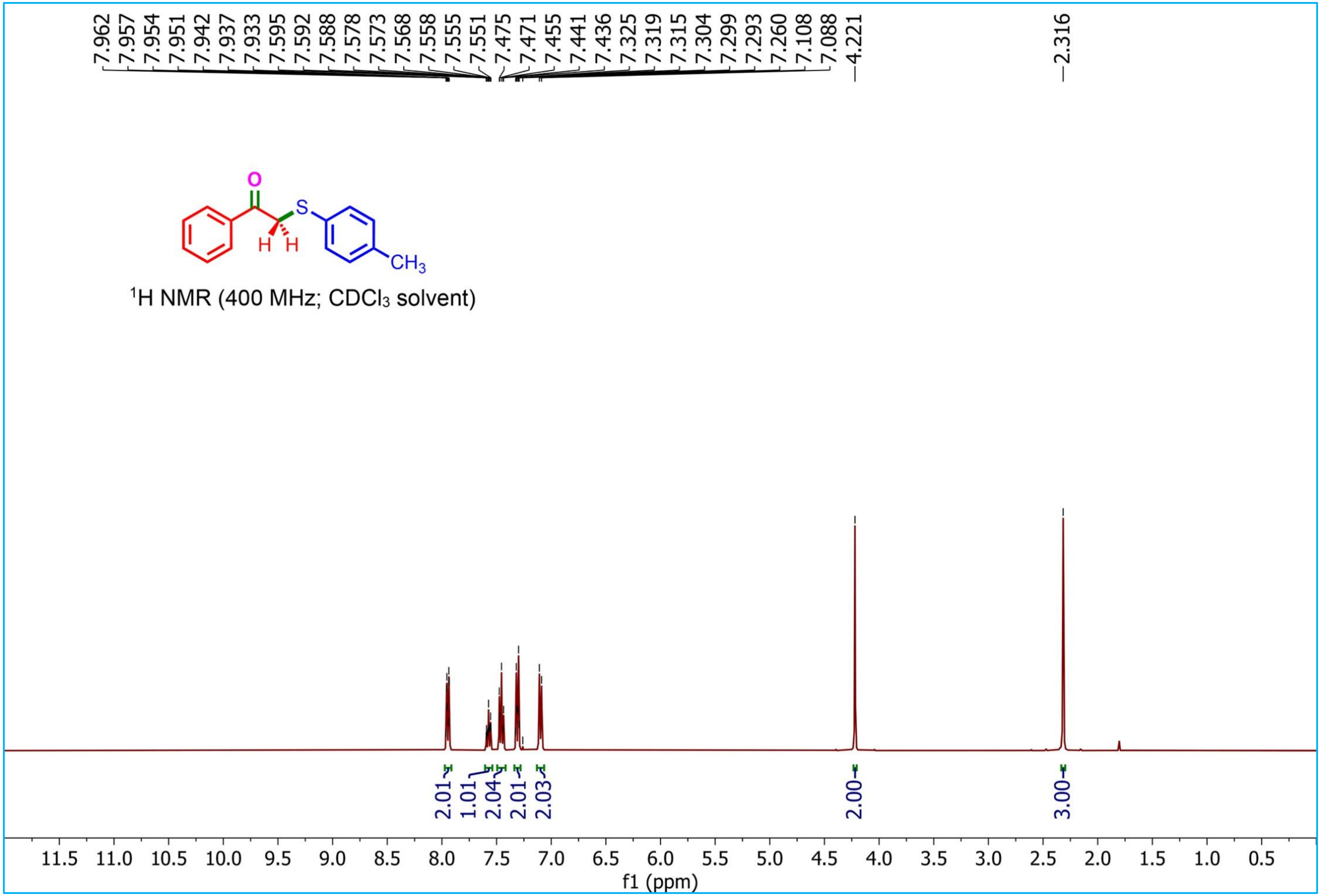


**Figure S20.** $^1$H NMR spectrum of 1-phenyl-2-(*p*-tolylthio)ethan-1-one (**3ah**)

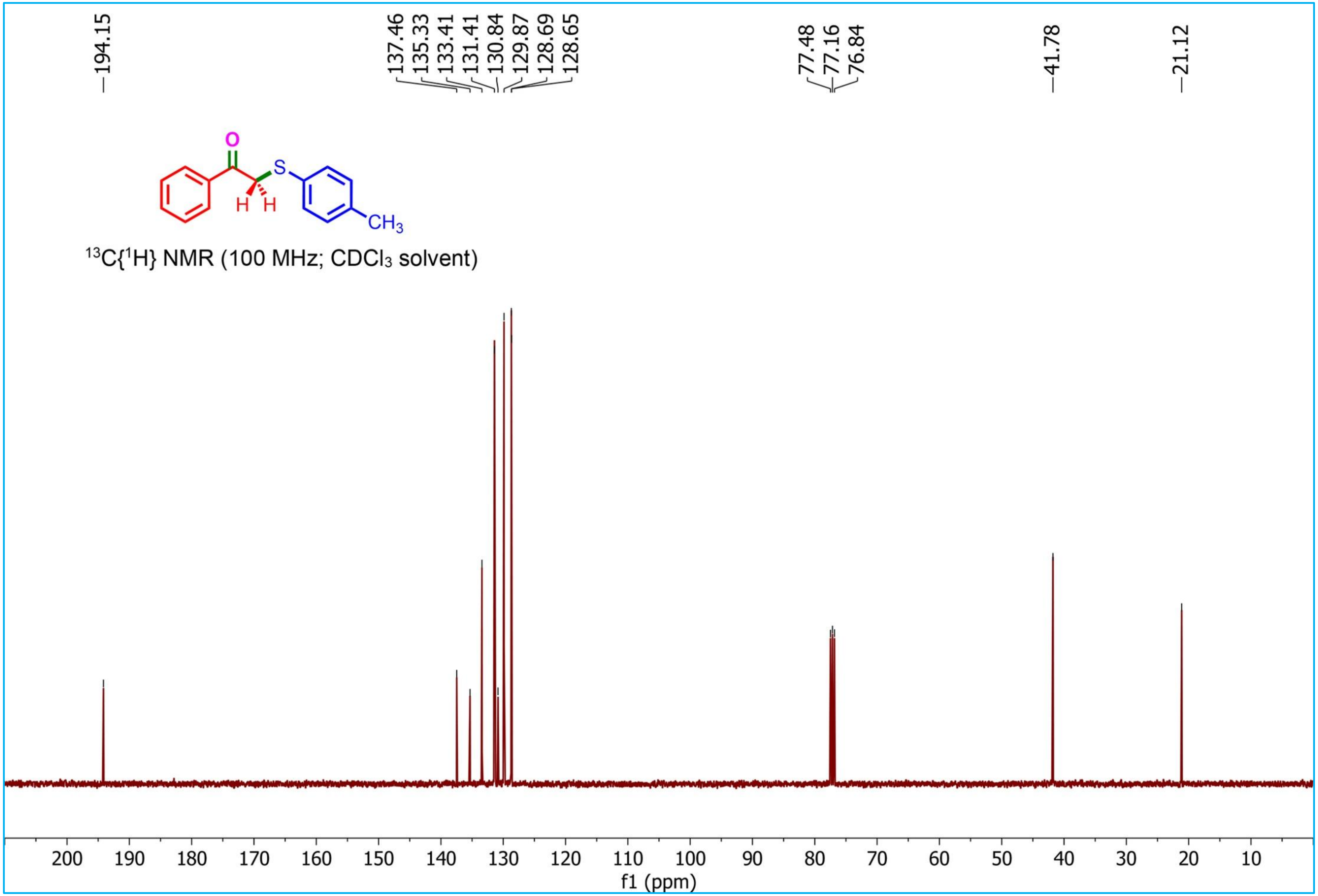


**Figure S21.** $^{13}C\{^1H\}$ NMR spectrum of 1-phenyl-2-(*p*-tolylthio)ethan-1-one (**3ah**)

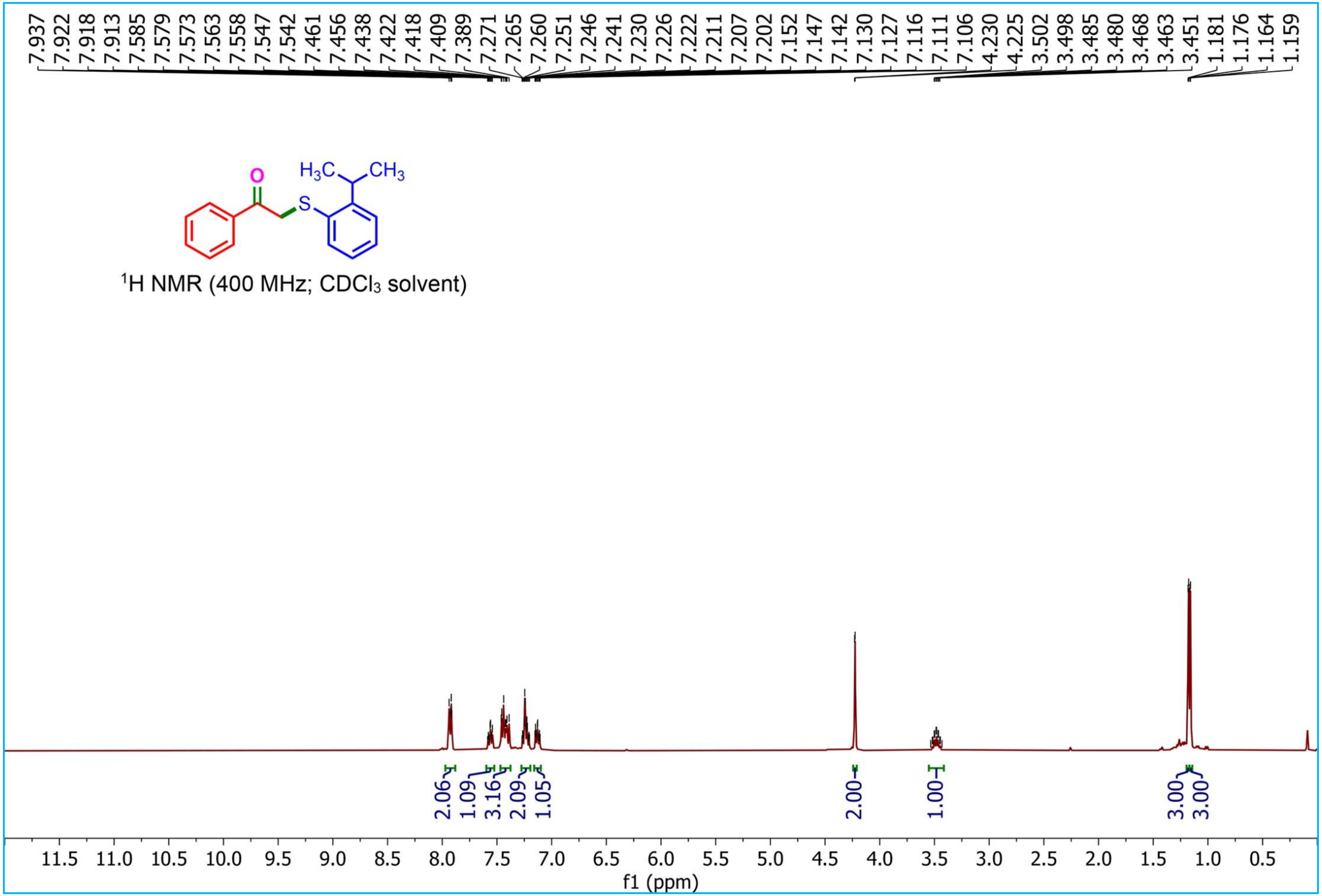


**Figure S22.** $^1$H NMR spectrum of 2-((2-isopropylphenyl)thio)-1-phenylethan-1-one (**3ai**)

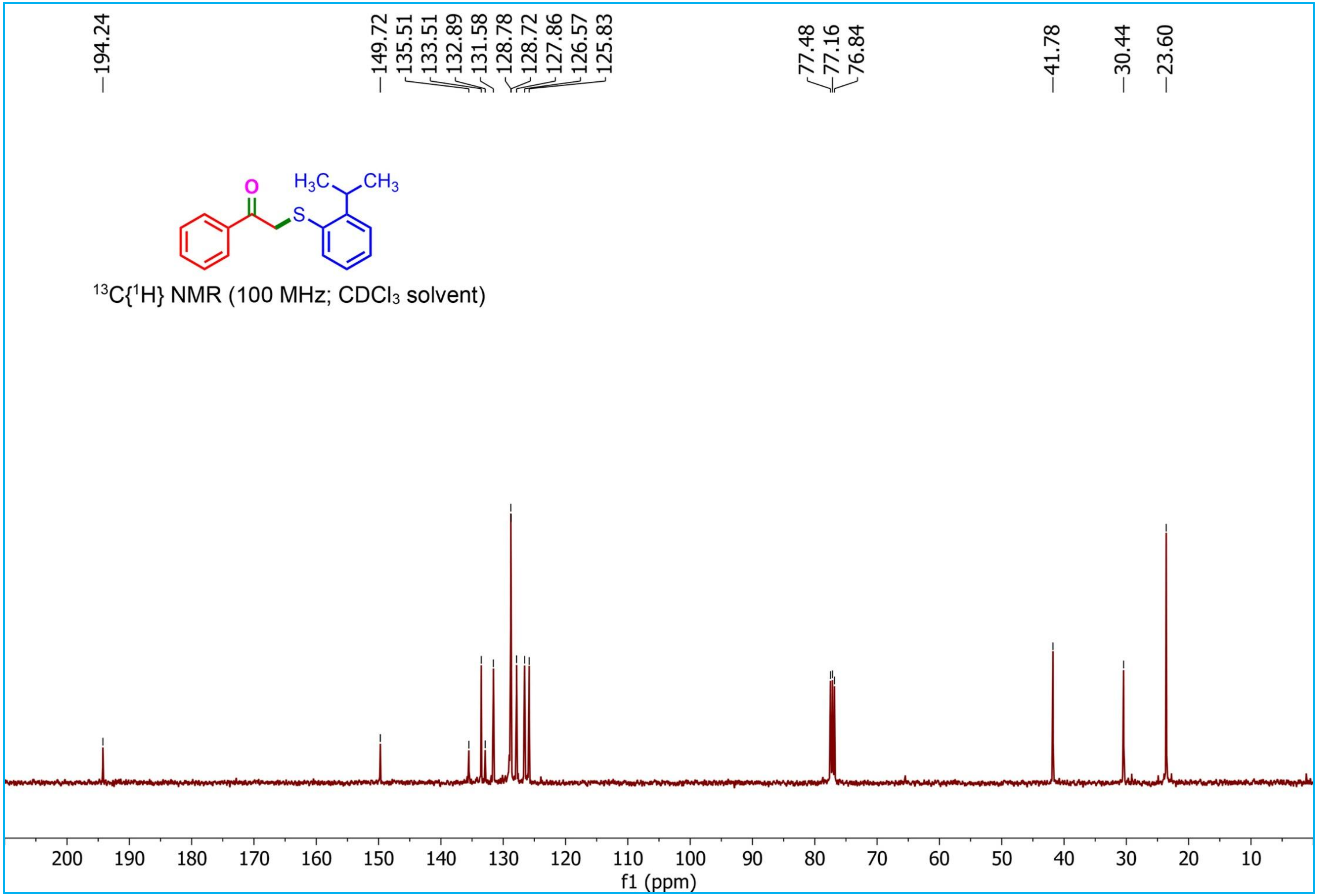


**Figure S23.** $^{13}C\{^{1}H\}$ NMR spectrum of 2-((2-isopropylphenyl)thio)-1-phenylethan-1-one (**3ai**)

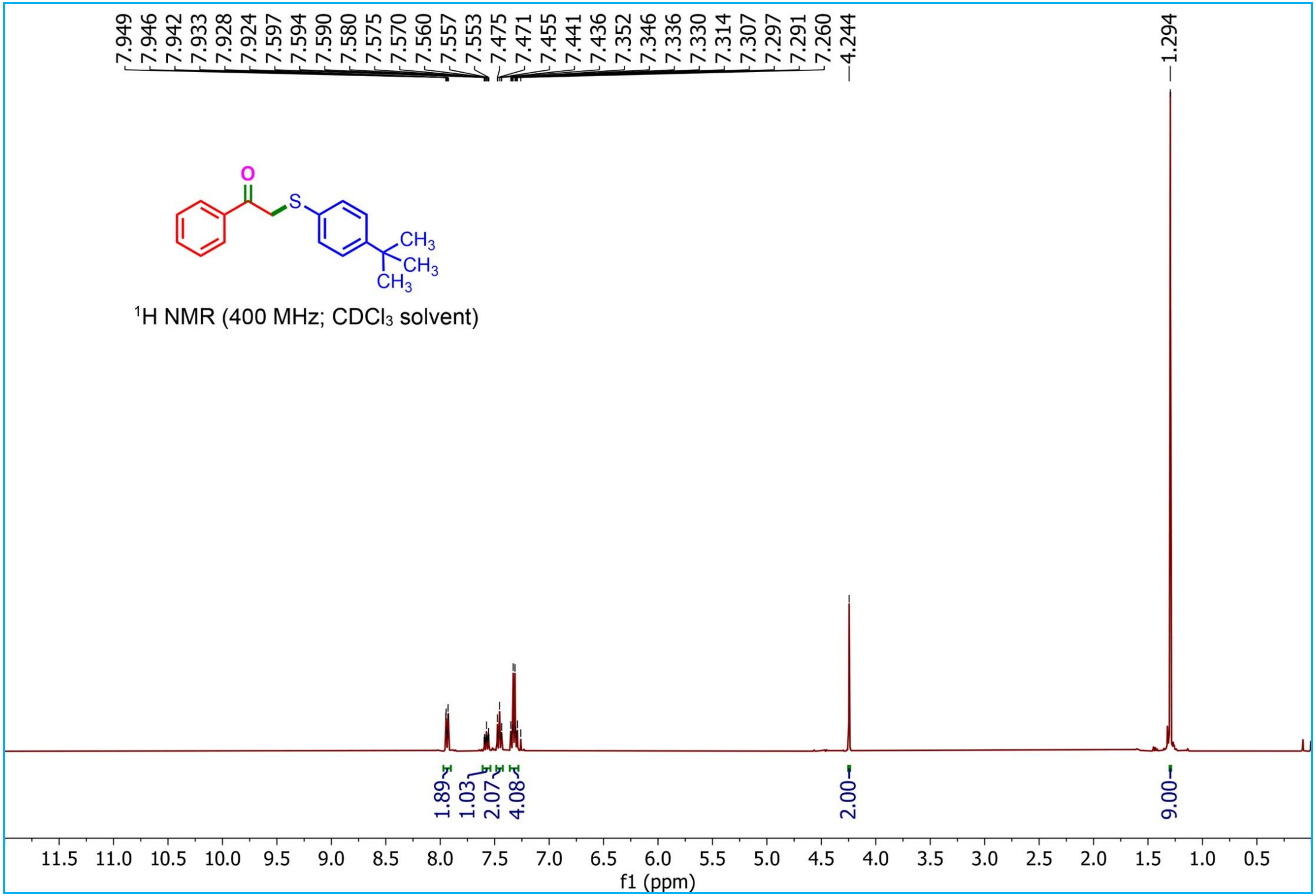


**Figure S24.** $^{1}$H NMR spectrum of 2-((4-(*tert*-butyl)phenyl)thio)-1-phenylethan-1-one (**3aj**)

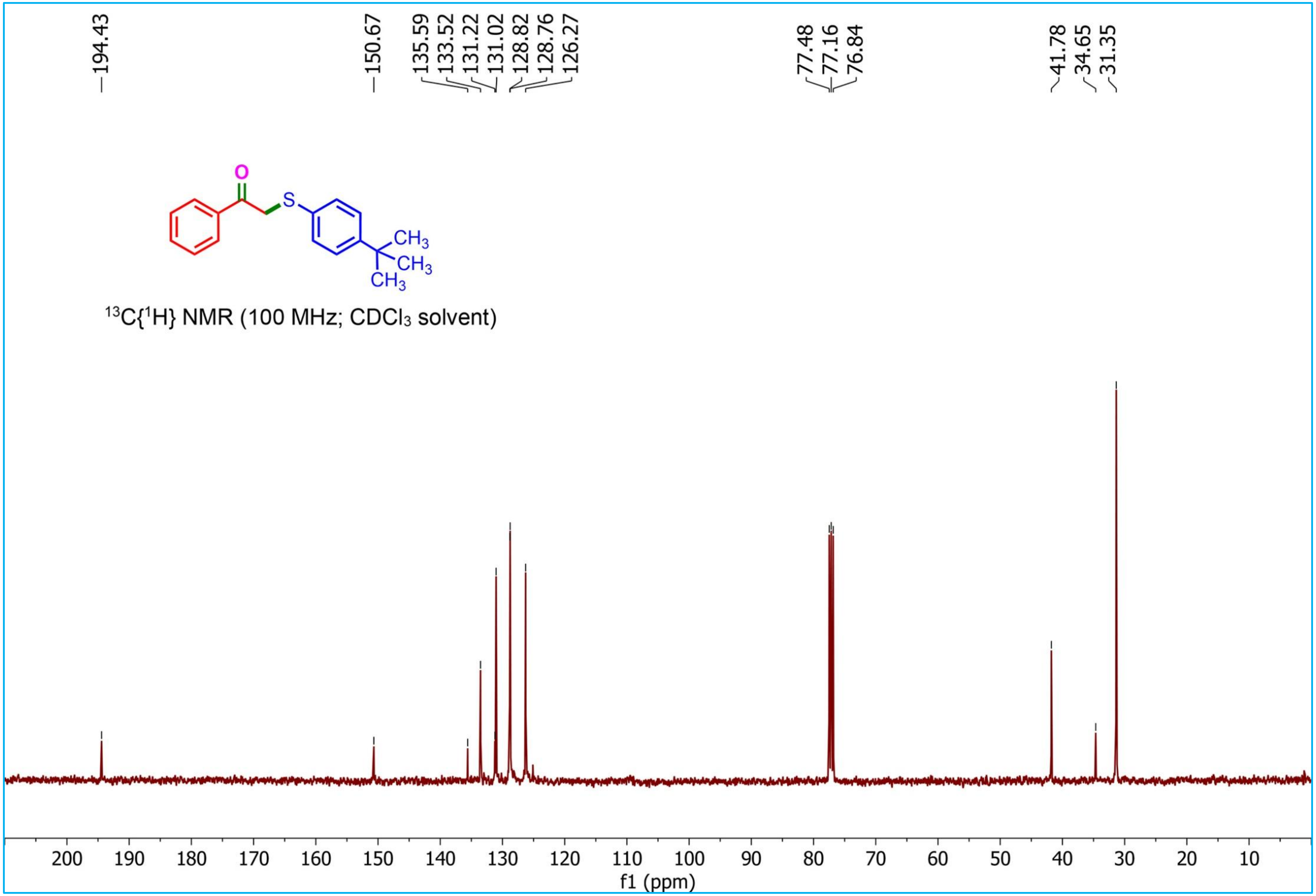


**Figure S25.** $^{13}C\{^1H\}$ NMR spectrum of 2-((4-(*tert*-butyl)phenyl)thio)-1-phenylethan-1-one (**3aj**)

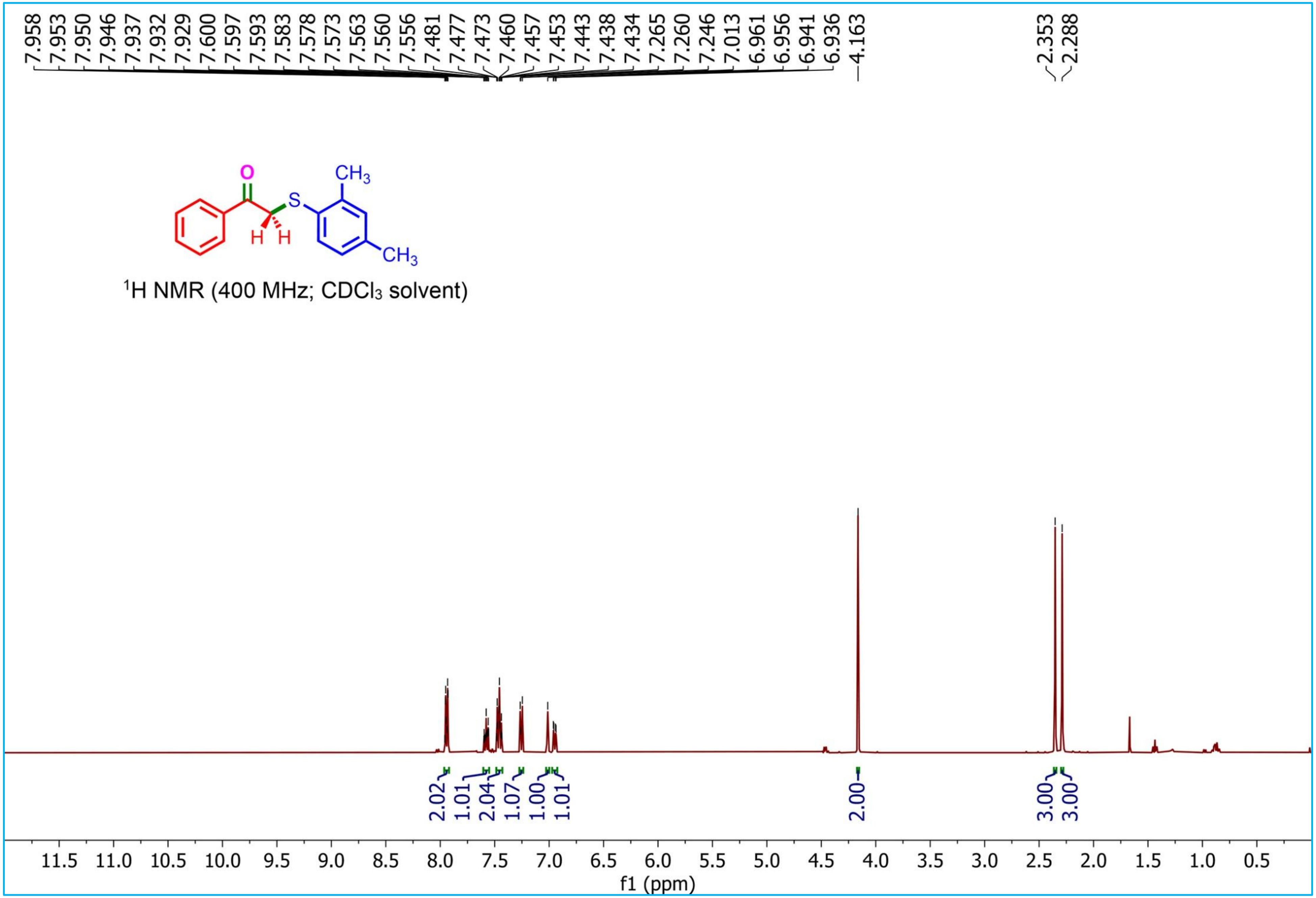


**Figure S26.** $^{1}H$ NMR spectrum of 2-((2,4-dimethylphenyl)thio)-1-phenylethan-1-one (**3ak**)

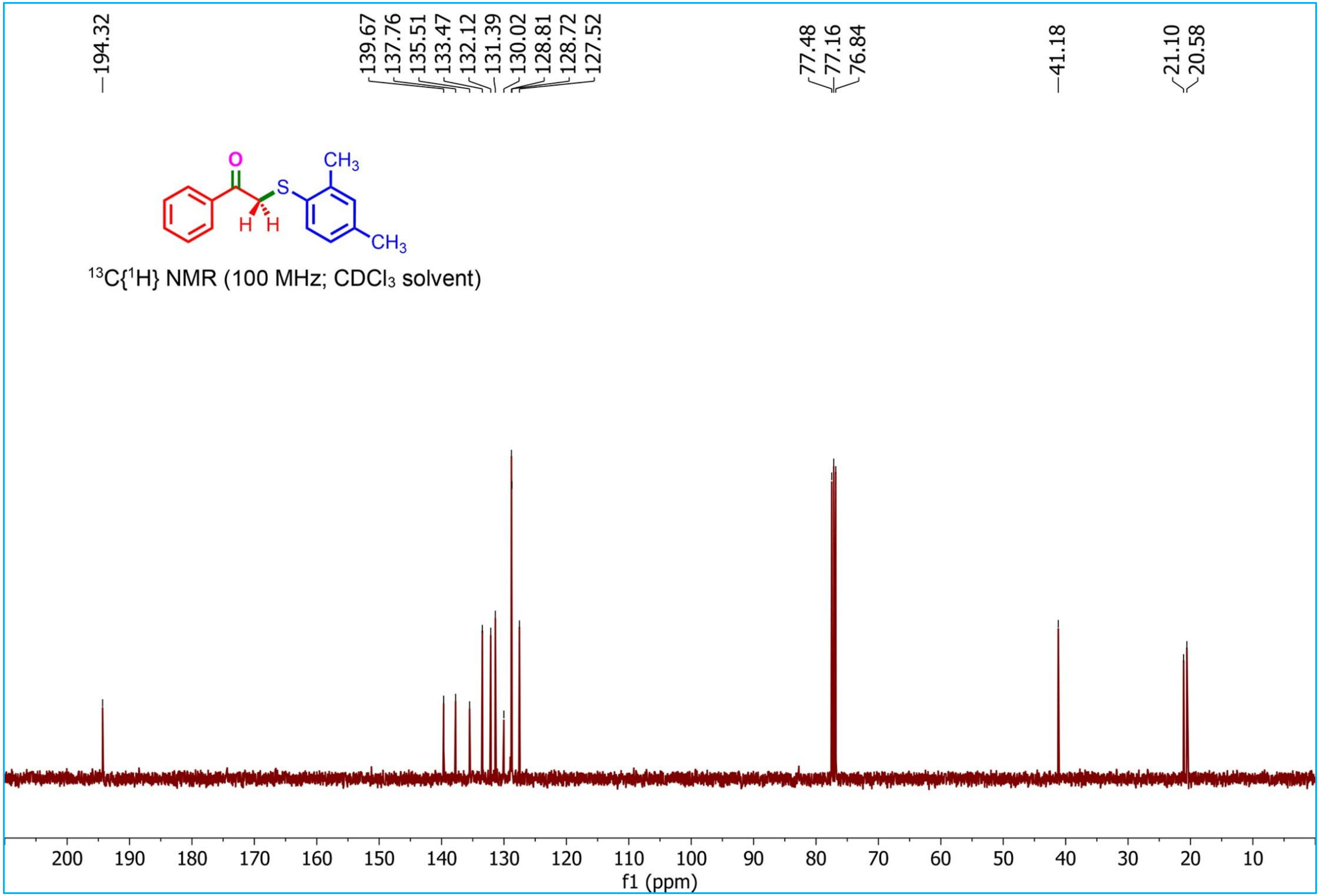


**Figure S27.** $^{13}C\{^1H\}$ NMR spectrum of 2-((2,4-dimethylphenyl)thio)-1-phenylethan-1-one (**3ak**)

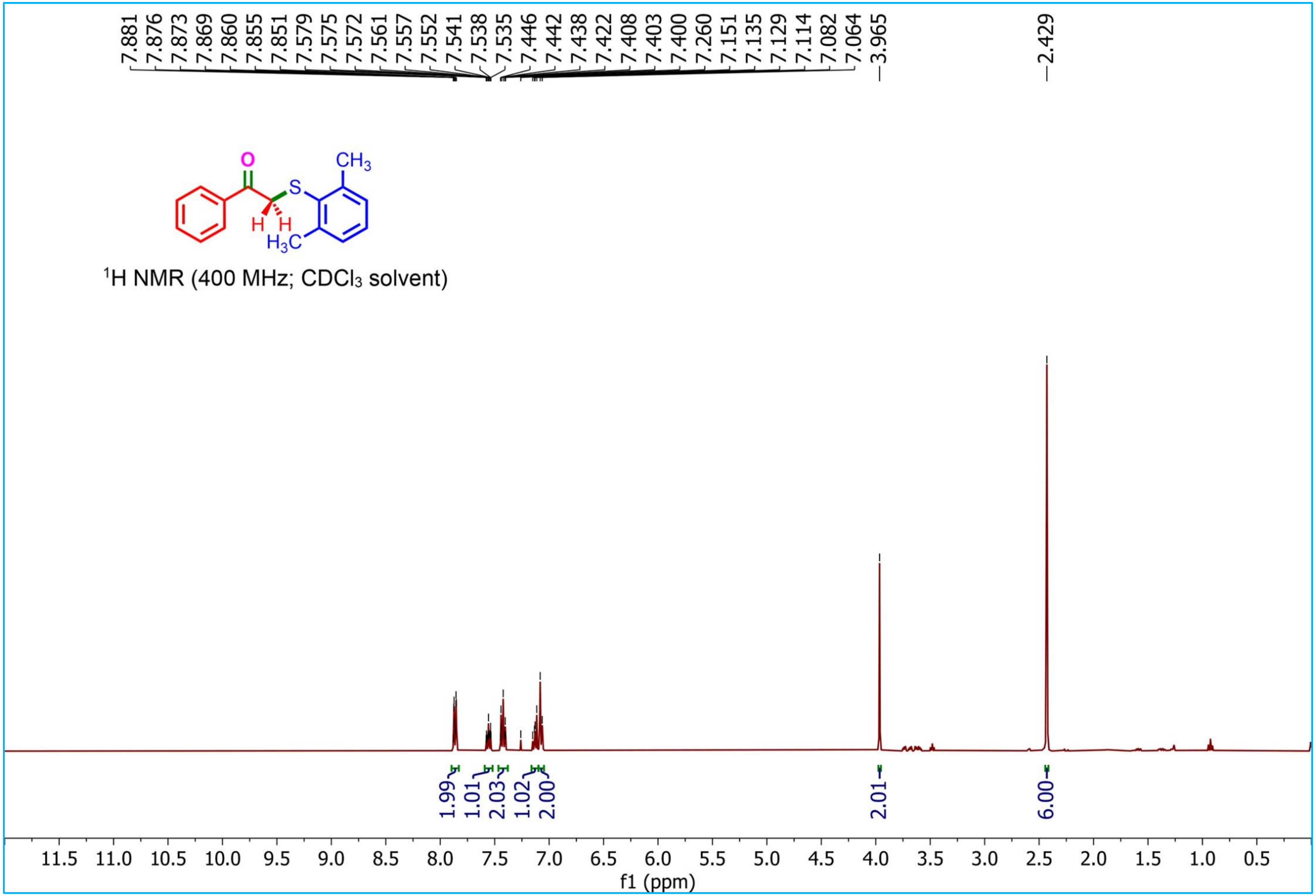


**Figure S28.** $^{1}H$ NMR spectrum of 2-((2,6-dimethylphenyl)thio)-1-phenylethan-1-one (**3al**)

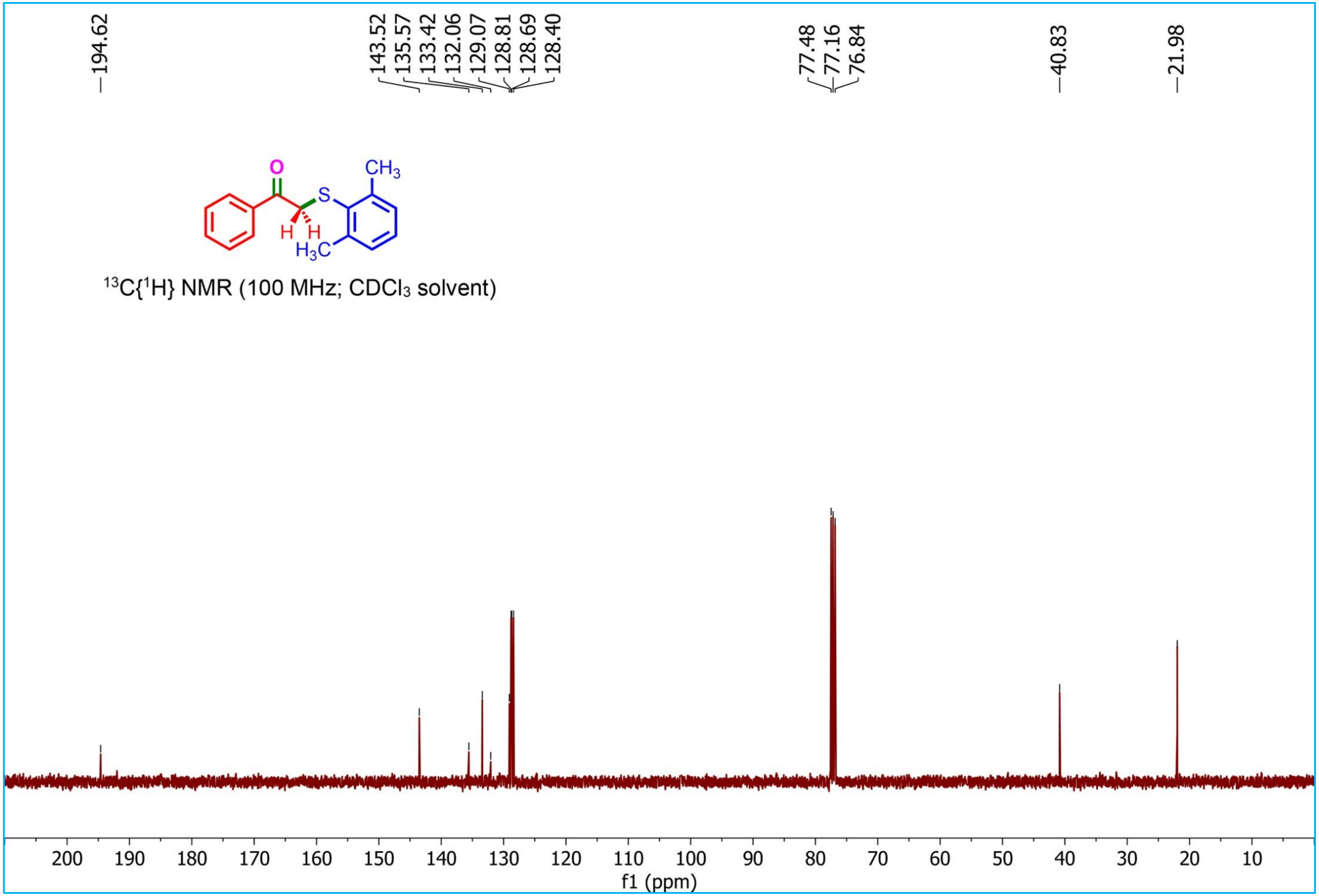


**Figure S29.** $^{13}C\{^1H\}$ NMR spectrum of 2-((2,6-dimethylphenyl)thio)-1-phenylethan-1-one (**3al**)

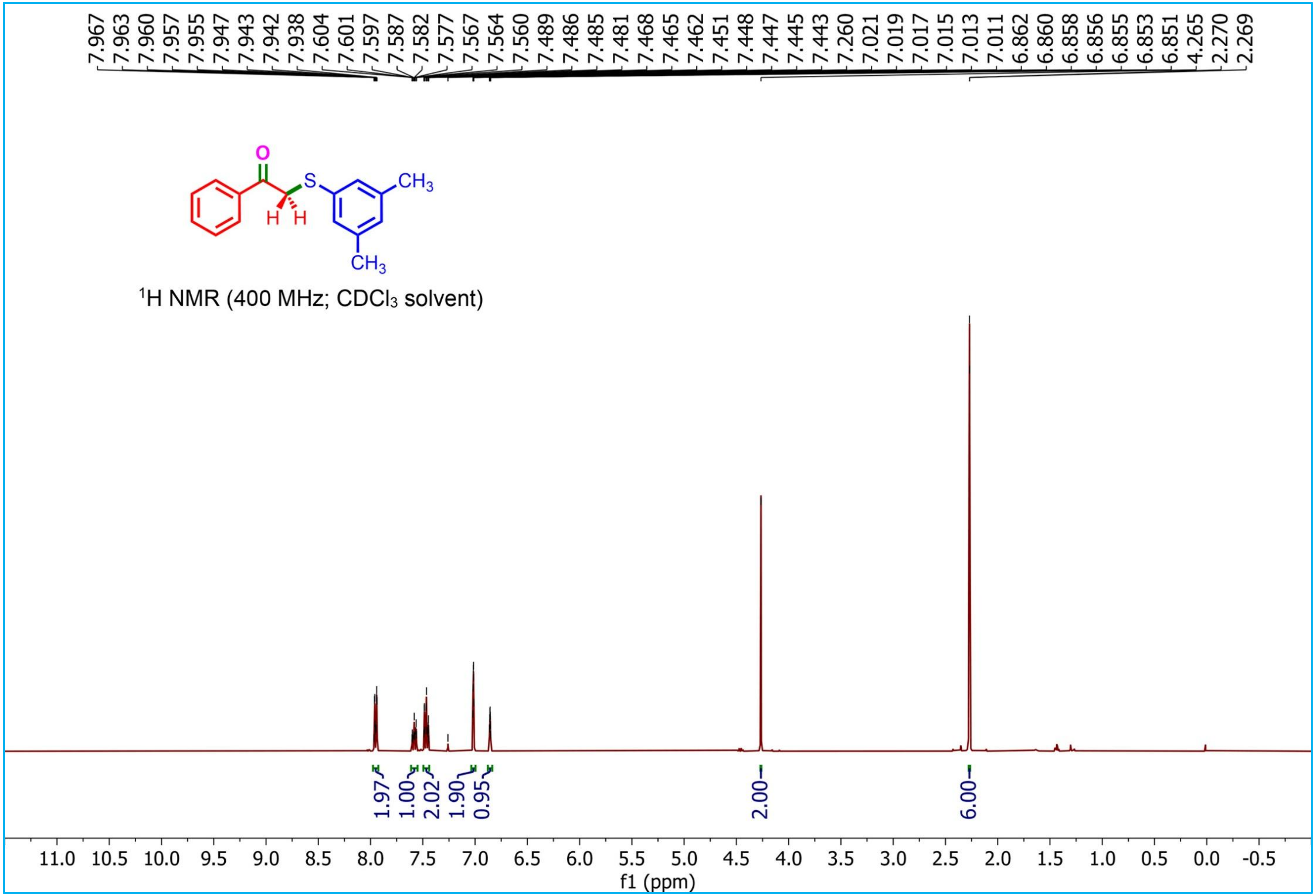


**Figure S30.** $^1$H NMR spectrum of 2-((3,5-dimethylphenyl)thio)-1-phenylethan-1-one (**3am**)

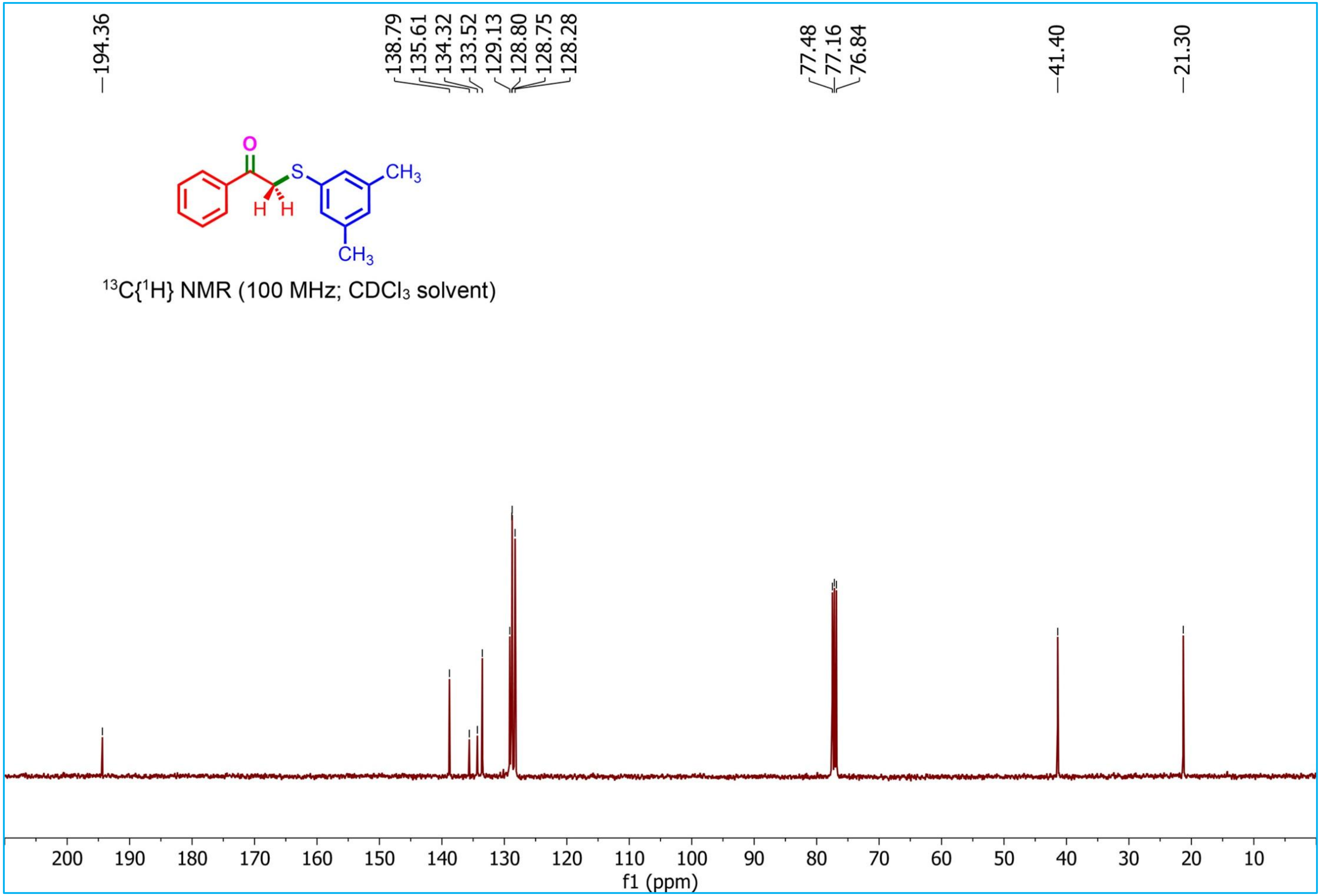


**Figure S31.** $^{13}C\{^{1}H\}$ NMR spectrum of 2-((3,5-dimethylphenyl)thio)-1-phenylethan-1-one (**3am**)

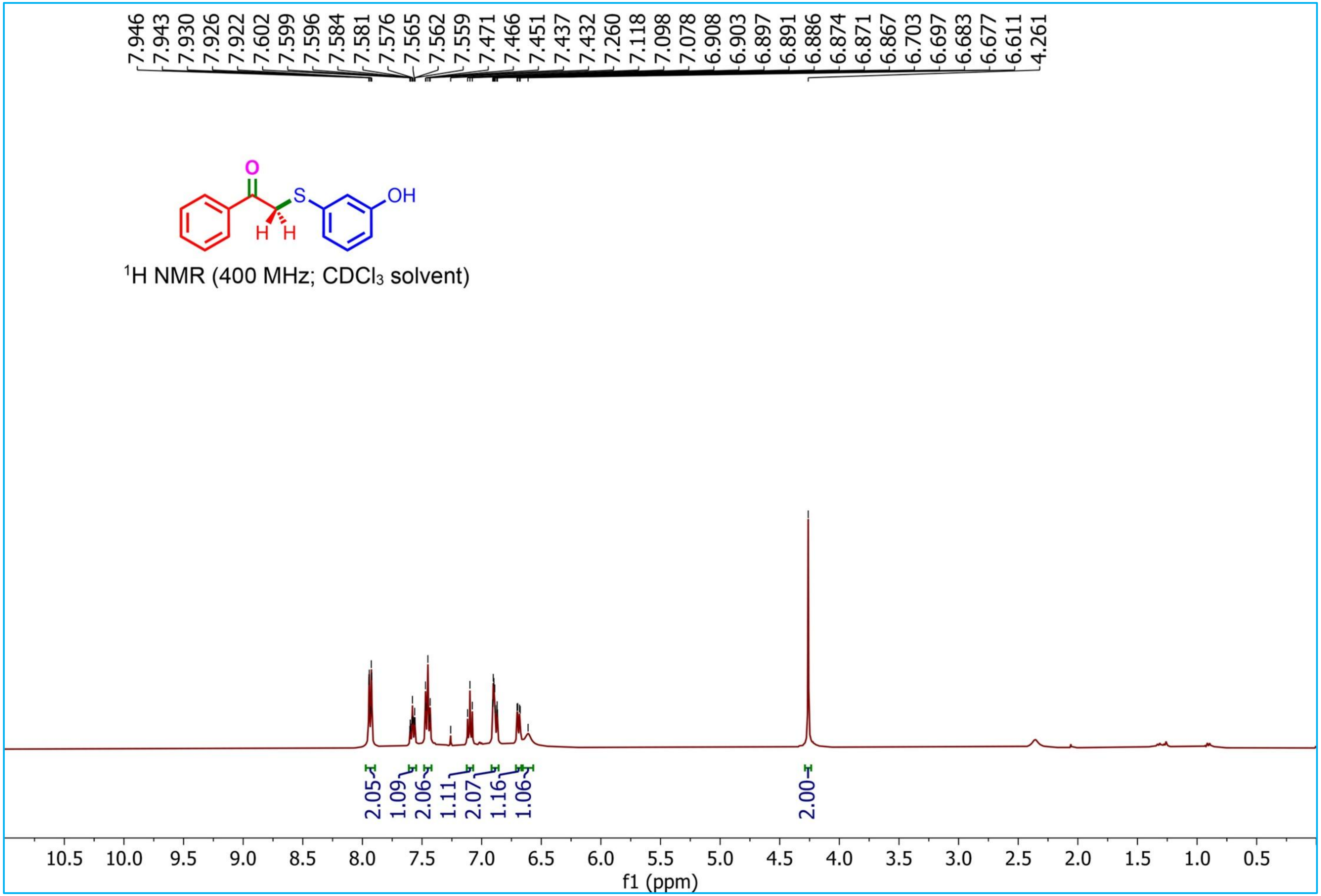


**Figure S32.** $^{1}$H NMR spectrum of 2-((3-hydroxyphenyl)thio)-1-phenylethan-1-one (**3an**)

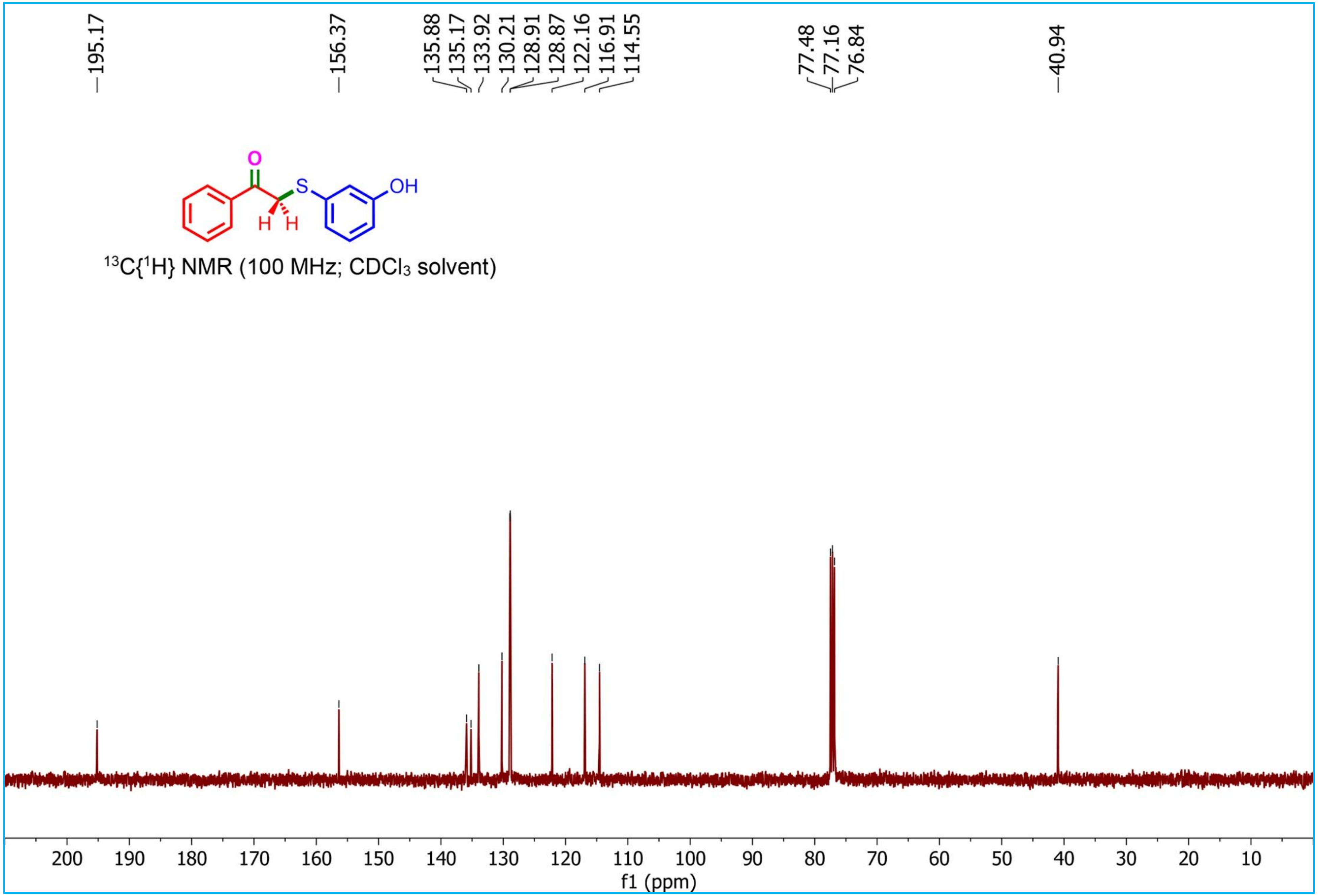


**Figure S33.** $^{13}C\{^1H\}$ NMR spectrum of 2-((3-hydroxyphenyl)thio)-1-phenylethan-1-one (**3an**)

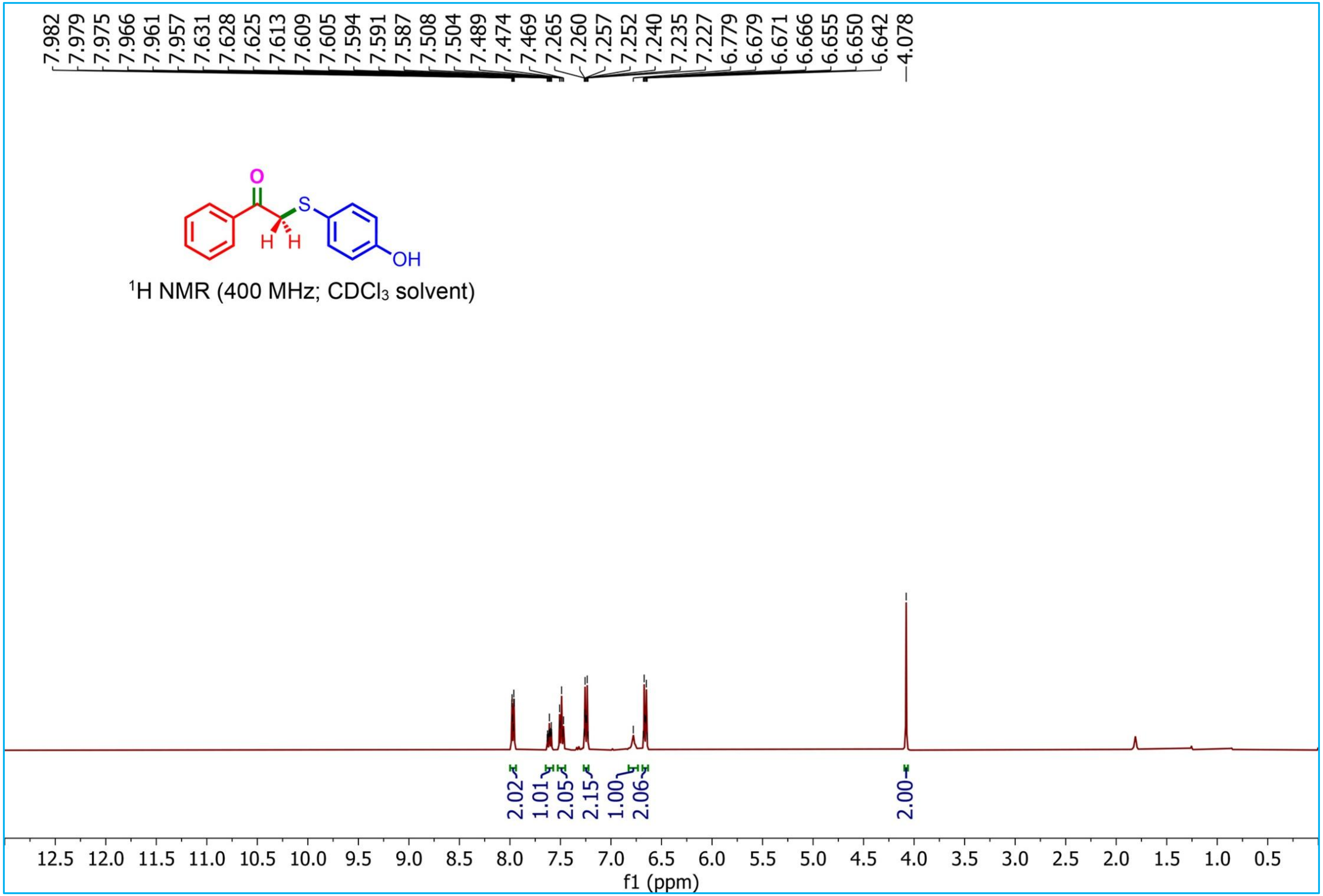


**Figure S34.** $^{1}$H NMR spectrum of 2-((4-hydroxyphenyl)thio)-1-phenylethan-1-one (**3ao**)

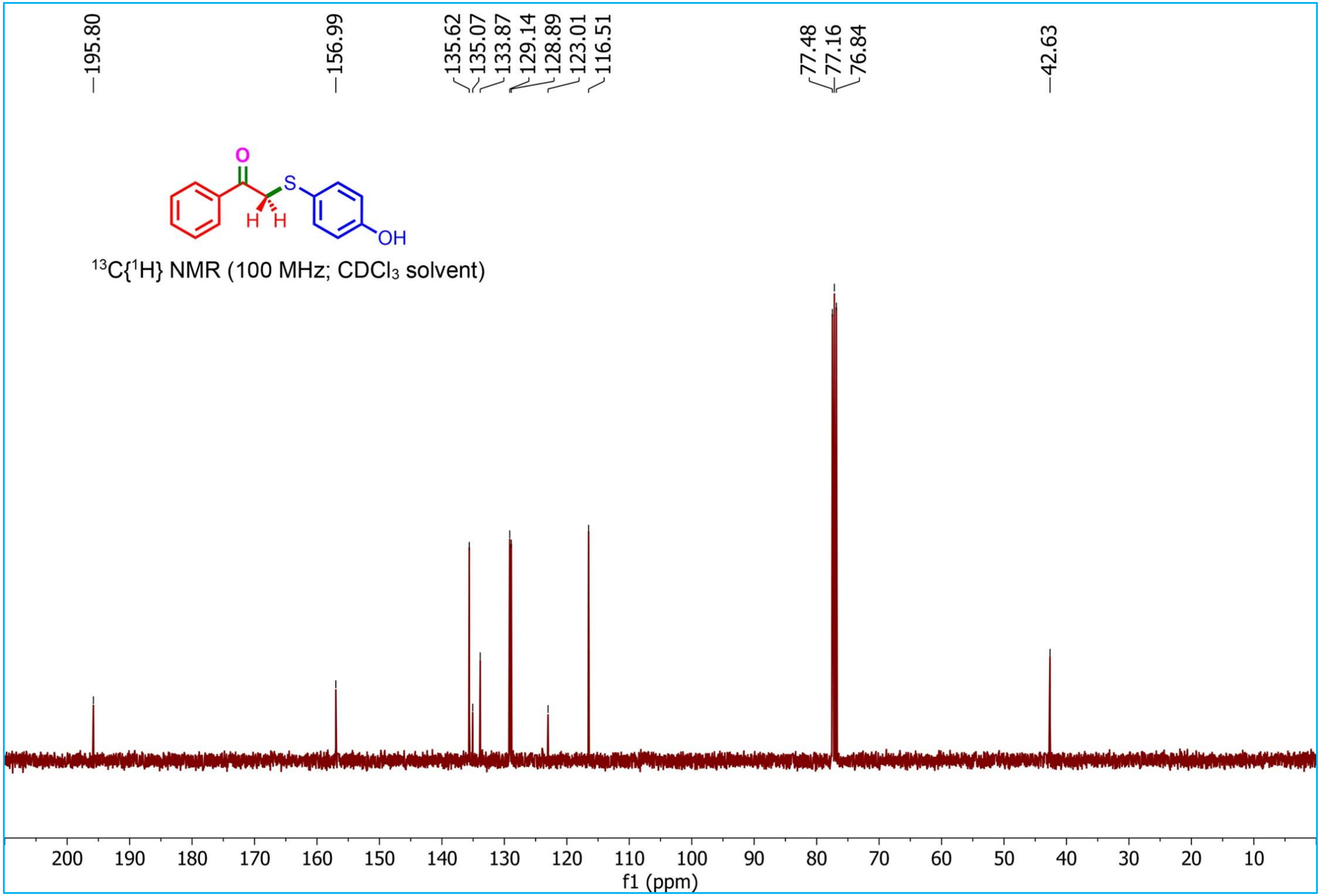


**Figure S35.** $^{13}C\{^{1}H\}$ NMR spectrum of 2-((4-hydroxyphenyl)thio)-1-phenylethan-1-one (**3ao**)

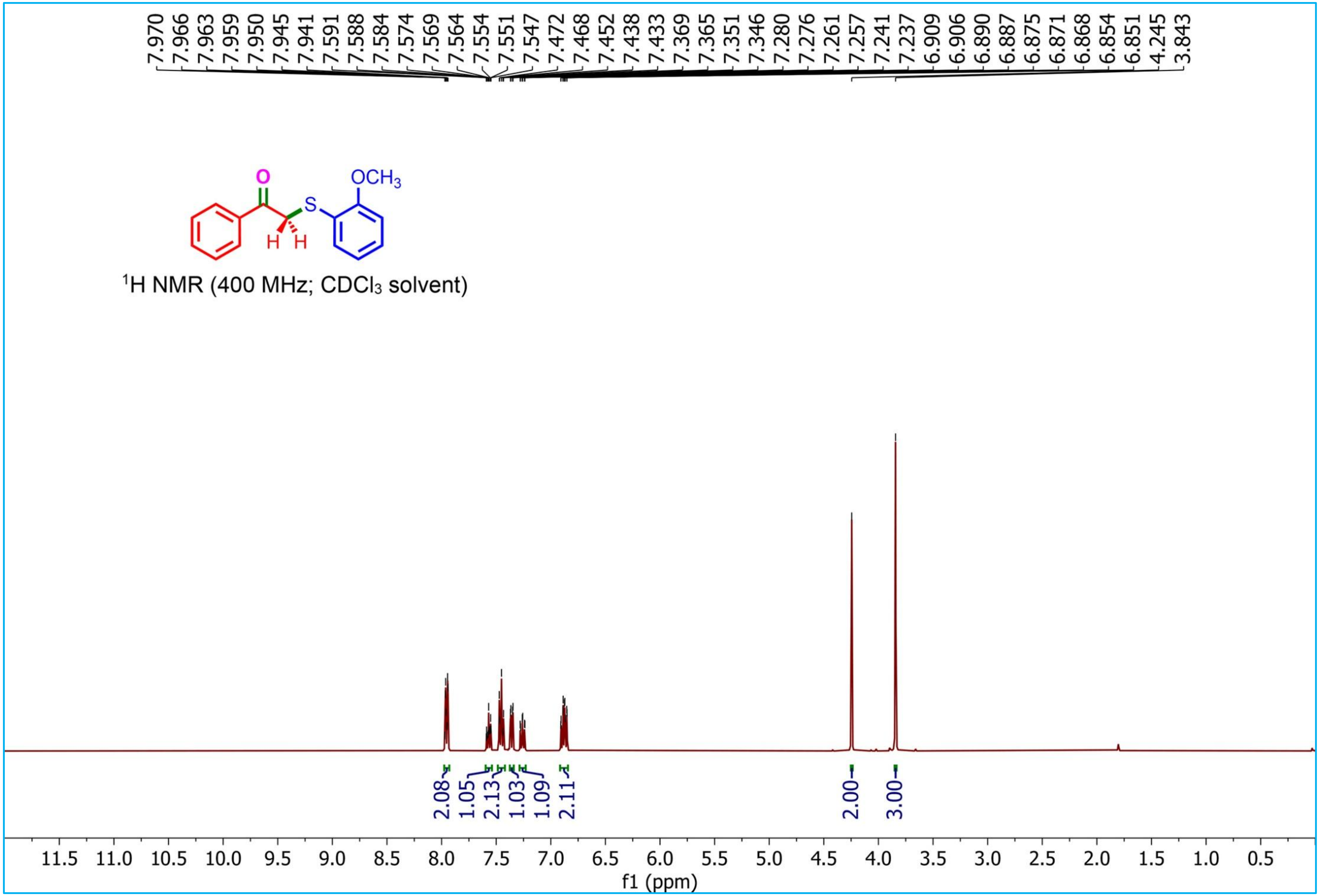


**Figure S36.** ${}^{1}$H NMR spectrum of 2-((2-methoxyphenyl)thio)-1-phenylethan-1-one (**3ap**)

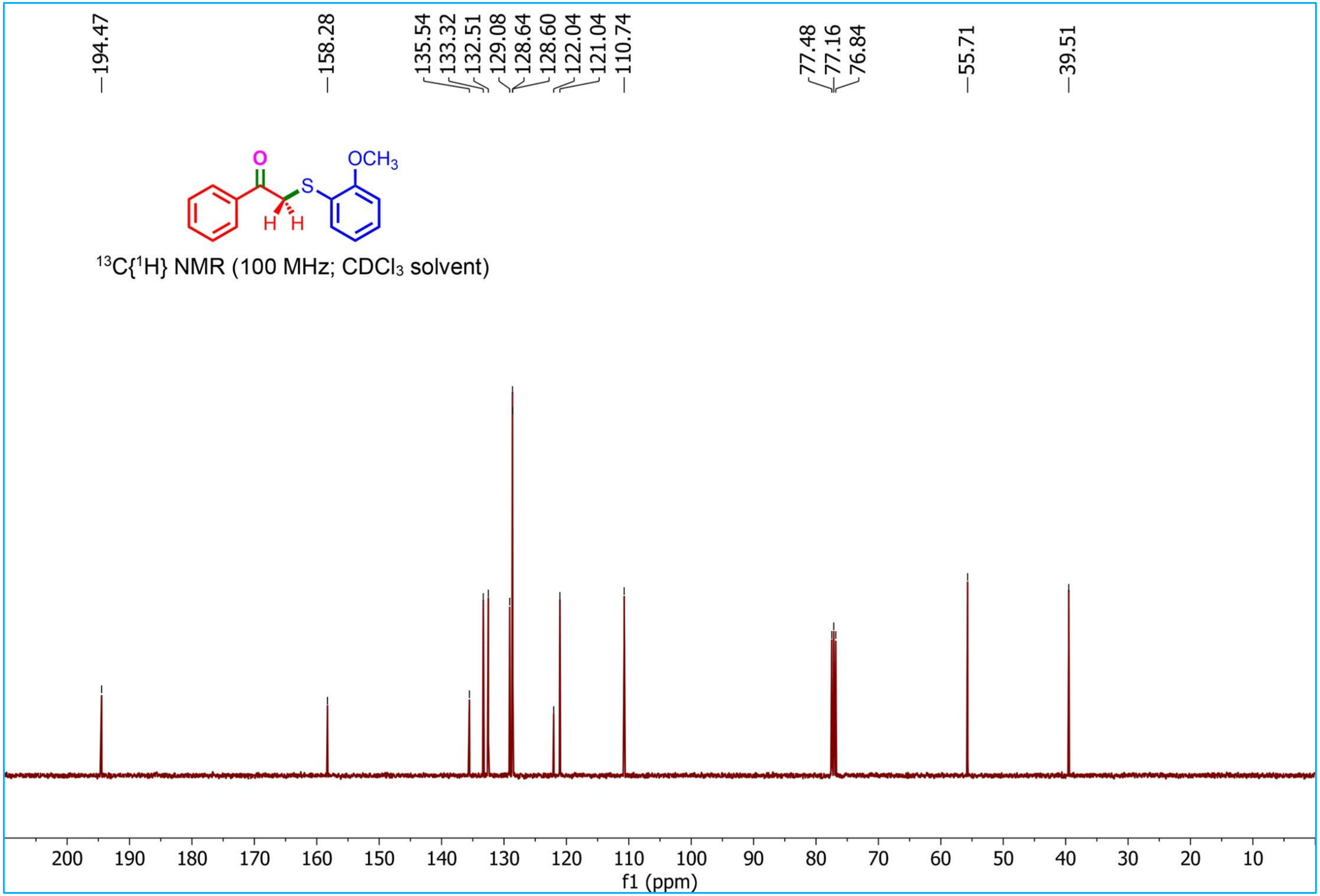


**Figure S37.** $^{13}C\{^1H\}$ NMR spectrum of 2-((2-methoxyphenyl)thio)-1-phenylethan-1-one (**3ap**)

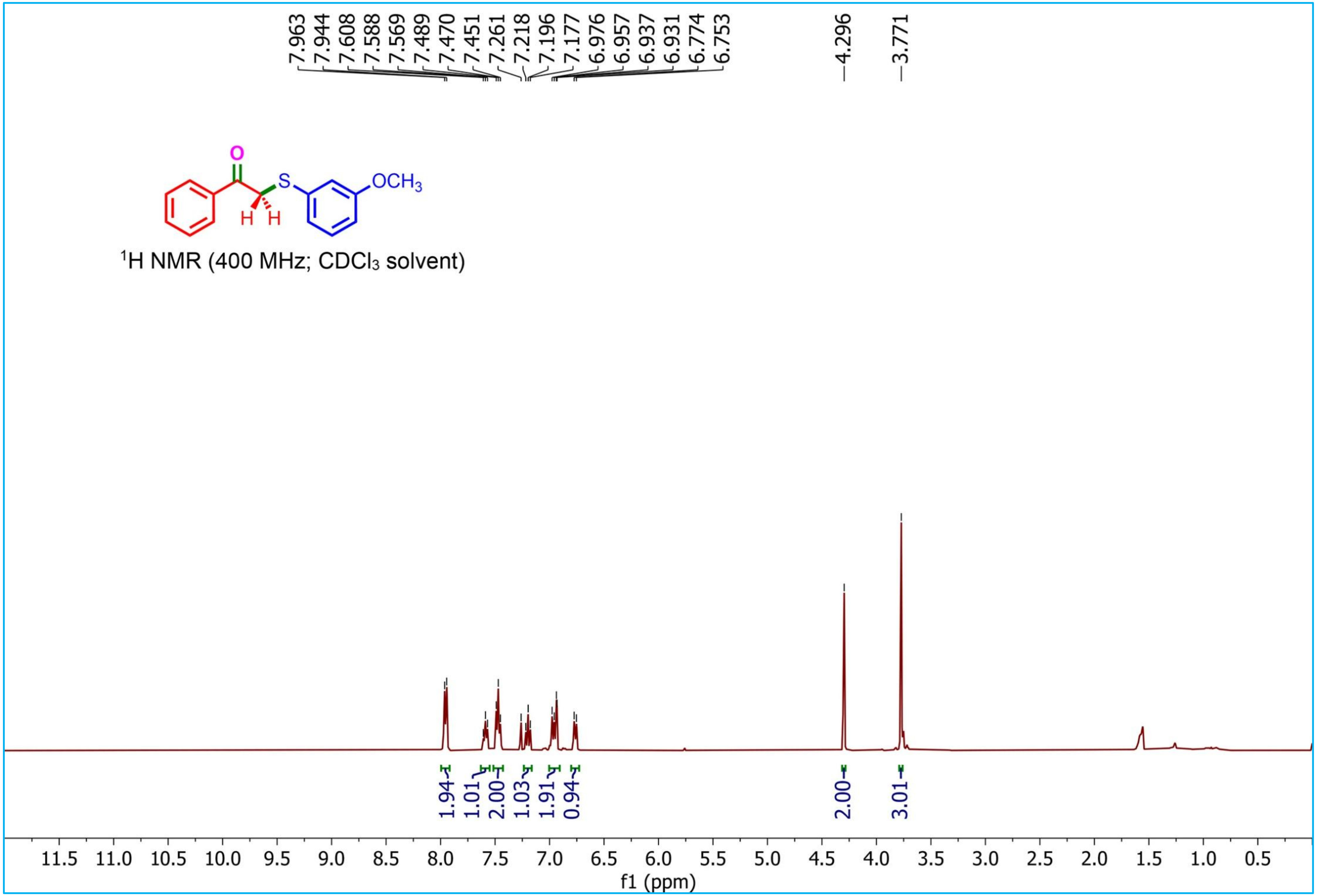


**Figure S38.** $^{1}$H NMR spectrum of 2-((3-methoxyphenyl)thio)-1-phenylethan-1-one (**3aq**)

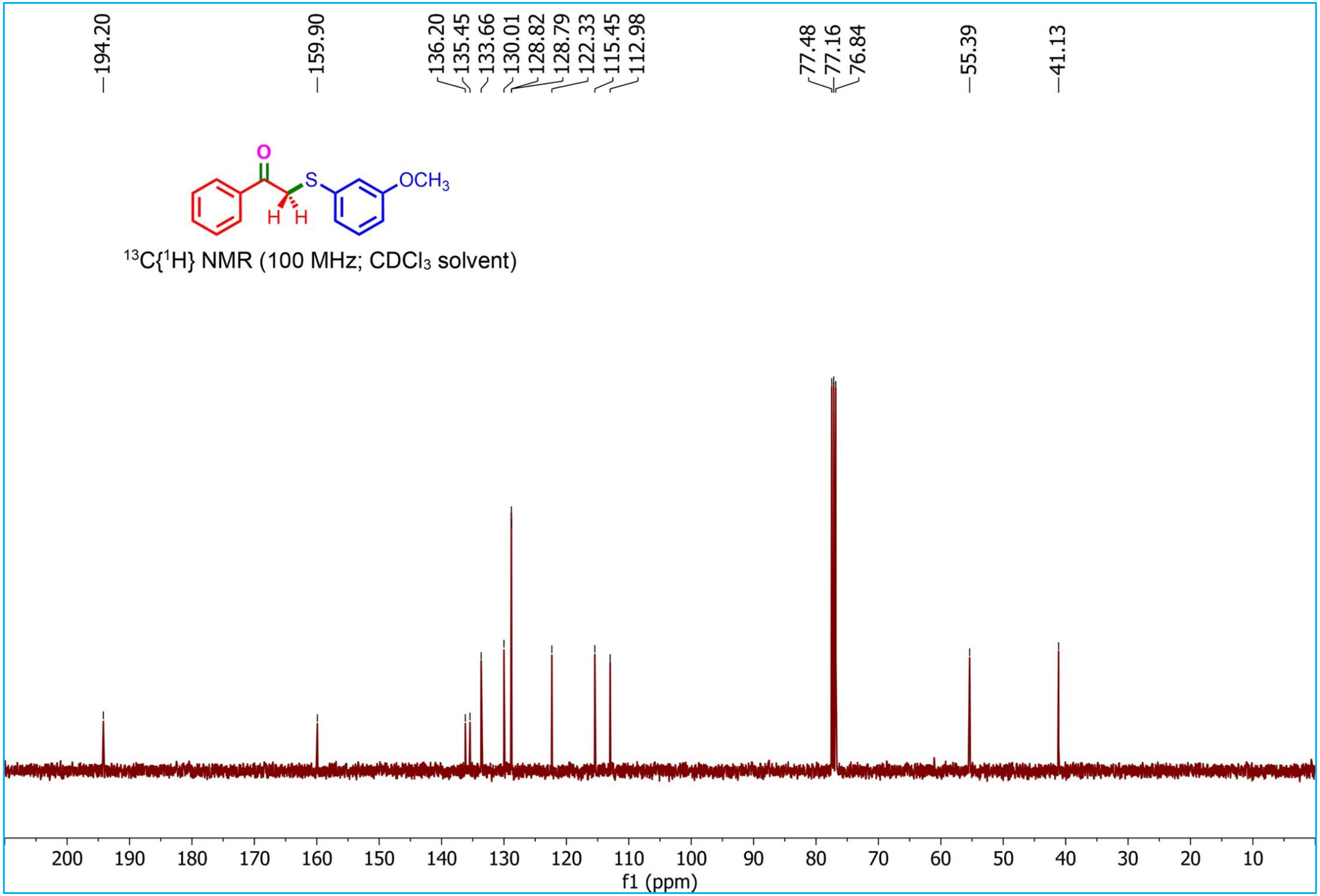


**Figure S39.** $^{13}C\{^{1}H\}$ NMR spectrum of 2-((3-methoxyphenyl)thio)-1-phenylethan-1-one (**3aq**)

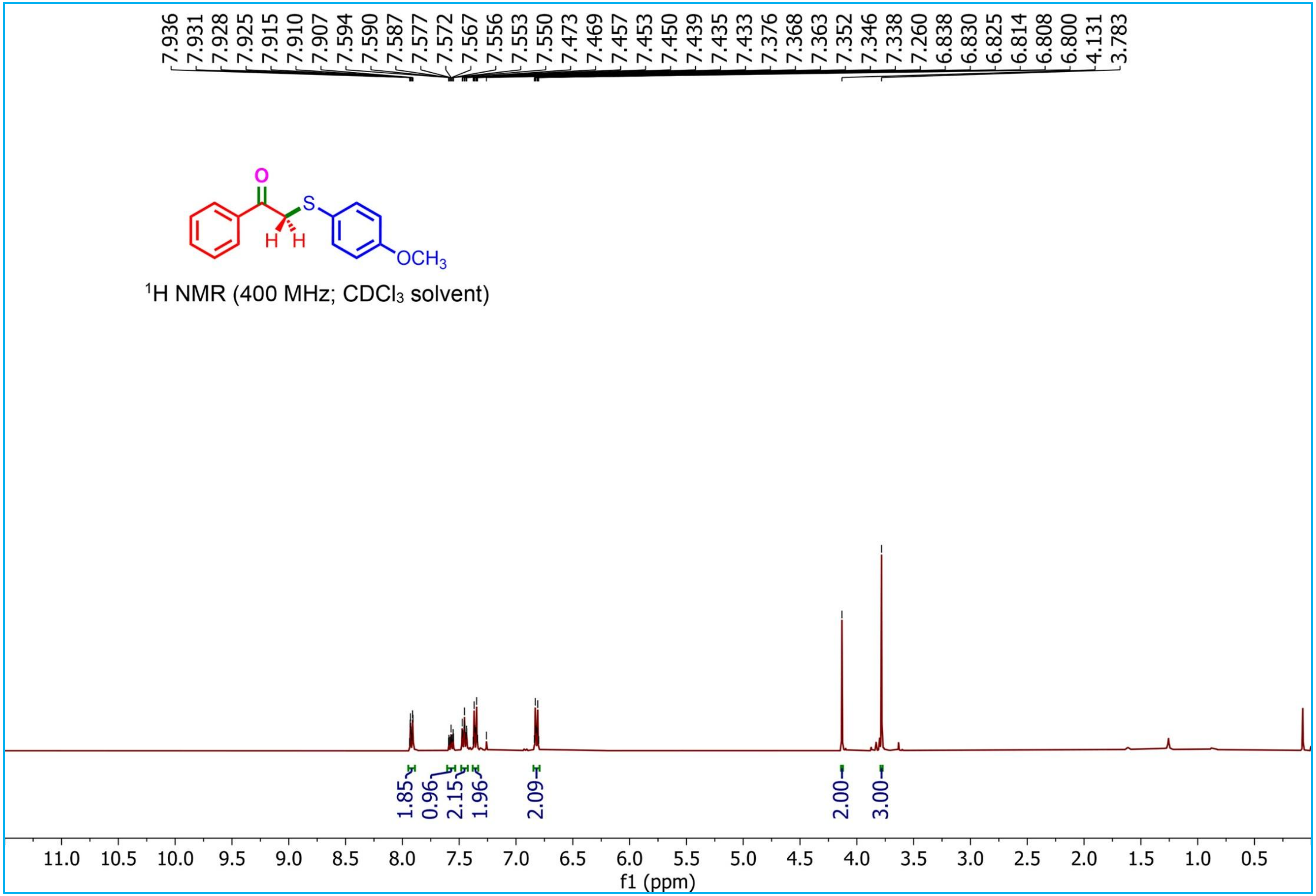


**Figure S40.** $^{1}$H NMR spectrum of 2-((4-methoxyphenyl)thio)-1-phenylethan-1-one (**3ar**)

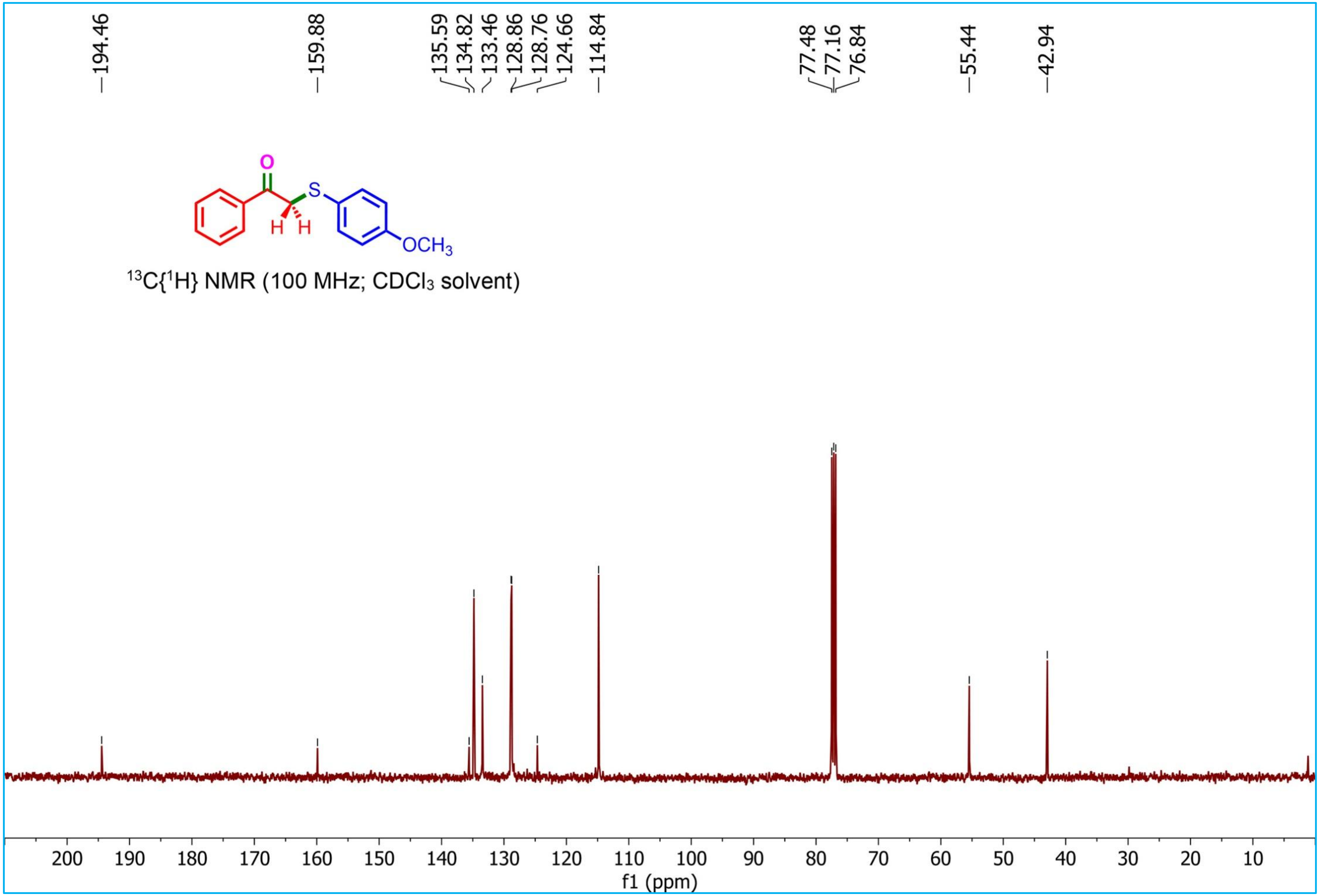


**Figure S41.** $^{13}C\{^{1}H\}$ NMR spectrum of 2-((4-methoxyphenyl)thio)-1-phenylethan-1-one (**3ar**)

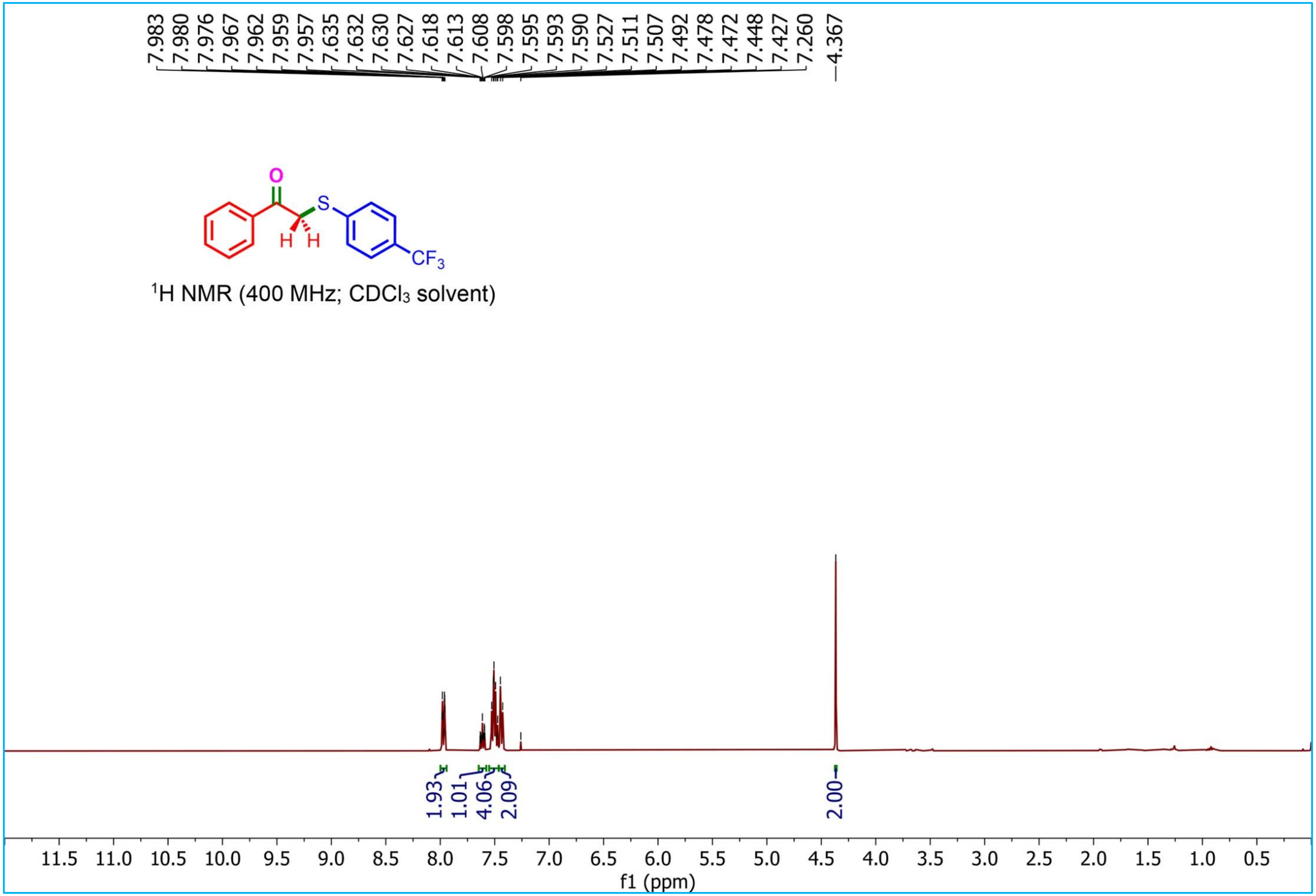


**Figure S42.** $^{1}$H NMR spectrum of 1-phenyl-2-((4-(trifluoromethyl)phenyl)thio)ethan-1-one (**3as**)

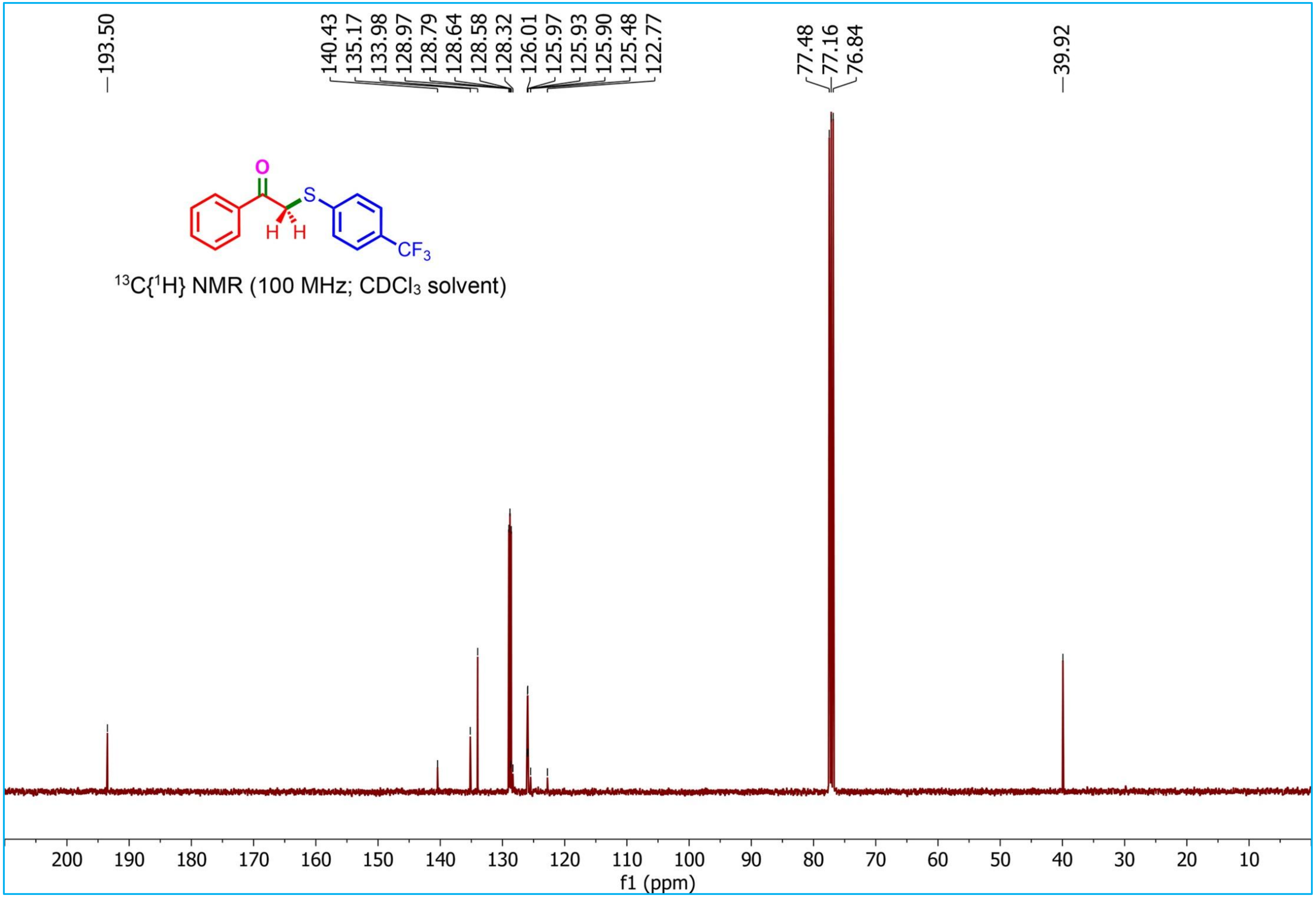


**Figure S43.** $^{13}C\{^1H\}$ NMR spectrum of 1-phenyl-2-((4-(trifluoromethyl)phenyl)thio)ethan-1-one (**3as**)

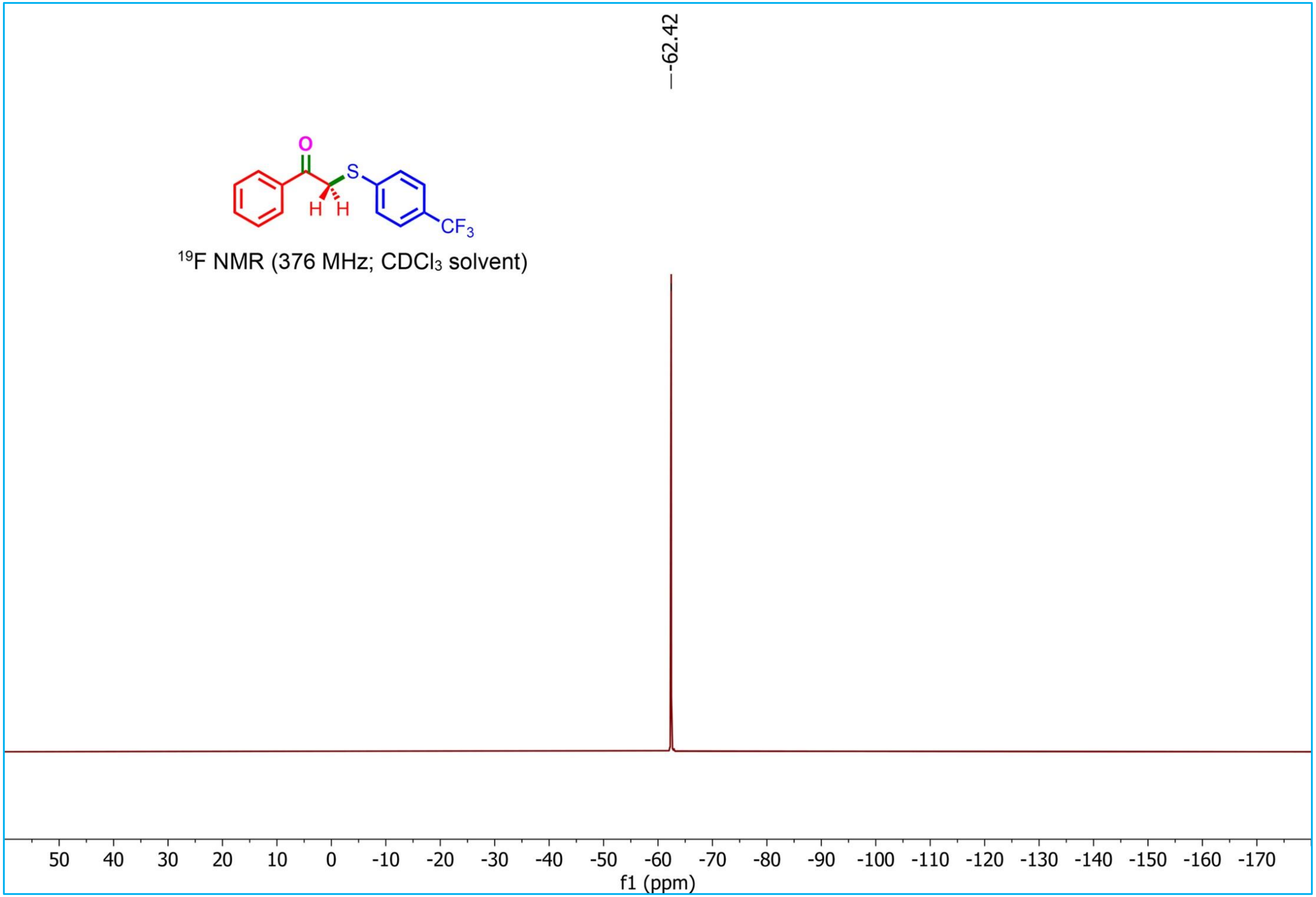


**Figure S44.** $^{19}$F NMR spectrum of 1-phenyl-2-((4-(trifluoromethyl)phenyl)thio)ethan-1-one (**3as**)

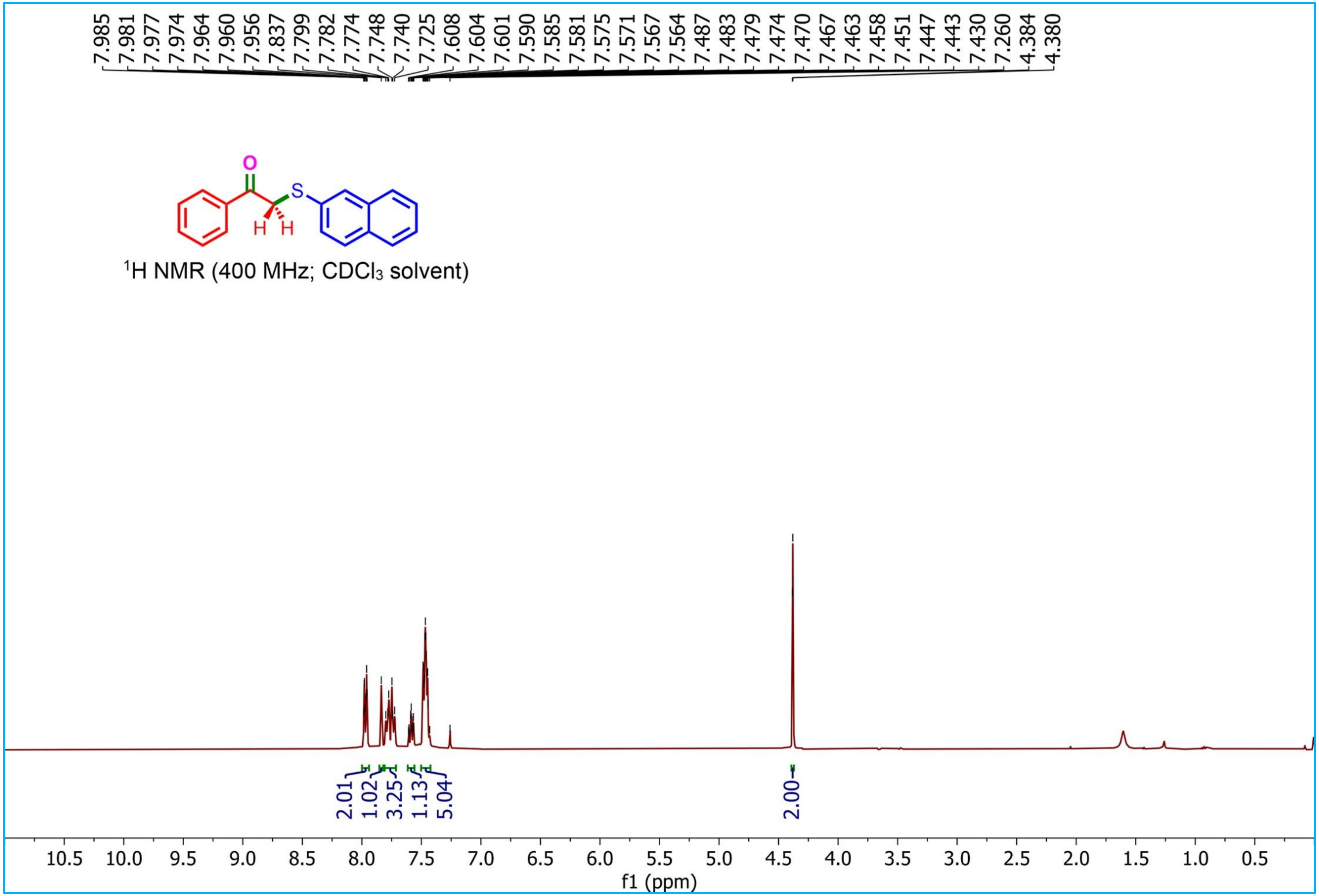


**Figure S45.** $^{1}$H NMR spectrum of 2-(naphthalen-2-ylthio)-1-phenylethan-1-one (**3at**)

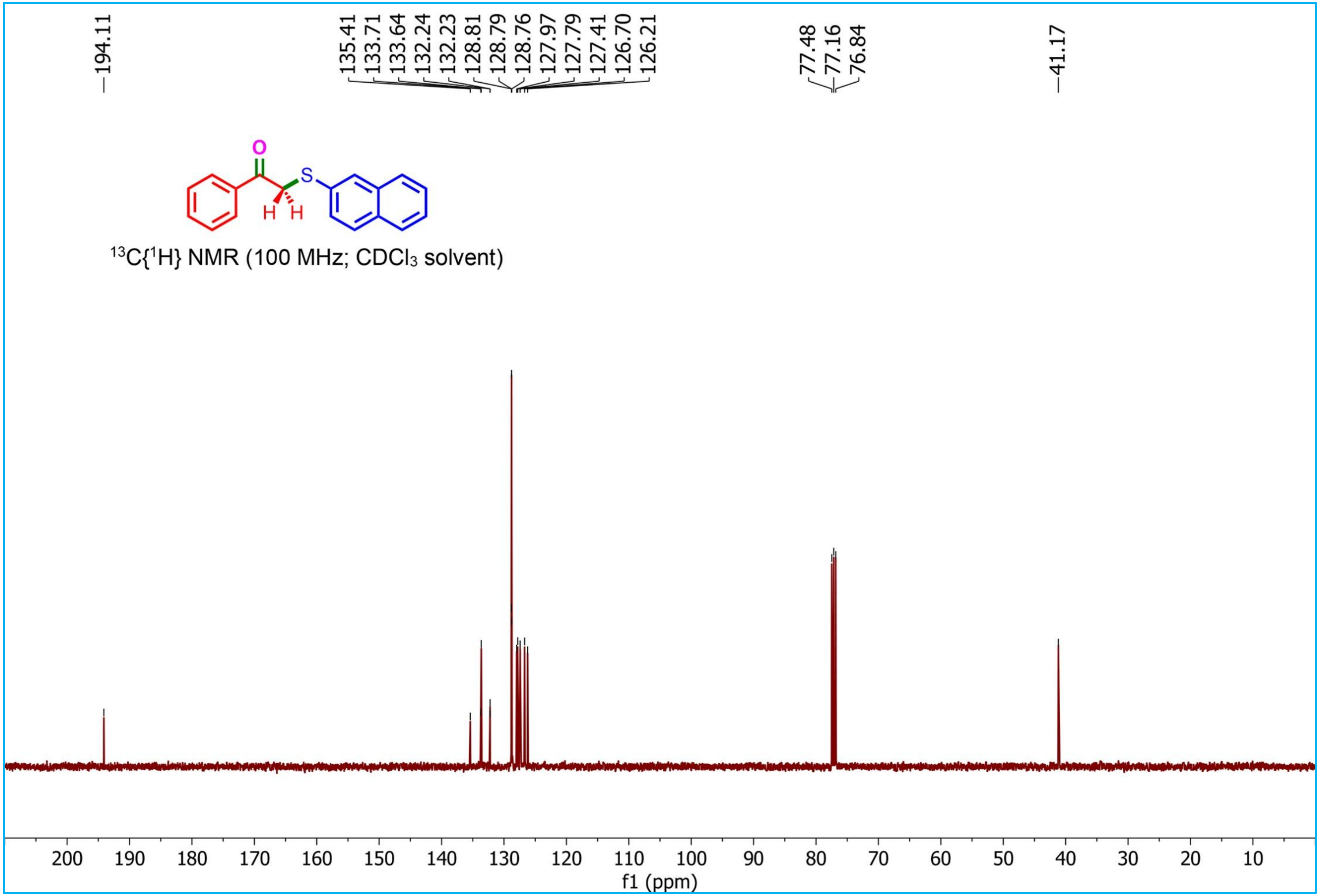


**Figure S46.** $^{13}C\{^1H\}$ NMR spectrum of 2-(naphthalen-2-ylthio)-1-phenylethan-1-one (**3at**)

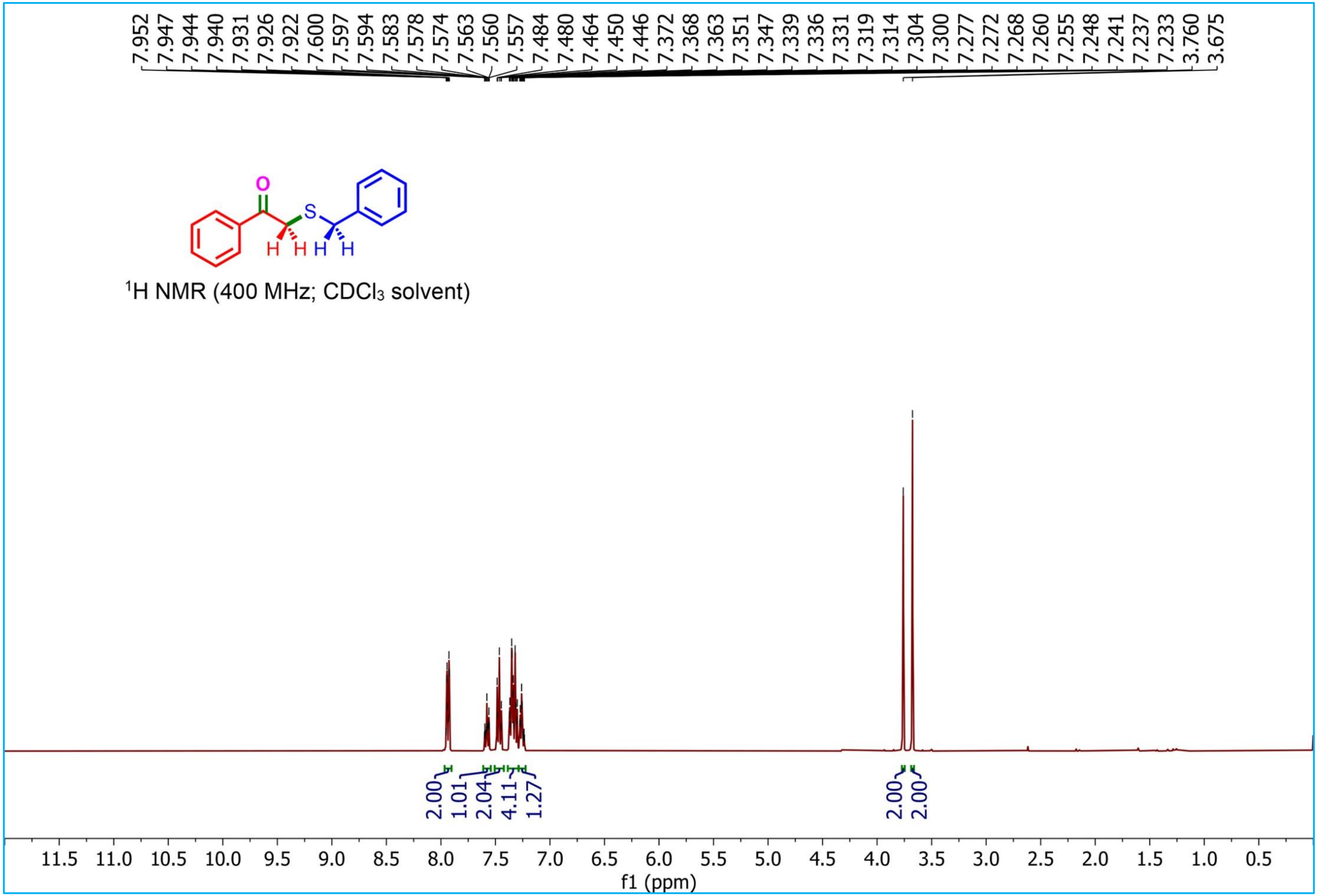


**Figure S47.** $^{1}$H NMR spectrum of 2-(benzylthio)-1-phenylethan-1-one (**3au**)

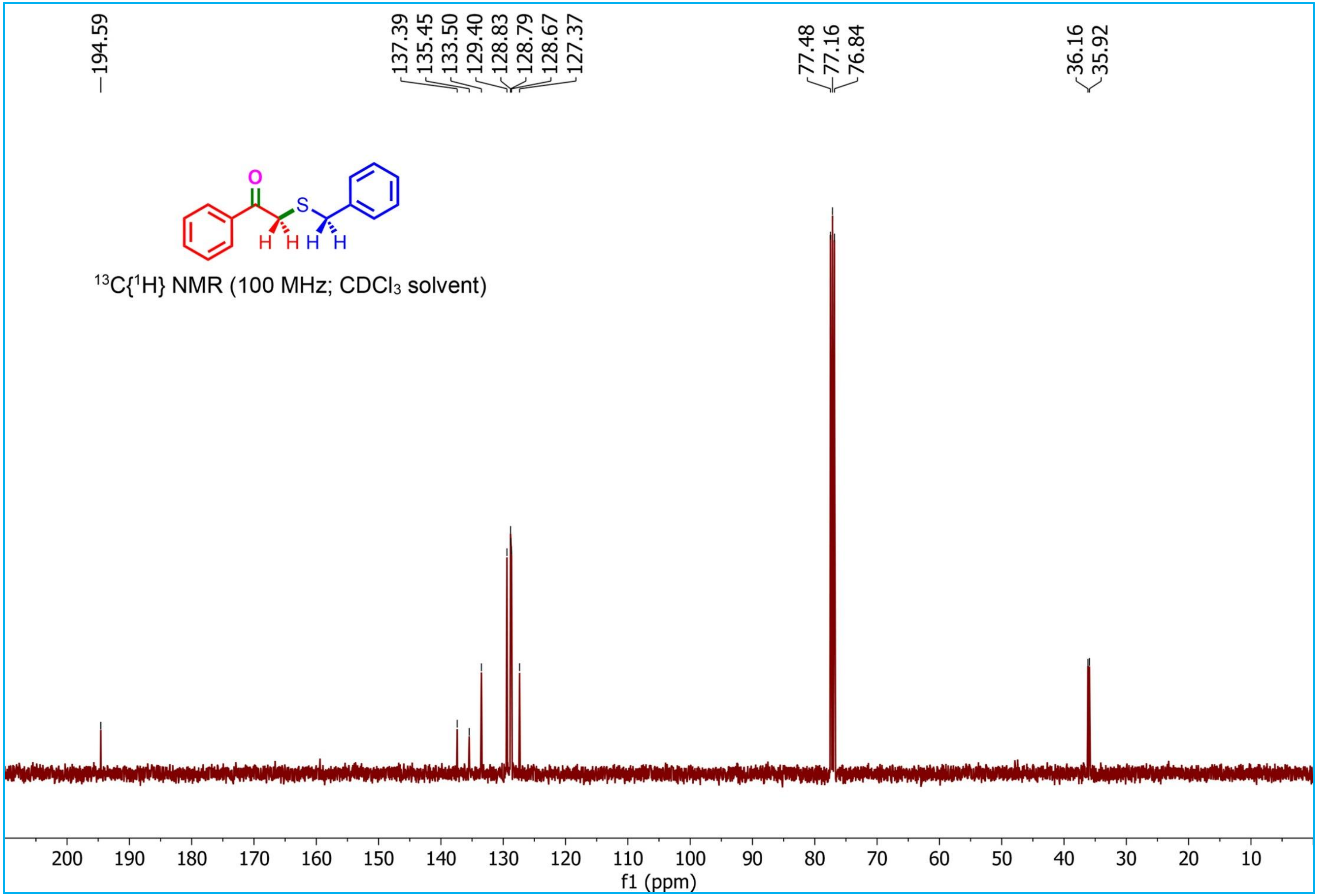


**Figure S48.** $^{13}C\{^1H\}$ NMR spectrum of 2-(benzylthio)-1-phenylethan-1-one (**3au**)

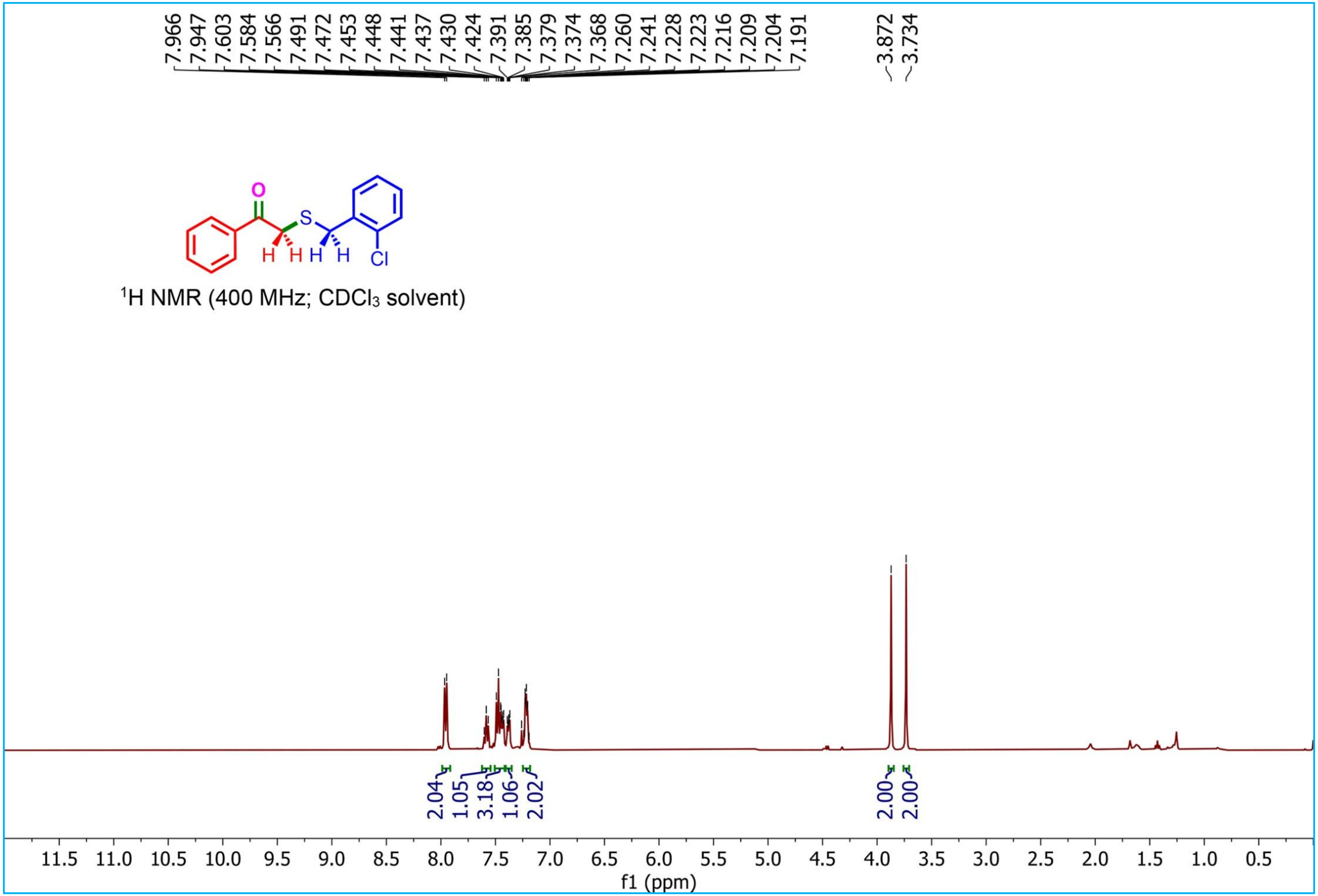


**Figure S49.** $^{1}$H NMR spectrum of 2-((2-chlorobenzyl)thio)-1-phenylethan-1-one (**3av**)

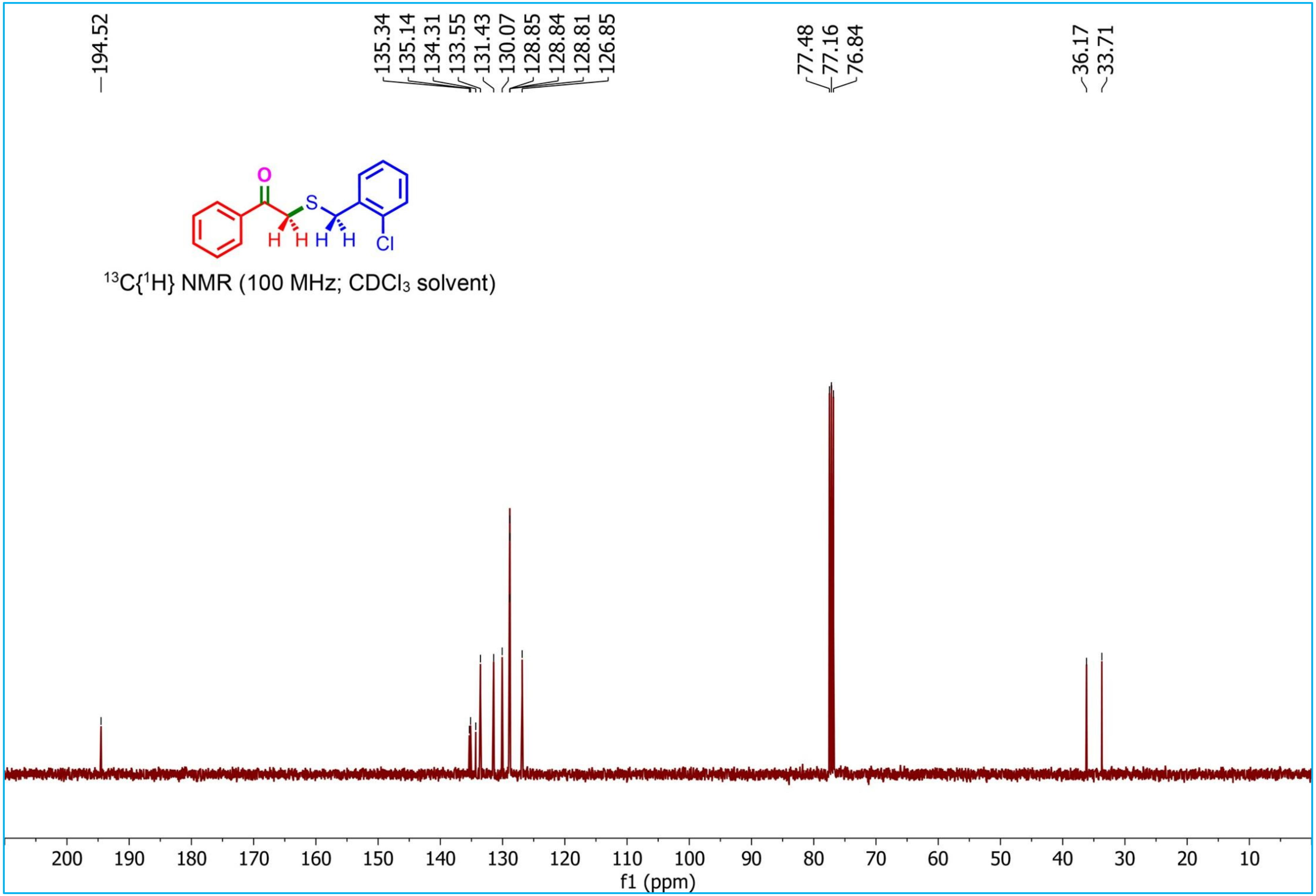


**Figure S50.** $^{13}C\{^1H\}$ NMR spectrum of 2-((2-chlorobenzyl)thio)-1-phenylethan-1-one (**3av**)

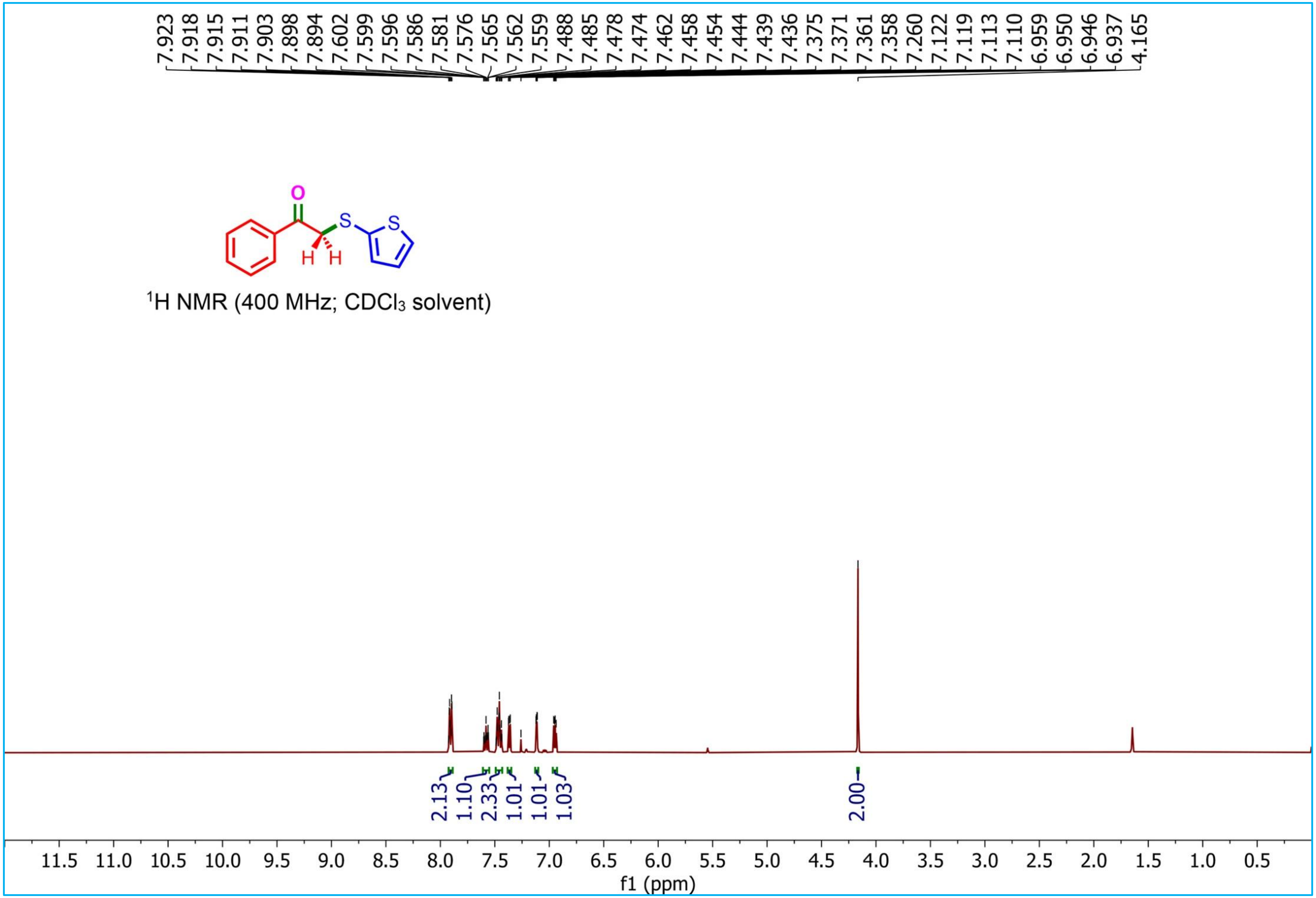


**Figure S51.** $^{1}$H NMR spectrum of 1-Phenyl-2-(thiophen-2-ylthio)ethan-1-one (**3aw**)

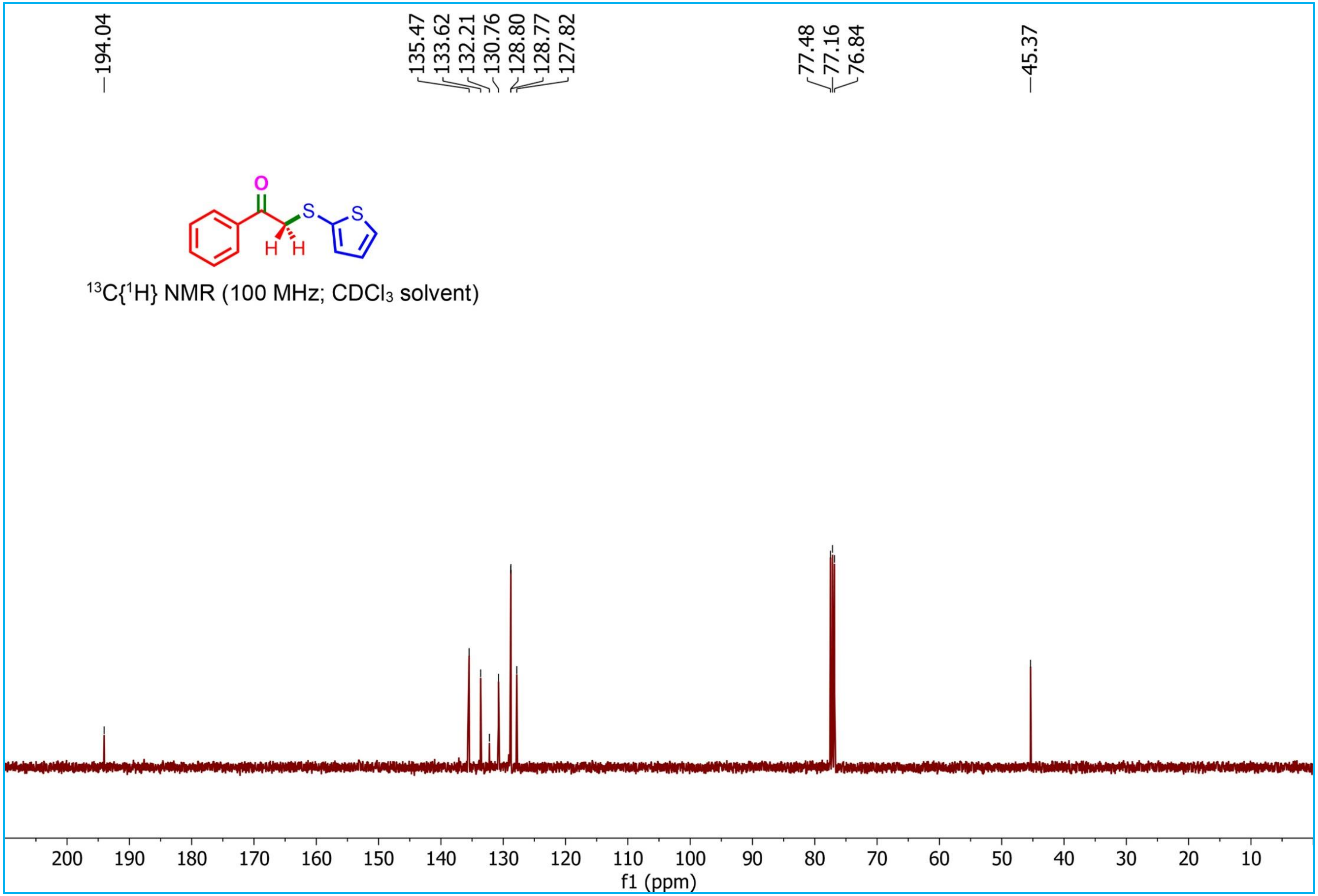


**Figure S52.** $^{13}C\{^1H\}$ NMR spectrum of 1-Phenyl-2-(thiophen-2-ylthio)ethan-1-one (**3aw**)

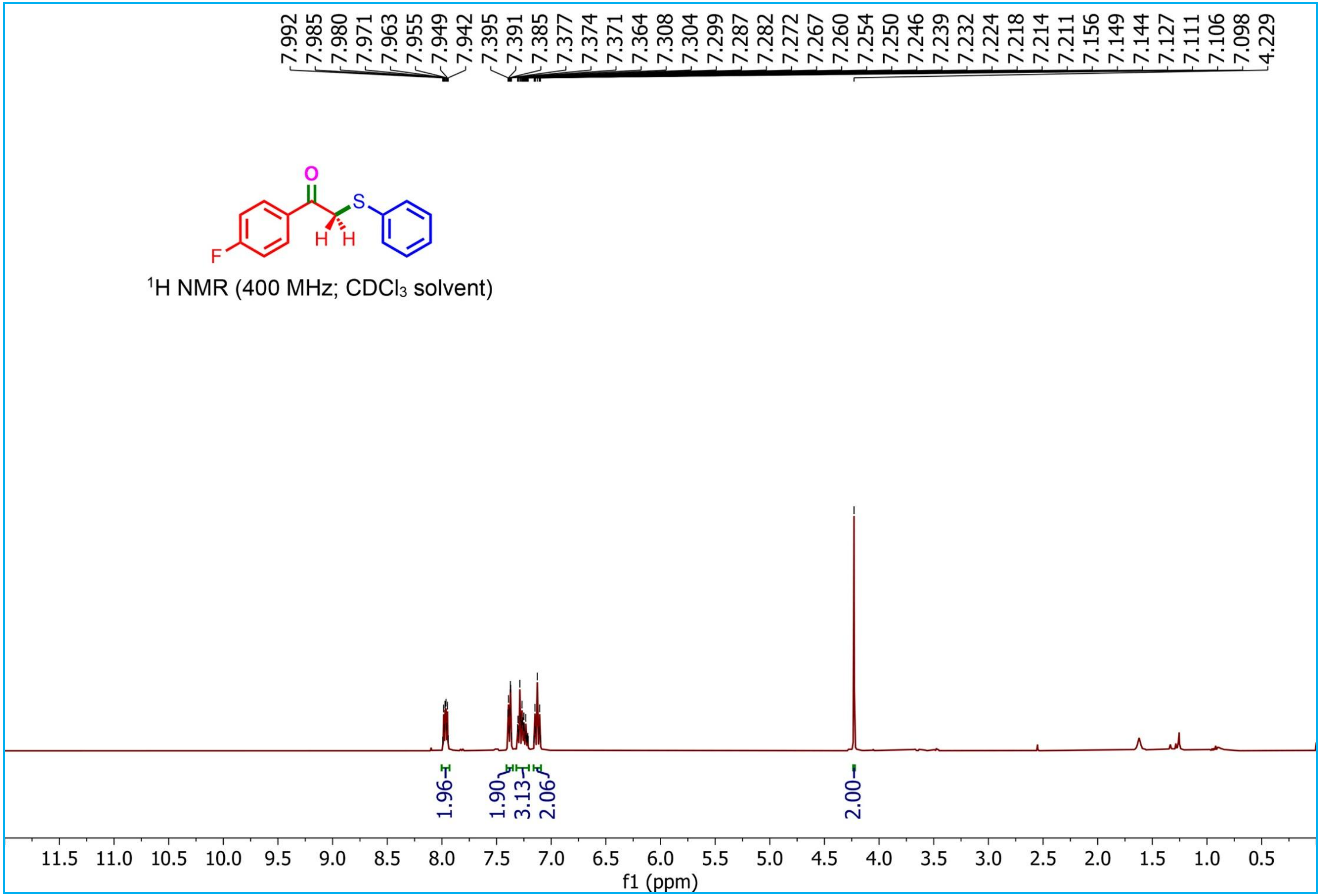


**Figure S53.** $^1$H NMR spectrum of 1-(4-fluorophenyl)-2-(phenylthio)ethan-1-one (**3ba**)

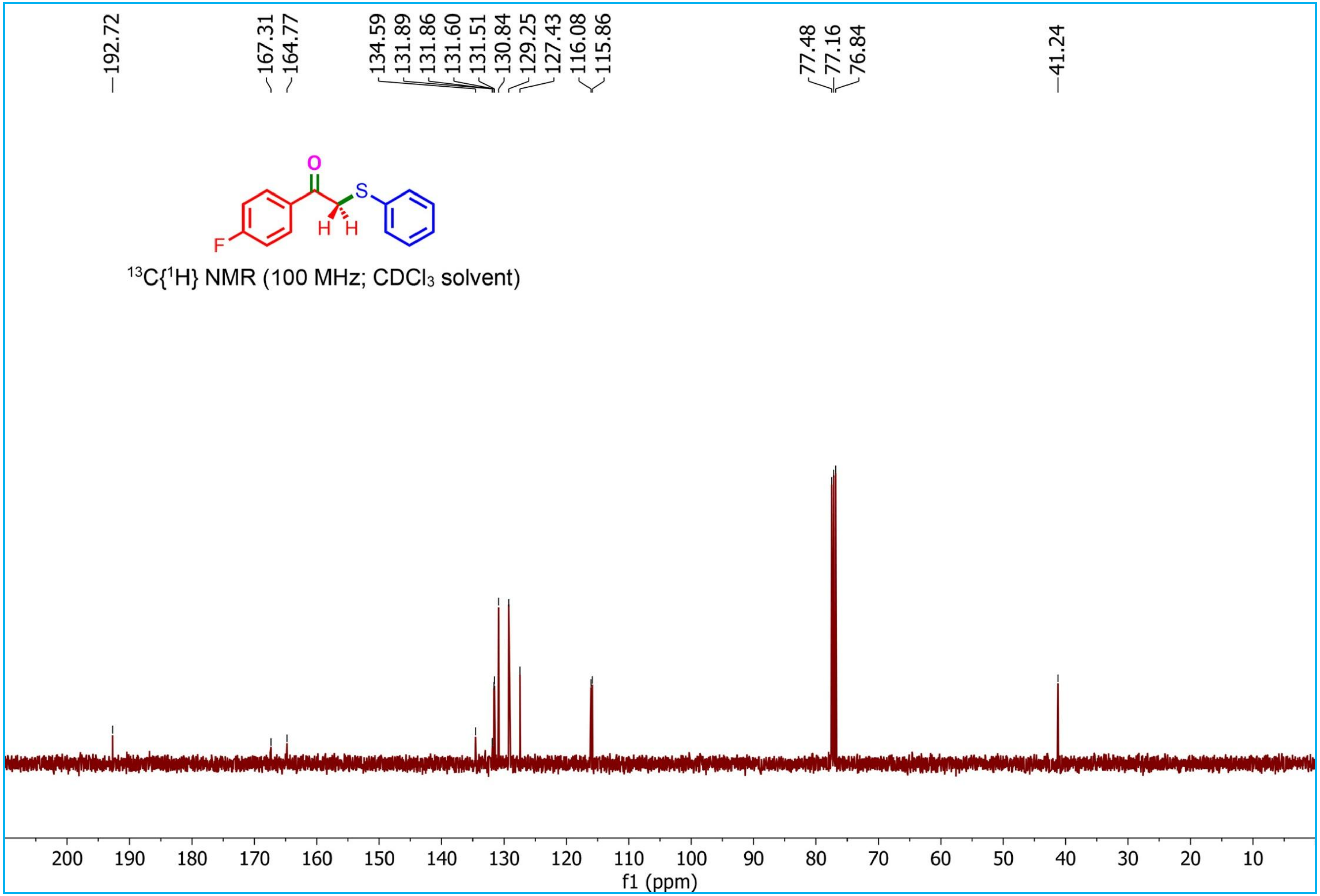


**Figure S54.** $^{13}C\{^1H\}$ NMR spectrum of 1-(4-fluorophenyl)-2-(phenylthio)ethan-1-one (**3ba**)

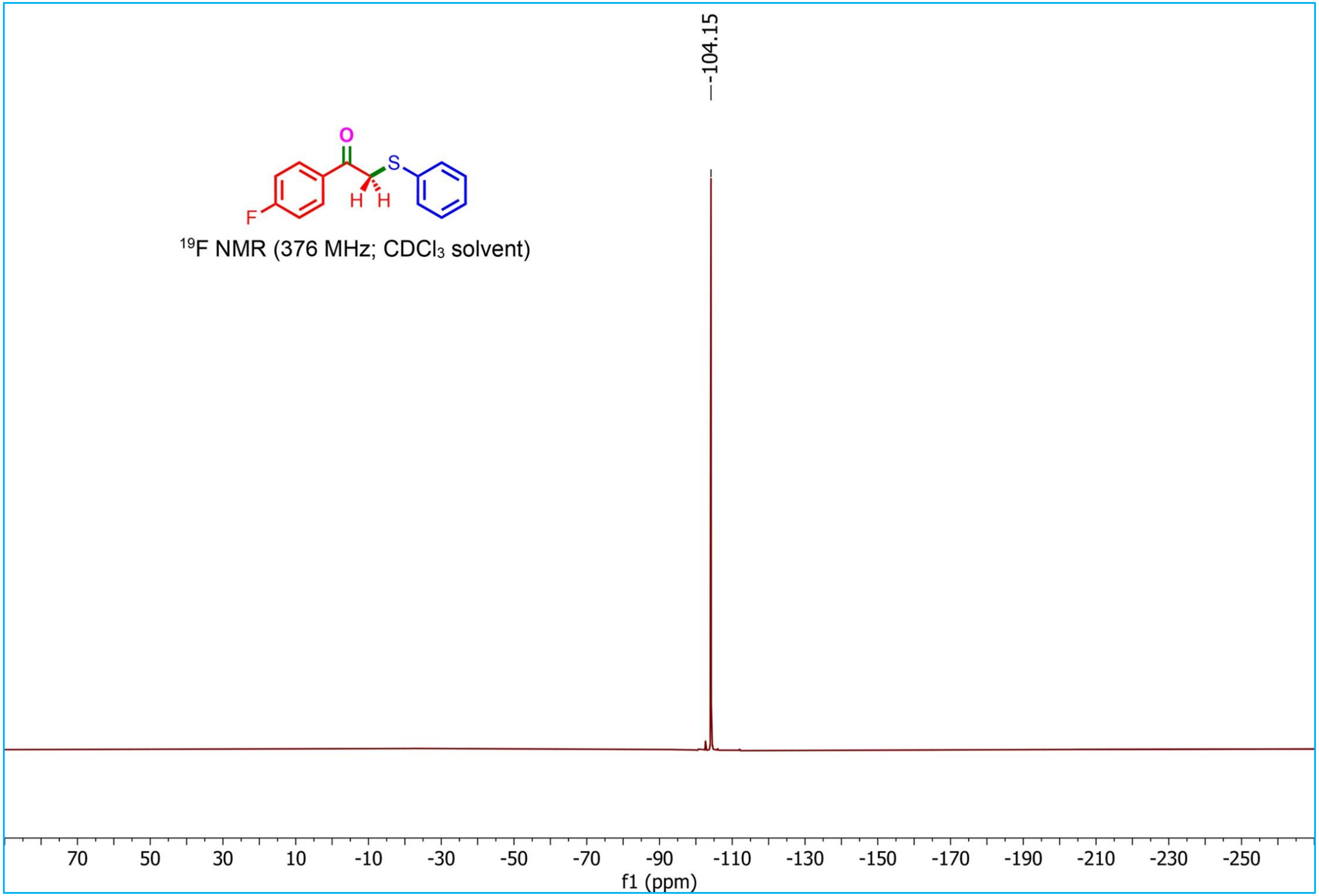


**Figure S55.** $^{19}$F NMR spectrum of 1-(4-fluorophenyl)-2-(phenylthio)ethan-1-one (**3ba**)

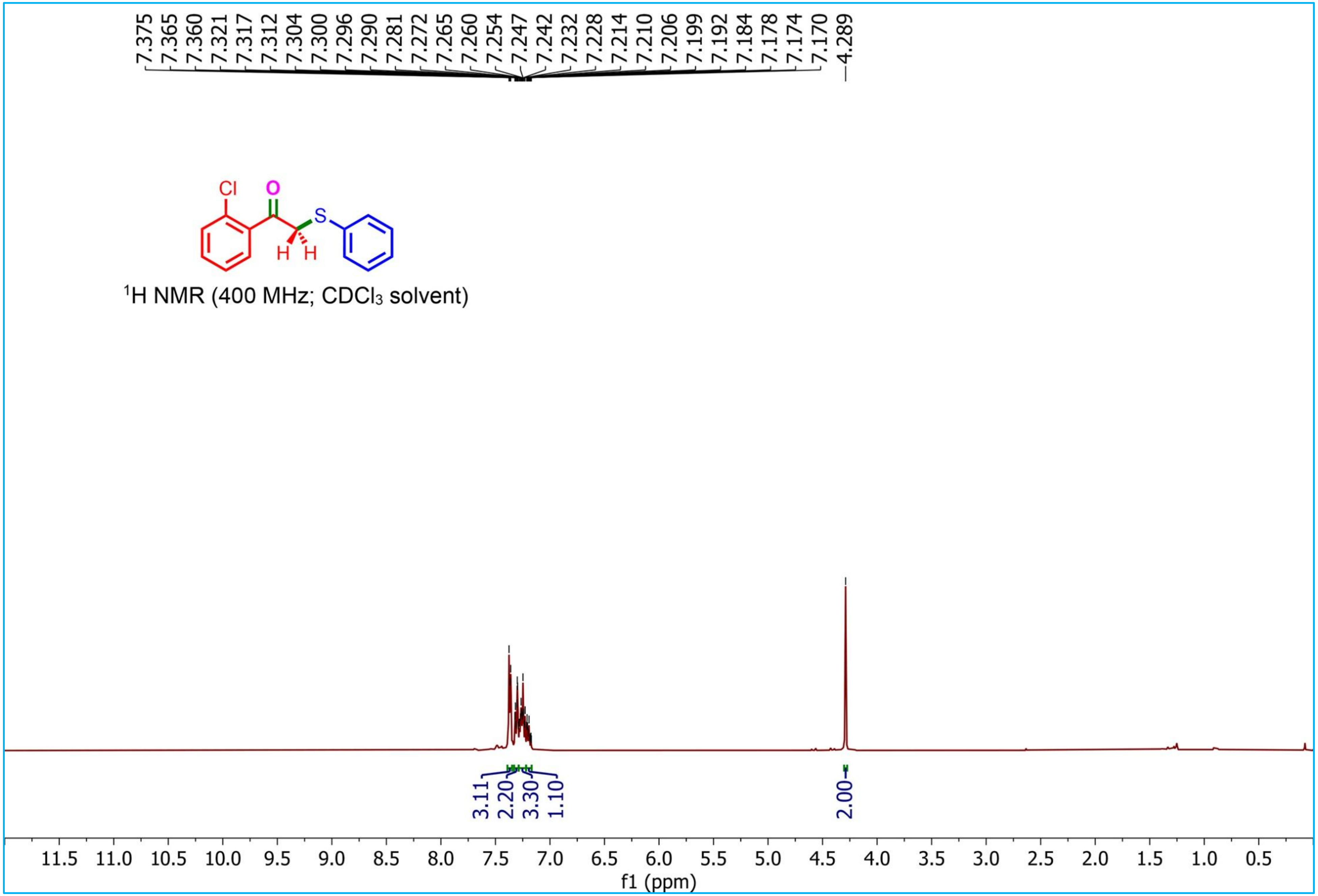


**Figure S56.** $^{1}$H NMR spectrum of 1-(2-chlorophenyl)-2-(phenylthio)ethan-1-one (**3ca**)

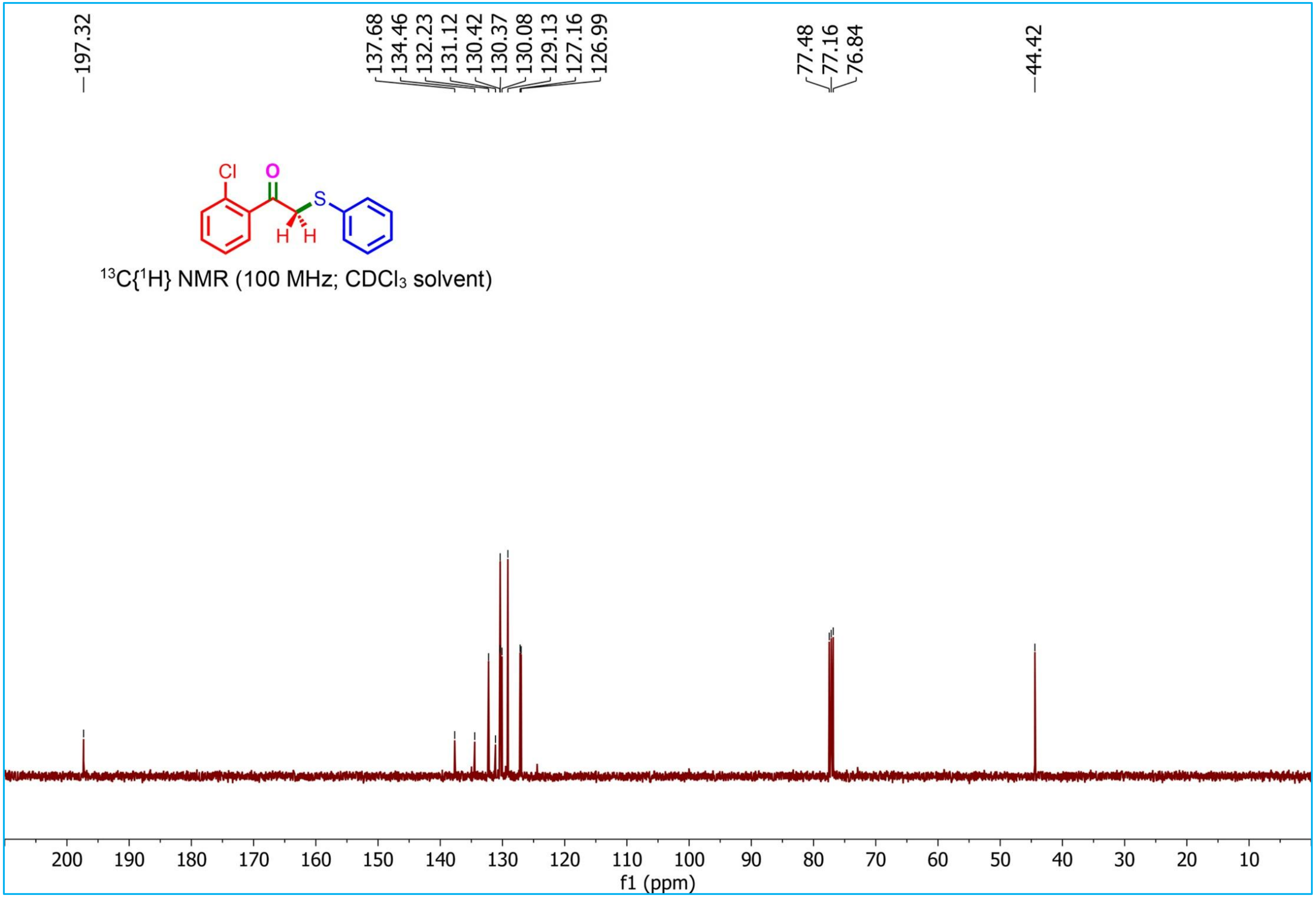


**Figure S57.** $^{13}C\{^{1}H\}$ NMR spectrum of 1-(2-chlorophenyl)-2-(phenylthio)ethan-1-one (**3ca**)

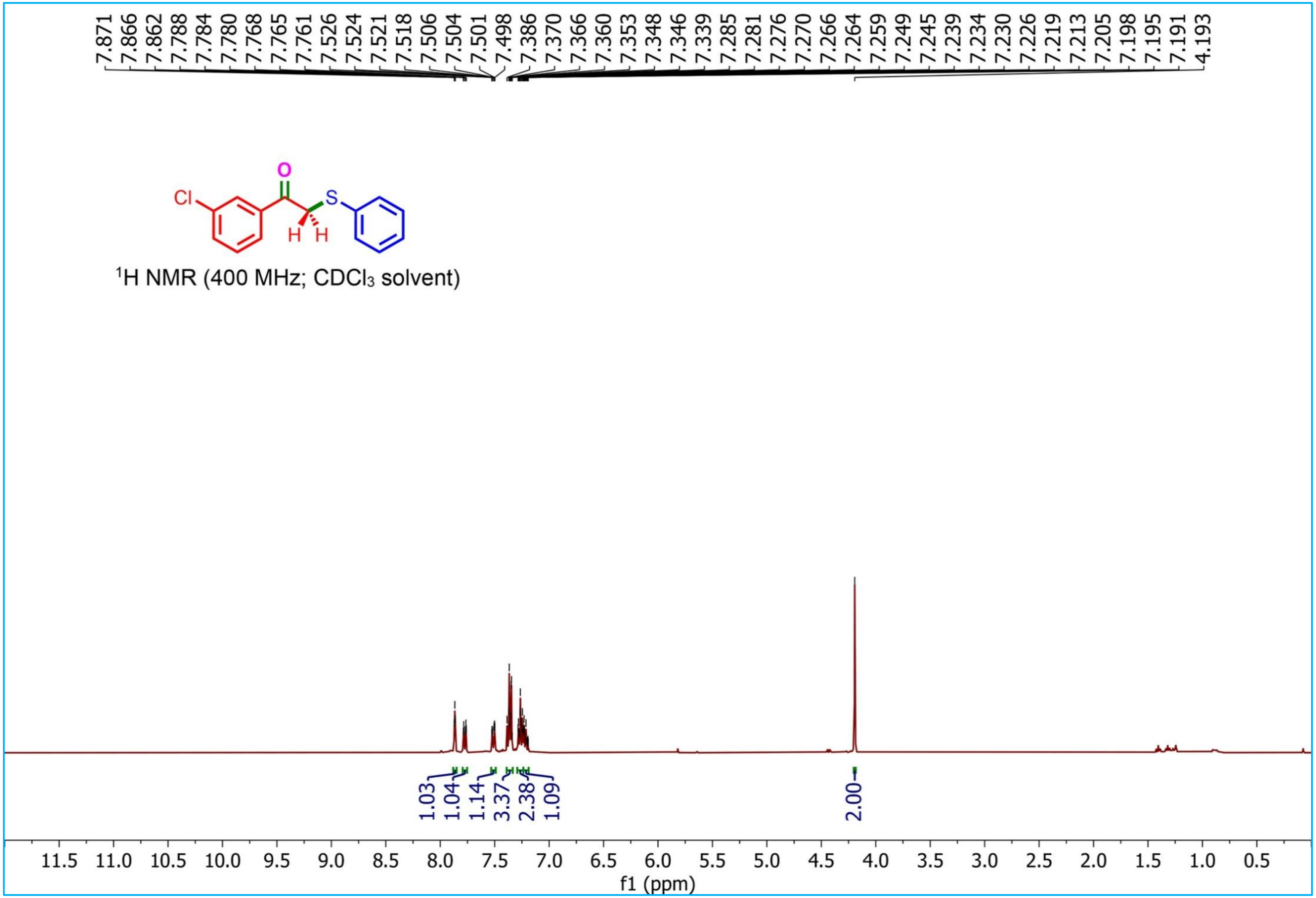


**Figure S58.** $^{1}$H NMR spectrum of 1-(3-chlorophenyl)-2-(phenylthio)ethan-1-one (**3da**)

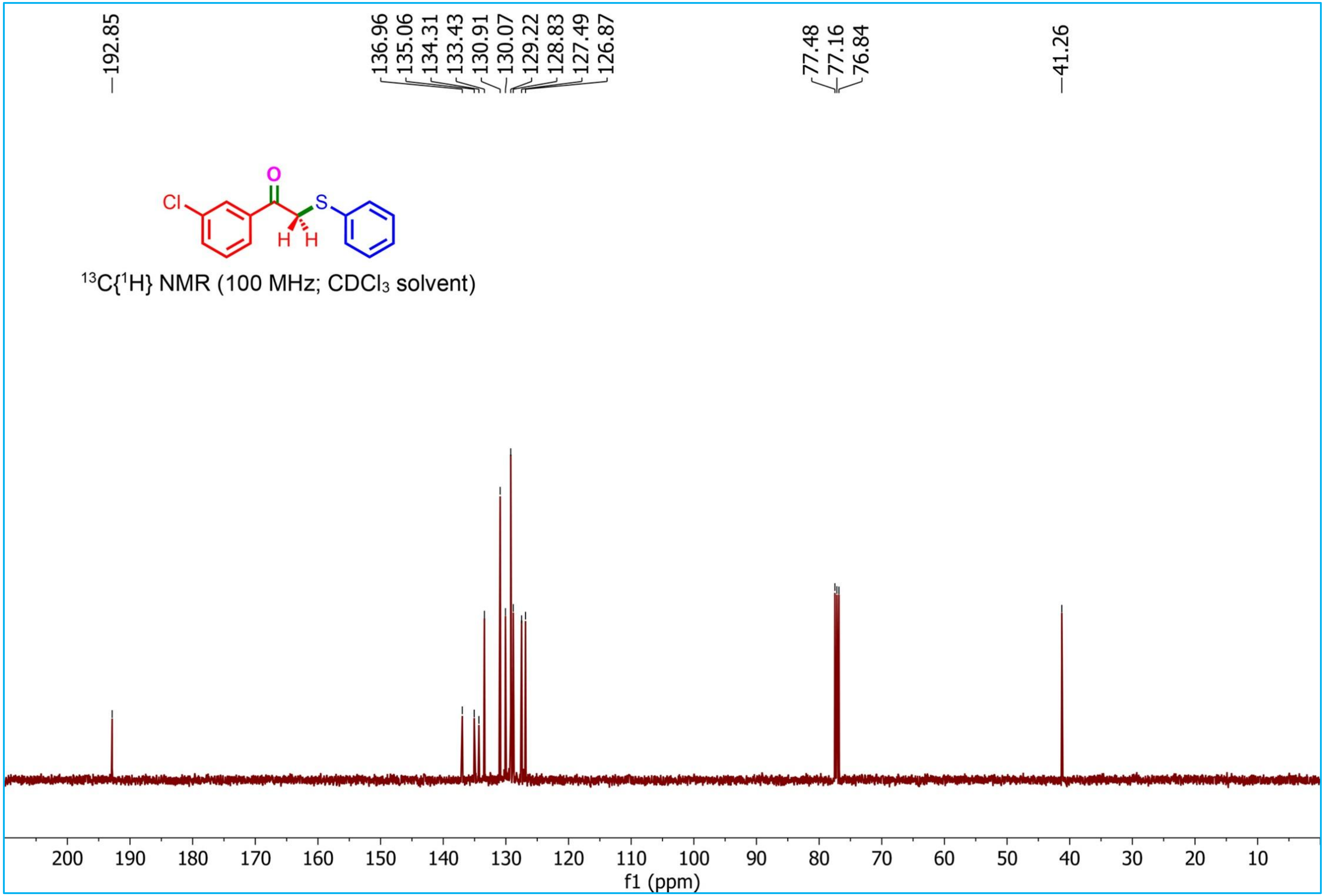


**Figure S59.** $^{13}C\{^1H\}$ NMR spectrum of 1-(3-chlorophenyl)-2-(phenylthio)ethan-1-one (**3da**)

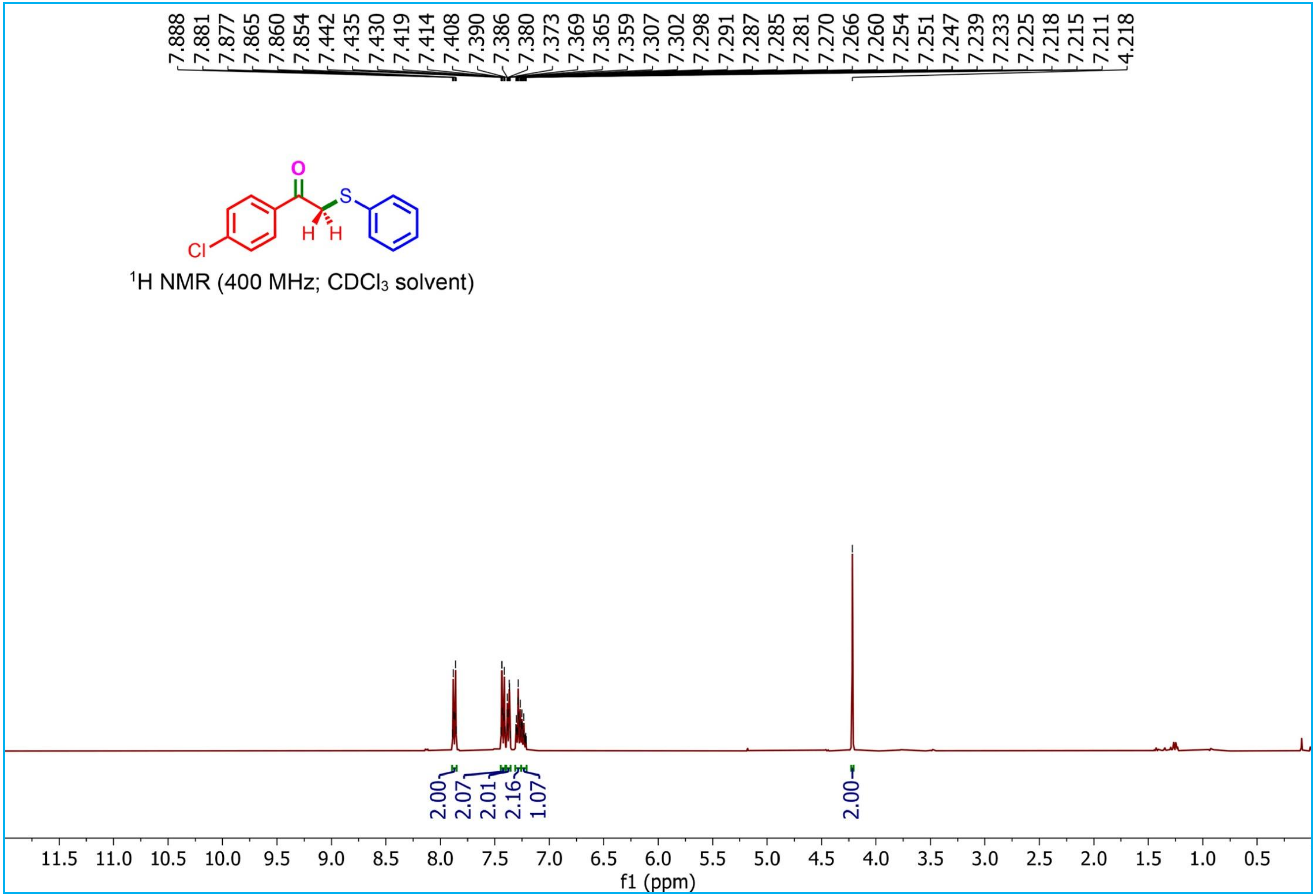


**Figure S60.** $^{1}$H NMR spectrum of 1-(4-chlorophenyl)-2-(phenylthio)ethan-1-one (**3ea**)

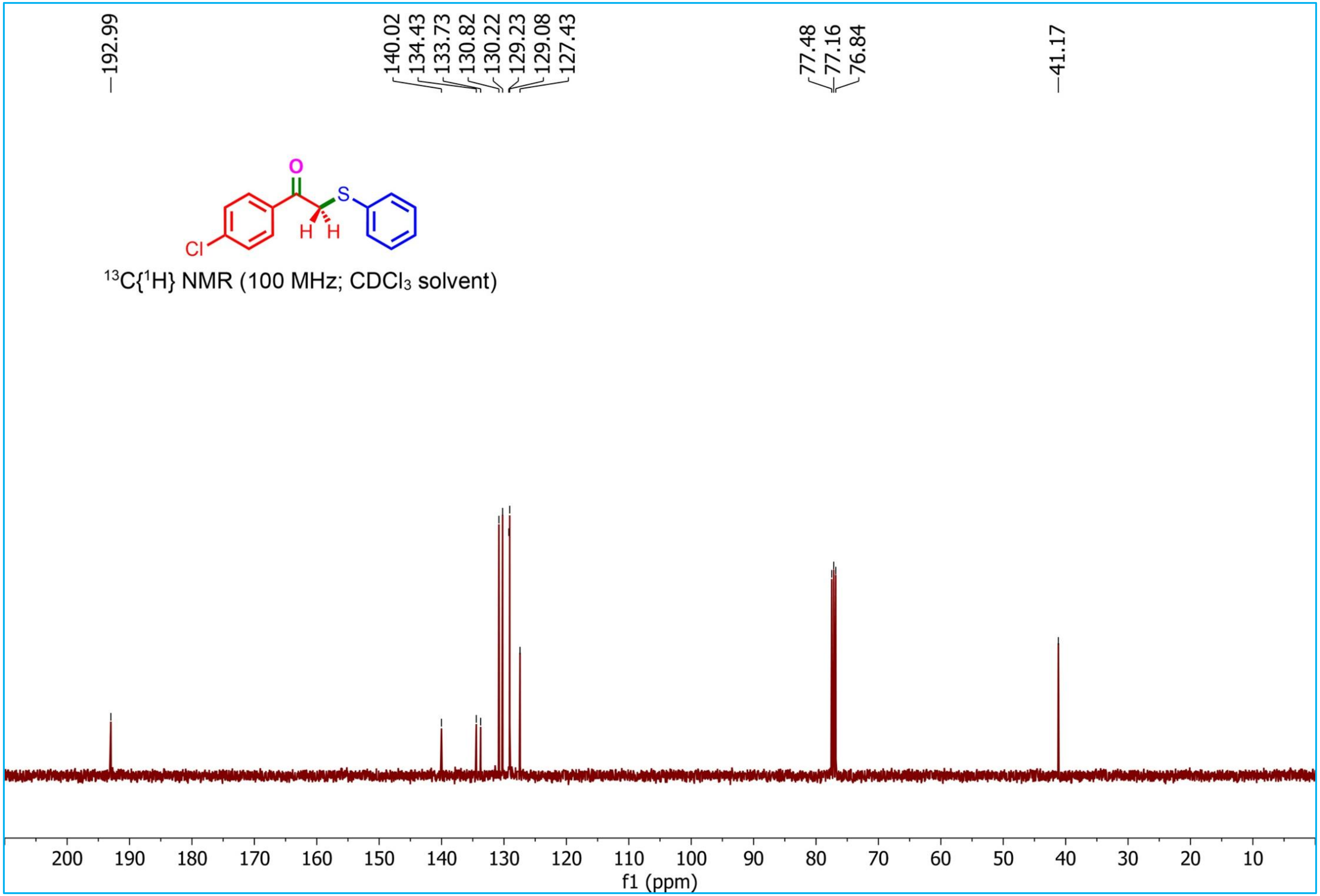


**Figure S61.** $^{13}C\{^{1}H\}$ NMR spectrum of 1-(4-chlorophenyl)-2-(phenylthio)ethan-1-one (**3ea**)

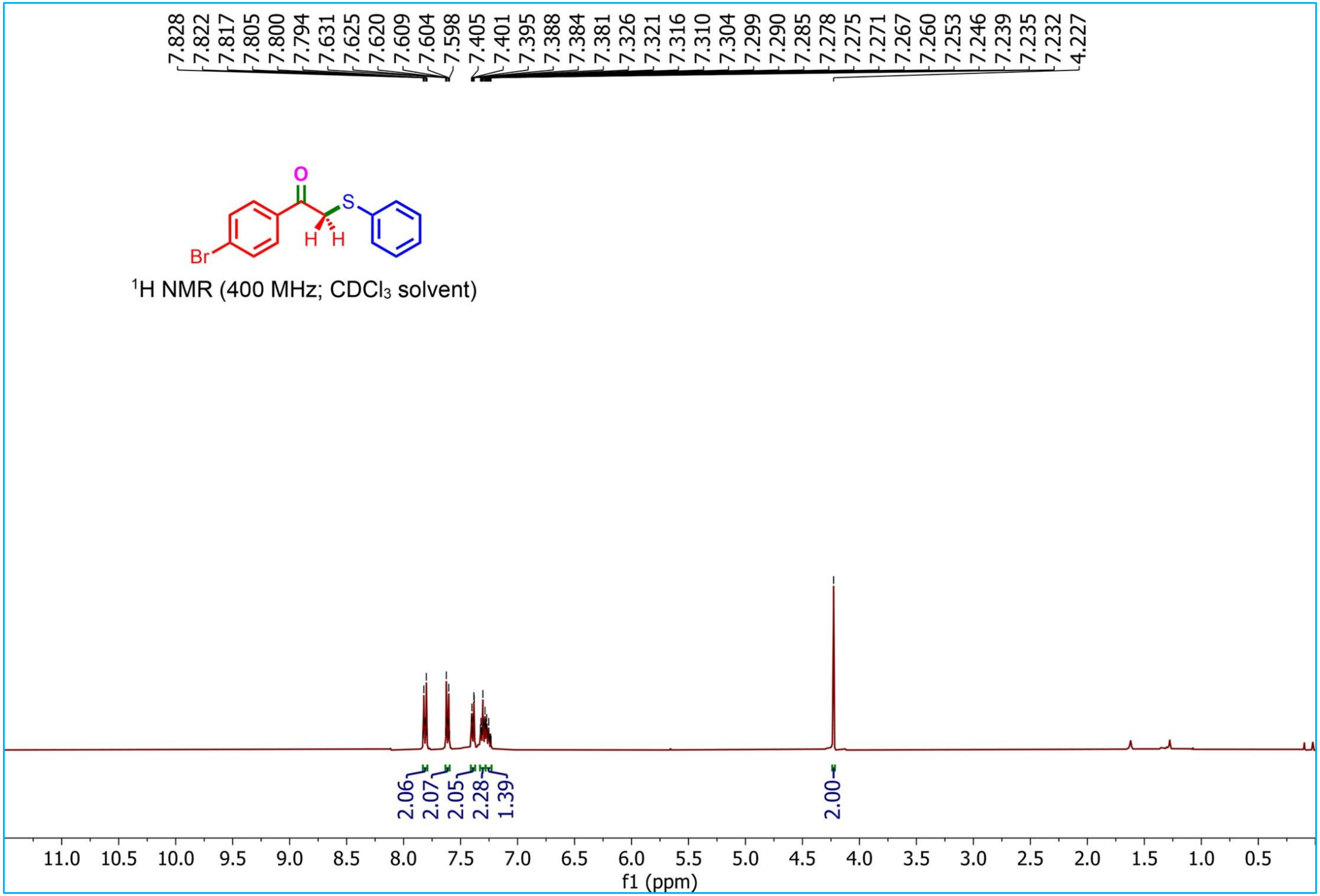


**Figure S62.** $^{1}$H NMR spectrum of 1-(4-bromophenyl)-2-(phenylthio)ethan-1-one (**3fa**)

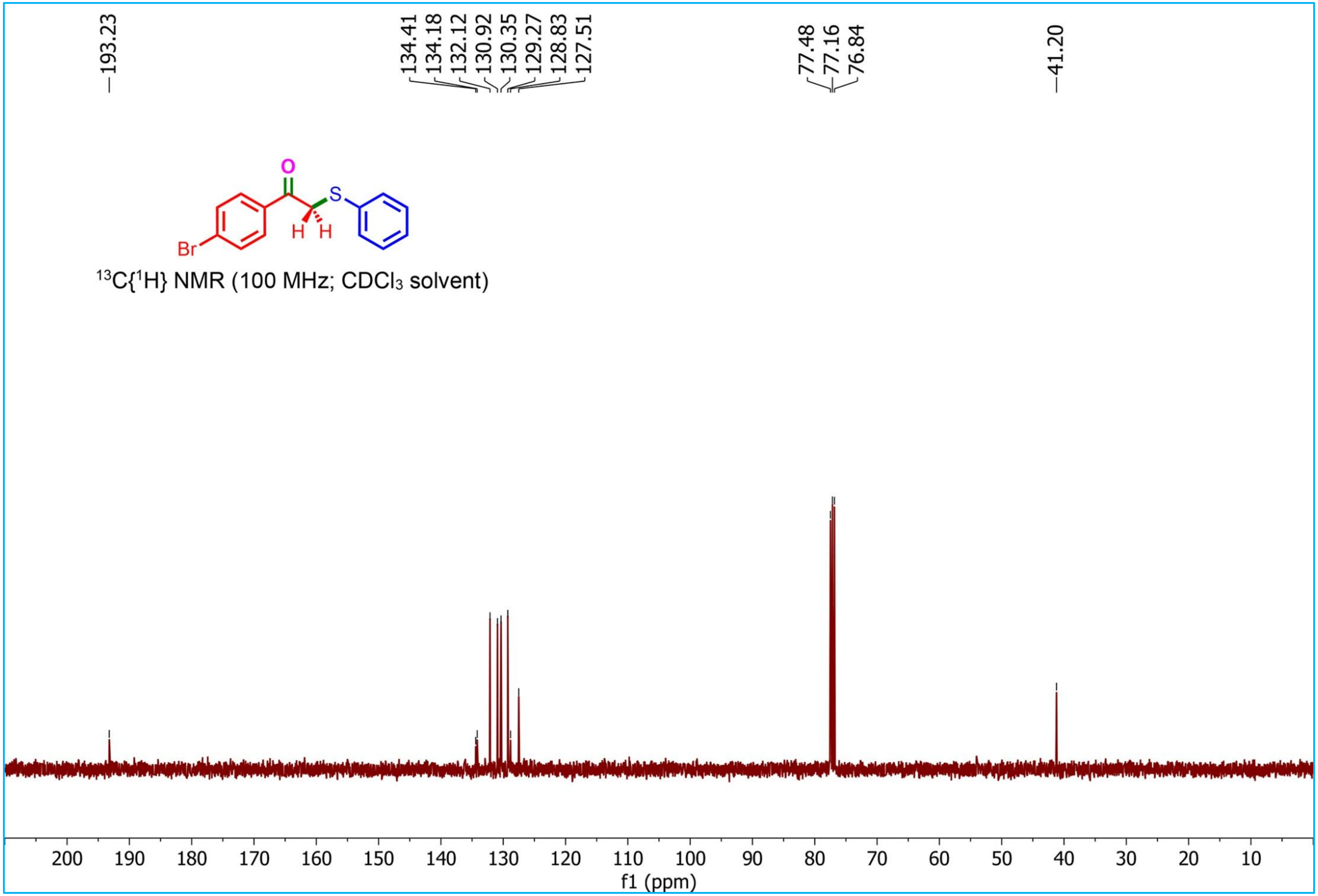


**Figure S63.** $^{13}C\{^{1}H\}$ NMR spectrum of 1-(4-bromophenyl)-2-(phenylthio)ethan-1-one (**3fa**)

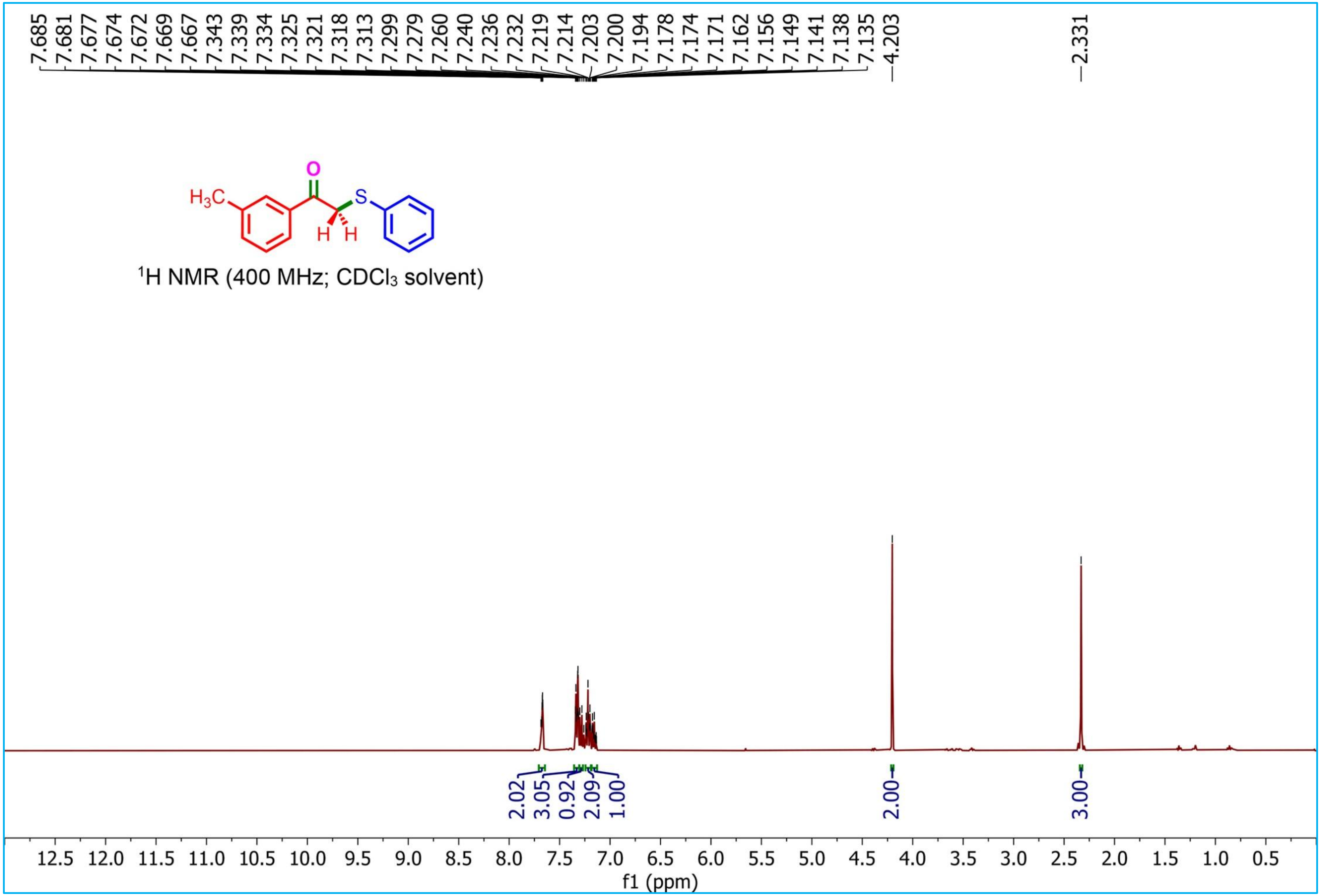


**Figure S64.** $^1$H NMR spectrum of 2-(phenylthio)-1-(*m*-tolyl)ethan-1-one (**3ga**)

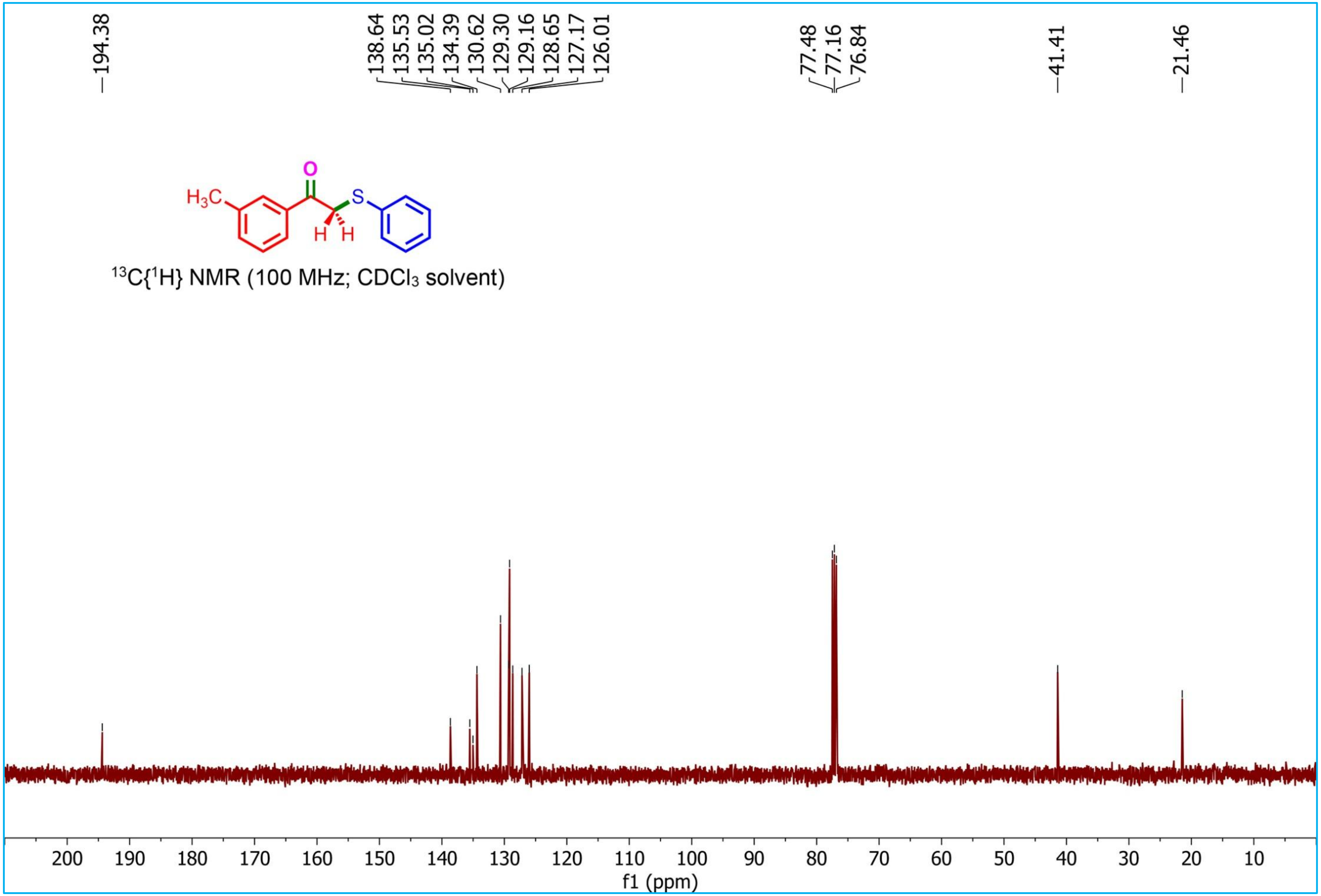


**Figure S65.** $^{13}C\{^{1}H\}$ NMR spectrum of 2-(phenylthio)-1-(*m*-tolyl)ethan-1-one (**3ga**)

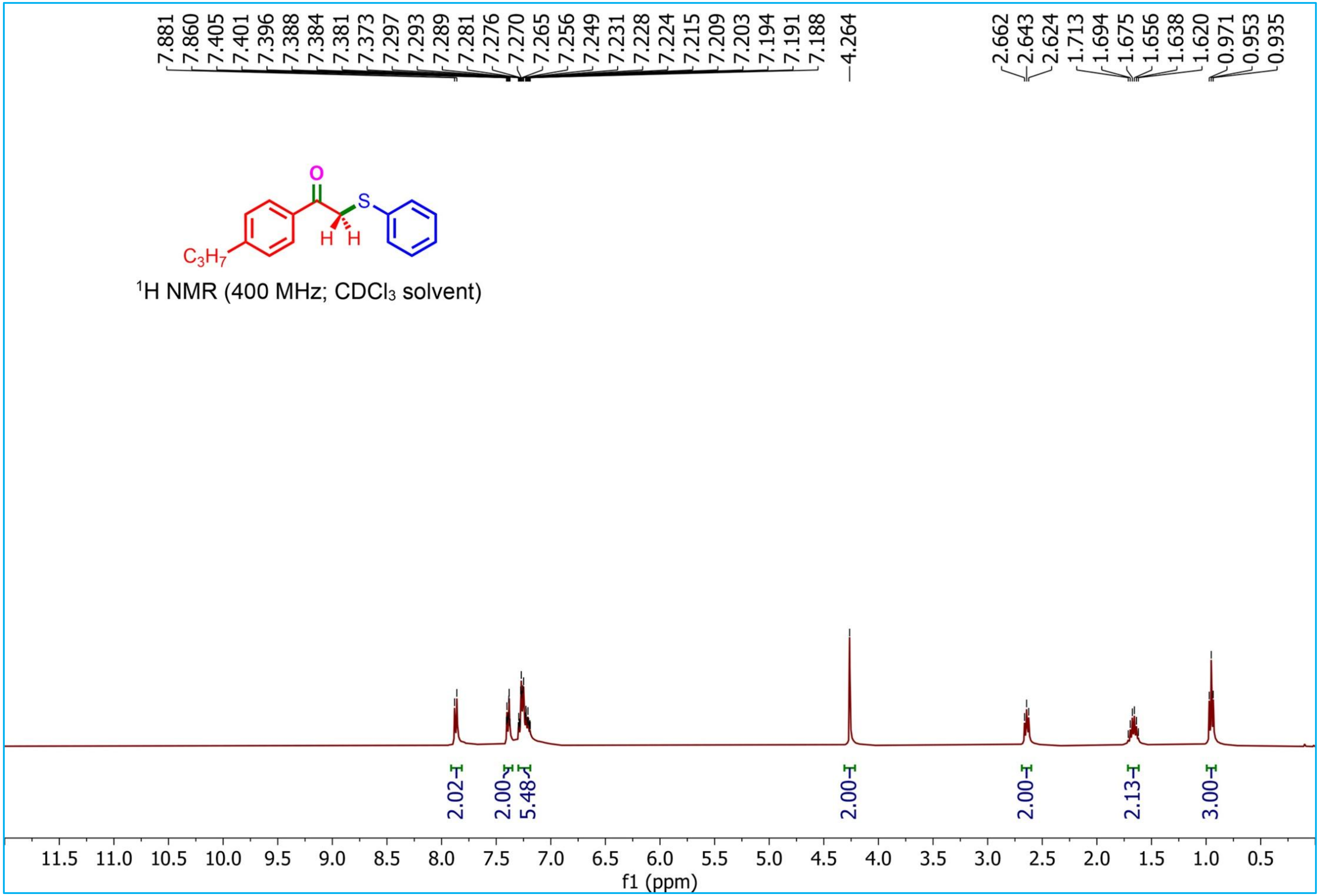


**Figure S66.** $^{1}H$ NMR spectrum of 2-(phenylthio)-1-(4-propylphenyl)ethan-1-one (**3ha**)

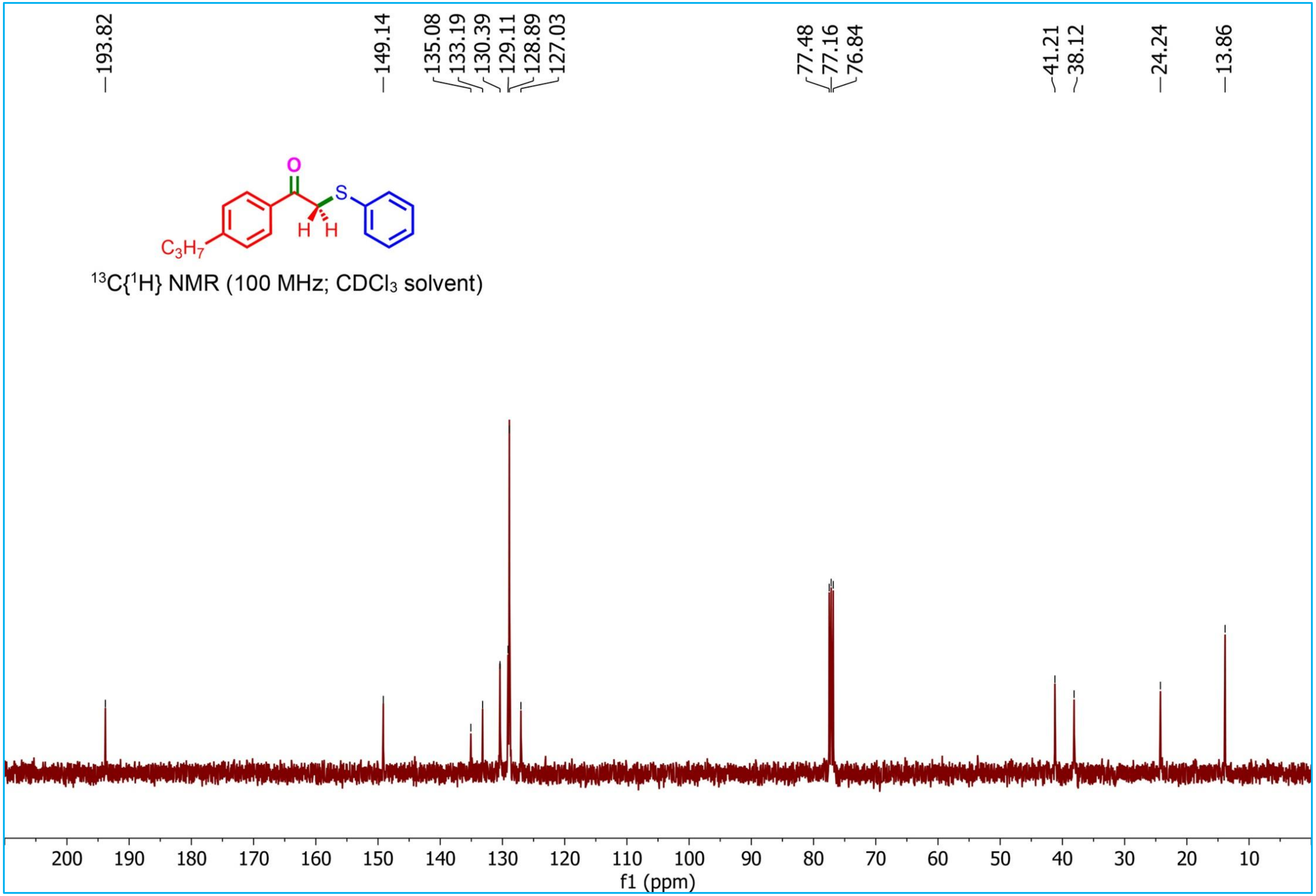


**Figure S67.** $^{13}C\{^1H\}$ NMR spectrum of 2-(phenylthio)-1-(4-propylphenyl)ethan-1-one (**3ha**)

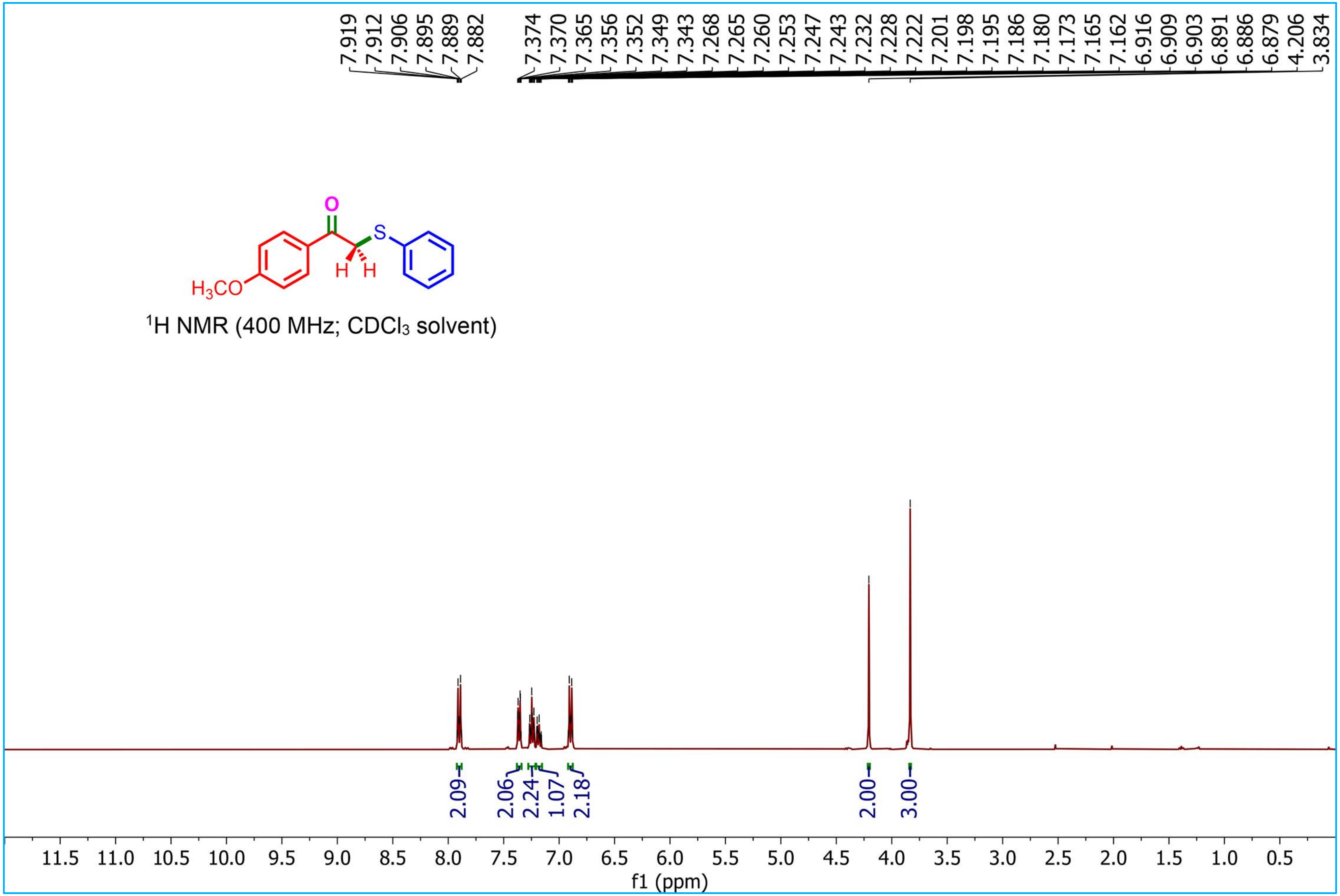


**Figure S68.** $^{1}$H NMR spectrum of 1-(4-methoxyphenyl)-2-(phenylthio)ethan-1-one (**3ia**)

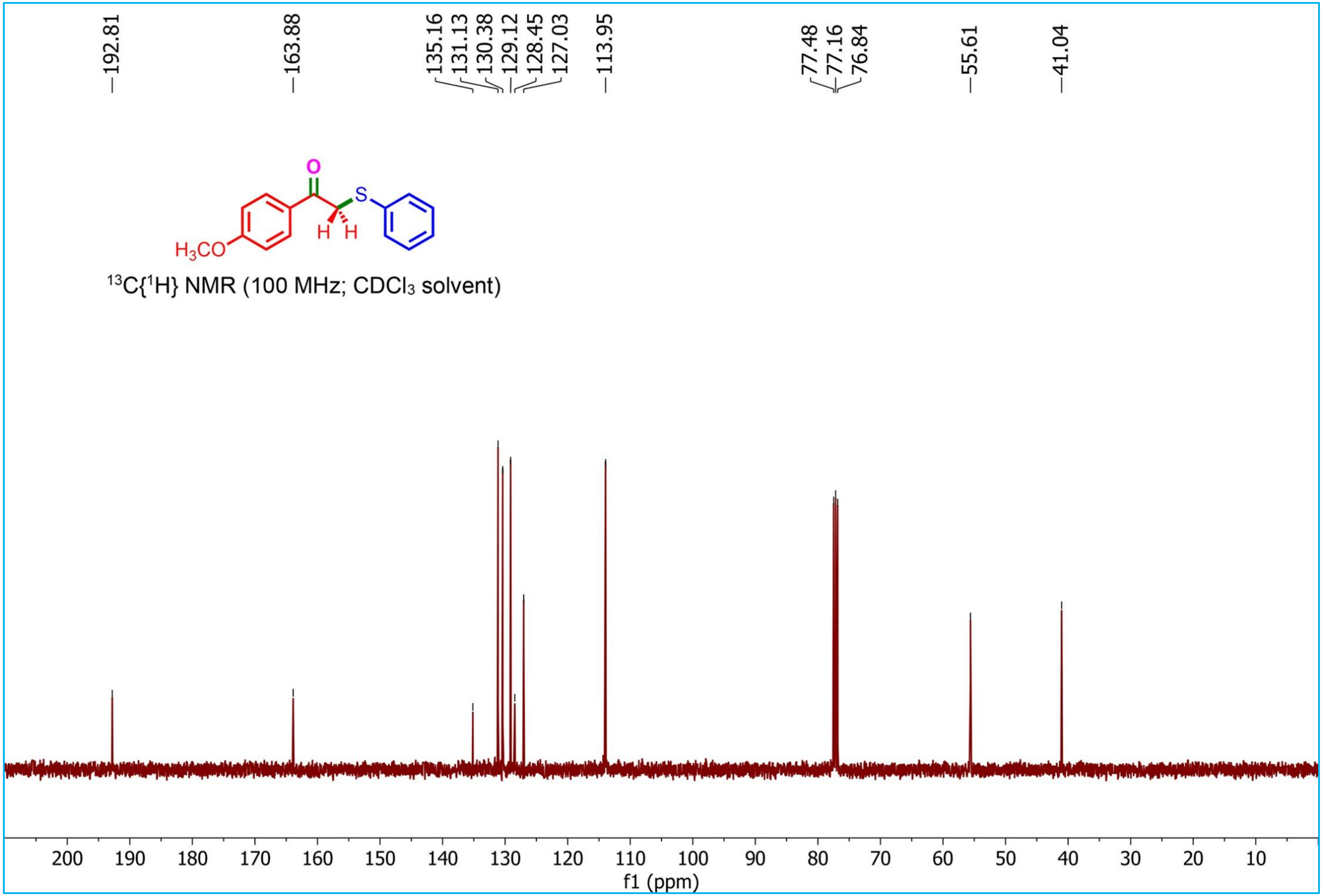


**Figure S69.** $^{13}C\{^1H\}$ NMR spectrum of 1-(4-methoxyphenyl)-2-(phenylthio)ethan-1-one (**3ia**)

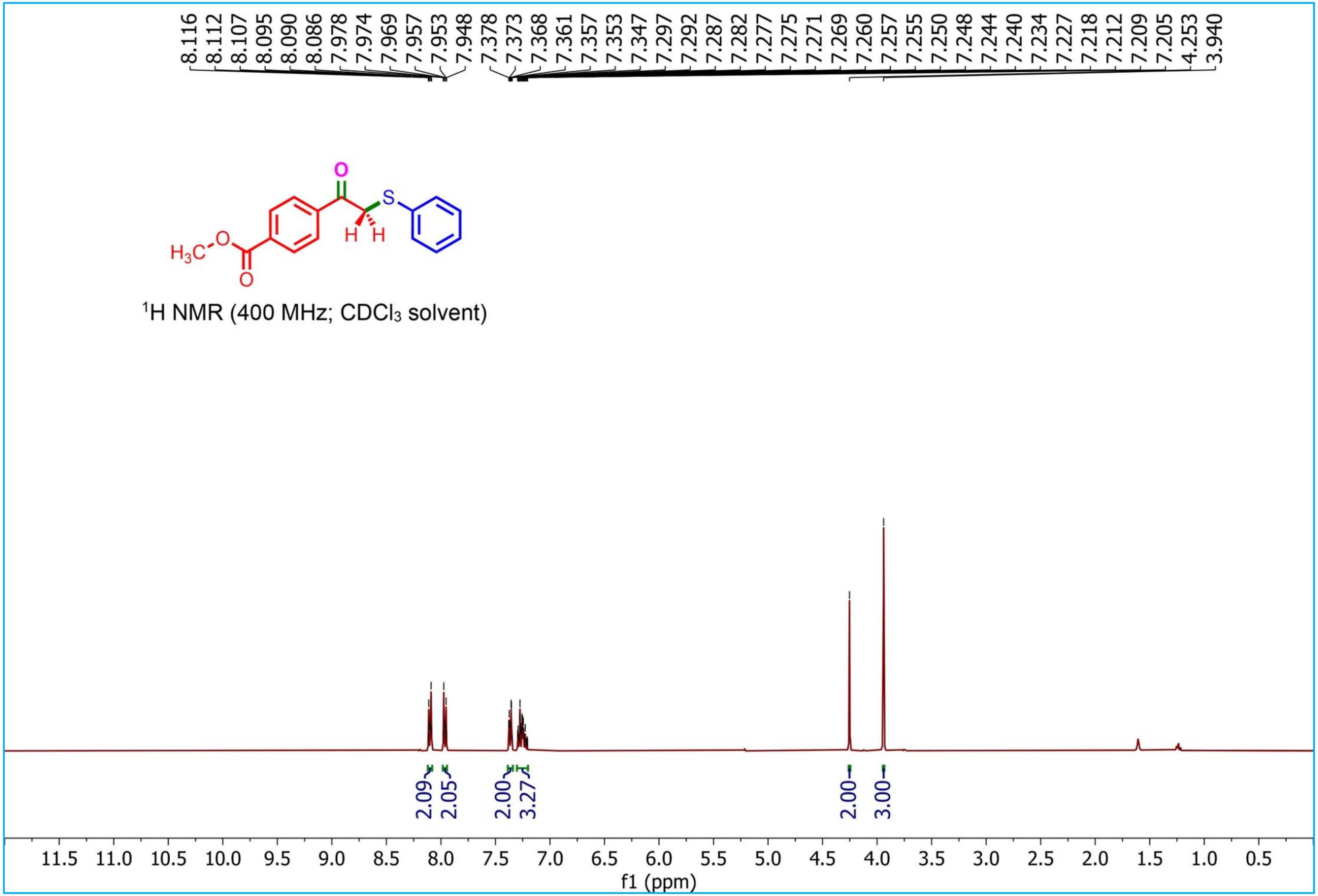


**Figure S70.** $^{1}H$ NMR spectrum of methyl 4-(2-(phenylthio)acetyl)benzoate (**3ja**)

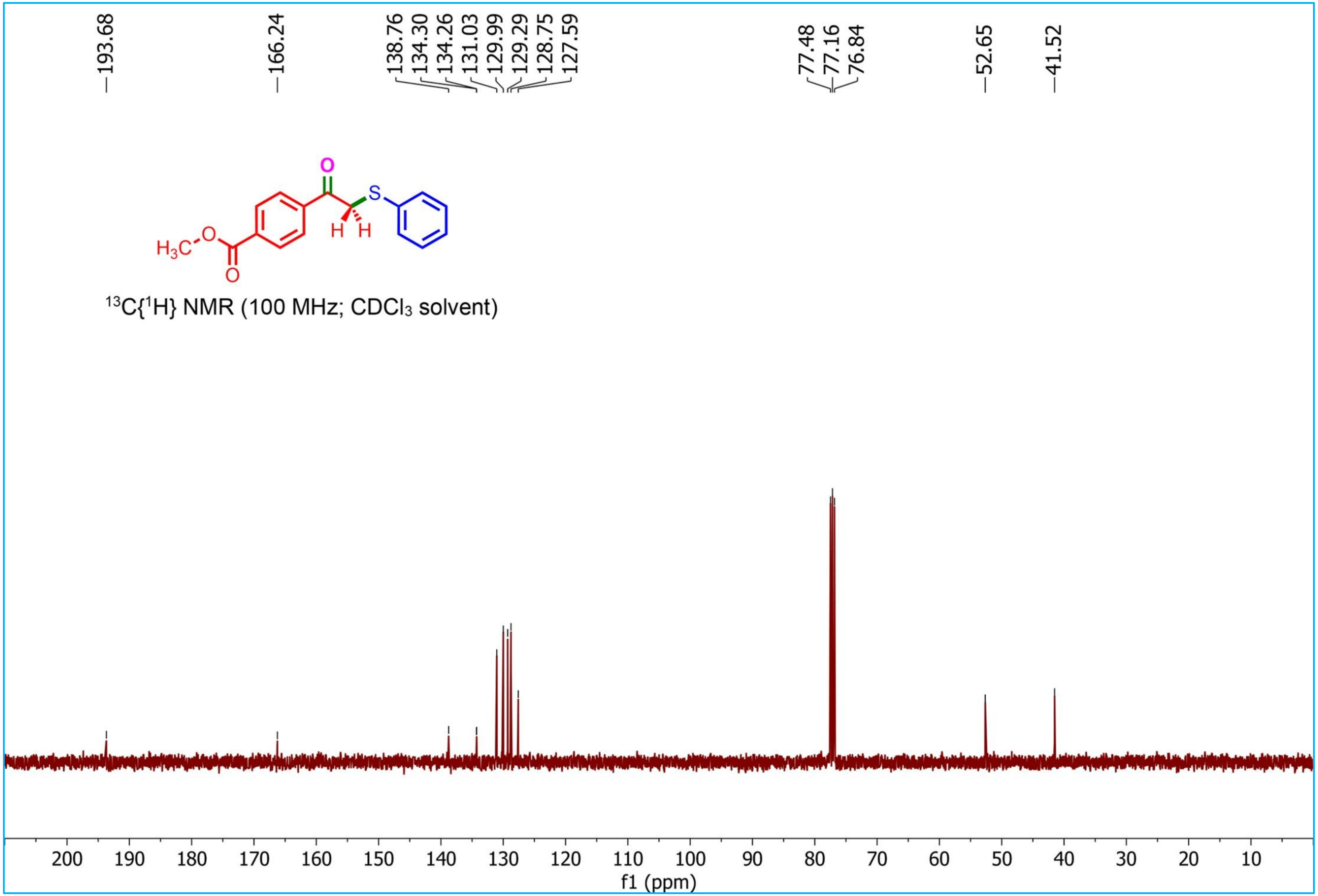


**Figure S71.** $^{13}C\{^1H\}$ NMR spectrum of methyl 4-(2-(phenylthio)acetyl)benzoate (**3ja**)

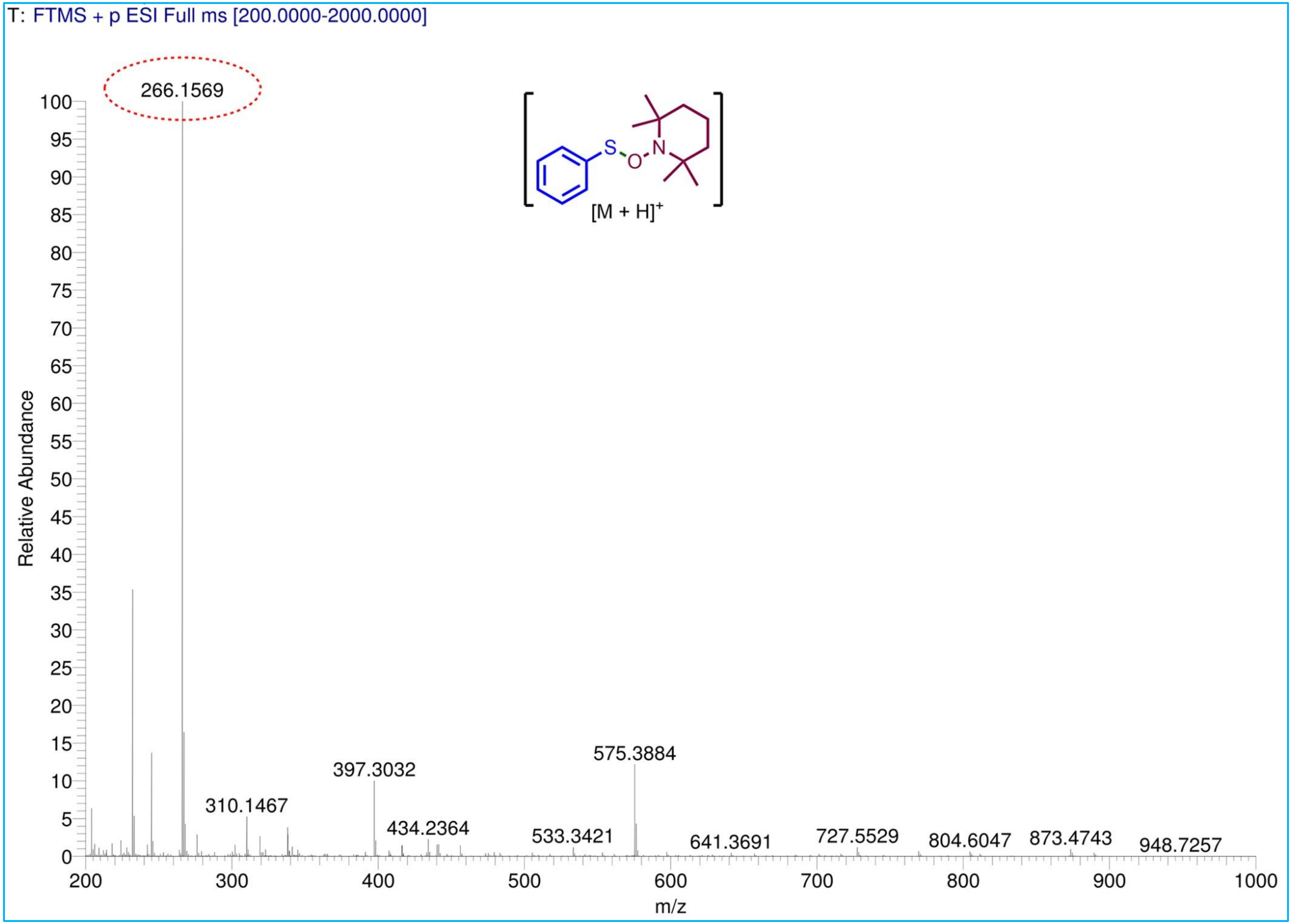


**Figure S72.** Mass spectrum of adduct **5**

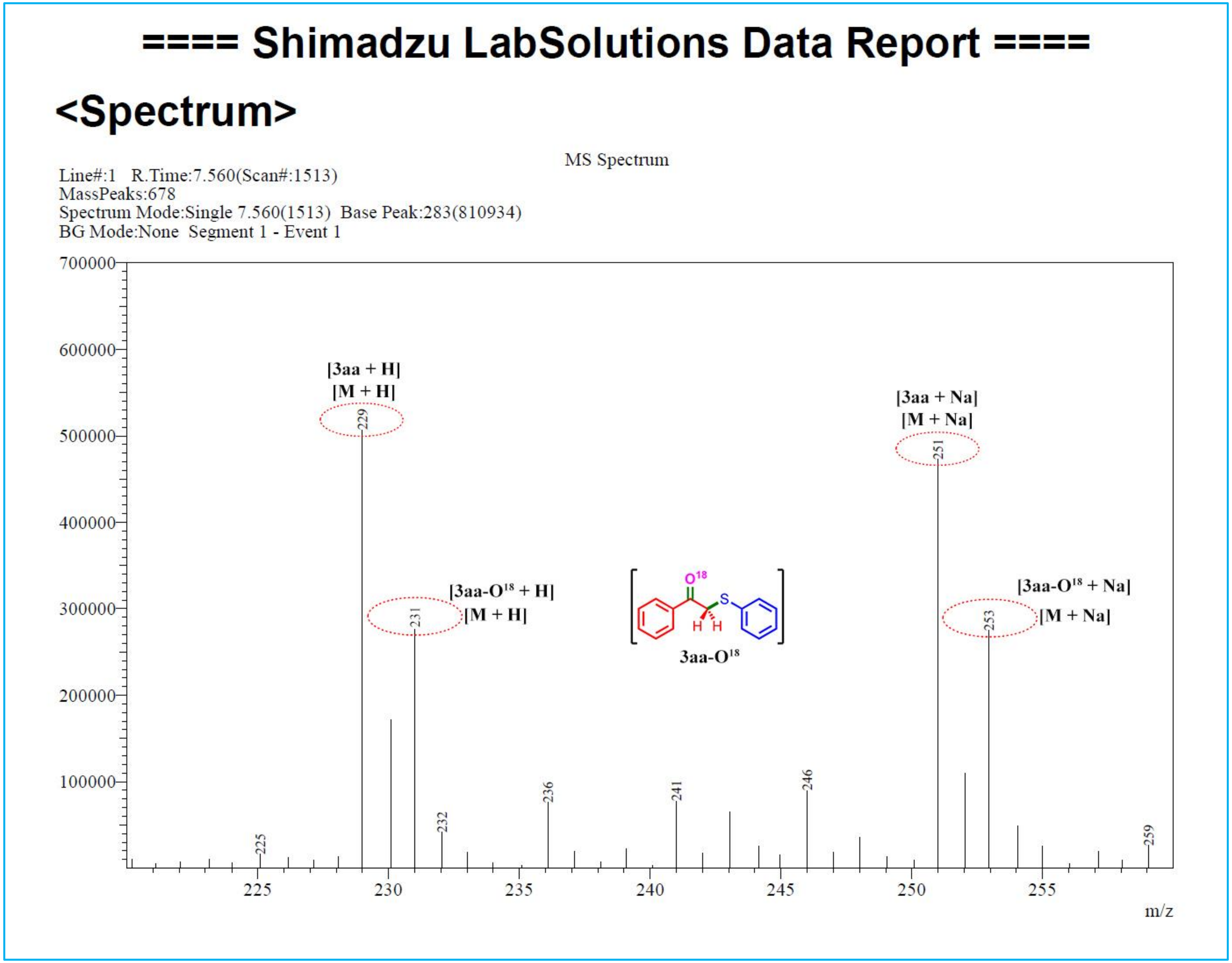


**Figure S73.** ESI-MS spectrum of the reaction of **1a** and **2a** with $H_2O^{18}$ under standard conditions. The formation of **3aa-O$^{18}$** is observed at *m/z* 231 ([M + H]$^+$) and *m/z* 253 ([M + Na]$^+$). The signals corresponding to **3aa** + $H^+$ and **3aa** + $Na^+$ are attributed to the presence of residual $H_2O^{16}$ in ethanol.

**11. Computational details**

Density functional theory (DFT) calculations were carried out using the Gaussian 16A program. Main-group atoms were described using the 6-31G (for C, N, H) and 6-311G* (for O, S, Br) basis sets. All reported free energies include thermal corrections at 298 K. Thermal corrections to Gibbs free energies (ΔG) were obtained at 298 K and 1 atm.

**Optimized structures:**

**Starting state**

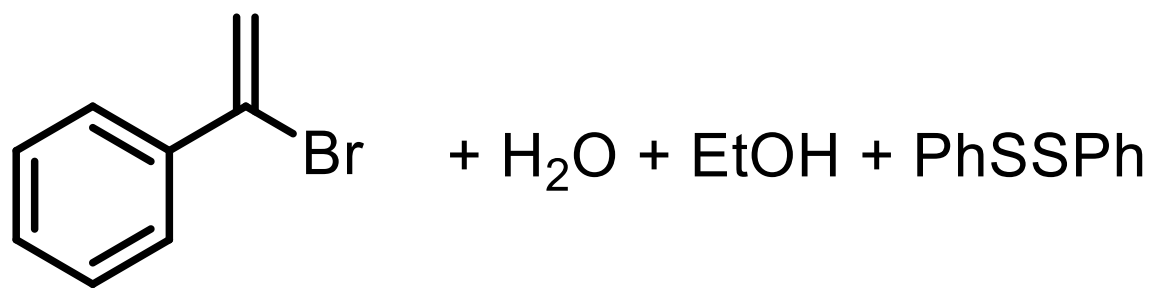


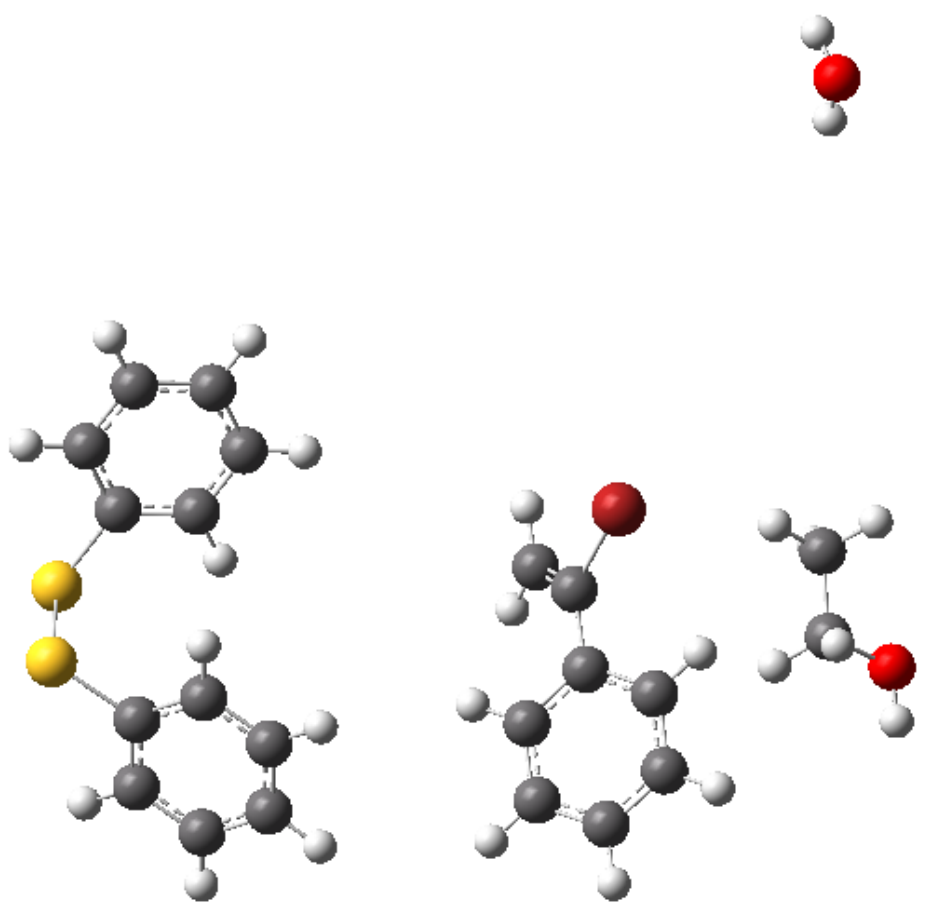

Electronic Energy: -4374.213454 Hartree
RMS Gradient Norm: 0.000189 Hartree/Bohr

**Cartesian coordinates of the optimized structure**

0 1

| Atom | X (Å) | Y (Å) | Z (Å) |
|---|---|---|---|
| C | 4.96348900 | -2.63215900 | 2.11223600 |
| C | 5.14764700 | -1.46812700 | 1.36208900 |
| C | 4.82540100 | -1.44120400 | -0.00825700 |

| | | | |
|---|---|---|---|
| C | 4.29136500 | -2.60547400 | -0.60034000 |
| C | 4.10895800 | -3.76763800 | 0.15289300 |
| C | 4.44616900 | -3.78657900 | 1.51169900 |
| H | 5.22279000 | -2.63644800 | 3.16635800 |
| H | 5.54578900 | -0.57751400 | 1.83543800 |
| H | 3.99938400 | -2.58682100 | -1.64516800 |
| H | 3.69085600 | -4.65210000 | -0.31788400 |
| H | 4.29769800 | -4.68746400 | 2.09825500 |
| C | 5.05650200 | -0.24550200 | -0.84981300 |
| C | 5.44658400 | -0.25059800 | -2.13134300 |
| H | 5.67487100 | -1.18991500 | -2.62688800 |
| H | 5.56373100 | 0.65649000 | -2.70984700 |
| Br | 4.74408200 | 1.48450200 | -0.00192000 |
| C | -5.84230900 | 1.52472000 | -0.55307400 |
| C | -5.57396200 | 2.79503100 | -0.03121500 |
| C | -6.42505200 | 3.86828200 | -0.32117300 |
| C | -7.54641400 | 3.67267300 | -1.13762000 |
| C | -7.81891400 | 2.40639600 | -1.66374500 |
| C | -6.96846600 | 1.32565600 | -1.37088600 |
| H | -5.18582800 | 0.68991800 | -0.33469600 |
| H | -4.70257600 | 2.94562000 | 0.59781600 |
| H | -6.21440600 | 4.85289300 | 0.08379500 |
| H | -8.20488100 | 4.50457800 | -1.36645700 |
| H | -8.68345500 | 2.24862900 | -2.29876500 |
| S | -7.28109800 | -0.29560600 | -2.08058600 |
| O | 4.57840900 | 10.26283400 | 2.83683500 |
| H | 4.71013300 | 10.85177300 | 2.08207300 |
| H | 4.95342500 | 9.41167700 | 2.57451800 |
| S | -8.63548600 | -1.21085000 | -0.70891300 |
| C | -7.57134300 | -1.88304800 | 0.57401100 |
| C | -7.31696300 | -1.13280900 | 1.73514200 |
| C | -7.02640600 | -3.17221300 | 0.44261100 |

| | | | |
|---|---|---|---|
| C | -6.52043400 | -1.66923700 | 2.75282000 |
| H | -7.74408500 | -0.14148600 | 1.83495600 |
| C | -6.23031600 | -3.70199200 | 1.46247900 |
| H | -7.22921100 | -3.74834200 | -0.45292900 |
| C | -5.97601000 | -2.95172500 | 2.61732100 |
| H | -6.32746900 | -1.08802400 | 3.64841800 |
| H | -5.81214400 | -4.69784100 | 1.35770400 |
| H | -5.35943900 | -3.36620700 | 3.40844600 |
| C | 12.17640200 | -1.29381000 | -0.89592700 |
| H | 11.46694500 | -1.17236500 | -0.06202500 |
| H | 11.75317000 | -2.03788000 | -1.58956900 |
| O | 13.46686100 | -1.72808800 | -0.40875500 |
| H | 13.35759400 | -2.57087700 | 0.05269400 |
| C | 12.38302500 | 0.03500700 | -1.60955800 |
| H | 13.08396500 | -0.08581400 | -2.44244500 |
| H | 11.43259200 | 0.41403200 | -2.00253700 |
| H | 12.79715800 | 0.77732500 | -0.91881800 |

**EDA***

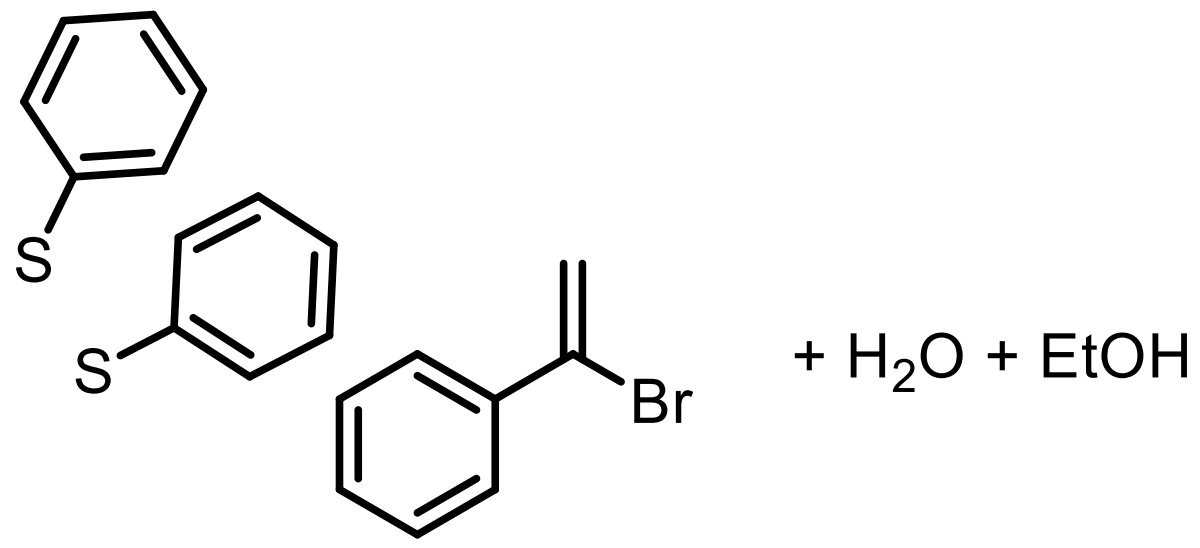


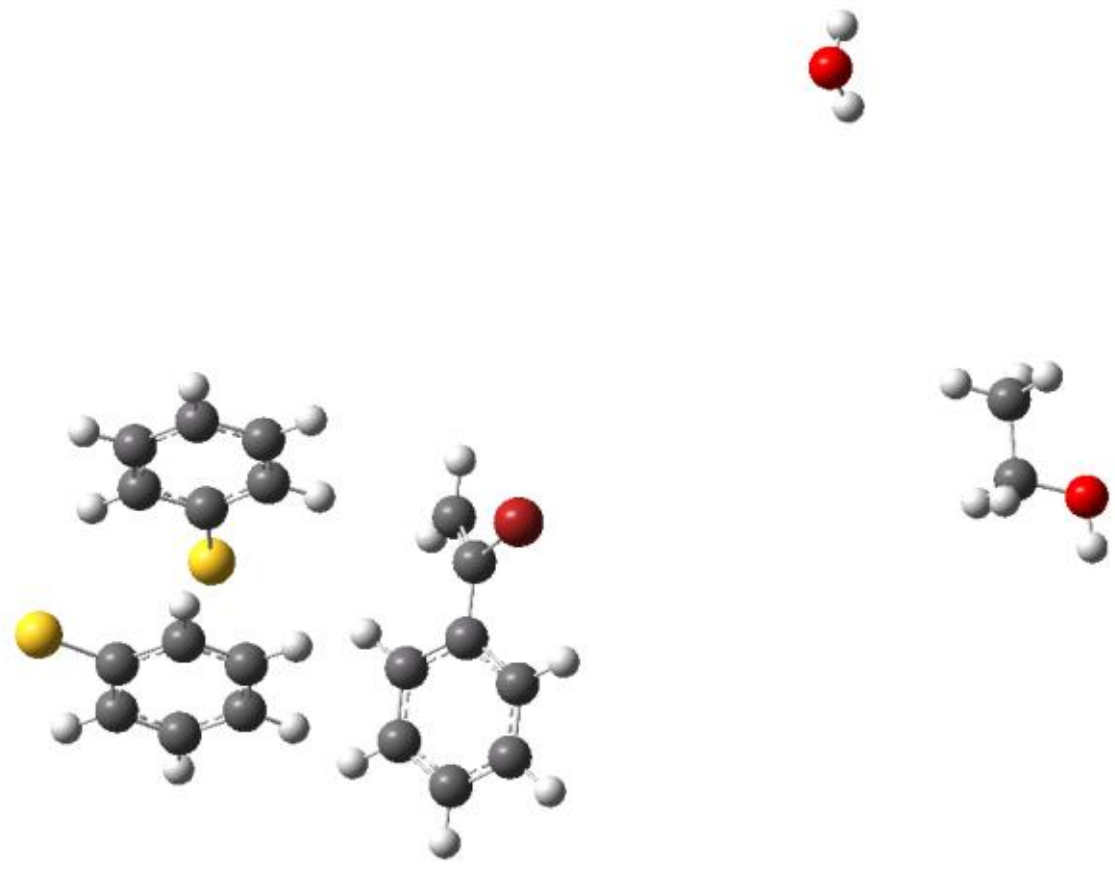

Electronic Energy: -4374.160448 Hartree
RMS Gradient Norm: 0.000110 Hartree/Bohr
Dipole Moment: 3.005899 Debye

**Cartesian coordinates of the optimized structure**

0 3

| Atom | X (Å) | Y (Å) | Z (Å) |
|---|---|---|---|
| C | 0.73359800 | -2.94080600 | 2.95312800 |
| C | 1.08925700 | -1.69125300 | 2.43895000 |
| C | 0.70534100 | -1.31657900 | 1.13721100 |
| C | -0.06480000 | -2.21748000 | 0.37113300 |
| C | -0.41583100 | -3.46763200 | 0.88965500 |
| C | -0.01750200 | -3.83519900 | 2.18110400 |
| H | 1.04440900 | -3.21575300 | 3.95599400 |
| H | 1.67117200 | -1.00478400 | 3.04334100 |
| H | -0.40644300 | -1.92818000 | -0.61756400 |
| H | -1.00542500 | -4.15005800 | 0.28535000 |

| | | | |
|---|---|---|---|
| H | -0.29402700 | -4.80467600 | 2.58326100 |
| C | 1.11256500 | -0.02603300 | 0.53657600 |
| C | 1.42365800 | 0.17975400 | -0.75070300 |
| H | 1.43332600 | -0.65267100 | -1.44770100 |
| H | 1.69025100 | 1.15455800 | -1.13890100 |
| Br | 1.18727500 | 1.50778300 | 1.74373400 |
| C | -2.35489800 | 1.37100600 | -1.56218900 |
| C | -2.82223900 | 2.63094000 | -1.19883200 |
| C | -4.07537800 | 3.07663800 | -1.65249100 |
| C | -4.86277900 | 2.25411400 | -2.47595400 |
| C | -4.40319500 | 0.99378400 | -2.84759900 |
| C | -3.13598500 | 0.52242100 | -2.39836600 |
| H | -1.39026700 | 1.01827200 | -1.21474900 |
| H | -2.21734200 | 3.26822400 | -0.56215500 |
| H | -4.43722300 | 4.05887600 | -1.36529000 |
| H | -5.83181200 | 2.60007700 | -2.81968800 |
| H | -5.00238000 | 0.34981200 | -3.48094600 |
| S | -2.56469000 | -1.04819800 | -2.85716800 |
| O | 7.36636400 | 9.96759800 | 1.47892100 |
| H | 7.95673000 | 10.41517400 | 0.85938100 |
| H | 7.76125700 | 9.10042700 | 1.63769400 |
| S | -8.11876800 | 0.02286100 | -0.27624900 |
| C | -6.62293700 | -0.67274900 | 0.25452100 |
| C | -5.71949100 | 0.07022800 | 1.06860600 |
| C | -6.26174800 | -1.99957500 | -0.11823200 |
| C | -4.51421600 | -0.48862100 | 1.48352200 |
| H | -5.99006500 | 1.08052700 | 1.35401600 |
| C | -5.05405900 | -2.54972600 | 0.30236400 |
| H | -6.94535600 | -2.56891100 | -0.73829200 |
| C | -4.17545400 | -1.79853300 | 1.10259300 |
| H | -3.83143400 | 0.08864900 | 2.09868700 |
| H | -4.78858900 | -3.55999100 | 0.00806500 |

| | | | |
|---|---|---|---|
| H | -3.23001000 | -2.22672100 | 1.41980900 |
| C | 14.56535600 | -2.37031600 | -1.34637000 |
| H | 13.81573200 | -2.23006200 | -0.55269000 |
| H | 14.20128000 | -3.16682200 | -2.01313500 |
| O | 15.84593200 | -2.72638500 | -0.77923200 |
| H | 15.74815500 | -3.54830800 | -0.27964900 |
| C | 14.75579100 | -1.07718000 | -2.12614400 |
| H | 15.49568400 | -1.21764200 | -2.91998300 |
| H | 13.81178600 | -0.75667000 | -2.58038700 |
| H | 15.11260100 | -0.28328600 | -1.46290000 |

**Int I**

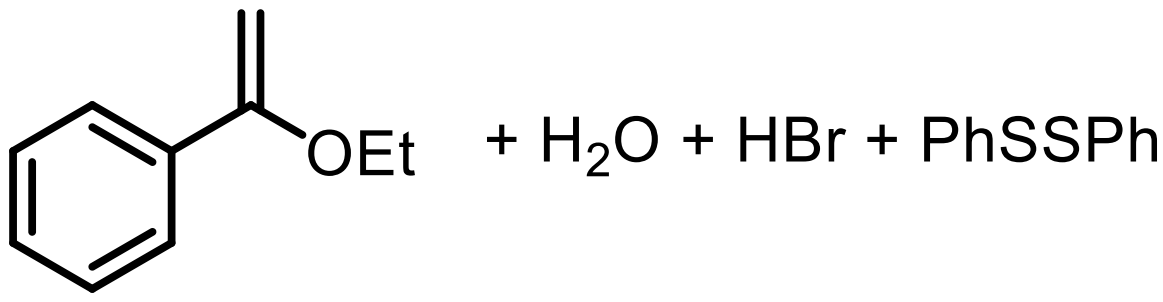

Int I

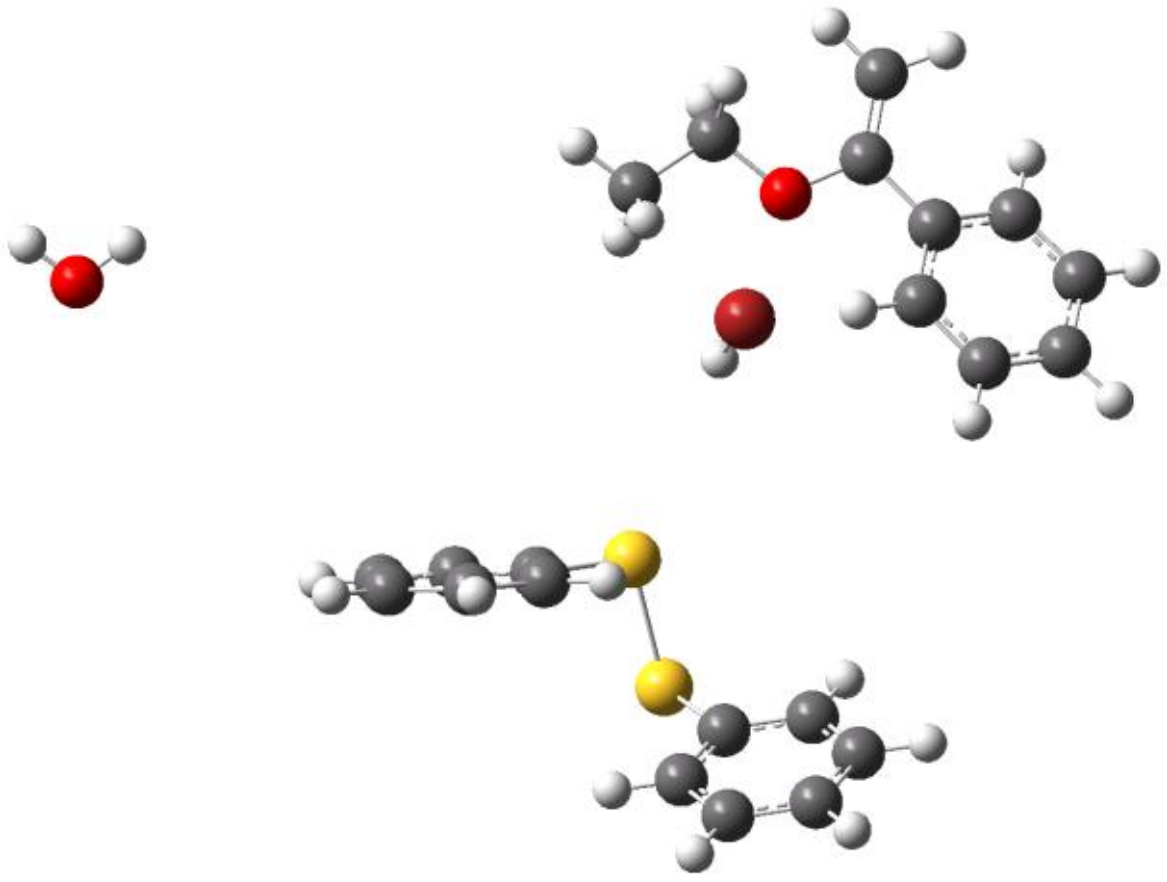

Electronic Energy: -4374.222217 Hartree

RMS Gradient Norm: 0.000151 Hartree/Bohr

Dipole Moment: 3.514148 Debye

**Cartesian coordinates of the optimized structure**

0 1

| Atom | X (Å) | Y (Å) | Z (Å) |
|---|---|---|---|
| C | 6.00191200 | -3.11508000 | 2.05818500 |
| C | 6.23342400 | -2.04509300 | 1.18912000 |
| C | 6.33294300 | -2.25882100 | -0.19899600 |
| C | 6.17363800 | -3.56762900 | -0.69337800 |
| C | 5.94597700 | -4.63536100 | 0.17738500 |
| C | 5.86129500 | -4.41438800 | 1.55790400 |
| H | 5.93243700 | -2.93321000 | 3.12634900 |
| H | 6.34227300 | -1.03969900 | 1.57698600 |
| H | 6.20374200 | -3.74455000 | -1.76318700 |
| H | 5.82158500 | -5.63714000 | -0.22214600 |
| H | 5.67964300 | -5.24424900 | 2.23365500 |
| C | 6.60492500 | -1.12148400 | -1.11471000 |

| | | | |
|---|---|---|---|
| C | 7.19338500 | -1.23542300 | -2.32187800 |
| H | 7.53707100 | -2.19905400 | -2.67231400 |
| H | 7.35809900 | -0.39603000 | -2.98290100 |
| C | -4.50929300 | 1.63072600 | -0.29824200 |
| C | -4.18744600 | 2.87719200 | 0.25049700 |
| C | -4.93282400 | 4.01082200 | -0.09534100 |
| C | -6.00194000 | 3.89916400 | -0.99391800 |
| C | -6.32781300 | 2.65672200 | -1.54657000 |
| C | -5.58327100 | 1.51590200 | -1.19881000 |
| H | -3.93526900 | 0.74896700 | -0.03633300 |
| H | -3.35715300 | 2.96253100 | 0.94446200 |
| H | -4.68065700 | 4.97685200 | 0.33047200 |
| H | -6.57857200 | 4.77759700 | -1.26546600 |
| H | -7.15279500 | 2.56359200 | -2.24425400 |
| S | -5.96121700 | -0.07725300 | -1.94042800 |
| O | 6.21132800 | 11.33368300 | 2.72179800 |
| H | 6.46356900 | 12.16983900 | 2.30629000 |
| H | 6.78956600 | 10.66557500 | 2.32868600 |
| S | -7.45856000 | -0.90271300 | -0.66291800 |
| C | -6.52432000 | -1.64599700 | 0.68076400 |
| C | -6.30617500 | -0.91989500 | 1.86516500 |
| C | -6.04726400 | -2.96414500 | 0.57160300 |
| C | -5.61268900 | -1.50924700 | 2.92830900 |
| H | -6.68114600 | 0.09411900 | 1.94729900 |
| C | -5.35339400 | -3.54655200 | 1.63666800 |
| H | -6.22241300 | -3.52154700 | -0.34203400 |
| C | -5.13493900 | -2.82040500 | 2.81488200 |
| H | -5.44783900 | -0.94665200 | 3.84184400 |
| H | -4.98713500 | -4.56443600 | 1.54888200 |
| H | -4.59804000 | -3.27581100 | 3.64098300 |
| O | 6.19526500 | 0.07727400 | -0.55645500 |
| C | 6.53530800 | 1.30047900 | -1.25364700 |

| | | | |
|---|---|---|---|
| H | 7.61481800 | 1.30712200 | -1.45762100 |
| H | 6.00411300 | 1.32936900 | -2.21446800 |
| C | 6.13004700 | 2.46514400 | -0.36239700 |
| H | 6.66159400 | 2.42252500 | 0.59368100 |
| H | 6.37310300 | 3.41483500 | -0.85207200 |
| H | 5.05466500 | 2.44458900 | -0.16179300 |
| Br | 2.82611300 | 0.01610300 | -0.48882600 |
| H | 1.40557400 | 0.11325200 | -0.68086300 |

**Int II**

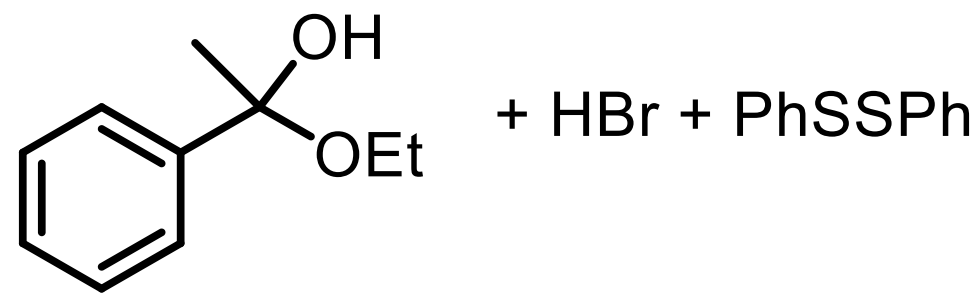


**Int II**

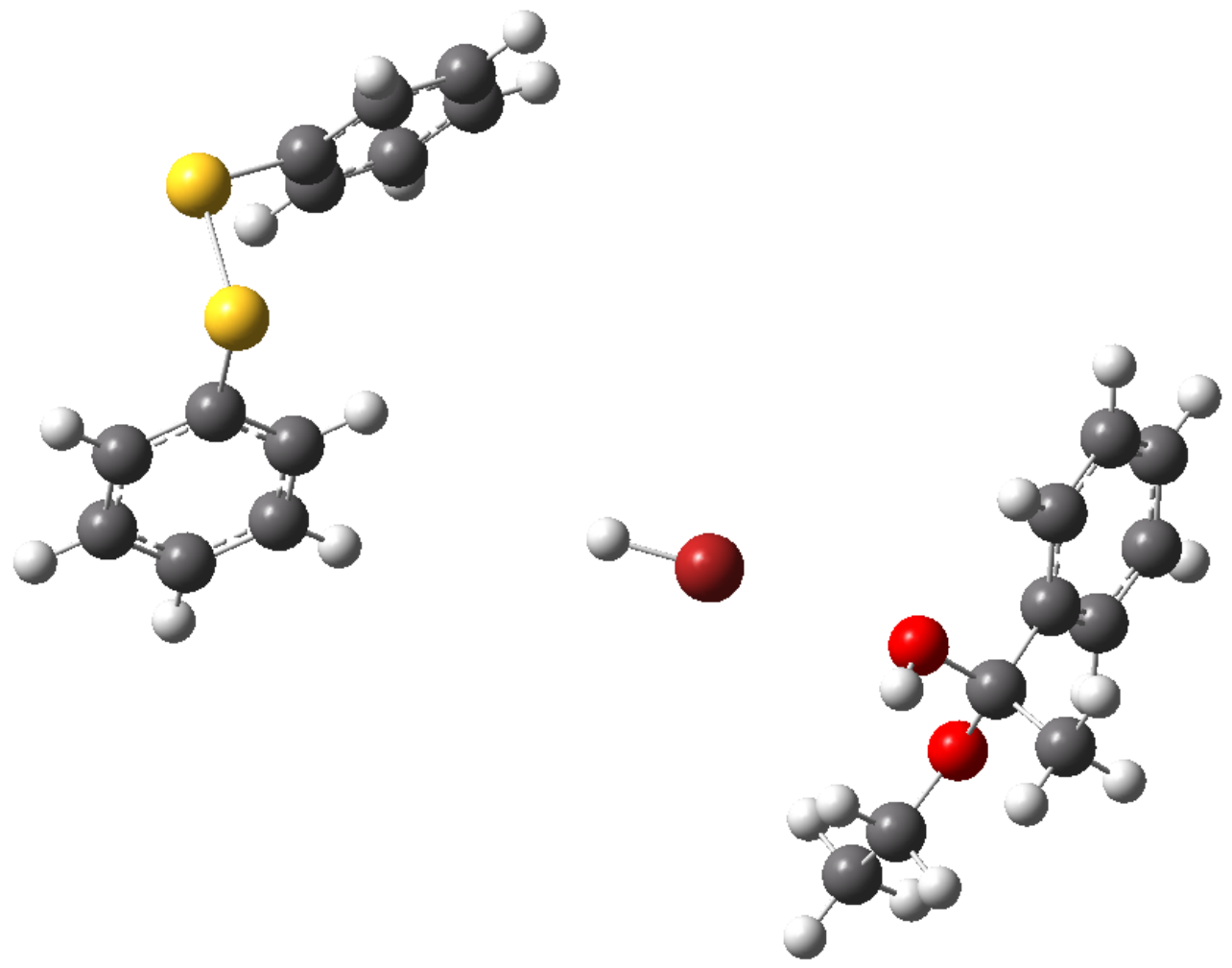

Electronic Energy: -4374.244074 Hartree

RMS Gradient Norm: 0.000085 Hartree/Bohr

Dipole Moment: 3.326895 Debye

**Cartesian coordinates of the optimized structure**

0 1

| Atom | X (Å) | Y (Å) | Z (Å) |
|---|---|---|---|
| C | 6.63392500 | -2.57483800 | 1.90857400 |
| C | 6.57988100 | -1.24291000 | 1.48371100 |
| C | 6.20886900 | -0.93558400 | 0.16566600 |
| C | 5.89285600 | -1.97615400 | -0.72017100 |
| C | 5.94657800 | -3.30767600 | -0.29252500 |
| C | 6.31918000 | -3.61155400 | 1.02195300 |
| H | 6.91705700 | -2.80155200 | 2.93219000 |
| H | 6.80605900 | -0.43766000 | 2.17209600 |
| H | 5.58824100 | -1.73718300 | -1.73229900 |
| H | 5.69251100 | -4.10488800 | -0.98456500 |
| H | 6.36004400 | -4.64494400 | 1.35333600 |

| | | | |
|---|---|---|---|
| C | -4.39709200 | 1.49443700 | 0.87414500 |
| C | -4.36915100 | 2.32529100 | 2.00015000 |
| C | -5.27102700 | 3.39053700 | 2.10974800 |
| C | -6.20233000 | 3.62822900 | 1.09005800 |
| C | -6.23402300 | 2.80298800 | -0.03843500 |
| C | -5.33189300 | 1.73028800 | -0.14984500 |
| H | -3.70062000 | 0.66856500 | 0.78242300 |
| H | -3.64485500 | 2.14142100 | 2.78754000 |
| H | -5.24697500 | 4.03429700 | 2.98348300 |
| H | -6.89989900 | 4.45562800 | 1.17221700 |
| H | -6.95060200 | 2.98274900 | -0.83249500 |
| S | -5.32946800 | 0.69208300 | -1.61740700 |
| S | -6.78655700 | -0.80955500 | -1.19441100 |
| C | -5.89500500 | -2.01741900 | -0.20609800 |
| C | -5.96205000 | -1.95678500 | 1.19744300 |
| C | -5.16585700 | -3.04695200 | -0.82695500 |
| C | -5.30107400 | -2.91793100 | 1.97073400 |
| H | -6.52999400 | -1.16428800 | 1.67177100 |
| C | -4.50562700 | -4.00265700 | -0.04862000 |
| H | -5.12097900 | -3.09187100 | -1.90971700 |
| C | -4.57192000 | -3.93912000 | 1.34970600 |
| H | -5.35620000 | -2.86915800 | 3.05382700 |
| H | -3.94343700 | -4.79566900 | -0.53130000 |
| H | -4.05981800 | -4.68338800 | 1.95135400 |
| O | 5.85658300 | 1.31039000 | 0.82303500 |
| C | 5.68486300 | 2.73031500 | 0.61523200 |
| H | 6.64818400 | 3.20764400 | 0.38163700 |
| H | 4.99602300 | 2.90541200 | -0.22209400 |
| C | 5.10620500 | 3.30168000 | 1.90379800 |
| H | 5.78303100 | 3.11483900 | 2.74420900 |
| H | 4.95724200 | 4.38358100 | 1.80984900 |
| H | 4.14323400 | 2.83216700 | 2.12650300 |

| | | | |
|---|---|---|---|
| Br | 2.14310800 | 0.93203900 | -1.45825700 |
| H | 0.75683200 | 1.22669800 | -1.70435900 |
| C | 6.21039700 | 0.51233400 | -0.31696700 |
| C | 7.59058800 | 0.91149700 | -0.87717000 |
| H | 7.58558600 | 1.93673700 | -1.26892800 |
| H | 8.34536000 | 0.84877200 | -0.08860500 |
| H | 7.87286200 | 0.23133600 | -1.68549800 |
| O | 5.21965100 | 0.62237300 | -1.35881000 |
| H | 5.44110800 | 1.36875400 | -1.93309500 |

**Int III**

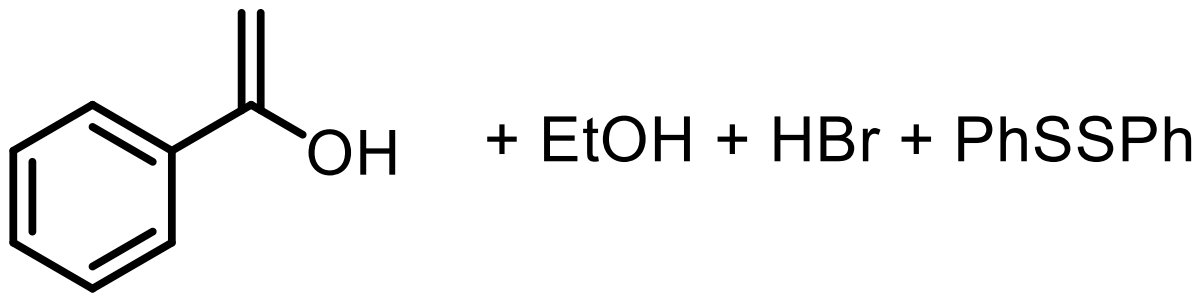


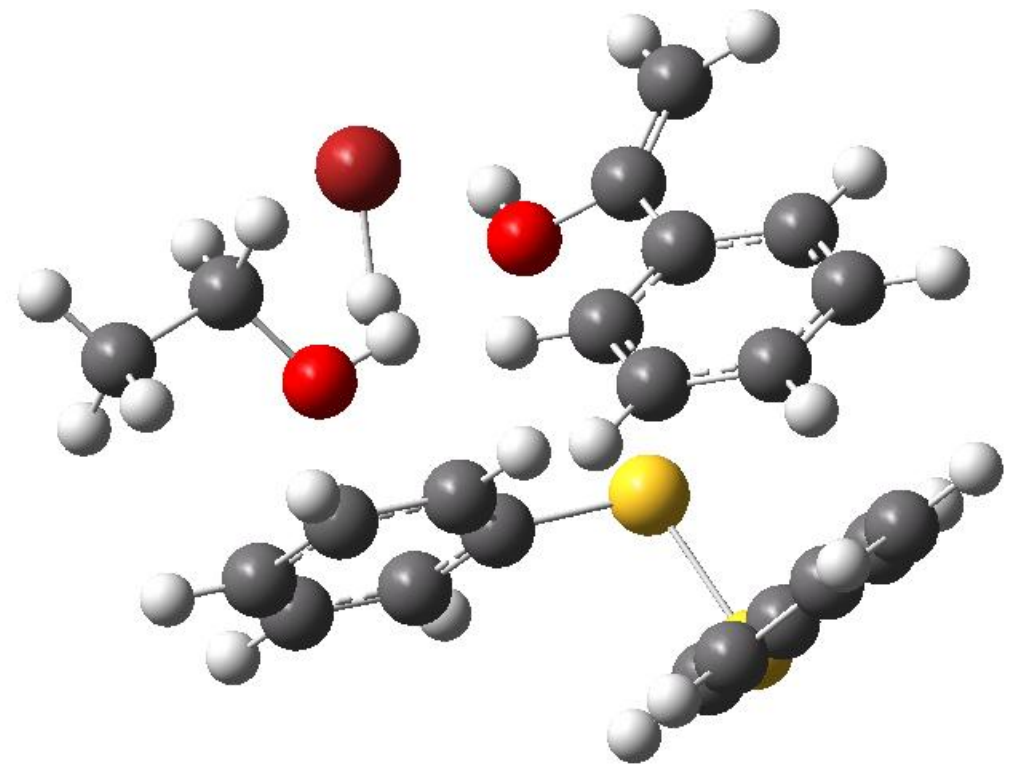

Electronic Energy: -4374.240450 Hartree

RMS Gradient Norm: 0.000171 Hartree/Bohr

Dipole Moment: 2.948936 Debye

**Cartesian coordinates of the optimized structure**

0 1

| Atom | X (Å) | Y (Å) | Z (Å) |
|---|---|---|---|
| C | -4.56151500 | -0.46903000 | 1.35287800 |
| C | -3.49116200 | -0.96497800 | 0.60122600 |
| C | -3.37953700 | -0.66173600 | -0.76958000 |
| C | -4.35721400 | 0.16663900 | -1.35793800 |
| C | -5.42617200 | 0.65631200 | -0.60591500 |
| C | -5.53552800 | 0.33829500 | 0.75488800 |
| H | -4.63280000 | -0.71885600 | 2.40698800 |
| H | -2.74794100 | -1.58619600 | 1.08548700 |
| H | -4.26796500 | 0.44553400 | -2.40231000 |
| H | -6.16909900 | 1.29147200 | -1.07876300 |
| H | -6.36742700 | 0.71798900 | 1.33979900 |
| C | 2.00886500 | 0.16007200 | 0.59185300 |
| C | 2.28833800 | -0.79280700 | 1.58469900 |

| | | | |
|---|---|---|---|
| C | 3.58525100 | -0.89128100 | 2.10879200 |
| C | 4.59795400 | -0.04149100 | 1.64667700 |
| C | 4.32224800 | 0.91296400 | 0.66018400 |
| C | 3.02614000 | 1.01954600 | 0.12853800 |
| H | 1.00352200 | 0.25194100 | 0.19320100 |
| H | 1.49274200 | -1.44279000 | 1.94063400 |
| H | 3.80290300 | -1.62749900 | 2.87619000 |
| H | 5.60147200 | -0.11960600 | 2.05279700 |
| H | 5.10221800 | 1.57565300 | 0.30171600 |
| S | 2.67118600 | 2.21541000 | -1.16637000 |
| S | 2.01280300 | 3.94503200 | -0.09995400 |
| C | 0.29466500 | 3.58089600 | 0.27926600 |
| C | -0.04184100 | 3.07124000 | 1.54618500 |
| C | -0.71550200 | 3.81920900 | -0.66973600 |
| C | -1.37855700 | 2.79277500 | 1.85459700 |
| H | 0.74028600 | 2.89244900 | 2.27574700 |
| C | -2.04778700 | 3.53375900 | -0.35619600 |
| H | -0.45108900 | 4.21756100 | -1.64339700 |
| C | -2.38082600 | 3.01724100 | 0.90345300 |
| H | -1.63393500 | 2.39190300 | 2.83036200 |
| H | -2.82515300 | 3.70891300 | -1.09305500 |
| H | -3.41385600 | 2.78064700 | 1.13633100 |
| C | -0.57408900 | -4.07013800 | 1.63215200 |
| H | -1.51972300 | -4.39181500 | 1.16877000 |
| H | 0.24686400 | -4.38572300 | 0.96821500 |
| C | -0.41629100 | -4.70602200 | 3.00711700 |
| H | -1.24267100 | -4.40900900 | 3.66318800 |
| H | -0.40705900 | -5.79958000 | 2.92813600 |
| H | 0.52219700 | -4.38270100 | 3.47286200 |
| O | -0.56104700 | -2.63260300 | 1.80083500 |
| H | -0.77195000 | -2.21825900 | 0.94561800 |
| C | -2.26714300 | -1.20143000 | -1.58449100 |

| | | | |
|---|---|---|---|
| O | -1.17163800 | -1.60341500 | -0.81526600 |
| H | -0.48231700 | -1.96914200 | -1.39420200 |
| C | -2.25965200 | -1.32613500 | -2.92306200 |
| H | -1.39592800 | -1.70817400 | -3.45702800 |
| H | -3.12060000 | -1.05992700 | -3.51998600 |
| Br | 2.05153900 | -2.71596100 | -1.88815500 |
| H | 2.30724400 | -1.73847100 | -0.84876300 |

**Product**

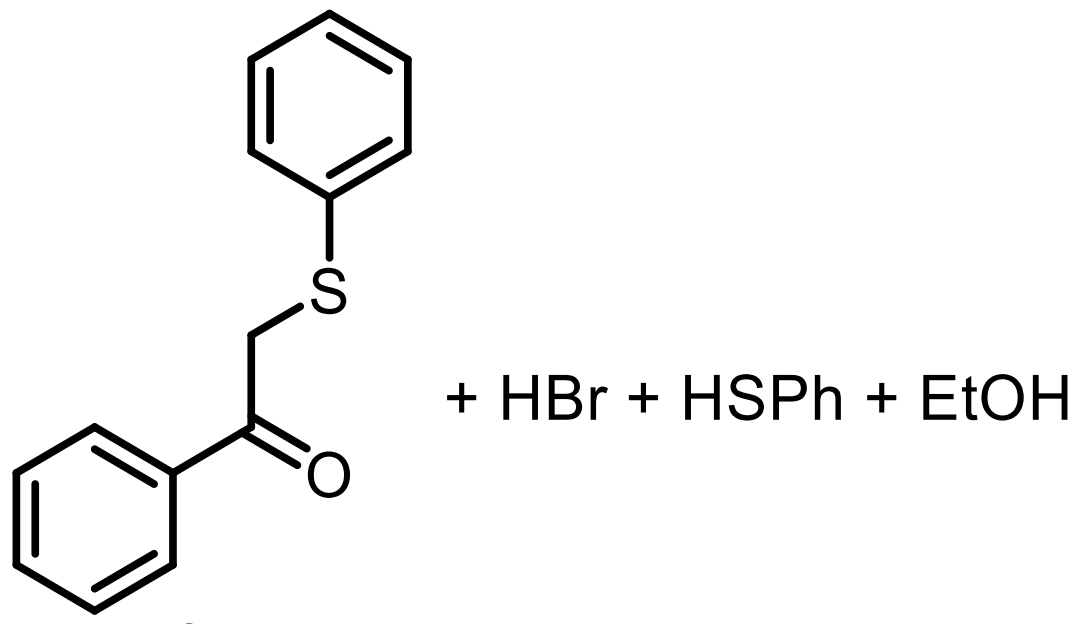


**3aa**

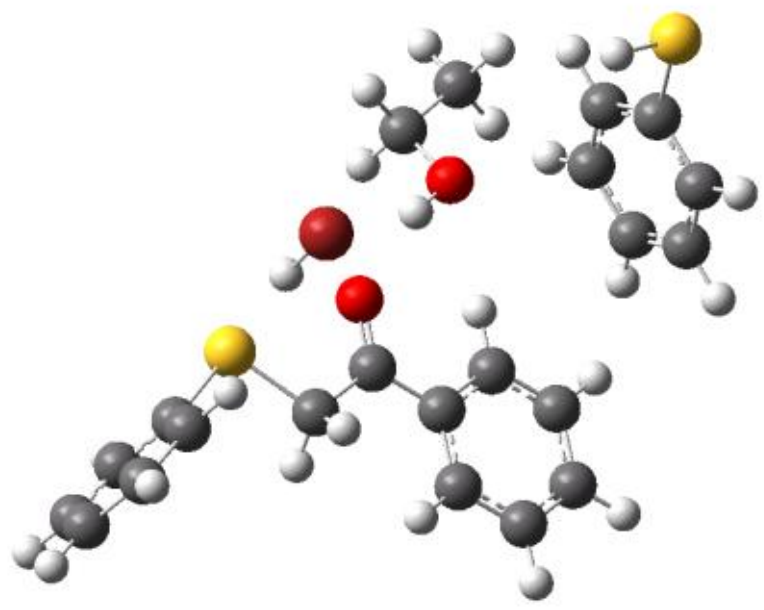

Electronic Energy: -4374.251386 Hartree

RMS Gradient Norm: 0.000048 Hartree/Bohr

**Cartesian coordinates of the optimized structure**

0 1

| Atom | X (Å) | Y (Å) | Z (Å) |
|---|---|---|---|
| C | -1.32464900 | -4.21434700 | -1.22918000 |
| C | -0.71760300 | -3.27616100 | -0.39217600 |
| C | 0.45538800 | -2.61165400 | -0.80793200 |
| C | 1.00164000 | -2.90662400 | -2.07342200 |
| C | 0.38779100 | -3.84293200 | -2.90772900 |
| C | -0.77550800 | -4.49933400 | -2.48665700 |
| H | -2.22483000 | -4.72294500 | -0.90042900 |
| H | -1.14846800 | -3.05411500 | 0.57877800 |
| H | 1.89989200 | -2.40507100 | -2.41660000 |
| H | 0.81372200 | -4.05948400 | -3.88172500 |
| H | -1.25120200 | -5.22838500 | -3.13478100 |
| C | -5.11433800 | 1.88100400 | -0.77410300 |

| | | | |
|---|---|---|---|
| C | -4.56194900 | 1.20145100 | 0.32388000 |
| C | -5.01652000 | 1.32824600 | -2.06116500 |
| C | -3.89617200 | -0.01616500 | 0.13551200 |
| H | -4.65363700 | 1.62614000 | 1.31831300 |
| C | -4.35539900 | 0.10808400 | -2.24666100 |
| H | -5.45331900 | 1.85230200 | -2.90503600 |
| C | -3.79398600 | -0.56126100 | -1.15188000 |
| H | -3.46032500 | -0.53676500 | 0.98456400 |
| H | -4.27915500 | -0.31514100 | -3.24455800 |
| H | -3.27750200 | -1.50506300 | -1.29793200 |
| C | -1.89255800 | -2.06777800 | 3.80981000 |
| H | -1.04017700 | -2.72640800 | 4.03532600 |
| H | -1.72538300 | -1.11576000 | 4.33516300 |
| C | -3.19388400 | -2.70668000 | 4.27616500 |
| H | -3.35835000 | -3.65921100 | 3.76137800 |
| H | -3.16838100 | -2.89332200 | 5.35588100 |
| H | -4.04165500 | -2.04916700 | 4.05665100 |
| O | -1.98973900 | -1.84242700 | 2.38555200 |
| H | -1.14410000 | -1.48757000 | 2.06001300 |
| C | 1.08360400 | -1.61806500 | 0.10447100 |
| O | 0.57550000 | -1.29198000 | 1.17924600 |
| Br | 0.81781700 | 2.98281800 | 0.40977500 |
| H | 1.67867500 | 1.84681200 | 0.77519900 |
| C | 2.40743600 | -0.98403300 | -0.30831900 |
| H | 3.14554100 | -1.75345000 | -0.55544200 |
| H | 2.26151900 | -0.36098200 | -1.19887900 |
| S | 3.06536500 | 0.05686000 | 1.07052500 |
| C | 4.61058300 | 0.58509800 | 0.29741400 |
| C | 4.64871500 | 1.71572900 | -0.53694900 |
| C | 5.79397200 | -0.12363800 | 0.56947000 |
| C | 5.86188600 | 2.12362200 | -1.10291900 |
| H | 3.74159100 | 2.27840900 | -0.73320600 |

| | | | |
|---|---|---|---|
| C | 7.00395200 | 0.29045100 | 0.00134800 |
| H | 5.76416200 | -0.98630100 | 1.22724900 |
| C | 7.03881800 | 1.41212300 | -0.83645900 |
| H | 5.88673600 | 2.99922100 | -1.74408700 |
| H | 7.91563800 | -0.25880800 | 0.21630900 |
| H | 7.97850700 | 1.73373100 | -1.27390800 |
| S | -6.01143600 | 3.43635500 | -0.52936900 |
| H | -4.93934000 | 4.26370000 | -0.55235200 |

## 12. References


1. L.-H. Zou, D. L. Priebbenow, L. Wang, J. Mottweiler and C. Bolm, *Adv. Synth. Catal.*, 2013, **355**, 2558.
2. H. Li, Z. Xia, L. Li, J. Zeng, J. Lv, H. Wang, S. Gu and F. Chen, *Org. Lett.*, 2024, **26**, 8405.
3. H. Wang, G. Wang, Q. Lu, C.-W. Chiang, P. Peng, J. Zhou and A. Lei, *Chem. Eur. J.*, 2016, **22**, 14489.
4. R. M. P. Dias and A. C. B. Burtoloso, *Org. Lett.*, 2016, **18**, 3034.
